# The location of the chemical bond. Application of long covalent bond theory to the structure of silica

**DOI:** 10.3389/fchem.2023.1123322

**Published:** 2023-02-16

**Authors:** Stephen A. Miller

**Affiliations:** The George and Josephine Butler Laboratory for Polymer Research, Department of Chemistry, University of Florida, Gainesville, FL, United States

**Keywords:** bonding, molecular structure, resonance, aromaticity, computational chemistry

## Abstract

Oxygen is the most abundant terrestrial element and is found in a variety of materials, but still wanting is a universal theory for the stability and structural organization it confers. Herein, a computational molecular orbital analysis elucidates the structure, stability, and cooperative bonding of α-quartz silica (SiO_2_). Despite geminal oxygen-oxygen distances of 2.61–2.64 Å, silica model complexes exhibit anomalously large O-O bond orders (Mulliken, Wiberg, Mayer) that increase with increasing cluster size—as the silicon-oxygen bond orders decrease. The average O-O bond order in bulk silica computes to 0.47 while that for Si-O computes to 0.64. Thereby, for each silicate tetrahedron, the six O-O bonds employ 52% (5.61 electrons) of the valence electrons, while the four Si-O bonds employ 48% (5.12 electrons), rendering the O-O bond the most abundant bond in the Earth’s crust. The isodesmic deconstruction of silica clusters reveals cooperative O-O bonding with an O-O bond dissociation energy of 4.4 kcal/mol. These unorthodox, long covalent bonds are rationalized by an excess of O 2*p*–O 2*p* bonding versus anti-bonding interactions within the valence molecular orbitals of the SiO_4_ unit (48 vs. 24) and the Si_6_O_6_ ring (90 vs. 18). Within quartz silica, oxygen 2*p* orbitals contort and organize to avoid molecular orbital nodes, inducing the chirality of silica and resulting in Möbius aromatic Si_6_O_6_ rings, the most prevalent form of aromaticity on Earth. This long covalent bond theory (LCBT) relocates one-third of Earth’s valence electrons and indicates that non-canonical O-O bonds play a subtle, but crucial role in the structure and stability of Earth’s most abundant material.

## Graphical Abstract

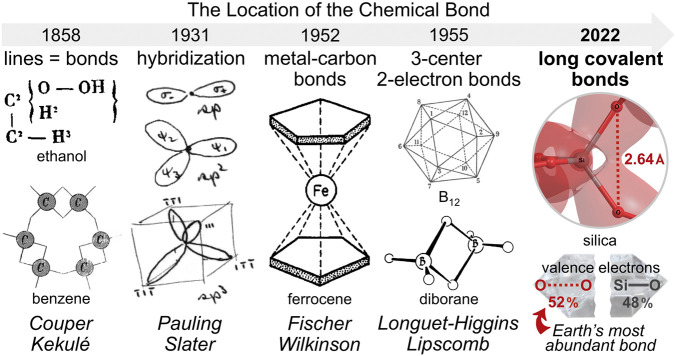

## 1 Introduction

**FIGURE 1:**
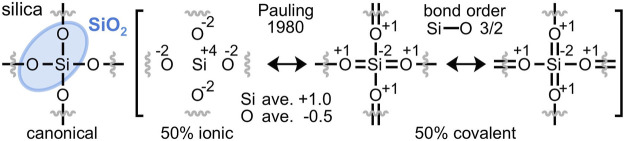
Pauling’s resonance hybrid model of α-quartz silica (SiO_2_) is 50% ionic and 50% covalent. Silicon-oxygen double bonds formally reduce silicon and oxidize oxygen, yielding averaged Si^+1^ and O^−0.5^ atomic charges and Si-O bond orders of 3/2 (Pauling, 1980). This resonance model is contradicted by modern computational investigations that discount silicon-oxygen double bonds in silica.

Silica (SiO_2_) constitutes about 59% of the Earth’s crust (Clarke and Washington, 1924) and its abundance is largely explained by its stability. By mass, silica is 53% oxygen, the most abundant element on Earth. A universal connection between oxygen and the material stability it bestows has not been described. For years, Pauling argued that a “molecule is stabilized by… resonance, its energy being less than the energy corresponding to any one of the structures among which it resonates.” (Pauling, 1946) “It is this extra stability of the system… that is called the *resonance energy.*” (Pauling, 1960) Although Pauling never quantified this value for silica (Torgunrud et al., 2020) his detailed resonance description of “low quartz”, illustrated in Figure 1, distinctly implies that it enjoys a resonance energy (Pauling, 1980).

The 50% ionic/50% covalent character of silica was ardently defended by Pauling and determined by the electronegativity difference of Δ*x* = 1.7 between oxygen (*x* = 3.5) and silicon (*x* = 1.8) (Pauling, 1952). With the premise of 50% ionic character and only silicon-oxygen single bonds, resonance hybrid averaging cannot achieve the charges of +1 on silicon and −0.5 on each oxygen, as limited by the electroneutrality postulate (Newton and Gibbs, 1980). However, Pauling’s silicon-oxygen double bonds introduce negative charge on silicon and positive charge on oxygen, allowing for resonance averaging to Si^+1^ and O^−0.5^ when the ionic character is 50%. Since each silicon atom forms two single bonds and two double bonds, Pauling concluded that the Si-O bond order in silica is 3/2, with the double bonds accommodated by *sp* ^3^ *d* ^2^ orbitals of silicon (Pauling, 1980). While a topic of considerable debate (Janes and Oldfield, 1986), recent experimental and computational results indicate that O 2*p*–Si 3*d* π-bonding is minimal in silica (Silvi et al., 1997; Garvie et al., 2000; Gibbs et al., 2009) and that Si-O bond orders are less than unity (Newton and Gibbs, 1980; Noritake and Kawamura, 2015; Torgunrud et al., 2020), contradicting Pauling’s resonance scheme and stated bond order. Without silicon-oxygen double bonds, the 50% covalent component defaults to the single silicon-oxygen bonds of the canonical resonance form. Resonance averaging that is 50% canonical and 50% ionic yields a net +2 charge on silicon and an obvious violation of the electroneutrality postulate.

Herein, an alternative resonance formulation for silica is presented that ameliorates these incongruities. Non-canonical, long covalent oxygen-oxygen bonds are proposed and rationalized—ostensibly necessitated—by a molecular orbital analysis of silica model complexes, thereby demystifying the structure, bonding, and stability of Earth’s most abundant material.

## 2 Results and discussion

### 2.1 Resonance hybrids with long bonds

**FIGURE 2:**
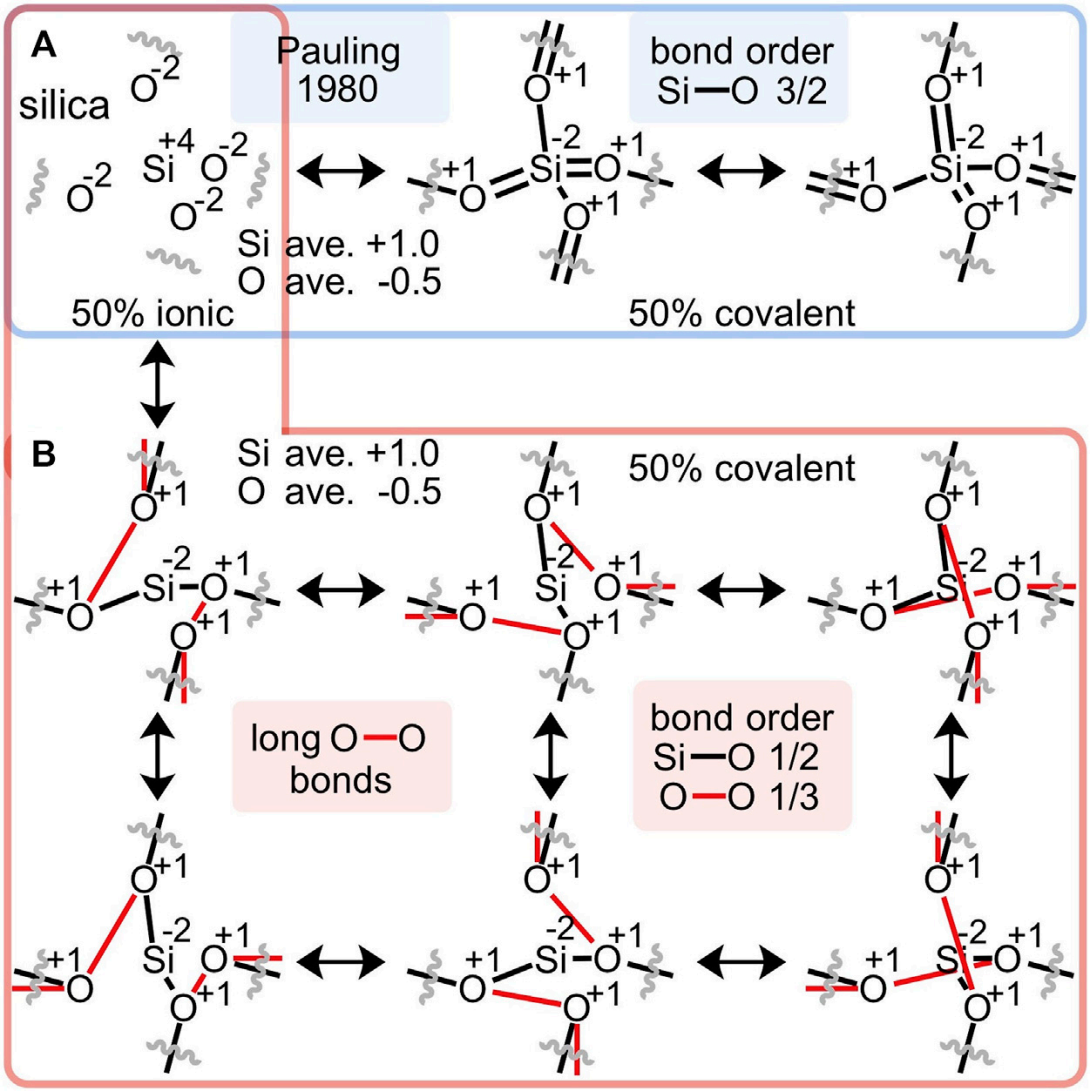
An alternative to Pauling’s resonance scheme for silica **(A)** proposes long oxygen-oxygen bonds, ranging from 2.61 to 2.64 Å **(B)**. This alternative conforms to Pauling’s 50% ionic/50% covalent apportionment, enlists the same average atomic charges and oxidation states, avoids unwarranted Si-O double bonds, suggests Si-O bond orders of 1/2 and geminal O-O bond orders of 1/3 and thus, employs equal numbers of Si-O and O-O valence electrons.

The ionic component of Pauling’s 1980 resonance scheme for silica (Figure 2A) employs oxidized silicon(IV) and reduced oxygen(-II); the covalent component employs reduced silicon(-II) and oxidized oxygen(I). An alternative covalent resonance formulation is proposed in Figure 2B, which assumes the same oxidation states, but a different arrangement of bonds. Instead of arguably discounted silicon-oxygen double bonds, long oxygen-oxygen bonds averaging 2.63 Å are enlisted. Each silicon-oxygen interaction comprises a 50% single bond and 50% no-bond (Roberts et al., 1950) resonance formulation; hence the predicted Si-O bond order is 1/2. Each geminal oxygen-oxygen interaction comprises a 33% single bond and 67% no-bond resonance formulation; hence the predicted O-O bond order is 1/3. Note that this resonance scheme involves an equal number of Si-O (4 = 4 x 2 x ½) and O-O (4 = 6 x 2 x ⅓) valence electrons.

### 2.2 Bond order analysis

This non-canonical O-O bond can be interrogated by a Mulliken bond order analysis (Mulliken, 1955a), which “characterizes the accumulation of the electrons in the region between the chemically bonded atoms, and is a very useful quantity often characterizing well the bond strength” (Mayer, 2007) and bond covalency (Mulliken, 1955b; Segall et al., 1996). The canonical silica structure employs 8 of its 16 valence electrons (= 4 + 6 + 6) to make four Si-O single bonds, leaving 4 lone pairs of non-bonding electrons on the oxygen atoms. Computed at the DFT/B3LYP/6-311++G** level of theory (Shao et al., 2015; Levien et al., 1980)^1^, silicic acid (Si(OH)_4_) appears nearly canonical with four Si-O bond orders averaging 1.03 (range = 1.01–1.04) and six oxygen-oxygen bond orders averaging an inconspicuous 0.05 (range = 0.04–0.06). Accordingly, each silicic acid drafts 8.21 valence electrons for Si-O bonding and 0.55 electrons for O-O bonding, totaling 8.77 valence bonding electrons and leaving 7.23 as non-bonding.

**FIGURE 3:**
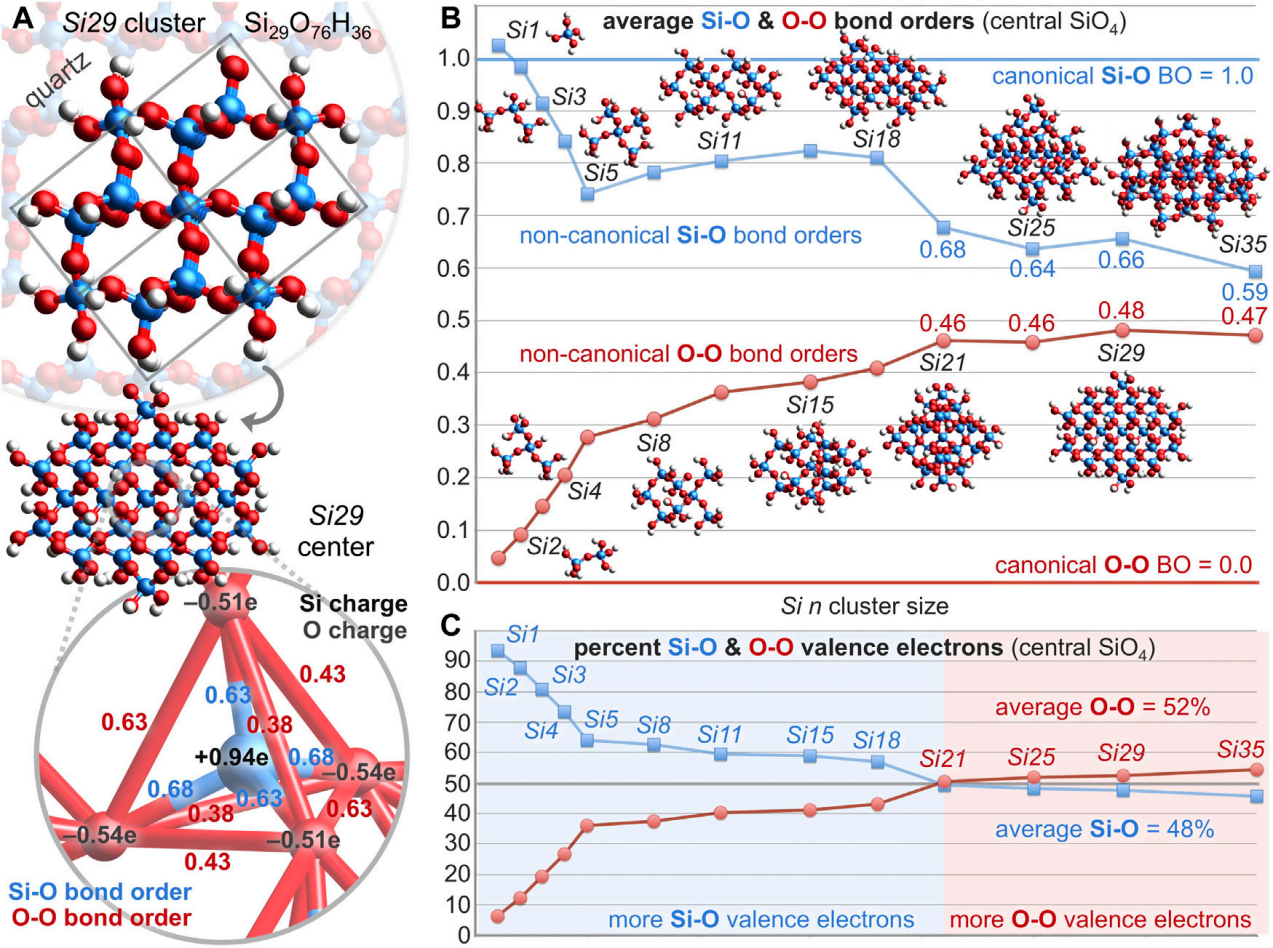
**(A)** For the central silicate of the quartz *Si29* cluster, the Si-O bond orders average 0.66 (blue) and the O-O bond orders average 0.48 (red). **(B)** For the central silicate of clusters ranging from *Si1* to *Si35*, average bond orders deviate substantially from the canonical bond order of 1.0 for Si-O (blue) and 0.0 for O-O (red). **(C)** For the central silicate of large clusters (*Si21*–*Si35*), more valence electrons belong to O-O bonds (52%) than Si-O bonds (48%)—indicating that the O-O bond is the most prevalent bond on Earth.

However, for the silicate at the center of a large 29-silicon cluster (*Si29*, Si_29_O_76_H_36_), where structural regularity is high and edge effects are low, the Si-O bond orders are substantially less than unity at 0.63, 0.63, 0.68, and 0.68, averaging 0.66 and requiring only 5.25 electrons. The O-O bond orders are conspicuously greater than zero at 0.38, 0.38, 0.43, 0.43, 0.63, and 0.63, averaging 0.48 and requiring 5.78 electrons—with these six O-O bonds occupying the edges of a silicate tetrahedron, as shown in Figure 3A. Thus, the central silicate of the *Si29* cluster employs 11.03 valence bonding electrons, leaving 4.97 electrons as non-bonding. Moreover, this central silicate enlists 5.23 (= 5.78–0.55) *additional* electrons for oxygen-oxygen bonding versus silicic acid. The electrostatic charge on silicon is +0.94e and the charges on the oxygen atoms are −0.51, −0.51, −0.54, and −0.54e, averaging −0.52e. The computed bond orders and atomic charges mirror the simplistic resonance hybrid averaging of Figure 2B.

Figure 3B plots the computed central silicate Si-O and O-O bond orders versus the number of silicon atoms in model quartz silica clusters ranging from *Si1* (silicic acid) to *Si35*. As the model complex is enlarged from *Si1* to *Si5*, the average O-O bond order increases about 0.05 for each additional silicate unit appended to the central silicate. Thereafter, the O-O bond order increases about 0.01 for each additional silicate—reaching 0.48 for *Si29* and 0.47 for *Si35* (presently the computational limit). The average Si-O bond order decreases with increasing cluster size—albeit with less regularity—reaching 0.66 for *Si29* and 0.59 for *Si35*. Note also that the Si 3*d* atomic orbital contribution to the valence molecular orbitals is consistently below 1% (*Si1*, 0.8%; *Si5*, 0.7%; *Si29*, 0.4%), further bolstering the premise that Si-O π bonding is minimal in silica.

For these same model clusters, Figure 3C plots the percentage of valence electrons belonging to the Si-O and O-O bonds of the central silicate unit. The O-O valence electron percentage climbs steeply to 36% (*Si5*) as the first shell of four silicates is added to the central silicate. The second shell accommodates twelve additional silicates, but their addition has a less dramatic effect. With 2–7 second-shell silicates (*Si8*–*Si18*), the O-O valence electron percentage spans 37%–43%. With 8–12 second-shell silicates (*Si21*–*Si35*), the percentage spans 51%–54%. These plots affirm a coherent dependence of bond order and valence electron apportionment on the size and structure of the silica model complexes. The averaged values for the largest group (*Si21*–*Si35*) indicate that only 48% of silica’s valence bonding electrons are allocated to silicon-oxygen bonds, while 52% are allocated to oxygen-oxygen bonds—insofar as the central silicate faithfully represents bulk silica. By this measure, *the oxygen-oxygen bond in silica is the most abundant bond on Earth*, either in number (six O-O bonds vs. four Si-O bonds) or by valence electron count (5.61 O-O electrons vs. 5.12 Si-O electrons, averaged over *Si21*–*Si35*). Long O-O bond ubiquity is further suggested by a similar analysis of alumina (Al_2_O_3_) (Kirfel and Eichhorn, 1990), the second most abundant material in the Earth’s crust (15%) (Clarke and Washington, 1924). In this case, maximum O-O bond orders increase from 0.13 to 0.20 to 0.32 for geminal oxygen atoms 2.72 Å apart in Al_8_O_12_, Al_12_O_18_, and Al_16_O_24_ clusters, respectively (see the Supplementary Material).

### 2.3 Molecular orbital analysis

Generally, atoms are not covalently bonded unless they experience an excess of bonding interactions versus anti-bonding interactions and thereby, “a concentration of charge between the two nuclei.” (Feynman, 1939) Similarly, Hoffmann stated succinctly that “positive overlap implies stabilization or bonding.” (Hoffmann, 1971) So, is there charge concentration and positive overlap between oxygen atoms of silica that account for the non-canonical O-O bond orders of Figure 3B?

**FIGURE 4:**
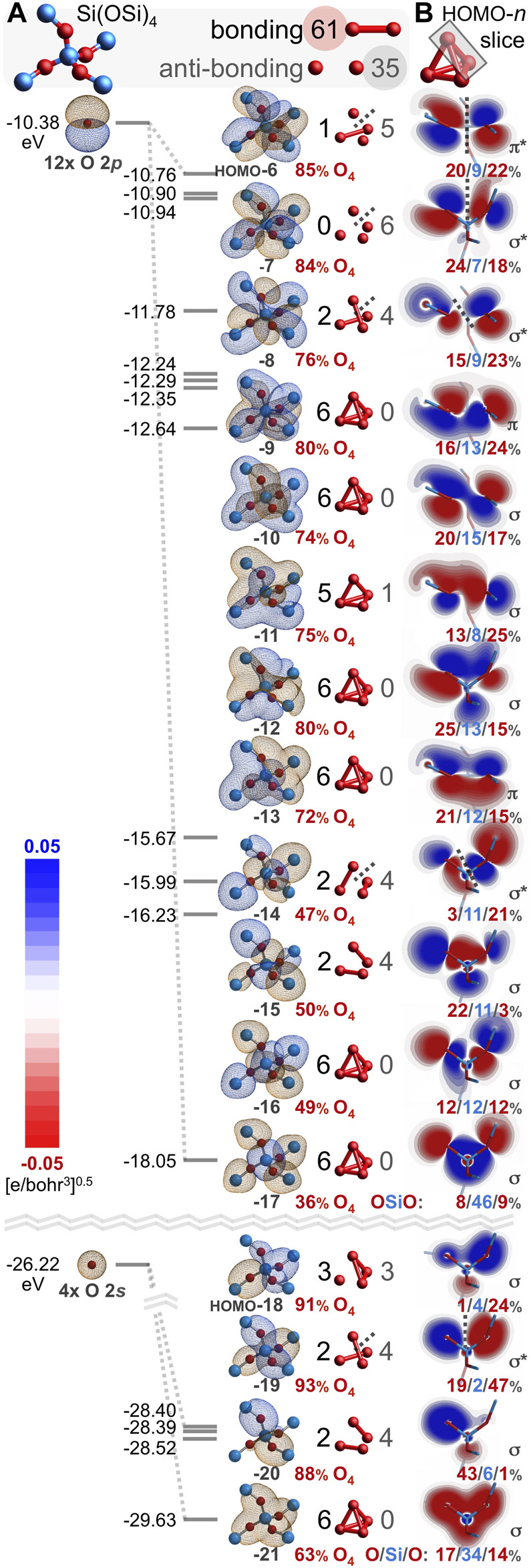
Oxygen-based valence molecular orbitals (IsoValue = 0.01 [e/bohr^3^]^0.5^) for Si(OSi)_4_ excised from quartz silica **(A)**. Bonding and anti-bonding O-O interactions are tabulated along with the oxygen atomic orbital contribution (red). Slices for each HOMO-*n* along the upper right O-O axis reveal excess bonding (11; σ and π) vs. anti-bonding (5; σ* and π*) interactions **(B)**. The corresponding O/Si/O atomic orbital contributions reveal the greatest O-O covalency for HOMO-13 through HOMO-9.

The repeat unit of silica can be considered as SiO_2_ or Si(**½**O)_4_ and the four oxygen atoms of the latter can be modeled by Si(OSi)_4_ to provide the molecular orbitals (MOs) of Figure 4A. Depicted are sixteen valence MOs built primarily from the four oxygen 2*s* orbitals and the twelve oxygen 2*p* orbitals (IsoValue = 0.01 [e/bohr^3^]^0.5^). The oxygen character is above 70% for eleven of these MOs, averages 71%, and exceeds the valence electron percentage contributed by oxygen, 55%^2^. The remaining six valence MOs (HOMO-5 through HOMO, not shown) are built primarily from silicon atomic orbitals and have a silicon character averaging 86%—well exceeding the valence electron percentage contributed by silicon, 45%. Clearly, there is disproportional and poor silicon/oxygen atomic orbital mixing and the oxygen-rich MOs rationalize a high degree of oxygen-oxygen covalency.

The six valence O-O interactions in each Si(OSi)_4_ MO can be categorized as bonding or anti-bonding^3^. These are illustrated and tabulated in Figure 4A. Of the 96 O-O interactions, 61 are bonding and 35 are anti-bonding; there are 26 excess bonding interactions. A *bonding excess* parameter can be defined as *be* = (bonding–anti-bonding)/(bonding + anti-bonding) and computes to *be*_O-O_ = 27% in Si(OSi)_4_. Among just the 24 O 2*s*–O 2*s* interactions, 13 are bonding and 11 are anti-bonding; there are 2 excess bonding interactions and *be*_2*s*_ = 8%. Among just the 72 O 2*p*–O 2*p* interactions, 48 are bonding and 24 are anti-bonding; there are 24 excess bonding interactions and *be*_2*p*_ = 33%. The greater *bonding excess* for the O 2*p* subset illuminates the importance of O 2*p* atomic orbitals vs. O 2*s* atomic orbitals for long covalent bonding. Collectively, there is a definitive excess of bonding interactions providing the net positive overlap that allows—or perhaps mandates—oxygen-oxygen covalent bonding. Accordingly, the O-O bond orders are decidedly greater than zero (0.07, 0.11, 0.13, 0.13, 0.14, 0.14) and average 0.12. The Si-O bond orders are decidedly less than unity (0.88, 0.88, 0.80, 0.79) and average 0.84.

The proclamation that “there are no chemical bonds, only bonded interactions,” (Gibbs et al., 2009) compels scrutiny of the Figure 4A MOs for electron density associated with O-O interactions. Figure 4B illustrates HOMO-*n* slices intersecting the upper right O-O axis of the O_4_ tetrahedron (bond order = 0.14). These planes contain Si or are perpendicular to that—depending on which shows greater overlap. Eleven of these sixteen interactions are bonding and five are anti-bonding; locally, this O-O relationship has *be*_O-O_ = 38%. Of the bonding interactions, nine utilize σ overlap and two utilize π overlap. Of the anti-bonding interactions, four are σ* and one is π*. The HOMO-13 through HOMO-9 slices reveal considerable O-O covalency, arising from three σ interactions and two π interactions. These MOs average an oxygen atomic orbital contribution of 76%—well above the 55% predicted by purely proportional silicon/oxygen atomic orbital mixing. This excess oxygen MO character portends high O-O covalency (bond order > canonical value of 0) and low Si-O covalency (bond order < canonical value of 1). The *bonding excess* and the abundance of O-O electron density, implied by Ψ^2^ of the HOMO-*n* slices, are further evidence of long bonded interactions in locations not previously described.

**FIGURE 5:**
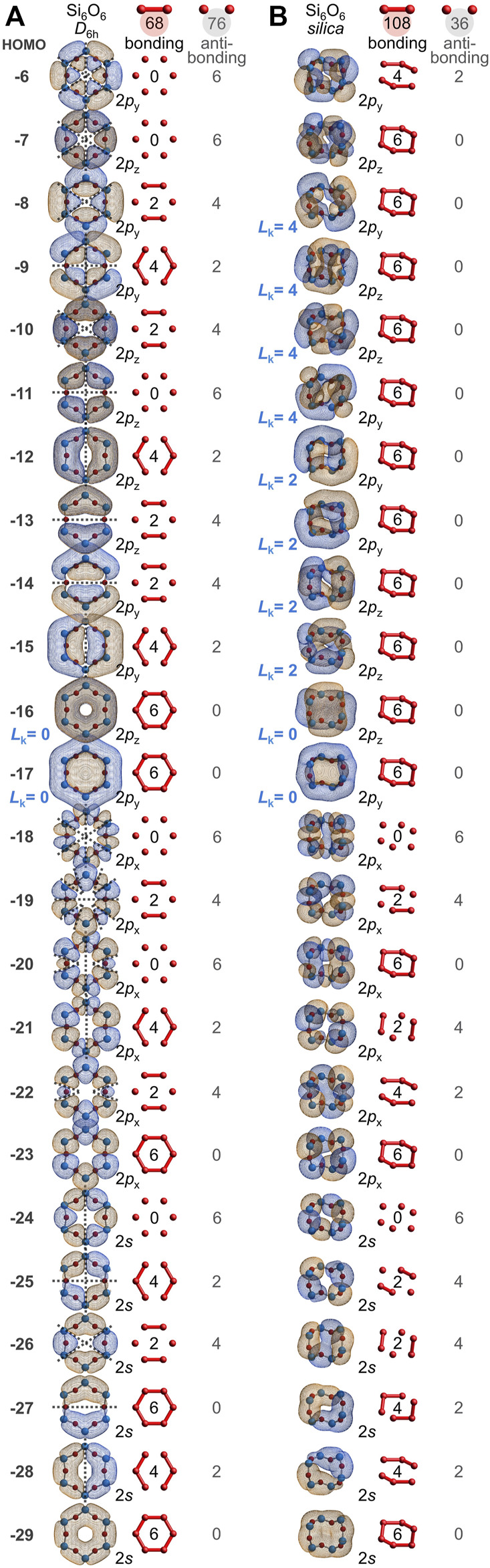
Oxygen-based valence molecular orbitals (IsoValue = 0.005 [e/bohr^3^]^0.5^) for geometry-optimized, planar *D*_6h_ Si_6_O_6_ **(A)** and for chiral, saddle-shaped Si_6_O_6_ found in quartz silica **(B)**. Bonding and anti-bonding O-O interactions are tabulated and contiguous π molecular orbitals are classified as either Hückel aromatic (*L*_k_ = 0) or Möbius aromatic (*L*_k_ = 2 or 4).

Every atom in quartz silica is part of the Si(**½**O)_4_ repeat unit. Additionally, every atom belongs to a twelve-membered ring of alternating silicon and oxygen atoms, Si_6_O_6_. It is illustrative to first consider the bonding arrangement of a geometry-optimized Si_6_O_6_ ring, which adopts a planar, *D*_6h_ conformation. Figure 5A depicts the twenty-four valence MOs built primarily from the six oxygen 2*s* orbitals and the eighteen oxygen 2*p* orbitals (IsoValue = 0.005 [e/bohr^3^]^0.5^). Of the 144 O-O interactions, 68 are bonding and 76 are anti-bonding; there are 8 excess anti-bonding interactions. Hence, the *bonding excess* parameter is negative: *be* = −6%. Accordingly, the computed O-O bond orders are also negative at −0.05 (Mulliken, 1955c). Note that the HOMO-16, HOMO-13, HOMO-12, HOMO-11, HOMO-10, and HOMO-7 mimic the six π MOs of benzene (also *D*_6h_), built from six C 2*p*_*z*_ atomic orbitals (Hoffmann, 1963). They possess the same number of π nodal planes (0, 1, 1, 2, 2, 3), mandating that half are bonding and half are anti-bonding. Naturally, there is no net π bonding when these six MOs are filled.

However, the Si_6_O_6_ ring of quartz silica is *not* planar. Instead, it contorts to a chiral, *C*_2_-symmetric saddle shape, yielding a dramatically different array of oxygen-based valence molecular orbitals, as illustrated in Figure 5B. Of the 144 O-O interactions, 108 are bonding and 36 are anti-bonding; there are 72 excess bonding interactions. Hence, the *bonding excess* parameter is large and positive: *be* = 50%. Among just the 108 O 2*p*–O 2*p* interactions, 90 are bonding, 18 are anti-bonding, and *be*_2*p*_ = 67%. Accordingly, the computed O-O bond orders are positive: 0.04, 0.04, 0.06, 0.06, 0.07, and 0.07, averaging 0.05. For comparison, the Si_6_O_6_ ring embedded in a larger Si_20_O_24_ cluster possesses 373 bonding and 203 anti-bonding O-O interactions, yielding a substantial *be* of 30% and O-O bond orders ranging from 0.13 to 0.33 and averaging 0.23. While there is no net oxygen-oxygen bonding in the planar *D*_6h_ Si_6_O_6_ ring, such long bonds prevail in the non-planar rings of silica.

**FIGURE 6:**
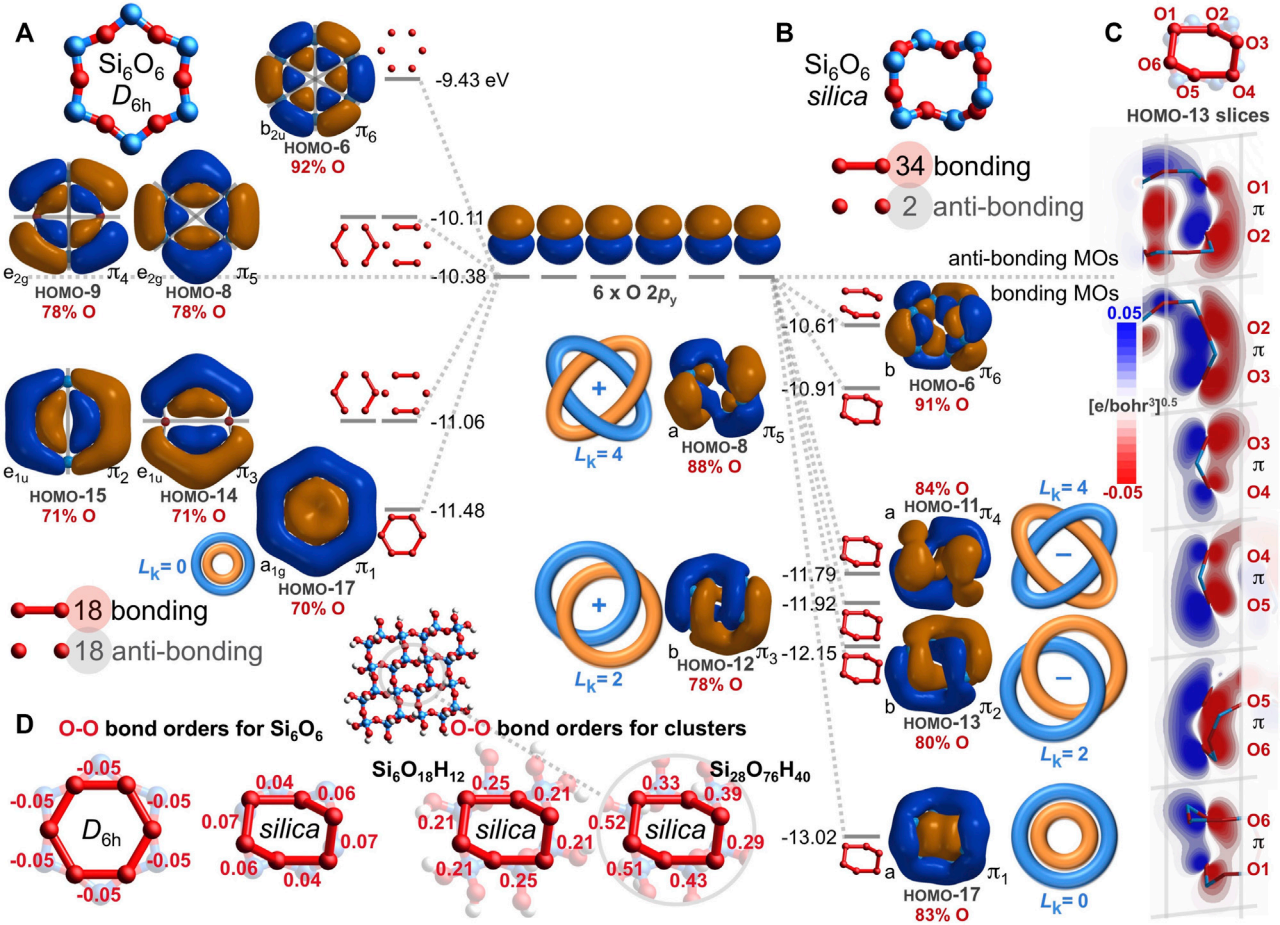
Oxygen 2*p*_y_-based valence molecular orbitals (IsoValue = 0.005 [e/bohr^3^]^0.5^) for geometry optimized, planar *D*_6h_ Si_6_O_6_ **(A)** and for chiral, saddle-shaped Si_6_O_6_ found in quartz silica **(B)**. Bonding and anti-bonding O-O interactions are tabulated along with the atomic orbital contributions for the six O atoms (red). Contiguous π molecular orbitals are classified as either Hückel aromatic (*L*_k_ = 0) or Möbius aromatic with two or four half-twists (*L*_k_ = 2 or 4). Oblique slices for the silica Si_6_O_6_ HOMO-13 along each O-O axis reveal contiguous O 2*p*_y_–O 2*p*_y_ π bonding interactions, resulting in Möbius aromaticity with *L*_k_ = 2 **(C)**. Oxygen-oxygen bond orders are negative for planar *D*_6h_ Si_6_O_6_ but positive for non-planar, silica-based Si_6_O_6_ and larger silica clusters **(D)**.

How can the deplanarization/contortion described in Figure 5 eliminate 40 anti-bonding O-O interactions and yield net O-O bonding? Nearly all of the eliminated nodes are lost from the HOMO-17 through HOMO-6; these twelve MOs derive mostly from the O 2*p*_y_ and O 2*p*_z_ atomic orbitals. Figure 6 compares and contrasts the O 2*p*_y_ MOs from the *D*_6h_ Si_6_O_6_ ring with those from the silica-based Si_6_O_6_ ring. The MOs for the planar ring have 18 bonding and 18 anti-bonding O-O interactions (Figure 6A). After warping to the chiral, saddle-shaped ring adopted by silica, O-O overlap greatly increases, resulting in 34 bonding and only 2 anti-bonding O-O interactions (Figure 6B). Inspection of these silica-based molecular orbitals reveals bonding, node-free, contiguous π MOs built mostly from oxygen atomic orbitals (78%–91%, averaging 84%) and minimally from silicon atomic orbitals (9%–22%, averaging 16%). Arguably, this constitutes a modified case of homoconjugation, which is defined as π overlap separated by a *single* non-conjugating atom or group (McNaught and Wilkinson, 1997). In the present case, the annular π-system spans *multiple* non-conjugating silicon atoms, being built from atomic orbitals of relatively distant (2.61–2.64 Å) and alternating oxygen atoms. Such an arrangement that nonetheless yields bonding molecular orbitals is herein defined as *alternoconjugation* (Hoffmann et al., 1970).

**FIGURE 7:**
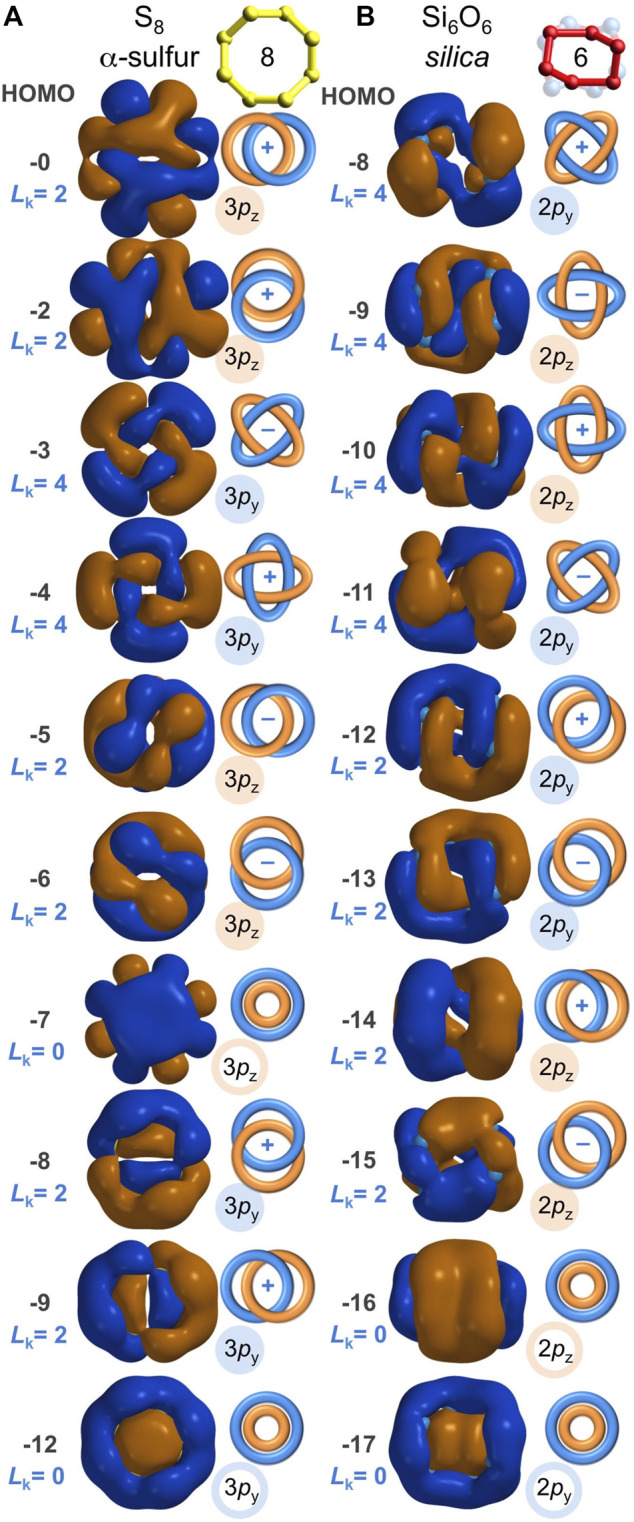
Analogous sulfur-based and oxygen-based valence molecular orbitals (IsoValue = 0.005 [e/bohr^3^]^0.5^) for α-sulfur S_8_ **(A)** and for Si_6_O_6_ found in quartz silica **(B)**. These molecular orbitals are built from contiguous *p* orbitals with a *bonding excess* of *be*_3*p*_ = 100% and *be*_2*p*_ = 100%, respectively, resulting in both Hückel (*L*_k_ = 0) and Möbius (*L*_k_ = 2 or 4) aromaticity. Their resemblance warrants equal expectations for covalent S-S bonding in sulfur and covalent O-O bonding in silica.

The HOMO-17 of Figure 6B is a twist-free π MO composed mostly (83%) of six O 2*p*_y_ orbitals pointing towards the middle of the Si_6_O_6_ silica ring. However, the HOMO-13 and HOMO-12 are contiguous π MOs with two half-twists and thus, have a topological linking number of *L*_k_ = 2 (Rappaport and Rzepa, 2008). Moreover, these MOs twist in opposite directions, resulting in quasi-enantiomorphic MOs (but not strictly enantiomorphic because the Si_6_O_6_ ring itself is chiral). The HOMO-11 and HOMO-8 are also contiguous π MOs, but with four half-twists and thus, a linking number of *L*_k_ = 4. These too are quasi-enantiomorphic because they twist in opposite directions. The HOMO-6 is apparently not a contiguous π MO because of a nodal surface bisecting the ring. Molecular orbitals with *L*_k_ = 2 or 4 are Möbius aromatic, although such twisting generally results in a smaller resonance energy (stabilization) compared to non-twisted (Hückel) analogues with *L*_k_ = 0 (Rzepa, 2005). For the HOMO-13, this Möbius aromaticity is further visualized by the six contiguous O 2*p*_y_–O 2*p*_y_ π bonding interactions of Figure 6C; these HOMO-13 slices are oblique but nonetheless lack a disruptive nodal plane. Figure 6 focuses on O 2*p*_y_ atomic orbitals, but there are also MOs built from O 2*p*_z_ atomic orbitals (Figure 5B and Figure 7B) that are Möbius aromatic: HOMO-15 and HOMO-14 with *L*_k_ = 2; HOMO-10 and HOMO-9 with *L*_k_ = 4. In total, eight of the 24 oxygen-based valence MOs are Möbius aromatic (HOMO-15 through HOMO-8). The HOMO-17 (O 2*p*_y_) and HOMO-16 (O 2*p*_z_) are twist-free π MOs with *L*_k_ = 0 and are thus Hückel aromatic.

Altogether, the Si_6_O_6_ ring of silica has ten total oxygen-based, contiguous π MOs (Figure 5B, HOMO-17 through HOMO-8), while the *D*_6h_ Si_6_O_6_ ring has only two (Figure 5A, HOMO-17 and HOMO-16). This difference largely explains the disparity in bonding/anti-bonding O-O interactions and thus, the negative O-O bond orders for the planar Si_6_O_6_ ring (−0.05) and the positive O-O bond orders (averaging 0.05) for the saddle-shaped Si_6_O_6_ ring based on silica (Figure 6D). These long bond orders are larger for the model silicate ring Si_6_O_18_H_12_ (averaging 0.22) and larger still for the central ring of Si_28_O_76_H_40_ (averaging 0.41). This molecular orbital analysis attests that the long covalent O-O bonds within quartz silica are significantly attributable to aromaticity, rendered by alternoconjugation of the oxygen atoms *via* uninterrupted O 2*p*_y_ or O 2*p*_z_ overlap. Moreover, this analysis claims Möbius aromaticity is the most prevalent kind of aromaticity on Earth since 25% of silica’s valence electrons belong to this category^4^.

### 2.4 Molecular orbital analysis of silica vs. sulfur

α-Sulfur is a stable chalcogen allotrope that consists of S_8_ rings with sulfur-sulfur bonding (Rettig and Trotter, 1987). Compared to the O-O bonds of silica (average = 2.63 Å), the S-S bonds are shorter (average = 2.05 Å) and sulfur shares no electrons with an electropositive element. Hence, the S-S Mulliken bond orders are typical of canonical single bonds, ranging from 1.13 to 1.18. A valence molecular orbital analysis of cyclic S_8_ (Figure 7A) reveals that its S-S bonds exist because of *p* atomic orbital overlap and not *s* atomic orbital overlap—akin to the O-O bonds of the silica Si_6_O_6_ ring. For the eight S 3*s*-based valence MOs, there are 32 bonding and 32 anti-bonding S-S interactions (*be*_3*s*_ = 0%). For the sixteen S 3*p*-based valence MOs, there are 128 bonding and 0 anti-bonding S-S interactions (*be*_3*p*_ = 100%). Collectively, *be* = 67%, allowing for net S-S bonding. Furthermore, as shown in Figure 7A and Supplementary Figure S5, ten of the sixteen 3*p*-based S_8_ MOs are aromatic, including: Hückel aromaticity for HOMO-12 (3*p*_y_) and HOMO-7 (3*p*_z_), with *L*_k_ = 0; Möbius aromaticity for HOMO-9 (3*p*_y_), HOMO-8 (3*p*_y_), HOMO-6 (3*p*_z_), HOMO-5 (3*p*_z_), HOMO-2 (3*p*_z_), and HOMO (3*p*_z_), with *L*_k_ = 2; and Möbius aromaticity for HOMO-4 (3*p*_y_) and HOMO-3 (3*p*_y_), with *L*_k_ = 4. Of the 24 valence MOs of S_8_, eight are Möbius aromatic and two are Hückel aromatic—the same aromatic MO representation found in the silica Si_6_O_6_ ring (Figure 7B). The chalcogen-chalcogen bonding in S_8_ has never been disputed. The chalcogen-chalcogen bonding in silica’s Si_6_O_6_ ring is equally evident and valid, given the molecular orbital and bonding parallels between S_8_ and the O_6_ ring embedded within silica.

### 2.5 Electron density analysis

**FIGURE 8:**
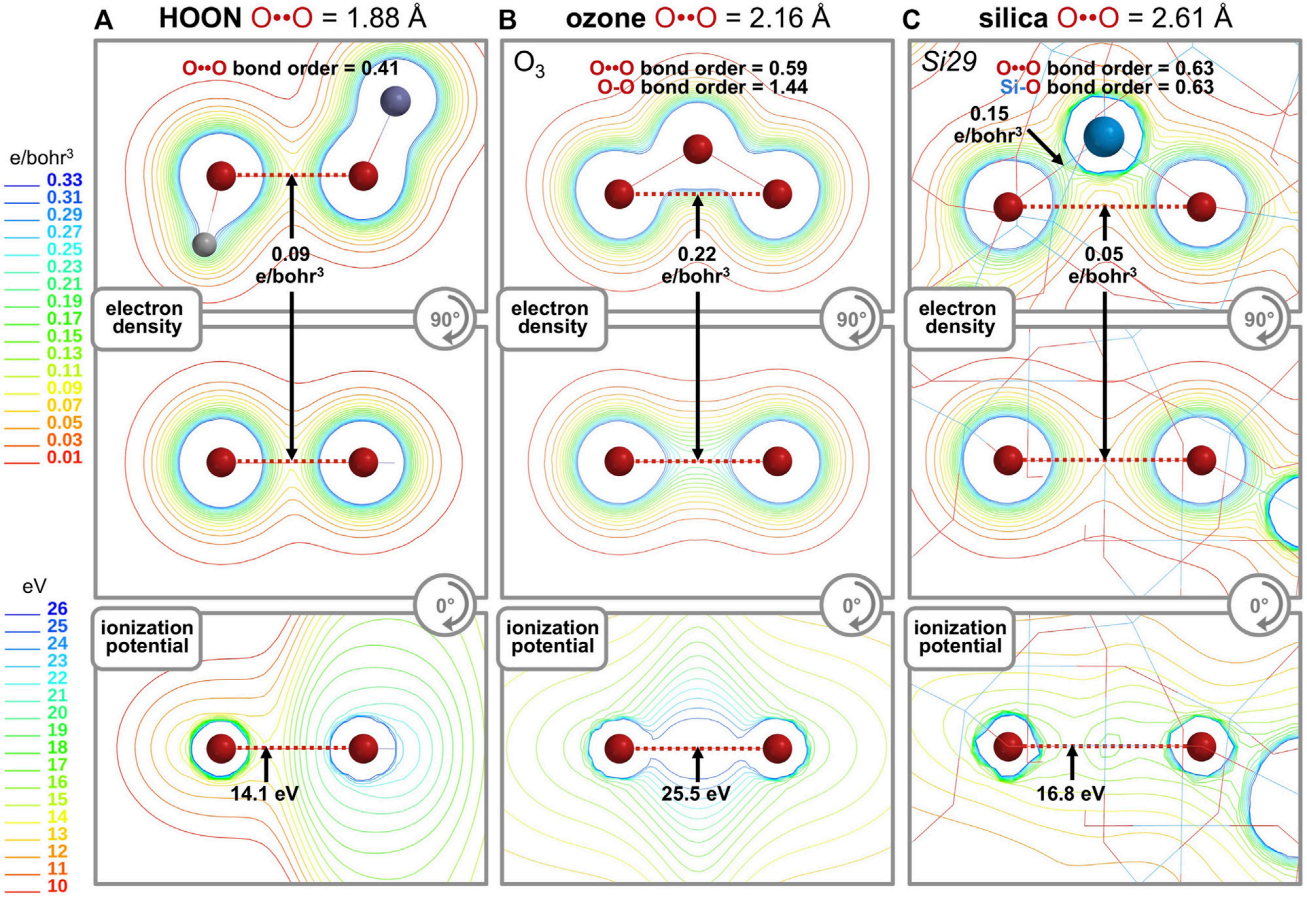
Computational analyses of HOON **(A)**, ozone **(B)**, and quartz silica **(C)** compare the O-O bond order, minimum O-O electron density (units of e/bohr^3^, core plus valence), and minimum O-O ionization potential (units of eV). Two orthogonal contour maps are shown for the electron density and a single contour map is shown for the ionization potential.

The “longest O-O bond in any known molecule” was reported for HOON, studied in a supersonic molecular beam. Its O-O distance was measured to be 1.91 Å (Jabłoński, 2012; Crabtree et al., 2013; Chen et al., 2016). A computational analysis (Figure 8A) shows its O-O bond order is 0.41, its minimum O-O electron density (ED, core plus valence) is 0.09 e/bohr^3^, and its minimum O-O ionization potential (IP) is 14.1 eV. This last parameter is an index of the MO energy, with a greater IP implying greater MO stability and more bonding between the atoms. The same computation (Figure 8B) for the terminal oxygen atoms (2.16 Å) of ozone (O_3_) (Hay et al., 1975) reveals a bond order of 0.59, a minimum O-O ED of 0.22 e/bohr^3^, and a minimum O-O IP of 25.5 eV. For an O-O bond (2.61 Å) in the center of the *Si29* cluster, the same computation (Figure 8C) reveals a bond order of 0.63, a minimum O-O ED of 0.05 e/bohr^3^, and a minimum O-O IP of 16.8 eV. Which one of these three long O-O bonds can claim the most bonding? A bond order comparison suggests the least O-O bonding in the canonical bond of HOON (0.41) and more O-O bonding in the long, non-canonical bonds of ozone (0.59) and silica (0.63).

**FIGURE 9:**
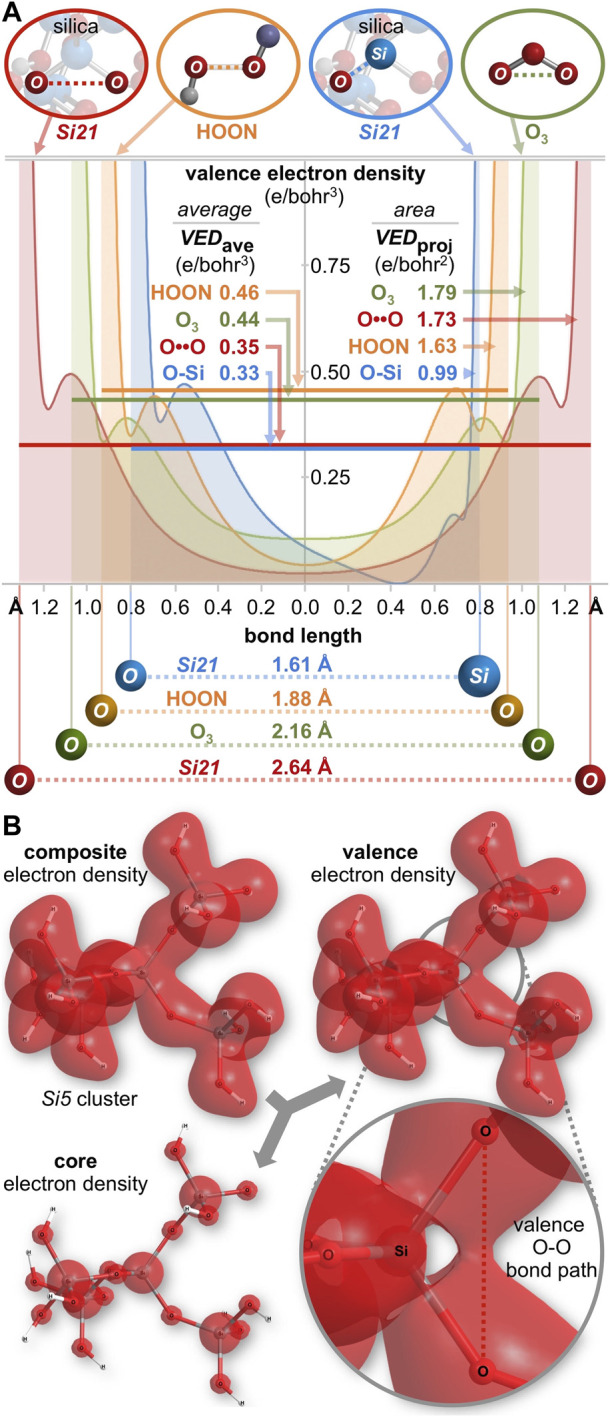
**(A)** Valence electron density along the O-O (or Si-O) bond axis for silica (*Si21* cluster), HOON, and ozone (O_3_). The average internuclear valence electron density is *VED*_average_, with units of e/bohr^3^. A superior metric for valence electron accumulation along the bond axis is the area under the curves, *VED*_projection_, with units of e/bohr^2^. For silica, *VED*_projection_ is greater for O-O (1.73 e/bohr^2^) than for O-Si (0.99 e/bohr^2^). **(B)** A three-dimensional valence electron density surface (IsoValue = 0.022 e/bohr^3^) for the *Si5* cluster (Si_5_O_16_H_12_) reveals an oxygen-oxygen valence bond path (*VED*_min_ = 0.025 e/bohr^3^), but no such path for silicon-oxygen (*VED*_min_ = 0.000 e/bohr^3^).

Bonding can also be gauged by the valence electron density (e/bohr^3^) along each O-O axis, as plotted in Figure 9A. Excluding core electron density^5^ the average valence electron density computes to *VED*_ave_ = 0.46, 0.44, and 0.35 e/bohr^3^ for HOON, O_3_, and the *Si21* cluster, respectively. The valence electron density can also be parameterized according to the area under the curves of Figure 9A. With units of e/bohr^2^ (= [e/bohr^3^] x [bohr]), this parameter measures valence electron accumulation, being a *projection* viewed down the O-O bond axis. This *bond axis valence electron density projection* computes to *VED*_proj_ = 1.63, 1.79, and 1.73 e/bohr^2^, respectively, revealing the largest value for ozone, but the smallest value for HOON. Consequently, there are more valence electrons along a central O-O bond axis in *Si21* than along that of HOON, indicating a new longest O-O bond of 2.64 Å belonging to quartz silica.

For comparison, analysis of a Si-O bond (1.61 Å) in the center of the *Si29* cluster (Figure 8C) reveals a bond order of 0.63, a minimum Si-O ED (core plus valence) of 0.15 e/bohr^3^, and a minimum Si-O IP of 18.9 eV. For the *Si21* cluster, the average Si-O valence electron density of *VED*_ave_ = 0.33 e/bohr^3^ (Figure 9A) is similar to that of its O-O bond (*VED*_ave_ = 0.35 e/bohr^3^)^6^. However, the shortness of the Si-O bond renders a minimal projection parameter of only *VED*_proj_ = 0.99 e/bohr^2^, which is markedly smaller than that of the long O-O bond of silica (1.73 e/bohr^2^). This difference can be further visualized by the three-dimensional valence electron density surface (IsoValue = 0.022 e/bohr^3^) of the *Si5* cluster (Si_5_O_16_H_12_), shown in Figure 9B. There is a clear oxygen-oxygen valence bond path along the O-O axis with a minimum valence electron density of *VED*_min_ = 0.025 e/bohr^3^ at the midpoint of this long bond. However, there is *no* corresponding silicon-oxygen valence bond path along the Si-O axis since the *VED*_min_ reaches 0.000 e/bohr^3^ approximately 0.4 Å from the silicon atom—corresponding to the outer nodal sphere of the Si 3*s* atomic orbital. This electron density analysis comports with the Mulliken population analysis (orbital-based bond orders) and further corroborates the supposition that silica’s valence electrons are not confined to their canonical locations.

### 2.6 Mulliken bond orders vs subsequent methods

Although Mulliken’s overlap population method “permits one to identify chemically bonded atoms,” it does not provide integral bond multiplicity values (corresponding to single, double, or triple bonds) (Mayer, 2003) and it is accompanied by other various objections (Reed et al., 1985). Historically, bond orders have been calculated *via* several generalized methods, including those described by Mulliken (1955b), Weinhold and Landis (2005), Wiberg (1968), Löwdin (1970), Mayer (1983), and Bader (1990)—although “none of them is the “right” one.” (Lewars, 2008) A comprehensive comparison is beyond the scope of this manuscript. However, a preliminary analysis demonstrates that the Mulliken, Wiberg, and Mayer methods yield comparably large O-O bond orders within silica clusters. These computed bond orders vary by only ±0.01 for a given silica cluster (see Supplementary Table S32) (Gorelsky and Lever, 2001)^7^. Furthermore, multicenter oxygen-based bond order indices have been computed and are proportional to silica cluster size. For the large *Si11*–*Si35* silica clusters, the 3-center 2-elecron (3c2e) bond order index ranges from *I*_OOO_ = 0.319 to 0.383; these values exceed that of *I*_BHB_ = 0.253 computed for diborane (B_2_H_6_) as well as the theoretical^7^ and computed value (Bochicchio et al., 1998) of 8/27 ≈ 0.296 for H_3_^+^. For the same large clusters, the 4-center 2-electron (4c2e) bond order index ranges from *I*_OOOO_ = 0.110 to 0.209; although such 4-center bonds are rare (de Giambiagi et al., 1997; Ponec and Mayer, 1997), these values mostly exceed that for B_4_ (*I*_BBBB_ = 0.118) (Sannigrahi and Kar, 1999) and the value of *I*_OOOO_ = 0.209 might be the largest 4c2e index reported to date. For comparison, multicenter bonding involving oxygen *and* silicon is uncommon within the silica clusters. There are no computed 3c2e *I*_OSiO_ values greater than 0.02 and only three clusters have 4c2e *I*_OSiOO_ values greater than 0.01, ranging from 0.023 to 0.141. This multicenter bonding analysis highlights the electron delocalization among oxygen atoms at the expense of silicon atom participation. An additional informative metric is the atomic valence (AV). For silicic acid (*Si1*), the Mulliken and Mayer atomic valencies are essentially canonical with Si_AV_ ≈ 4.33 and O_AV_ ≈ 2.05. However, in accord with the valence electron apportionment reported in Figure 3C, the Mulliken and Mayer atomic valencies invert for large silica clusters reaching, for example, Si_AV_ ≈ 3.14 and O_AV_ ≈ 4.35 for *Si29*—confirming the high atomic valence of oxygen markedly exceeding the canonical value of 2. The Löwdin method routinely yields smaller O-O bond orders near 0.10, invariant to the silica cluster size or the location within the cluster. The Natural Bond Order (NBO) method fails with all silica clusters because of their electronic delocalization (Goodman and Sauers, 2007). The NBO method also cannot determine long bond orders in the following molecules: the terminal oxygen atoms of ozone (Hay et al., 1975); the transannular *sp* ^2^ hybridized carbons of norbornadiene (Hoffmann et al., 1970); and the transannular sulfur atoms of cyclic S_4_N_4_ (Bridgeman et al., 2001). For silica, a substantial Mulliken O-O bond order is required to accord with the resonance scheme of Figure 2B (BO = 1/3). Without such long covalent bonds, Earth’s most abundant material fails to have a suitable resonance formulation. Not only did Mulliken deem his method “a good measure of covalent bonding,” (Mulliken, 1955b) he championed its “obvious” advantages for the interrogation of resonance and delocalized structures (Mulliken, 1955a).

### 2.7 Mulliken bond orders vs. computational methods and basis sets

Mulliken populations are often “unduly sensitive to basis set.” (Reed et al., 1985) Table 1 reports the Mulliken bond order sensitivity for the *Si5*, *Si11*, and *Si21* silica clusters subjected to various computational methods and basis sets. From the center of each cluster, the four Si-O bond orders and the six O-O bond orders are averaged to provide the values of Table 1. For the 6-311++G** basis set (used throughout this study), the bond order results are largely invariant to the computational method. Si-O bond orders are substantially less than unity and O-O bond orders are substantially greater than zero—converging as the cluster size increases and mimicking the plot for B3LYP/6-311++G** in Figure 3B. For the B3LYP method, basis set variation yields a broader range of bond orders. Pople-style basis sets (6-…) substantiate the claim of oxygen-oxygen bonding, although O-O bond orders (0.04–0.62) generally decrease with increasing basis set size. Dunning-style basis sets (cc-…) yield smaller O-O bond orders (0.00–0.30) and Ahlrichs/Weigend-style basis sets (def2-…) yield the broadest range of O-O bond orders (0.00–1.59). Note that the majority of computed Si-O bond orders are significantly below unity, arguing against the simplistic Lewis structure for silica having solely single Si-O bonds; negative Si-O bond orders are possibly an artifact of small cluster size and/or an indication of anti-bonding (Mulliken, 1955c). Without diffuse functionals in the basis set (see Supplementary Table S30), the Si-O bond order is near unity and the O-O bond order is near zero, yielding the simple Lewis structure having no option for resonance.

**TABLE 1:** Average computed Mulliken bond orders for the central SiO_4_ unit within the *Si5* (Si_5_O_16_H_12_), *Si11* (Si_11_O_32_H_20_), and *Si21* (Si_21_O_56_H_28_) clusters. Four bond order values are averaged for Si-O and six bond order values are averaged for O-O. (Not all computations converged).

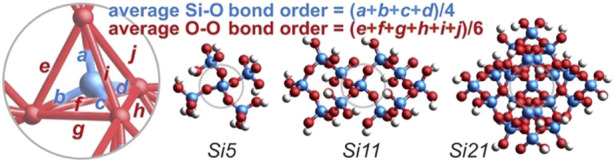							

Metdod	Basis set	*Si5*		*Si11*		*Si21*	
		Si-O	O-O	Si-O	O-O	Si-O	O-O
B3LYP	6-311++G**	0.74	0.28	0.80	0.36	0.68	0.46
B3LYP-D3	6-311++G**	0.74	0.28	0.80	0.36	0.68	0.46
HF	6-311++G**	0.65	0.33	0.77	0.41	0.63	0.50
ωB97X	6-311++G**	0.74	0.26	0.80	0.34	0.70	0.42
ωB97X-D	6-311++G**	0.76	0.27	0.83	0.35	0.71	0.44
ωB97X-V	6-311++G**	0.82	0.23	0.91	0.30	0.78	0.38
MP2	6-311++G**	0.65	0.33	0.78	0.41		
EDF2	6-311++G**	0.75	0.27	0.81	0.36	0.68	0.46
M06–2X	6-311++G**	0.74	0.27	0.75	0.35	0.59	0.46
VV10	6-311++G**	0.79	0.25	0.81	0.33	0.67	0.43
B97-D2	6-311++G**	0.80	0.27	0.89	0.34	0.73	0.44
ωB97X-V	6-311++G(2df,2p)	1.23	0.10	1.38	0.16	1.25	0.23
B3LYP	6-31+G	−0.13	0.52	0.55	0.36	0.79	0.13
B3LYP	6-31++G**	0.01	0.57	0.83	0.42	1.22	0.13
B3LYP	6-311++G(2d,p)	0.37	0.34	0.37	0.53	0.28	0.62
B3LYP	6-311++G(2df,2p)	1.25	0.10	1.38	0.18	1.24	0.25
B3LYP	6-311++G(3df,2p)	1.06	0.04	0.94	0.12		
B3LYP	aug-cc-pVDZ	−0.48	0.00	−0.27	0.06	−0.01	0.30
B3LYP	aug-cc-pVTZ	0.41	0.22	0.53	0.07		
B3LYP	aug-cc-pVQZ	0.43	0.12				
B3LYP	def2-SVPD	−2.61	1.59	0.89	0.63	0.50	0.21
B3LYP	def2-TZVPD	0.80	0.01	0.63	0.11		
B3LYP	def2-QZVPD	1.16	0.00				

### 2.8 Computed oxygen-oxygen bond dissociation energy for silica

**FIGURE 10:**
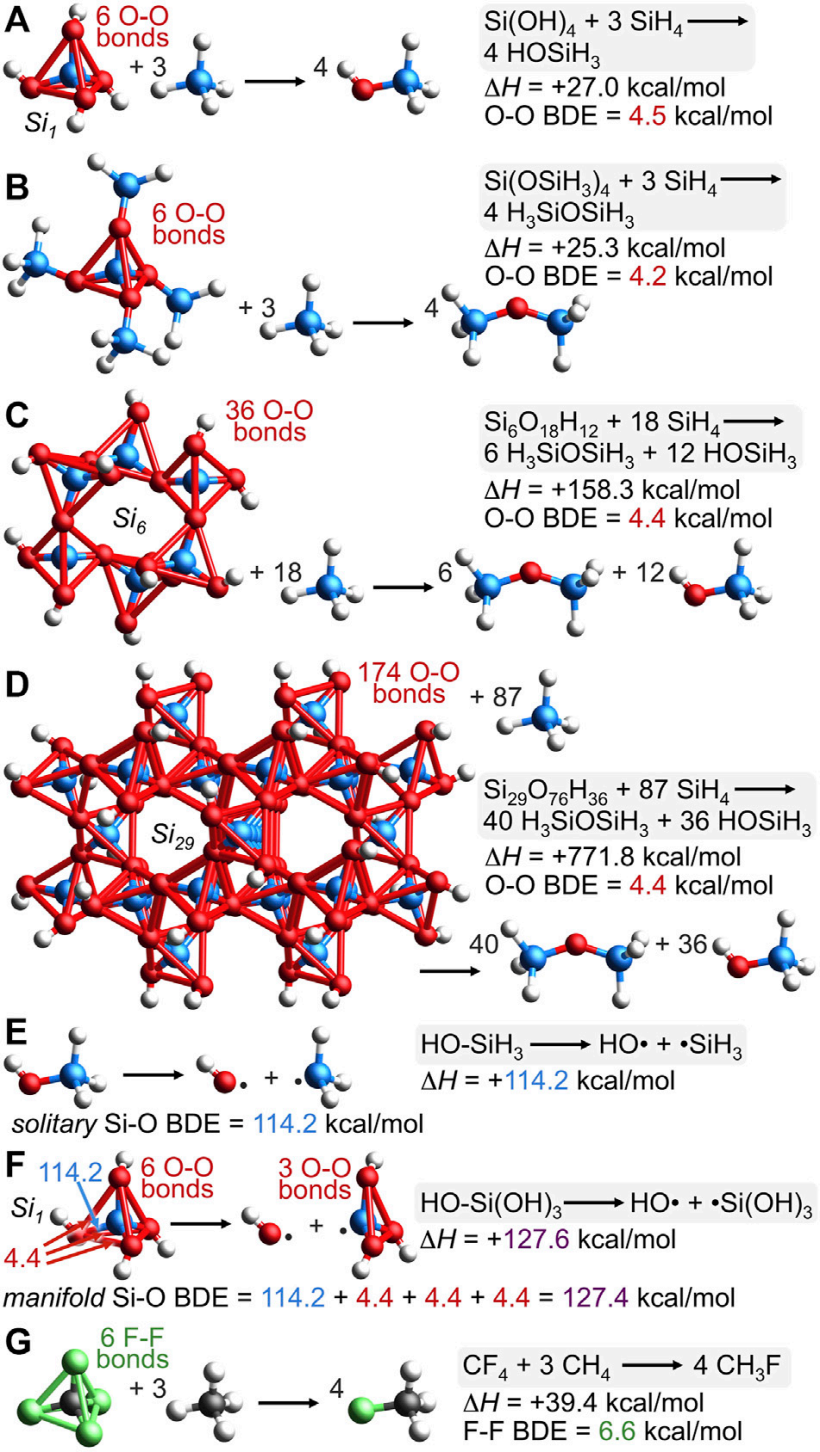
The conventionally isodesmic deconstruction of various silica clusters **(A–D)** reveals a substantial cooperative bonding energy that suggests an O-O bond dissociation energy (BDE) of 4.4 kcal/mol. This O-O BDE, combined with the *solitary* Si-O BDE of reaction **(E)**, 114.2 kcal/mol, predicts the *manifold* Si-O BDE of silicic acid (*Si1*) to be 127.4 kcal/mol; this value represents the homolytic cleavage of one Si-O bond *and* three O-O bonds and matches the computed enthalpy of homolytic reaction **(F)**, Δ*H* = +127.6 kcal/mol. The isodesmic deconstruction of CF_4_ **(G)** reveals cooperative F-F bonding in CF_4_ of 39.4 kcal/mol, suggesting an F-F BDE of 6.6 kcal/mol.

If oxygen-oxygen bonding in silica is real, then perhaps an apt computational analysis could reveal silica’s O-O bond dissociation energy (BDE). Conventionally, the reaction of Figure 10A between Si(OH)_4_ and 3 SiH_4_ is an isodesmic (Ponomarev and Takhistov, 1997) and homodesmotic (Wheeler, 2012) reaction since 4 Si-O bonds, 12 Si-H bonds, and 4 O-H bonds are maintained along with no hybridization changes. Unconventionally, however, six long O-O bonds in Si(OH)_4_ are broken to yield 4 molecules of HOSiH_3_ for which O-O bonding is not possible. The computed enthalpy of this reaction is Δ*H* = +27.0 kcal/mol; dividing by six suggests a geminal O-O BDE value of 4.5 kcal/mol. Concerns about hydrogen bonding within Si(OH)_4_ are eliminated by the reaction of Figure 10B (also conventionally isodesmic and homodesmotic) because it has no possibility of hydrogen bonding, but has a similar reaction enthalpy of Δ*H* = +25.3 kcal/mol and an O-O BDE value of 4.2 kcal/mol. Oxygen-oxygen BDE values for larger silica clusters, *Si6* and *Si29*, are computed *via* the conventionally isodesmic reactions of Figures 10C, D. In both cases, the O-O BDE values compute to 4.4 kcal/mol. Note that Si(OSiH_3_)_4_, the *Si6* cluster, and the *Si29* cluster possess siloxane oxygens (Si-O-Si)—thus requiring the production of H_3_SiOSiH_3_ to maintain the number and kind of bonds. If there were no cooperative bonding among the oxygen atoms of Si(OH)_4_, Si(OSiH_3_)_4_, or the silica clusters, then these isodesmic reaction enthalpies (Figures 10A–D) should be zero. However, the cooperative stabilization is substantial and the energetic cost for breaking a long O-O bond in silica averages to 4.4 kcal/mol.

Figure 10D illustrates the conventionally isodesmic deconstruction of the *Si29* cluster. The dendriform array of 174 O-O bonds stabilizing this cluster engenders a new term to describe the pervasive O-O bonding that supplements the conventional Si-O bonding: *dendrivalent*. The 8 bonds emanating from a single oxygen atom (six to oxygen and two to silicon) simultaneously depict the bonds of multiple resonance forms, none being more than trivalent at oxygen (Figure 2B). This number of bonds will not alarm anyone fluent in resonance formulations. Such multivalent bonding is commonplace, as in the 6 bonds emanating from carbon in the resonance hybrids of the carbonate dianion (3 sigma and 3 pi) or the 9 bonds emanating from carbon in the resonance hybrids of polycyclic aromatic hydrocarbons or graphite (3 sigma, 3 pi, and arguably 3 transannular bonds of the Dewar benzene type (Sorella et al., 2014; Pauling and Wheland, 1933);^8^ see Supplementary Figures S19–S21 for these multiple resonance hybrids). Inspection of valence electron density surfaces for *Si5* or *Si21* reveals clear valence bond paths between oxygen and all six of its oxygen neighbors (Figure 9B; Supplementary Figures S14–S17). As noted above, the outer spherical node of the Si 3*s* orbital precludes silicon-oxygen valence bond paths (Figure 9, *VED*_min_ = 0.000 e/bohr^3^).

While silica employs more O-O valence electrons than Si-O valence electrons according to Figure 3C, the greater O-O distance ensures that the O-O BDE of 4.4 kcal/mol is much less than that of Si-O, which computes to 114.2 kcal/mol according to the homolytic bond cleavage of HO-SiH_3_, depicted in Figure 10E. Furthermore, this *solitary* Si-O BDE in HO-SiH_3_ can be used to predict the *manifold* Si-O BDE in HO-Si(OH)_3_, which should cost 114.2 kcal/mol plus three times the aforementioned O-O BDE value since such a reaction breaks one Si-O bond and three O-O bonds, as depicted in Figure 10F. The prediction is [114.2 + 4.4 + 4.4 + 4.4] kcal/mol or 127.4 kcal/mol, which is very close to the enthalpy computed for the homolytic bond cleavage of HO-Si(OH)_3_, Δ*H* = +127.6 kcal/mol.

The similarity of these values indicates that a consistent and predictive bond energy additivity scheme can be formulated by proper inclusion of both long and short covalent bonds. For example, the greater C-F bond strength in CF_4_ (130.5 kcal/mol) versus that in CH_3_F (109.9 kcal/mol) has been rationalized by electronegativity and atomic charge effects (Lemal, 2004). However, a bond energy additivity scheme, analogous to that for silica in Figure 10, provides a simpler explanation. The computational isodesmic reaction of CF_4_ + 3 CH_4_ → 4 CH_3_F has Δ*H* = +39.4 kcal/mol (Figure 10G), suggesting an F-F BDE of 6.6 kcal/mol since this reaction breaks six long (2.17 Å), unconventional F-F bonds. Separately, the *manifold* C-F BDE in F-CF_3_, which represents one C-F bond and three F-F bonds, is predicted by adding the experimental *solitary* C-F BDE in F-CH_3_ to three F-F BDE values: [109.9 + 6.6 + 6.6 + 6.6] kcal/mol = 129.7 kcal/mol. This result is strikingly close to the experimental value of 130.5 kcal/mol and does not invoke nebulous arguments about electronegativity/atomic charge (Lemal, 2004), negative hyperconjugation (Hine, 1963; Reed and von Ragué Schleyer, 1987), or Coulombic interactions (Wiberg and Rablen, 1993), but elaborates on double bond/no-bond resonance formulations (Dolbier et al., 1982) by adding six new resonance hybrids, each with a long F-F bond, as in [F-C-F][F-F] (explicitly drawn in Supplementary Figure S22). A valence molecular orbital analysis corroborates the F-F bond in this unconventional resonance hybrid for CF_4_ since *be*_2*s*_ = 0% and *be*_2*p*_ = 39%, yielding a composite *bonding excess* greater than zero at *be*_F-F_ = 29%, which is rather close to *be*_O-O_ = 27% for the silicic acid model Si(OSi)_4_ (Figure 4), or *be*_O-O_ = 21% for silicic acid itself. To recapitulate: CF_4_ and silica clusters have a substantial excess of bonding versus anti-bonding F-F or O-O interactions that mandate an F-F or O-O bond dissociation energy of non-zero magnitude. While these long BDE values are small compared to those of short bonds, there are six per tetrahedron and thus, they stabilize CF_4_ by 39.4 kcal/mol and silica clusters by 26.4 kcal/mol of SiO_2_—thereby augmenting the measured strength of *manifold* C-F and Si-O bonds to values considerably greater than those of *solitary* C-F and Si-O bonds.

### 2.9 Ball-and-stick model vs cooperative sphere model

**FIGURE 11:**
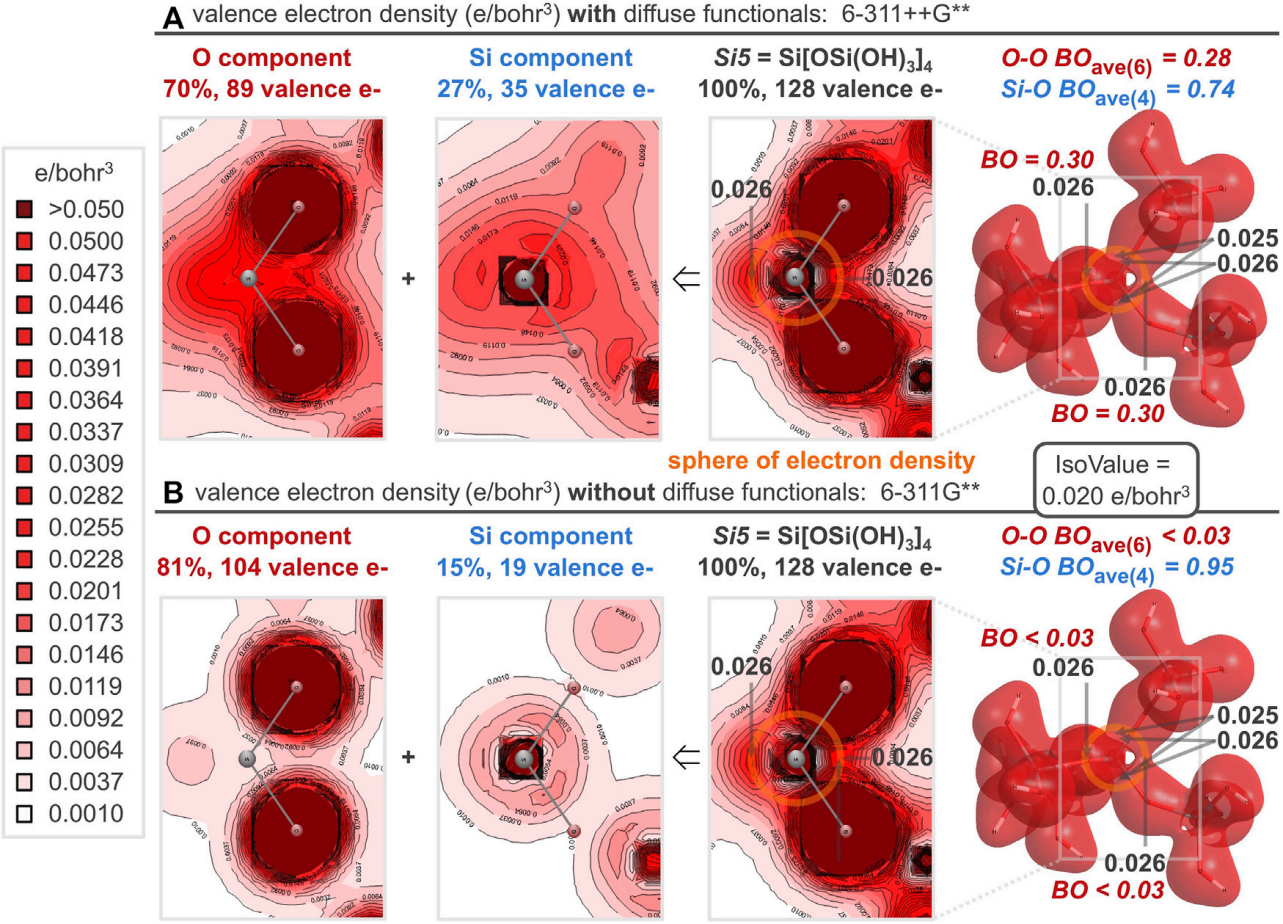
For the *Si5* silica cluster, the computed valence electron density distribution *with* diffuse functionals **(A)** is nearly identical to that *without* diffuse functionals **(B)**, despite differential employment of the constituent oxygen and silicon atomic orbitals. This differential accounting of each electron’s orbital origin results in non-canonical **(A)** or canonical **(B)** O-O and Si-O bond orders. Nonetheless, both computational methods show a spherical node between silicon and oxygen, but a definitive accumulation of electron density between all geminal oxygens, resulting in a *sphere of electron density* (orange circle) responsible for the cooperative bonding in silica.

For silica, it is evident that silicon-oxygen bond dissociation energies are cooperative (Figures 10E, F) and there are no valence bond paths between oxygen and silicon because of an intervening nodal surface (Figures 9A, B). Hence, a ball-and-stick bonding model—germane to water, methane, and many other molecules—is too simplistic to accurately describe the bonding in silica. For Si[OSi(OH)_3_]_4_ (the *Si5* silica cluster), the oxygen and silicon contributions to the valence electron density can be functionally separated into the 2D plots of Figure 11A ^9^. Electron density from the silicon 3*s* and 3*p* atomic orbitals accumulates about 0.8 Å from silicon, coinciding with the midpoint of the geminal O-O axes, where oxygen 2*s* and 2*p* atomic orbitals also accumulate electron density. Thus, silicon and oxygen are perfectly matched to create a *sphere of electron density* 0.8 Å from silicon, about halfway along the silicon-oxygen axes, which are 1.61 Å long; Figure 11A locates this sphere (in orange) just beyond the outer spherical node of the Si 3*s* atomic orbital, which encircles silicon 0.4 Å away (where *VED* = 0 in Figure 9A). Because of this nodal surface, the silicon-oxygen bond of silica does not merit a canonical line between the nuclei; instead, bonding is better defined by a *cooperative sphere model*, where the valence electron density accumulates in a spherical region 0.8 Å from silicon and 0.8–1.3 Å from oxygen.

**FIGURE 12:**
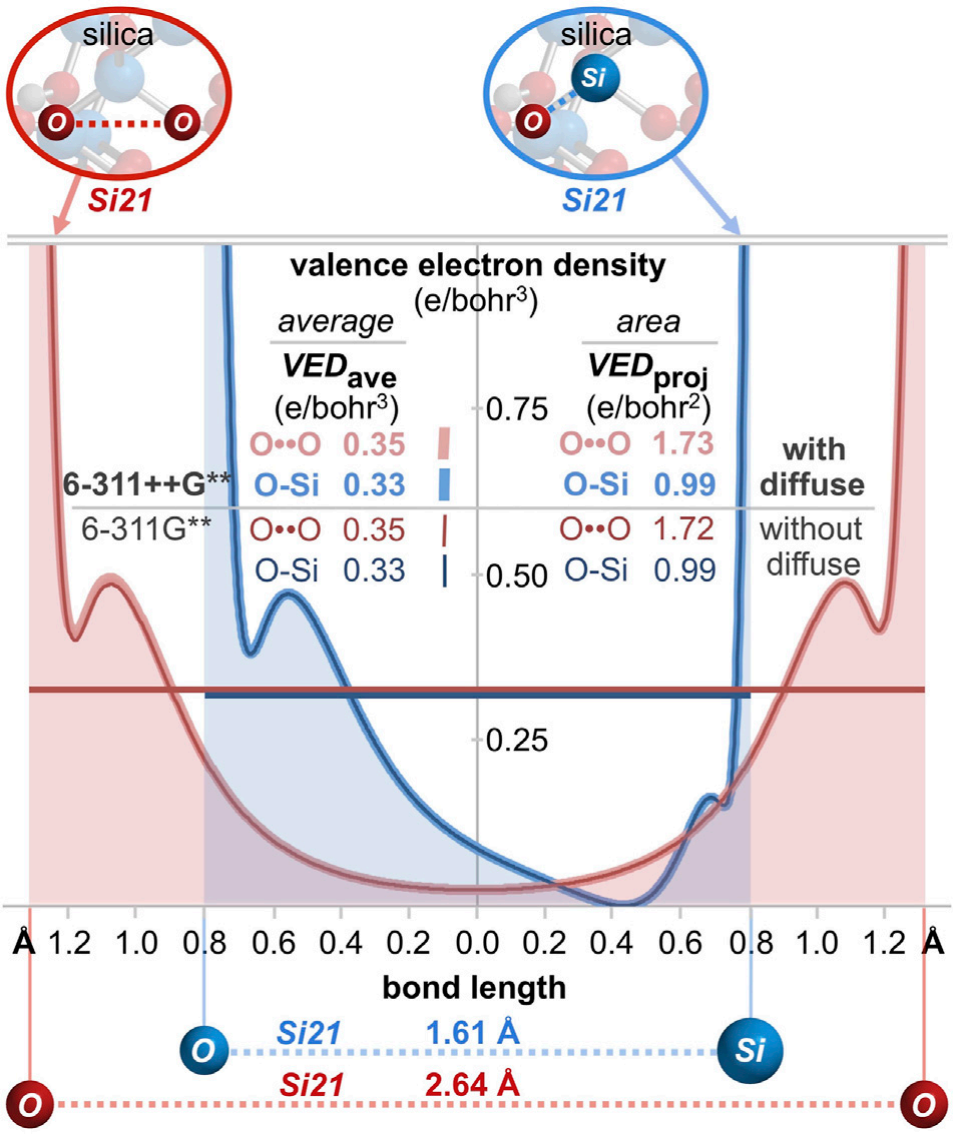
1D valence electron density (*VED*) plots along central O-O and O-Si bond axes of the *Si21* cluster for computations *with* diffuse functionals (thick lines; 6-311++G**) and *without* diffuse functionals (thin lines; 6-311G**). The curves are nearly coincident while the *VED*_ave_ and *VED*_proj_ values are greater for O-O than for O-Si, demonstrating a definitive excess of valence electrons employed for oxygen-oxygen bonding—regardless of diffuse functional employment.

Between each pairwise O-O interaction of the *Si5* central silicate, the valence electron density bond paths reach minima of 0.026, 0.026, 0.026, 0.026, 0.025, and 0.025 e/bohr^3^ (Figure 11A). Notably, the same analysis computed *without* diffuse functionals (Figure 11B) yields the exact same minima between each pairwise O-O interaction and generates indistinguishable valence electron density 3D surfaces and 2D plots. Both diffuse and non-diffuse computational methods aspire to the same valence electron density distribution—with equivalent accumulation of electron density between all geminal oxygen atoms and a nodal surface between canonically bonded silicon and oxygen atoms. However, the two computational methods employ oxygen and silicon atomic orbitals differently to build the valence molecular orbitals. With diffuse functionals, the valence molecular orbitals are 70% oxygen-based and 27% silicon-based. This allocation is more balanced than the computation without diffuse functionals, for which the valence molecular orbitals are 81% oxygen-based and 15% silicon-based. (In both cases, about 3% of the valence molecular orbitals are hydrogen-based.) A purely canonical model intervenes with valence molecular orbitals that are 75% oxygen-based (96 e/128 e) and 16% silicon-based (20 e/128 e). Furthermore, with diffuse functionals, the oxygen component shows significant overlap of the oxygen atomic orbitals (Figure 11A). This results in substantially greater O-O bond orders prescribed by diffuse functionals, averaging 0.28 for the six O-O bonds of the *Si5* central silicate, and commensurately smaller Si-O bond orders, averaging 0.74 for the four Si-O bonds of the *Si5* central silicate. Without diffuse functionals, the oxygen component shows minimal overlap of the oxygen atomic orbitals (Figure 11B). This results in small O-O bond orders near the canonical value of zero (all below the computational threshold of 0.025) and Si-O bond orders near the canonical value of 1.0 (average = 0.95). Since the diffuse and confined computational models allocate electrons differently to atomic orbitals, they differ in the resultant bond orders. Nonetheless, the valence electron density is unmistakably similar, as exhibited by the 3D and 2D valence electron density plots of Figure 11 as well as the 1D plots of Figure 12 for the *Si21* silica cluster. Specifically, the *VED*, *VED*_ave_, and *VED*_proj_ along O-O or O-Si axes are essentially invariant to the employment of diffuse functionals. But, regardless of whether the computational model includes or excludes diffuse functionals, more valence electrons reside between oxygen atoms (*VED*_ave_ = 0.35; *VED*_proj_ = 1.73 or 1.72) than between silicon and oxygen atoms (*VED*_ave_ = 0.33; *VED*_proj_ = 0.99).

### 2.10 Overlap population density of states analysis

**FIGURE 13:**
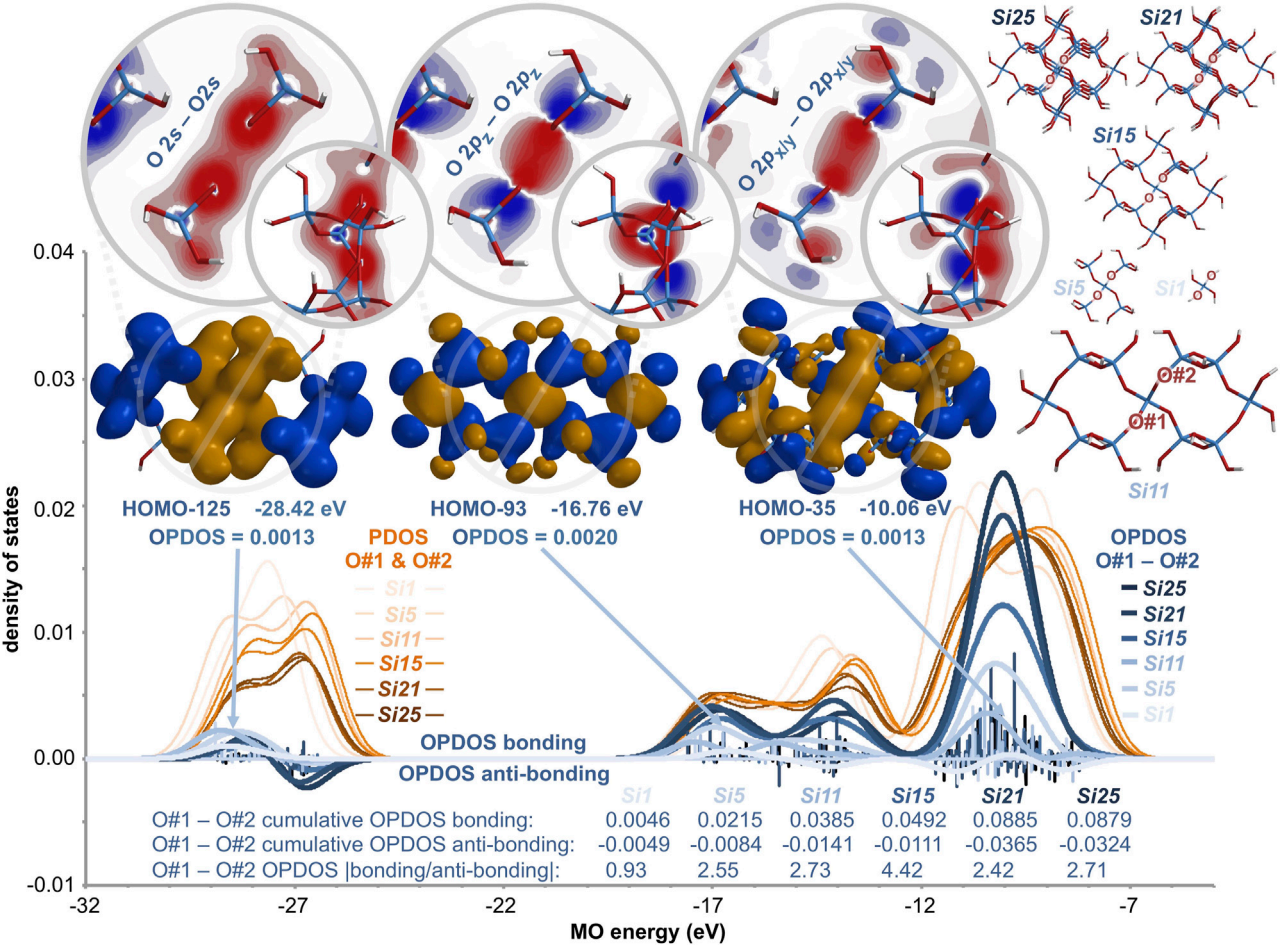
Partial (PDOS, orange) and overlap population (OPDOS, blue) density of states map for a central oxygen-oxygen interaction in silica clusters *Si1*, *Si5*, *Si11*, *Si15*, *Si21*, and *Si25*. Conventional bonding models predict minimal O-O bonding or net O-O anti-bonding, as indicated for *Si1* (|bonding/anti-bonding| < 1). However, OPDOS computations reveal net bonding (|bonding/anti-bonding| > 1) for a central O-O of *Si5* and larger clusters. The computational method is DFT/B3LYP/6-311++G** and representative O#1–O#2 bonding molecular orbitals (HOMO-125, HOMO-93, and HOMO-35) for the *Si11* cluster are depicted with IsoValue = 0.01 [e/bohr^3^]^0.5^—along with orthogonal cross sectional slices having the same wavefunction scale as that in Figure 4.

Additional bonding insight for silica is provided by the valence density of states map (Lu and Chen, 2012) shown in Figure 13. For a central oxygen-oxygen interaction in the silica clusters *Si1*, *Si5*, *Si11*, *Si15*, *Si21*, and *Si25*, the partial density of states (PDOS, orange thin lines) is plotted as a function of molecular orbital energy. Also plotted is the overlap population density of states (OPDOS) (Hughbanks and Hoffmann, 1983), which reveals the bonding states (positive) and anti-bonding states (negative) for a series of molecular orbitals—in analogy to the crystal orbital overlap population (COOP) (Grechnev et al., 2003) method applicable to extended solids. Classical bonding models for silica predict non-bonding or somewhat anti-bonding O-O interactions and thus, OPDOS values near zero as a function of MO energy. The generated O-O OPDOS curve for the *Si1* cluster (thick light blue line) is relatively flat and indicates net anti-bonding, as measured by the sub-unity cumulative OPDOS |bonding/anti-bonding| quotient of 0.93. All extant bonding models predict similarly flat OPDOS curves for any O-O interaction, in any silica cluster, of any size. However, for clusters *Si5* and larger, the curves are not flat and the OPDOS |bonding/anti-bonding| quotient exceeds unity, ranging from 2.42 to 4.42. For these larger clusters, the cumulative O-O OPDOS bonding parameter is proportional to the cluster size and the generated OPDOS curves are positive at nearly all MO energy levels. For the largest cluster, *Si25*, the OPDOS curve closely tracks the PDOS curve and the cumulative OPDOS value (0.088) reaches 43% of the cumulative PDOS value (0.102 + 0.101).

In the molecular orbital region of −29 to −26 eV for all six clusters, the OPDOS curves (Figure 13, thick blue lines) are both positive and negative. This suggests that MOs built from O 2*s* atomic orbitals do not provide substantial net bonding. However, in the molecular orbital region of −18 to −8 eV, the OPDOS curves become increasingly positive with increasing silica cluster size. This suggests that O 2*p* atomic orbital overlap is largely responsible for the oxygen-oxygen bonding in silica. Exemplary molecular orbitals are shown in Figure 13 for the *Si11* cluster. Oxygen atom #1 and oxygen atom #2 have σ bonding interactions *via* their 2*s* atomic orbitals in the HOMO-125, their 2*p*_z_ atomic orbitals in the HOMO-93, and their 2*p*_x/y_ atomic orbitals in the HOMO-35. Orthogonal cross sections of these MOs locate the accumulation of electron density along the O-O axis. Importantly, the OPDOS analysis shows that increasing cluster size augments the bonding for the O 2*p*_x/y_–O 2*p*_x/y_ interactions (−12 to −8 eV) more than the O 2*p*_z_–O 2*p*_z_ interactions (−18 to −12 eV).

What is the basis for this bonding augmentation? A detailed OPDOS analysis of all six central O-O valence interactions in the *Si1*–*Si25* clusters (Table 2) reveals two effects. The first effect concerns the *number* of bonding vs anti-bonding interactions. Adding oxygen atoms to the *periphery* of a silica cluster decreases the nodal density between oxygen atoms in the *center* of the silica cluster. The nodal density is inversely related to the OPDOS % *bonding excess* reported in Table 2. While the % *bonding excess* is somewhat invariant to cluster size for the O 2*s* MOs (−29 to −26 eV) and the O 2*p*_z_ MOs (−18 to −12 eV), this parameter increases correspondingly with cluster size for the O 2*p*_x/y_ MOs (−12 to −8 eV)—from −38% to 26%—fully consistent with the dominant growth of the OPDOS curve in the same region (Figure 13). The second effect concerns the *magnitude* of the bonding vs anti-bonding interactions. Adding oxygen atoms to the *periphery* of a silica cluster increases the cumulative OPDOS bonding parameter more than the cumulative anti-bonding OPDOS parameter for the oxygen atoms in the *center* of the silica cluster. Thus, the |bonding/anti-bonding| quotient steadily increases from 0.76 to 2.42 with increasing cluster size (Table 2). (Note that the |bonding/anti-bonding| quotient in Figure 13 represents only one of the six O-O interactions in the central silicate unit of the clusters—all of which are averaged to yield the values in Table 2.) It is clear that the oxygen-oxygen bonding metrics of a silicate unit are sensitive to the number of peripheral silicate units. Assuredly, this effect tapers with cluster size, but the trends all indicate considerable covalent O-O bonding in bulk silica.

**TABLE 2:** Bonding vs anti-bonding interactions for the six central silicate oxygen-oxygen pairs according to overlap population density of states (OPDOS) analysis for silica clusters *Si1*–*Si25*.

cluster	*Si1*	*Si5*	*Si11*	*Si15*	*Si21*	*Si25*
# oxygen atoms	4	16	32	42	56	66
# valence MOs	16	64	128	168	224	264
# O-O interactions (+& −)	96	384	768	1,008	1,344	1,584
# O-O bonding (+)	43	219	462	623	826	991
2*s*	16	63	118	153	188	248
2*p*_z_	12	60	129	163	218	245
2*p*_x_ and 2*p*_y_	15	96	215	307	420	498
# O-O anti-bonding (−)	53	165	306	385	518	593
2*s*	8	33	74	99	148	148
2*p*_z_	12	36	63	89	118	151
2*p*_x_ and 2*p*_y_	33	96	169	197	252	294
OPDOS % *bonding excess*	−10	14	20	24	23	25
2*s*	33	31	23	21	12	25
2*p*_z_	0	25	34	29	30	24
2*p*_x_ and 2*p*_y_	−38	0	12	22	25	26
cumulative O-O OPDOS						
cumulative bonding	0.003	0.015	0.024	0.037	0.067	0.079
cumulative anti-bonding	−0.004	−0.008	−0.013	−0.017	−0.030	−0.032
|bonding/anti-bonding|	0.76	1.85	1.78	2.17	2.26	2.42
average Mulliken O-O BO	0.05	0.28	0.36	0.38	0.46	0.46

## 3 Conclusions and outlook

A molecular orbital analysis of α-quartz silica model complexes reveals that oxygen valence electrons abandon their canonically prescribed locations to form long covalent oxygen-oxygen bonds. Oblique arrangements of the oxygen atoms minimize molecular orbital nodes and maximize bonding interactions that are 2.61–2.64 Å apart, yet have Mulliken bond orders reaching 0.63 and averaging 0.47. This nodal minimization is inherently challenging for linear or planar molecules, but may prove widespread among atoms arranged in three dimensions—accomplished especially *via p* atomic orbitals. Thereby, the geminal oxygen atoms of silica bond cooperatively with a computed O-O bond dissociation energy of 4.4 kcal/mol when they are suitably arranged and interspersed with the electropositive element silicon. Generally, as silica model complexes increase in size, canonical bonding paradigms decrease in accuracy and resonance hybrids increase in relevance. This comports with the original Hund-Mulliken molecular orbital theory, which states that the “best MOs … spread at least to some slight extent over all atoms.” (Mulliken, 1970) For Pauling, the concept of resonance had “its most important chemical applications” to “molecules to which no satisfactory single structure in terms of single bonds, double bonds, and triple bonds can be assigned.” (Pauling, 1946) Indeed, the structure, bonding, and energetic stability of silica cannot be fully understood without resonance hybrids involving long oxygen-oxygen bonds. This covalent bonding likely exists between other distant atoms and promises to impact the understanding of many materials and processes.

The hybridization theory of Pauling (Pauling, 1930; Pauling, 1931) and Slater (Slater, 1931) compelled chemists to reconsider the location of nearly *all* valence electrons. The curious bonding in ferrocene (Fischer and Pfab, 1952; Wilkinson et al., 1952), with multiple metal-carbon bonds, also provided a paradigm shift in our understanding of bonding and the location of electrons; however, this bonding arrangement pertains only to a class of esoteric, man-made organometallic sandwich compounds. The bonding in diborane (Lipscomb, 1973), with three-center two-electron bonds, provided another paradigm shift in our understanding of bonding arrangements in molecules; but this bonding motif is also uncommon, primarily ascribed to a limited number of main group and transition metal complexes (Longuet-Higgins and Roberts, 1955). The long covalent bond theory (LCBT) posited herein signals a new paradigm shift in the location of the chemical bond. As evidenced by resonance formulations, bond orders (Mulliken, Wiberg, Mayer), multicenter bond order indices (3-center and 4-center), atomic valencies (Mulliken, Mayer), molecular orbitals (bonding > anti-bonding interactions), bonding analogies, electron density calculations, valence bond path calculations, overlap population density of states (OPDOS) analysis, and bonding energetics, it is clear that Nature builds materials with long covalent bonds—not just the short canonical bonds introduced by Couper (1858) and Kekulé (1866) whose primitive bonding model has somehow reigned over 160 years. These long bonds underpin an explanation for the contorted, chiral structure and energetic stability of α-quartz silica, wherein distant oxygen-oxygen bonding supplements conventional bonding. This bonding paradigm is abundant and pervasive since more than one-third of the 10^49^ valence electrons in the Earth’s crust are allocated to long covalent bonds^10^. Moreover, 10^48^ crustal valence electrons inhabit silica’s oxygen-based Möbius aromatic orbitals—manifestly the most prevalent sort of aromaticity. Astonishingly, LCBT implicates the oxygen-oxygen bond as the most abundant bond on Earth. While this study focuses on silica, future work will reveal the prevalence, energetics, and importance of long covalent bonds in a rather wide variety of materials—especially those with periodic structures, including ice, biopolymers, bone, and superconducting ceramics.

## 4 Experimental section

All calculations herein are performed with Spartan/Q-Chem according to DFT/B3LYP/6-311++G**, except where noted in Table 1, Figure 11B, and Figure 12 (Shao et al., 2015). Spartan Output files were processed with *AOMix: Program for Molecular Orbital Analysis* (version 6.94, written by S. I. Gorelsky) (Gorelsky and Lever, 2001)^7^ or with *Chemissian* (version 4.67, written by L. Skripnikov)^5^ to generate additional computational metrics, as noted. OPDOS plots were computed with Multiwfn (version 3.8, written by T. Lu and F. Chen) (Lu and Chen, 2012) *via* Gaussian 09 formatted checkpoint files (Frisch et al., 2009).

## Data Availability

The original contributions presented in the study are included in the article/Supplementary Material; further inquiries can be directed to the corresponding author.

## Footnotes

1 Diffuse functionals (++) are employed since they better account for long range interactions and periodic bonding while universally providing lower electronic energies versus confined functionals by about 0.21 kcal/mol of valence electrons. For example, electron confinement costs 136.1 kcal/mol for the Si_29_O_76_H_36_ cluster (*Si29*) and about 3.4 kcal/mol of SiO_2_.

2 For Si(OSi)_4_, oxygen contributes 55% (= [4x6]/[4x6 + 5x4]) of the 44 valence electrons and silicon contributes 45% (= [5x4]/[4x6 + 5x4]).

3 For mathematical simplicity, apparent non-bonding interactions are counted as anti-bonding interactions.

4 Oxygen contributes (6 + 6)/(6 + 6+4) = 75% of silica’s valence electrons and 8 of the 24 oxygen-based valence MOs are Möbius aromatic for the Si_6_O_6_ model. Accordingly, 25% (= 75% x [8/24]) of silica’s valence electrons are Möbius aromatic. Analogously, only 6% (= 75% x [2/24]) of silica’s valence electrons are Hückel aromatic.

5 Valence bond path computations were performed with the *Chemissian* software program via Spartan Output files for HOOH, O_3_, and the *Si5* and *Si21* silica clusters. See the Supplementary Material. *Chemissian*; version 4.67 www.chemissian.com.

6 As expected, this same analysis for the short, canonical O-O bond of ozone (1.26 Å) indicates metrics of greater bonding: Bond order = 1.44; minimum O-O ED = 0.47 e/bohr^3^; minimum O-O IP = 28.5 eV; *VED*_ave_ = 0.68 e/bohr^3^; *VED*_min_ = 0.23 e/bohr^3^; and *VED*_proj_ = 1.60 e/bohr^2^.

7 Wiberg bond order, Mayer bond order, multicenter Mayer-type bond order indices, and Mayer atomic valencies were calculated with the *AOMix* software package via the Spartan Output files for various silica cluster complexes. *AOMix: Program for molecular orbital analysis*. See the Supplementary Material. Gorelsky, S. I. version 6.94. http://www.sg-chem.net/.

8 Herein, the Mulliken bond order for the central transannular sigma bond (Dewar type) computes to 0.07 for coronene or 0.11 for hexabenzocoronene.

9 The valence electron density of *Si5* was parsed to the oxygen valence electron density component by setting all silicon and hydrogen coefficients to zero in the Spartan Output (.txt) file prior to the *Analyze MO compositions* function in *Chemissian*. The silicon valence electron density component was similarly computed after setting all oxygen and hydrogen coefficients to zero.

10 Silica constitutes 59% of the Earth’s crust (Clarke and Washington, 1924) and provides about 66% of crustal valence electrons. Since over half (52%) of these electrons are spent on oxygen-oxygen bonding, over one-third of crustal valence electrons participate in the long O-O covalent bonds of silica.

## References

[B2] Bader R. F. W. (1990). Atoms in molecules: A quantum theory. Oxford, UK: Oxford University Press. https://global.oup.com/academic/product/atoms-in-molecules-9780198558651.

[B3] Bochicchio R. C. Ponec R. Lain L. Torre A. (1998). On the physical meaning of bond indices from the population analysis of higher order densities. J. Phys. Chem. A 102, 7176–7180. 10.1021/jp981816d

[B4] Bridgeman A. J. Cavigliasso G. Ireland L. R. Rothery J. (2001). The Mayer bond order as a tool in inorganic chemistry. J. Chem. Soc. Dalton Trans. 2001, 2095–2108. The transannular S-S Mayer bond order (2.67 Å) in S_4_N_4_ reportedly ranges from 0.22 to 0.56, while its Mulliken bond order is herein computed to be 0.40. 10.1039/b102094n

[B5] Chen S. Xu Z. Li J. (2016). The observation of oxygen-oxygen interactions in ice. New J. Phys. 18, 023052. A long “oxygen-oxygen interaction” in various pressurized ices (2.5–2.7 Å) has been proposed to explain vibrational spectra, but “a detailed mechanism is still unclear.” 10.1088/1367-2630/18/2/023052

[B6] Clarke F. W. Washington H. S. (1924). The composition of the Earth's crust. Washington, DC: USGS Professional Paper 127, Department of the Interior, United States Geological Survey, U.S. Government Printing Office. 10.3133/pp127

[B7] Couper A. S. (1858). Sur une nouvelle théorie chimique. Ann. Chim. Phys. 53, 469–489. https://gallica.bnf.fr/ark:/12148/bpt6k34794n/f468.item.

[B8] Crabtree K. N. Talipov M. R. Martinez O. Jr. O’Connor G. D. Khursan S. L. McCarthy M. C. (2013). Detection and structure of HOON: Microwave spectroscopy reveals an O–O bond exceeding 1.9 Å. Science 342, 1354–1357. 10.1126/science.1244180 24337293

[B9] de Giambiagi M. S. Giambiagi M. de Souza Fortes M. (1997). Multicenter bonds, bond valence and bond charge apportionment. J. Mol. Struct. (Theochem) 391, 141–150. 10.1016/S0166-1280(96)04815-4

[B10] Dolbier W. R. Jr. Medinger K. S. Greenberg A. Liebman J. F. (1982). The thermodynamic effect of fluorine as a substituent: Vinylic CF_2_ and CFH and allylic CF_2_C. Tetrahedron 38, 2415–2420. 10.1016/0040-4020(82)87020-8

[B11] Feynman R. P. (1939). Forces in molecules. Phys. Rev. 56, 340–343. 10.1103/PhysRev.56.340

[B12] Fischer E. O. Pfab W. (1952). Cyclopentadien-Metallkomplexe, ein neuer Typ metallorganischer Verbindungen. Z. Naturforsch. B 7, 377–379. 10.1515/znb-1952-0701

[B13] Frisch M. J. Trucks G. W. Schlegel H. B. Scuseria G. E. Robb M. A. Cheeseman J. R. (2009). Gaussian 09, revision D.01. Wallingford CT: Gaussian, Inc. https://gaussian.com/g09citation/.

[B14] Garvie L. A. J. Rez P. Alvarez J. R. Buseck P. R. Craven A. J. Brydson R. (2000). Bonding in alpha-quartz (SiO_2_): A view of the unoccupied states. Am. Mineral. 85, 732–738. 10.2138/am-2000-5-611

[B15] Gibbs G. V. Wallace A. F. Cox D. F. Downs R. T. Ross N. L. Rosso K. M. (2009). Bonded interactions in silica polymorphs, silicates, and siloxane molecules. Am. Mineral. 94, 1085–1102. 10.2138/am.2009.3215

[B16] Goodman L. Sauers R. R. (2007). Diffuse functions in natural bond orbital analysis. J. Comput. Chem. 28, 269–275. 10.1002/jcc.20519 17149729

[B17] Gorelsky S. I. Lever A. B. P. (2001). Electronic structure and spectra of ruthenium diimine complexes by density functional theory and INDO/S. Comparison of the two methods. J. Organomet. Chem. 635, 187–196. 10.1016/S0022-328X(01)01079-8

[B18] Grechnev A. Ahuja R. Eriksson O. (2003). Balanced crystal orbital overlap population—A tool for analysing chemical bonds in solids. J. Phys. Condens. Matter 15, 7751–7761. 10.1088/0953-8984/15/45/014

[B19] Hay P. J. Dunning T. H. Goddard W. A. III (1975). Configuration interaction studies of O_3_ and O^+^ _3_. Ground and excited states. J. Chem. Phys. 62, 3912–3924. Ozone reportedly has “weak bonding between the… terminal oxygen atoms.” 10.1063/1.430306

[B20] Hine J. (1963). Polar effects on rates and equilibria. VIII. Double bond-no bond resonance. J. Am. Chem. Soc. 85, 3239–3244. 10.1021/ja00903a041

[B21] Hoffmann R. (1963). An extended Hückel theory. I. Hydrocarbons. J. Chem. Phys. 39, 1397–1412. 10.1063/1.1734456

[B22] Hoffmann R. Heilbronner E. Gleiter R. (1970). Interaction of nonconjugated double bonds. J. Am. Chem. Soc. 92, 706–707. An early version of *alternoconjugation* described norbornadiene with alternating pi bonds (C2=C3 and C5=C6) and *sp* ^3^ hybridized carbons (C1 and C4). Presently, a computational analysis of norbornadiene indicates long carbon-carbon bonds (C2–C6 and C3–C5) with a length of 2.49 Å and a Mulliken bond order of 0.19 via interaction of distant C 2*p* orbitals. 10.1021/ja00706a051

[B23] Hoffmann R. (1971). Interaction of orbitals through space and through bonds. Acc. Chem. Res. 4, 1–9. 10.1021/ar50037a001

[B24] Hughbanks T. Hoffmann R. (1983). Chains of trans-edge-sharing molybdenum octahedra: Metal-metal bonding in extended systems. J. Am. Chem. Soc. 105, 3528–3537. 10.1021/ja00349a027

[B25] Jabłoński M. (2012). Energetic and geometrical evidence of nonbonding character of some intramolecular Halogen···Oxygen and other Y···Y interactions. J. Phys. Chem. A 116, 3753–3764. Longer oxygen-oxygen interactions in organic molecules (*e.g.*, 2.71–2.90 Å in HO-(CR)_3_=O) were studied computationally, but deemed “most likely” repulsive and presently are calculated to have small bond orders ranging from 0.03 to 0.06. 10.1021/jp300993b 22432471

[B26] Janes N. Oldfield E. (1986). Oxygen-17 NMR study of bonding in silicates: The d-orbital controversy. J. Am. Chem. Soc. 108, 5743–5753. 10.1021/ja00279a014 22175322

[B27] Kekulé A. (1866). Lehrbuch der Organischen chemie. Second Edition. Erlangen: Verlag von Ferdinand Enke. See page 496 for an early structure of benzene https://books.google.com.ag/books?id=9KMEAAAAYAAJ .

[B28] Kirfel A. Eichhorn K. (1990). Accurate structure analysis with synchrotron radiation. The electron density in Al_2_O_3_ and Cu_2_O. Acta Cryst. A46, 271–284. 10.1107/S0108767389012596

[B29] Lemal D. M. (2004). Perspective on fluorocarbon chemistry. J. Org. Chem. 69, 1–11. 10.1021/jo0302556 14703372

[B30] Levien L. Prewitt C. T. Weidner D. J. (1980). Structure and elastic properties of quartz at pressure. Am. Mineral. 65, 920–930. For all model complexes, silicon and oxygen atoms are restricted to the atomic coordinates of α-quartz silica. https://pubs.geoscienceworld.org/msa/ammin/article-abstract/65/9-10/920/41195 .

[B31] Lewars E. G. (2008). “Pyramidal carbon (chapter 2),” in Modeling marvels: Computational anticipation of novel molecules (Netherlands: Springer Science+Business Media B.V.), 13–29. 10.1007/978-1-4020-6973-4

[B32] Lipscomb W. N. (1973). Three-center bonds in electron-deficient compounds. Localized molecular orbital approach. Acc. Chem. Res. 6, 257–262. 10.1021/ar50068a001

[B33] Longuet-Higgins H. C. Roberts M. D. V. (1955). The electronic structure of an icosahedron of boron atoms. Proc. R. Soc. Lond. Ser. A 230, 110–119. 10.1098/rspa.1955.0115

[B34] Löwdin P.-O. (1970). On the nonorthogonality problem. Adv. Quantum Chem. 5, 185–199. 10.1016/S0065-3276(08)60339-1

[B35] Lu T. Chen F. (2012). Multiwfn: A multifunctional wavefunction analyzer. J. Comput. Chem. 33, 580–592. 10.1002/jcc.22885 22162017

[B36] Mayer I. (2007). Bond order and valence indices: A personal account. J. Comput. Chem. 28, 204–221. 10.1002/jcc.20494 17066501

[B37] Mayer I. (1983). Charge, bond order and valence in the *ab initio* SCF theory. Chem. Phys. Lett. 97, 270–274. 10.1016/0009-2614(83)80005-0

[B38] Mayer I. (2003). Population analysis, bond orders, and valences (chapter 7) *in* Simple theorems, proofs, and derivations in quantum chemistry. New York: Springer Science+Business Media, 227–249. 10.1007/978-1-4757-6519-9

[B39] McNaught A. D. Wilkinson A. (1997). IUPAC Gold Book definition for homoconjugation: The overlap of two π-systems separated by a non-conjugating group. IUPAC Compendium of Chemical Terminology. 2nd ed. Oxford: Blackwell Scientific Publications. (the “Gold Book”). Compiled by McNaught, A. D.; Wilkinson, A. Online version 2019, created by Chalk, S. J. https://goldbook.iupac.org/terms/view/H02842.

[B40] Mulliken R. S. (1955a). Electronic population analysis on LCAO–MO molecular wave functions. II. Overlap populations, bond orders, and covalent bond energies. J. Chem. Phys. 23, 1841–1846. Mulliken bond orders are more precisely termed *overlap populations*. Mulliken stated that “the advantages of overlap populations over bond orders as measures of resonance energy are obvious”. 10.1063/1.1740589

[B41] Mulliken R. S. (1955b). Electronic population analysis on LCAO–MO molecular wave functions. I. J. Chem. Phys. 23, 1833–1840. 10.1063/1.1740588

[B42] Mulliken R. S. (1955c). Electronic population analysis on LCAO-MO molecular wave functions. IV. Bonding and antibonding in LCAO and valence-bond theories. J. Chem. Phys. 23, 2343–2346. While negative bond orders have no physical meaning, Mulliken interpreted negative overlap populations to signify anti-bonding between atoms. Negative Mulliken bond orders generally translate to Wiberg and Mayer bond orders of zero, as they do here for *D* _6h_ Si_6_O_6_ . 10.1063/1.1741877

[B43] Mulliken R. S. (1970). The path to molecular orbital theory. Pure Appl. Chem. 24, 203–216. 10.1351/pac197024010203

[B44] Newton M. D. Gibbs G. V. (1980). *Ab initio* calculated geometries and charge distributions for H_4_SiO_4_ and H_6_Si_2_O_7_ compared with experimental values for silicates and siloxanes. Phys. Chem. Miner. 6, 221–246. 10.1007/BF00309858

[B45] Noritake F. Kawamura K. (2015). The nature of Si-O-Si bonding via molecular orbital calculations. J. Comput. Chem. Jpn. 14, 124–130. 10.2477/jccj.2015-0009

[B46] Pauling L. (1930). “Eigenfunctions for chemical bonds,” in Linus Pauling, the Nature of the Chemical Bond, A Documentary History. Special Collections & Archives Research Center, Oregon State University Libraries. http://scarc.library.oregonstate.edu/coll/pauling/bond/notes/sci3.002.2.html.

[B47] Pauling L. (1952). Interatomic distances and bond character in the oxygen acids and related substances. J. Phys. Chem. 56, 361–365. According to Pauling’s original equation for *partial ionic character* = 1 – e^–¼(Δx)(Δx)^ . 10.1021/j150495a016

[B48] Pauling L. (1946). “Resonance,” in Linus Pauling, the Nature of the Chemical Bond, A Documentary History, 5–6. Special Collections & Archives Research Center, Oregon State University Libraries. http://scarc.library.oregonstate.edu/coll/pauling/bond/notes/1946a.3.html.

[B49] Pauling L. (1980). The nature of silicon-oxygen bonds. Am. Mineral. 65, 321–323. https://pubs.geoscienceworld.org/msa/ammin/article-abstract/65/3-4/321/41136.

[B50] Pauling L. (1960). The nature of the chemical bond. Third Edition. Ithaca, New York: Cornell University Press, 12. https://books.google.com/books?id=L-1K9HmKmUUC&pg=PA12.

[B51] Pauling L. (1931). The nature of the chemical bond. Application of results obtained from the quantum mechanics and from a theory of paramagnetic susceptibility to the structure of molecules. J. Am. Chem. Soc. 53, 1367–1400. 10.1021/ja01355a027

[B52] Pauling L. Wheland G. W. (1933). The nature of the chemical bond. V. The quantum-mechanical calculation of the resonance energy of benzene and naphthalene and the hydrocarbon free radicals. J. Chem. Phys. 1, 362–374. 10.1063/1.1749304

[B53] Ponec R. Mayer I. (1997). Investigation of some properties of multicenter bond indices. J. Phys. Chem. A 101, 1738–1741. 10.1021/jp962510e

[B54] Ponomarev D. A. Takhistov V. V. (1997). What are isodesmic reactions? J. Chem. Educ. 74, 201–203. 10.1021/ed074p201

[B55] Rappaport S. M. Rzepa H. S. (2008). Intrinsically chiral aromaticity. Rules incorporating linking number, twist, and writhe for higher-twist Möbius annulenes. J. Am. Chem. Soc. 130, 7613–7619. Because of opposite twisting directions, one MO has *L* _k_ = +2 and one MO has *L* _k_ = –2. 10.1021/ja710438j 18505260

[B56] Reed A. E. von Ragué Schleyer P. (1987). The anomeric effect with central atoms other than carbon. 1. Strong interactions between nonbonded substituents in polyfluorinated first- and second-row hydrides. J. Am. Chem. Soc. 109, 7362–7373. 10.1021/ja00258a020

[B57] Reed A. E. Weinstock R. B. Weinhold F. (1985). Natural population analysis. J. Chem. Phys. 83, 735–746. 10.1063/1.449486

[B58] Rettig S. J. Trotter J. (1987). Refinement of the structure of orthorhombic sulfur, α-S_8_ . Acta Cryst. C43, 2260–2262. 10.1107/S0108270187088152

[B59] Roberts J. D. Webb R. L. McElhill E. A. (1950). The electrical effect of the trifluoromethyl group. J. Am. Chem. Soc. 72, 408–411. 10.1021/ja01157a111

[B60] Rzepa H. S. (2005). Möbius aromaticity and delocalization. Chem. Rev. 105, 3697–3715. 10.1021/cr030092l 16218564

[B61] Sannigrahi A. B. Kar T. (1999). Some remarks on multi-center bond index. Chem. Phys. Lett. 299, 518–526. 10.1016/S0009-2614(98)01267-6

[B62] Segall M. D. Shah R. Pickard C. J. Payne M. C. (1996). Population analysis of plane-wave electronic structure calculations of bulk materials. Phys. Rev. B 54, 16317–16320. 10.1103/PhysRevB.54.16317 9985733

[B63] Shao Y. Gan Z. Epifanovsky E. Gilbert A. T. B. Wormit M. Kussmann J. (2015). Advances in molecular quantum chemistry contained in the Q-Chem 4 program package. Mol. Phys. 113, 184–215. 10.1080/00268976.2014.952696

[B64] Silvi B. Savin A. Wagner F. R. (1997). “The nature of silicon-oxygen bonds in silica polymorphs,” in Modelling of minerals and silicated materials. Editors Silvi B. D’Arco P. (Dordrecht: Springer), Vol. 15. Topics in Molecular Organization and Engineering. 10.1007/0-306-46933-2_7

[B66] Slater J. C. (1931). Directed valence in polyatomic molecules. Phys. Rev. 37, 481–489. 10.1103/PhysRev.37.481

[B67] Sorella S. Zen A. (2014). “The new resonating valence bond method for ab-initio electronic simulations,” in Many-electron approaches in physics, chemistry and mathematics. Editors Bach V. Delle Site L. (Switzerland: Springer International Publishing), 377–392. 10.1007/978-3-319-06379-9_21

[B68] Torgunrud J. L. Faria A. J. Miller S. A. (2020). Thermodynamics of silica depolymerization with alcohols. Polyhedron 187, 114562. A resonance energy of 16.0 kcal/mol of SiO_2_ has been estimated via computational hydrolysis of Si[OSi(OH)_3_]_4_ . 10.1016/j.poly.2020.114562

[B69] Weinhold F. Landis C. R. (2005). Valency and bonding: A natural bond orbital donor-acceptor perspective. Cambridge: Cambridge University Press. 10.1017/CBO9780511614569

[B70] Wheeler S. E. (2012). Homodesmotic reactions for thermochemistry. WIREs Comput. Mol. Sci. 2, 204–220. 10.1002/wcms.72

[B71] Wiberg K. B. (1968). Application of the Pople-Santry-Segal CNDO method to the cyclopropylcarbinyl and cyclobutyl cation and to bicyclobutane. Tetrahedron 24, 1083–1096. 10.1016/0040-4020(68)88057-3

[B72] Wiberg K. B. Rablen P. R. (1993). Origin of the stability of carbon tetrafluoride: Negative hyperconjugation reexamined. J. Am. Chem. Soc. 115, 614–625. 10.1021/ja00055a034

[B73] Wilkinson G. Rosenblum M. Whiting M. C. Woodward R. B. (1952). The structure of iron biscyclopentadienyl. J. Am. Chem. Soc. 74, 2125–2126. 10.1021/ja01128a527

