# Supplementary material for: The location of the chemical bond. Application of long covalent bond theory to the structure of silica
Source: Front Chem. 2023 Feb 16;11:1123322. doi: 10.3389/fchem.2023.1123322 (PMC9978528; doi:10.3389/fchem.2023.1123322)

Supplementary Material

The Location of the Chemical Bond. Application of Long Covalent Bond Theory to the Structure of Silica

Stephen A. Miller*
ORCID ID 0000-0001-7005-7537
https://orcid.org/0000-0001-7005-7537

The George and Josephine Butler Polymer Research Laboratory
Department of Chemistry, University of Florida, Gainesville, Florida 32611-7200, USA
Email: miller@chem.ufl.edu

Table of Contents

Computational Methods S2
CIF Files and Model Complex Construction S4
Computational Results S6
Valence Bond Path Computations S122
Bond Dissociation Energy Calculations S135
Additional Resonance Hybrids S149
Overlap Population Density of States Analysis S152
Valence Electron Calculations for the Earth's Crust S177
References S178

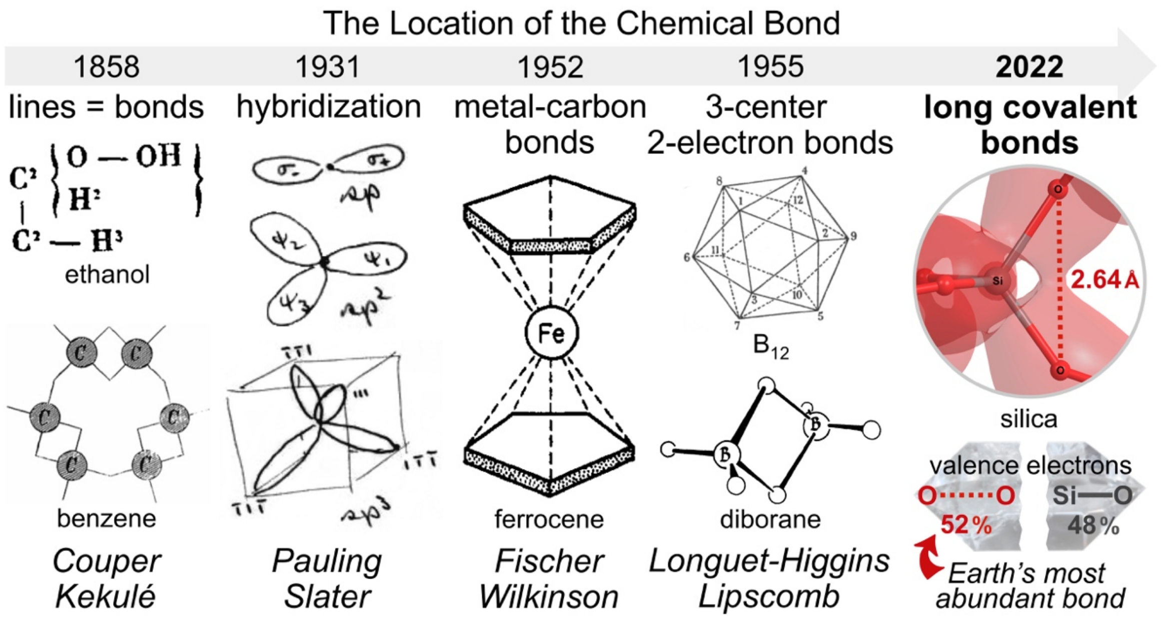

Molecular orbital analysis of model silica clusters reveals that over one-third of Earth's valence electrons belong to long covalent oxygen-oxygen bonds, which conspire to stabilize silica via Möbius aromaticity.

(The valence electron density surface (IsoValue = 0.022 e/bohr^3) depicts an oxygen-oxygen valence bond path within the silica model complex, $Si_5O_{16}H_{12}$. See Figure S14 for details regarding its generation.)

Computational Methods

Computational results were obtained with Q-Chem/Spartan'18 (version 1.4.4)[1] running on an Apple iMac with Mac OS Mojave version 10.14.6 and a 3.4 GHz Intel Quad Core i5 processor. Single point energy calculations (silica clusters) and geometry optimizations (other than silica clusters) were performed at the DFT/B3LYP/6-311++G** level of theory, employed for consistency and because its versatility allowed convergence of both small and large structures described below. Enthalpic energies were taken as the electronic energy, E, provided in hartrees. Bond orders, atomic charges, and molecular orbital energies are provided in the Spartan property archive (spproparc) file, with the caveat that bond orders between –0.025 and 0.025 are not calculated.

Diffuse functionals (++) are employed since they better account for long range interactions and periodic bonding while universally providing lower electronic energies versus confined functionals. Table S1 tabulates the electronic energies calculated for silica clusters via DFT/B3LYP/6-311++G** vs. DFT/B3LYP/6-311G**, which lacks diffuse functionals. Figure S1 plots this energy difference versus the number of valence electrons in each cluster. The slope of the regression line describes the energetic advantage of using diffuse functionals to be about 0.214 kcal/mol per valence electron. This is an energy difference of about 3.4 kcal/mol of SiO_2 (16 valence electrons).

Table S1. Electronic energies (E) calculated with and without diffuse functionals (++).

cluster	formula	valence e	diffuse	diffuse	no diffuse	no diffuse	difference	diff per val e
			E, hartrees	E, kcal/mol	E, hartrees	E, kcal/mol	E, kcal/mol	E, kcal/mol
Si1	SiO4H4	32	-593.1208008	-372188.6406	-593.1037523	-372177.9425	10.69808719	0.334315225
Si2	Si2O7H6	56	-1109.794721	-696406.1756	-1109.768525	-696389.7372	16.43841402	0.293543107
Si3	Si3O10H8	80	-1626.469013	-1020623.944	-1626.433422	-1020601.61	22.33411208	0.279176401
Si4	Si4O13H10	104	-2143.140363	-1344839.866	-2143.094948	-1344811.368	28.49807023	0.274019906
Si5	Si5O16H12	128	-2659.811570	-1669055.698	-2659.75631	-1669021.022	34.67639834	0.270909362
Si8	Si8O24H16	192	-4133.379723	-2593732.976	-4133.301943	-2593684.169	48.80746177	0.25420553
Si11	Si11O32H20	256	-5606.947295	-3518409.89	-5606.846854	-3518346.862	63.02800797	0.246203156
Si15	Si15O42H24	336	-7520.743929	-4719334.502	-7520.616424	-4719254.492	80.01066055	0.238126966
Si18	Si18O50H28	400	-8994.315128	-5644013.692	-8994.164209	-5643918.988	94.70315627	0.236757891
Si21	Si21O56H28	448	-10315.0041	-6472757.908	-10314.84263	-6472656.585	101.3232507	0.22616797
Si25	Si25O66H32	528	-12228.80179	-7673683.185	-12228.61218	-7673564.202	118.9829228	0.225346445
Si29	Si29O76H36	608	-14142.58951	-8874602.2	-14142.37259	-8874466.082	136.118562	0.223879214
Si35	Si35O90H40	720	-16936.85433	-10628028.52	-16936.60204	-10627870.21	158.3129906	0.219879154

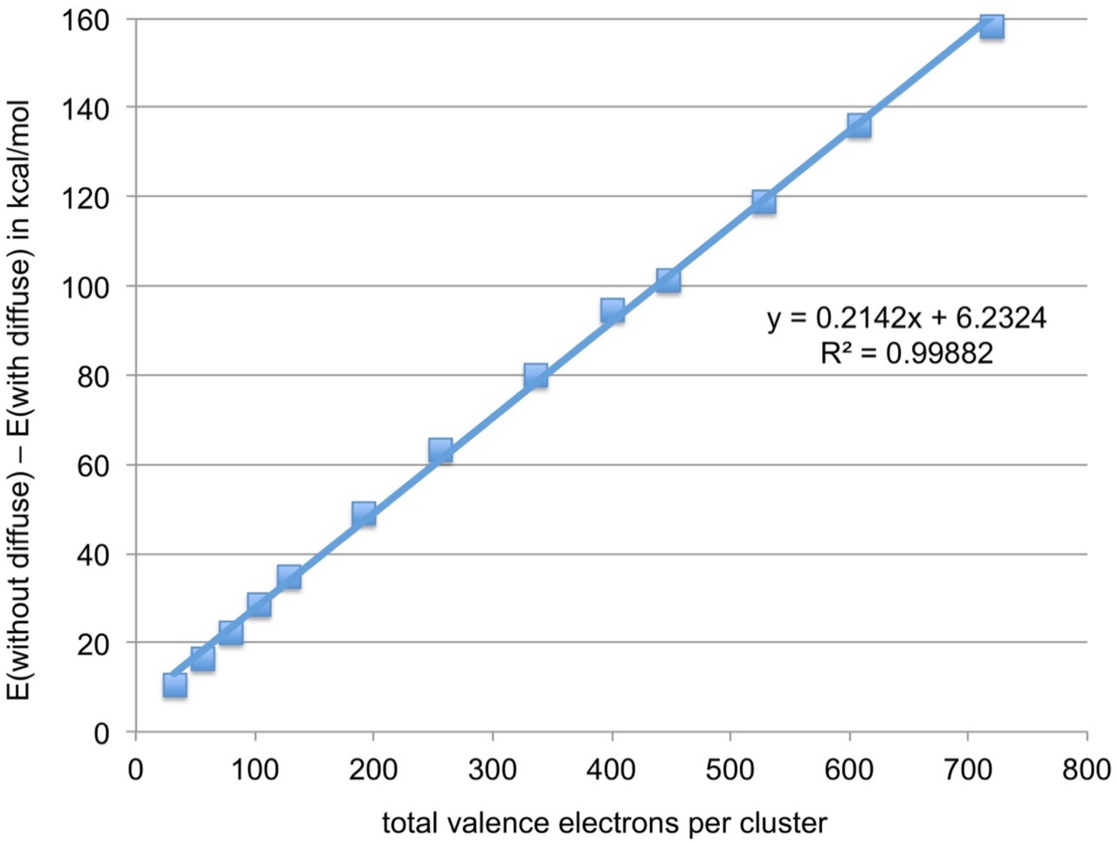

Figure S1. $E_{without\ diffuse} - E_{with\ diffuse}$ plotted for 13 silica clusters versus the total number of valence electrons per cluster. The use of diffuse functionals (++) always lowers the computational electronic energy (E) and the energy difference is proportional to the number of valence electrons. Data points are for clusters *Si1*–*Si35*, as described in Table S1.

CIF Files and Model Complex Construction

Model silica structures were built as pdb files in CrystalMaker (version 7.2.4) according to the quartz silica (SiO_2) crystal structure obtained at 1.0 atm published by Levien, *et al.*,[2] with the CIF file obtained here:
http://rruff.geo.arizona.edu/AMS/download.php?id=00865.cif&down=cif.
The CIF parameters and fractional coordinates for quartz are provided in Table S2. Truncation of the extended crystal was performed to create well-behaved clusters with no intramolecular hydrogen bonding. All silicon and oxygen atoms are restricted to the atomic coordinates of quartz silica. For the cluster complexes described in Table S1, hydrogen atoms were installed in place of silicon atoms at the periphery. All Si-O-H bond angles are fixed at 120°, O-H bond lengths are fixed at 0.958 Å, and O-Si-O-H dihedral angles are left unaltered from the original O-Si-O-Si dihedral angle.

Model alumina structures were built as pdb files in CrystalMaker (version 7.2.4) according to the alumina (corundum, Al_2O_3) crystal structure published by Kirfel and Eichhorn,[3] with the CIF file obtained here:
http://rruff.geo.arizona.edu/AMS/download.php?id=10785.cif&down=cif.
The CIF parameters and fractional coordinates for alumina (corundum) are provided in Table S2. Truncation of the extended crystal was performed to create well-behaved clusters with all silicon and oxygen atoms restricted to the atomic coordinates of corundum. All three clusters have Al_xO_y with an x:y ratio of 2:3.

The S_8 ring of α-sulfur was built as a pdb file in CrystalMaker (version 7.2.4) according to the α-sulfur crystal structure with high-angle refinements published by Rettig and Trotter,[4] with the CIF file obtained here:
http://rruff.geo.arizona.edu/AMS/download.php?id=11686.cif&down=cif.
The CIF parameters and fractional coordinates for α-sulfur are provided in Table S2.

Table S2. CIF parameters and fractional coordinates for quartz silica according to Levien, *et al.*[2] and for alumina according to Kirfel and Eichhorn[3] and for α-sulfur according to Rettig and Trotter.[4]

chemical_name_mineral	'**Quartz**'			
database_code_amcsd	0000789			
chemical_formula_sum	'Si O2'			
cell_length_a	4.916			
cell_length_b	4.916			
cell_length_c	5.4054			
cell_angle_alpha	90			
cell_angle_beta	90			
cell_angle_gamma	120			
symmetry_space_group_name_H-M	'P 32 2 1'			
Si	0.46970	0.00000	0.00000	
O	0.41350	0.26690	0.11910	
chemical_name_mineral	'**Corundum**'			
database_code_amcsd	0009327			
chemical_formula_sum	'Al2 O3'			
cell_length_a	4.7570			
cell_length_b	4.7570			
cell_length_c	12.9877			
cell_angle_alpha	90			
cell_angle_beta	90			
cell_angle_gamma	120			
symmetry_space_group_name_H-M	'R -3 c'			
Al	0.00000	0.00000	0.35218	
O	0.30625	0.00000	0.25000	
chemical_name_mineral	'**Sulphur**'			
database_code_amcsd	0010057			
chemical_formula_sum	'S'			
cell_length_a	10.4646			
cell_length_b	12.8660			
cell_length_c	24.4860			
cell_angle_alpha	90			
cell_angle_beta	90			
cell_angle_gamma	90			
symmetry_space_group_name_H-M	'F d d d'			
S1	0.85585	-0.04732	-0.04860	0.04400
S2	0.70723	-0.02031	0.00406	0.04400
S3	0.78402	0.03022	0.07618	0.04500
S4	0.78595	-0.09239	0.12947	0.04300

Computational Results

Table S3 compiles the computational results for the 13 silica clusters. The average O-O bond order (Mulliken) is computed for the six O-O bonds of the central silicate. The average Si-O bond order is computed for the four Si-O bonds of the central silicate. See Tables S4–S16 for the individual bond orders for the central silicate of each cluster. The # O-O electrons = AVE O-O BO x 6 x 2. The # Si-O electrons = AVE Si-O BO x 4 x 2. The corresponding percentages are given by % O-O e and % Si-O e. Average values for the four largest clusters are provided. Figure S2 plots AVE O-O BO and AVE Si-O BO versus the number of silicon atoms (# Si). Figure S3 plots % O-O e and % Si-O e versus the number of silicon atoms (# Si).

Table S3. Compiled computational results for silica clusters *Si1–Si35*.

cluster	formula	# Si	AVE O-O BO	AVE Si-O BO	# O-O e	# Si-O e	% O-O e	% Si-O e
Si1	SiO_4H_4	1	0.04587	1.02683	0.55044	8.21461	6.280	93.720
Si2	$Si_2O_7H_6$	2	0.09101	0.98392	1.09210	7.87139	12.184	87.816
Si3	$Si_3O_{10}H_8$	3	0.14512	0.91441	1.74142	7.31528	19.228	80.772
Si4	$Si_4O_{13}H_{10}$	4	0.20423	0.84105	2.45072	6.72837	26.699	73.301
Si5	$Si_5O_{16}H_{12}$	5	0.27750	0.74093	3.32994	5.92745	35.971	64.029
Si8	$Si_8O_{24}H_{16}$	8	0.31134	0.78256	3.73603	6.26046	37.373	62.627
Si11	$Si_{11}O_{32}H_{20}$	11	0.36300	0.80461	4.35600	6.43691	40.360	59.640
Si15	$Si_{15}O_{42}H_{24}$	15	0.38251	0.82289	4.59017	6.58314	41.082	58.918
Si18	$Si_{18}O_{50}H_{28}$	18	0.40870	0.80998	4.90434	6.47987	43.080	56.920
Si21	$Si_{21}O_{56}H_{28}$	21	0.46092	0.67706	5.53108	5.41649	50.523	49.477
Si25	$Si_{25}O_{66}H_{32}$	25	0.45746	0.63532	5.48955	5.08253	51.925	48.075
Si29	$Si_{29}O_{76}H_{36}$	29	0.48125	0.65654	5.77501	5.25233	52.370	47.630
Si35	$Si_{35}O_{90}H_{40}$	35	0.47152	0.59284	5.65822	4.74272	54.401	45.599
Average for *Si21–Si35*			**0.468**	**0.640**	**5.613**	**5.124**	**52.30**	**47.70**

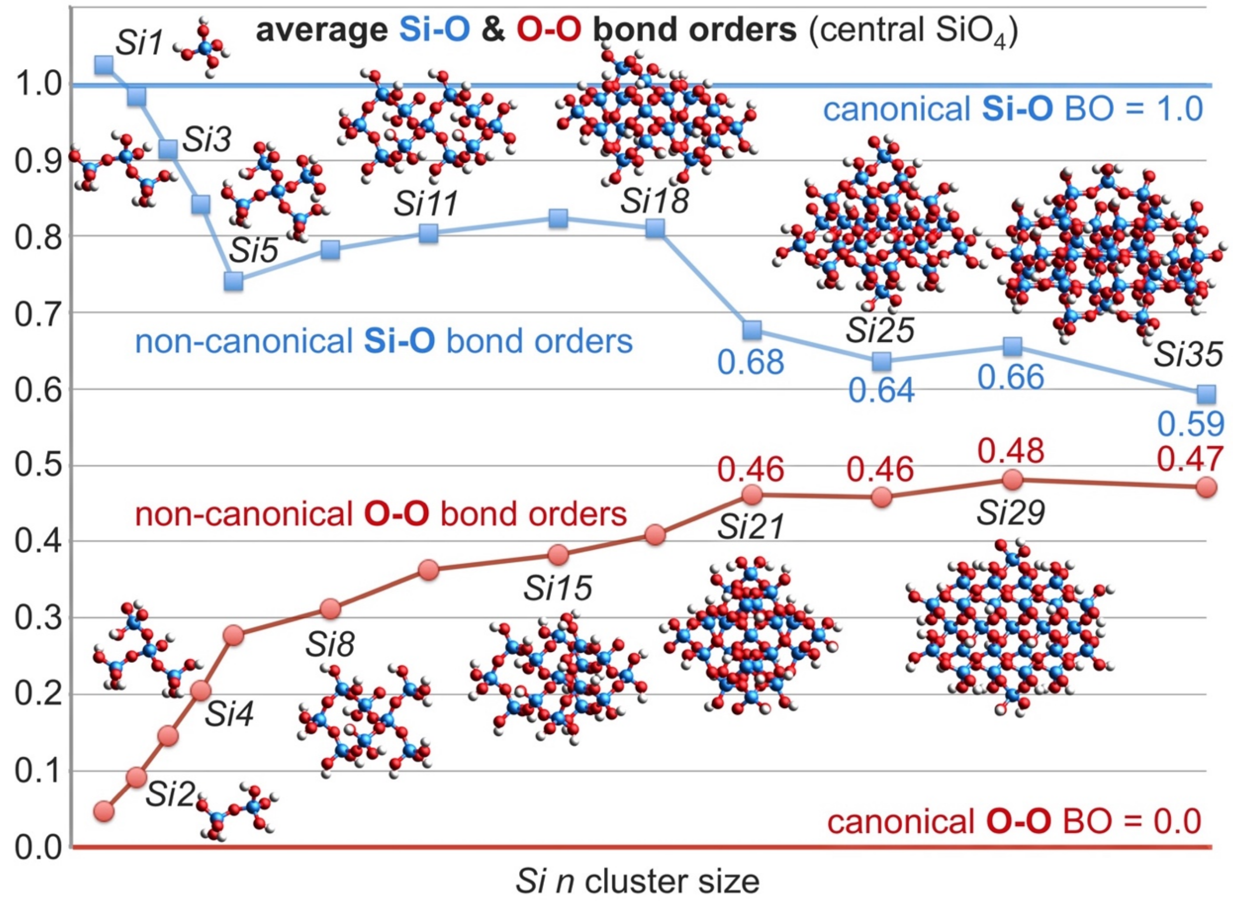

Figure S2. For the central silicate of clusters ranging from *Si1* to *Si35*, average bond orders deviate substantially from the canonical bond order of 1.0 for Si-O (blue) and 0.0 for O-O (red).

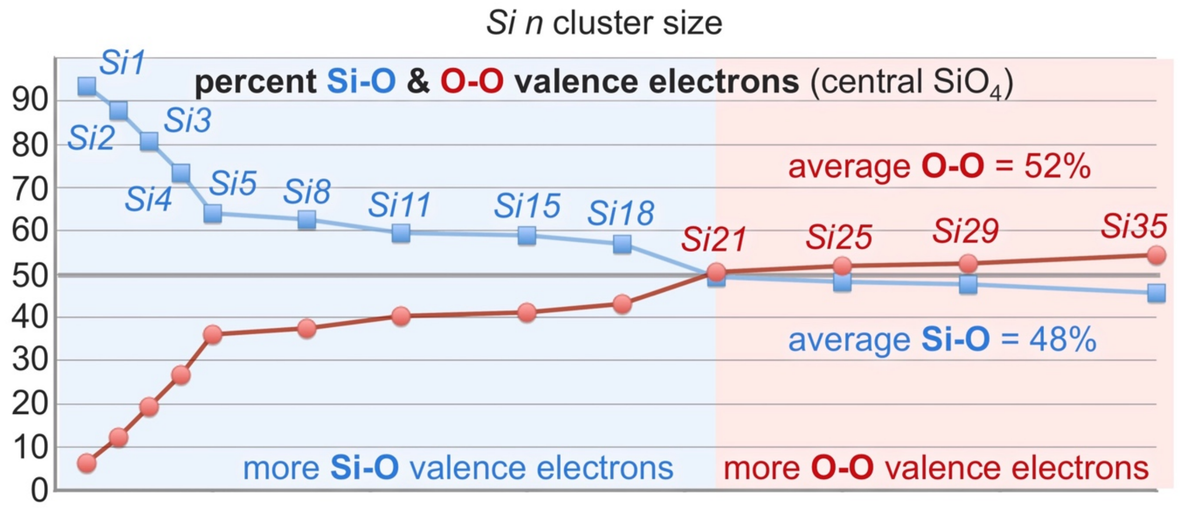

Figure S3. For the central silicate of large clusters (*Si21*–*Si35*), more valence electrons belong to O-O bonds (52%) than Si-O bonds (48%).

Table S4. Computational results for *Si1* (SiO_4H_4, silicic acid).

Structure

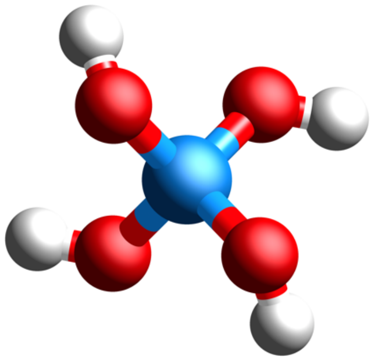

Labeled Structure

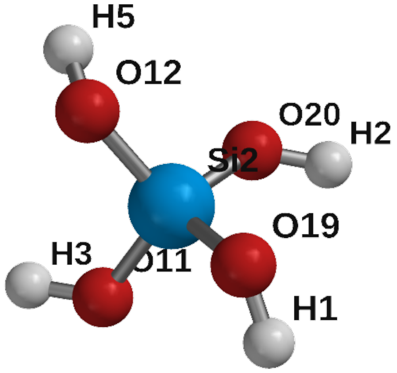

SPARTAN '18 Quantum Mechanics Program: (x86/Darwin) build 1.4.4
SPARTAN file name: Si1.spartan
Job type: Single point.
Method: RB3LYP
Basis set: 6-311++G**
Number of basis functions: 146
Number of electrons: 50
Parallel Job: 3 threads
SCF total energy: **-593.1208008** hartrees
Reason for exit: Successful completion
Quantum Calculation CPU Time: 18.81
Quantum Calculation Wall Time: 18.96
Cartesian Coordinates (Ångstroms)

atom#	type	label	X	Y	Z
1	O	O20	0.7479078	-0.9796416	0.9608887
2	O	O19	0.2415416	1.5697605	0.4758237
3	O	O12	0.7105011	-0.0836683	-1.4957903
4	O	O11	-1.5374235	-0.2528116	-0.1150248
5	Si	Si2	0.0391188	0.0605915	-0.0423451
6	H	H3	-1.9095409	-0.6582861	-0.8991694
7	H	H5	1.0568716	-0.929907	-1.7815727
8	H	H1	-0.3960796	1.9601189	1.074842
9	H	H2	1.047103	-0.6861562	1.8223479

Mulliken bond orders in *Si1*

atom#1	atom#2	Bond Order	# electrons (BO x 2)
Si2	O11	1.04422	2.08844
Si2	O12	1.01069	2.02138
Si2	O19	1.00875	2.01750
Si2	O20	1.04364	2.08728
Si-O average		**1.02683**	2.05365
Si-O total		4.10730	**8.21461**
atom#1	atom#2	Bond Order	# electrons (BO x 2)
O11	O12	0.05438	0.10876
O11	O19	0.03709	0.07418
O11	O20	0.03628	0.07255
O12	O19	0.05640	0.11279
O12	O20	0.03715	0.07430
O19	O20	0.05393	0.10786
O-O average		**0.04587**	0.09174
O-O total		0.27522	**0.55044**
grand total bonding		4.38253	**8.76505**
percent Si-O		93.72001	93.72001
percent O-O		6.27999	6.27999
grand total non-bonding		3.61747	**7.23495**

Oxygen-oxygen bonding vs anti-bonding interactions in *Si1*, $Si(OH)_4$. The percent bonding excess (% *be*) is calculated.

valence molecular orbital	eV	bonding	anti-bonding
HOMO	-8.62	0	6
HOMO{-1}	-9.16	2	4
HOMO{-2}	-9.17	3	3
HOMO{-3}	-9.69	6	0
HOMO{-4}	-10.5	5	1
HOMO{-5}	-10.55	5	1
HOMO{-6}	-11.02	3	3
HOMO{-7}	-11.12	6	0
HOMO{-8}	-14.23	2	4
HOMO{-9}	-14.6	2	4
HOMO{-10}	-15.16	6	0
HOMO{-11}	-16.61	6	0
HOMO{-12}	-27.44	2	4
HOMO{-13}	-27.49	2	4
HOMO{-14}	-27.6	2	4
HOMO{-15}	-28.68	6	0
	sum 2*s*	12	12
	% be_{2s}		**0.000**
	sum 2*p*	46	26
	% be_{2p}		**27.778**
	sum (2*s*+2*p*)	58	38
	% $be_{O\text{-}O}$		**20.833**

be_{2s} = (12-12)/(12+12) = 0.0 %
be_{2p} = (46-26)/(46+26) = 27.8 %
$be_{O\text{-}O}$ = (58-38)/(58+38) = 20.8 %

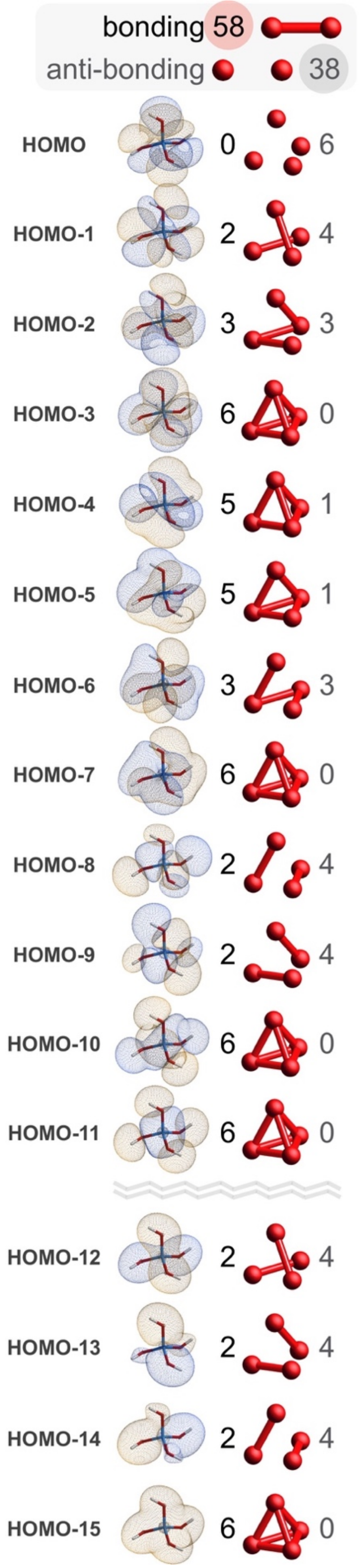

Figure S4. Oxygen-based valence molecular orbitals for $Si(OH)_4$ (IsoValue = 0.01 [e/bohr^3]$^{0.5}$). Bonding and anti-bonding O-O interactions are tabulated. Bonding interactions > anti-bonding interactions and $be_{O\text{-}O}$ = 21%.

Table S5. Computational results for *Si2* ($Si_2O_7H_6$, pyrosilicic acid).

Structure

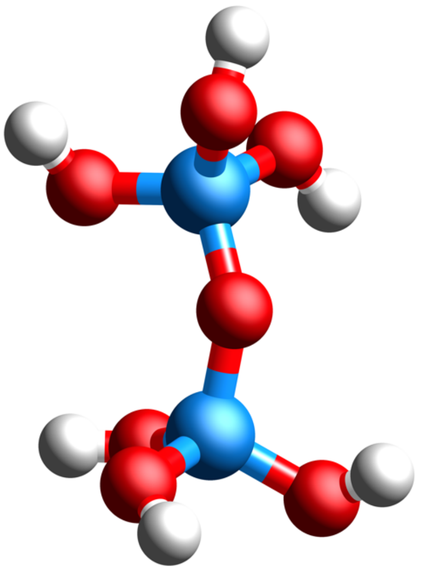

Labeled Structure

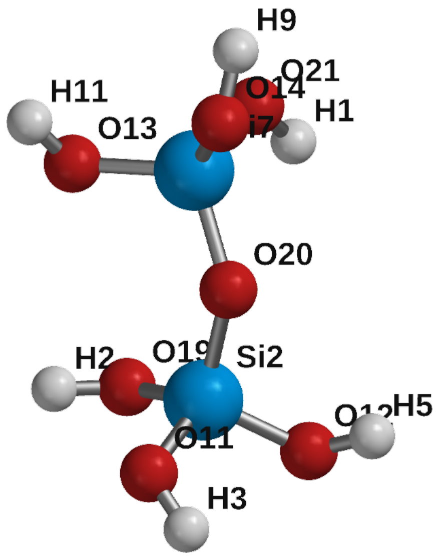

SPARTAN '18 Quantum Mechanics Program:	(x86/Darwin) build 1.4.4
SPARTAN file name:	Si2.spartan
Job type:	Single point.
Method:	RB3LYP
Basis set:	6-311++G**
Number of basis functions:	256
Number of electrons:	90
SCF total energy:	**-1109.7947211** hartrees
Reason for exit:	Successful completion
Quantum Calculation CPU Time:	1:36.27
Quantum Calculation Wall Time:	1:51.44

Cartesian Coordinates (Ångstroms)

atom#	type	label	X	Y	Z
1	O	O21	2.2666576	-0.292212	1.0975047
2	O	O14	0.4876507	-2.1140941	1.6894768
3	O	O13	-0.0177929	0.4343429	2.1729586
4	O	O20	0.0195363	-0.4618623	-0.2820509
5	O	O19	-0.4868303	2.0875411	-0.7671162
6	O	O12	-0.0178705	0.4341115	-2.7387312
7	O	O11	-2.2657962	0.264968	-1.357965
8	Si	Si7	0.6908764	-0.6059772	1.1710553
9	Si	Si2	-0.689253	0.5783713	-1.2852853
10	H	H3	-2.6379137	-0.1405066	-2.14211
11	H	H5	0.3285001	-0.4121275	-3.0245136
12	H	H11	-0.3174468	0.1413256	3.0344183
13	H	H9	1.1259421	-2.5052157	2.2872832
14	H	H1	2.6381918	0.1134354	0.3131728
15	H	H2	-1.1244517	2.4778996	-0.1680977

Mulliken bond orders in *Si2*

atom#1	atom#2	Bond Order	# electrons (BO x 2)
O21	Si7	1.03175	2.06350
O13	Si7	1.02073	2.04147
O14	Si7	1.00756	2.01512
O20	Si7	0.87565	1.75129
Si-O average		0.98392	1.96785
Si-O total		3.93569	**7.87139**
atom#1	atom#2	Bond Order	# electrons (BO x 2)
O21	O20	0.18041	0.36081
O14	O20	0.09632	0.19264
O13	O20	0.08593	0.17185
O14	O13	0.06585	0.13169
O21	O14	0.06453	0.12905
O21	O13	0.05303	0.10605
O-O average		0.09101	0.18202
O-O total		0.54605	**1.09210**
grand total bonding		4.48174	**8.96349**
percent Si-O		87.81612	**87.81612**
percent O-O		12.18388	**12.18388**
grand total non-bonding		3.51826	**7.03651**

Table S6. Computational results for *Si3* ($Si_3O_{10}H_8$).

Structure

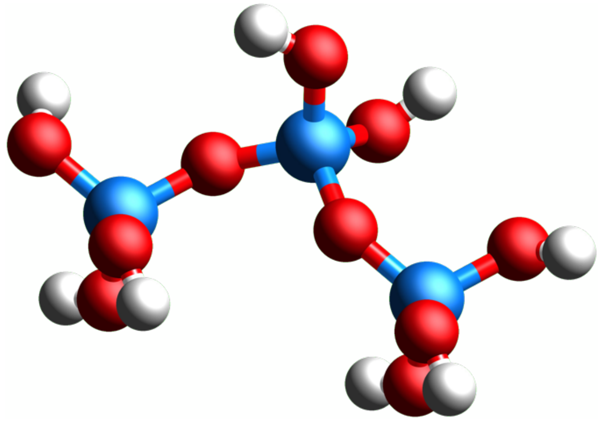

Labeled Structure

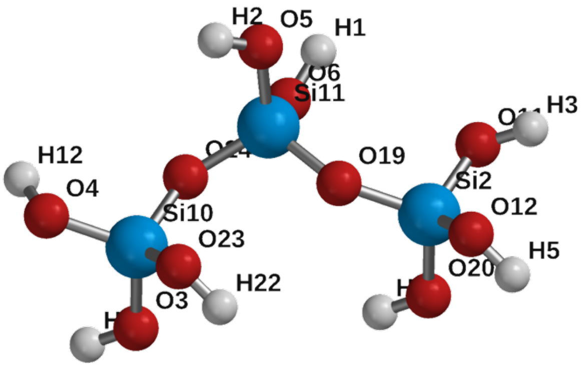

SPARTAN '18 Quantum Mechanics Program:	(x86/Darwin)	build 1.4.4
SPARTAN file name:	Si3.spartan	
Job type:	Single point.	
Method:	RB3LYP	
Basis set:	6-311++G**	
Number of basis functions:	366	
Number of electrons:	130	
SCF total energy:	**-1626.4690133** hartrees	
Reason for exit:	Successful completion	
Quantum Calculation CPU Time:	6:22.04	
Quantum Calculation Wall Time:	7:27.13	

Cartesian Coordinates (Ångstroms)

atom#	type	label	X	Y	Z
1	O	O23	2.310381	1.4588005	-0.2834524
2	O	O24	0.0610749	1.2893536	1.0973613
3	O	O4	1.8408838	3.1113885	1.6882179
4	O	O3	2.3472504	0.5619853	2.1732832
5	O	O20	0.0520574	-3.019335	-0.2828639
6	O	O5	-1.7335294	1.825425	-0.7684095
7	O	O19	-0.4543091	-0.4699317	-0.7679292
8	O	O6	-2.2501522	0.0654278	1.0962148
9	O	O12	0.0146506	-2.1233613	-2.7395441
10	O	O11	-2.233275	-2.2925046	-1.3587779
11	Si	Si10	1.6384611	1.6022188	1.1700489
12	Si	Si11	-1.0940992	0.6772557	0.1608169
13	Si	Si2	-0.6567319	-1.9791014	-1.2860982
14	H	H12	1.2032622	3.5017471	2.2872367
15	H	H2	-1.2998869	2.6761954	-0.8452762
16	H	H1	-3.1554944	0.0398492	0.7840199
17	H	H3	-2.6053925	-2.6979793	-2.1429229
18	H	H5	0.3610212	-2.9696002	-3.0253265
19	H	H10	2.6464457	0.8554709	3.0347429
20	H	H16	0.3512527	-2.7258494	0.5785956
21	H	H22	2.6561298	0.612545	-0.5699373

Mulliken bond orders in *Si3*

atom#1	atom#2	Bond Order	# electrons (BO x 2)
Si11	O5	1.05486	2.10972
Si11	O6	0.92323	1.84647
Si11	O19	0.85373	1.70746
Si11	O24	0.82582	1.65163
Si-O average		0.91441	1.82882
Si-O total		3.65764	**7.31528**
atom#1	atom#2	Bond Order	# electrons (BO x 2)
O24	O5	0.21231	0.42462
O24	O19	0.21172	0.42344
O19	O6	0.15314	0.30628
O24	O6	0.13728	0.27455
O5	O19	0.07947	0.15895
O5	O6	0.07679	0.15358
O-O average		0.14512	0.29024
O-O total		0.87071	**1.74142**
grand total bonding		4.52835	**9.05670**
percent Si-O		80.77205	**80.77205**
percent O-O		19.22795	**19.22795**
grand total non-bonding		3.47165	**6.94330**

Table S7. Computational results for *Si4* ($Si_4O_{13}H_{10}$).

Structure

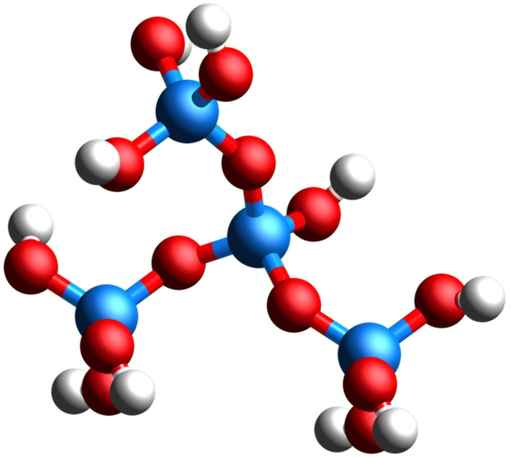

Labeled Structure

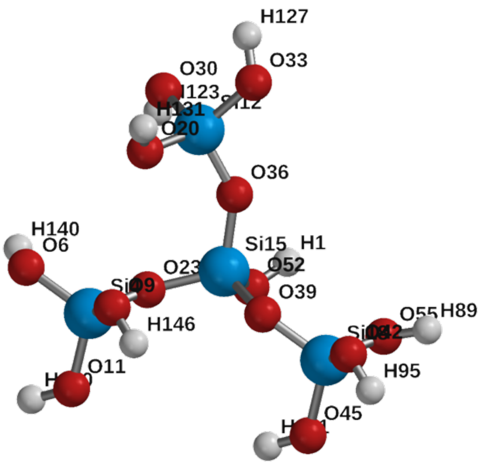

SPARTAN '18 Quantum Mechanics Program:	(x86/Darwin)	build 1.4.4
SPARTAN file name:	Si4.spartan	
Job type:	Single point.	
Method:	RB3LYP	
Basis set:	6-311++G**	
Number of basis functions:	476	
Number of electrons:	170	
Parallel Job:	3 threads	
SCF total energy:	**-2143.1403630** hartrees	
Reason for exit:	Successful completion	
Quantum Calculation CPU Time:	36:33.80	
Quantum Calculation Wall Time:	26:16.59	

Cartesian Coordinates (Ångstroms)

atom#	type	label	X	Y	Z
1	O	O23	-0.5264825	1.4497227	-0.1326027
2	O	O36	-0.0304823	-0.4282778	1.6603977
3	O	O52	1.743518	1.4277227	1.1643975
4	O	O39	1.223518	-0.4222778	-0.6496029
5	O	O9	-1.8824827	0.0877224	-1.9486031
6	O	O11	-1.1304826	2.5427229	-2.4456032
7	O	O6	-3.015483	2.0597228	-0.6566028
8	O	O30	-1.9104827	0.0567224	3.4553981
9	O	O33	-1.1874826	-2.4002782	2.9293979
10	O	O20	-2.5264828	-1.0012779	1.1293975
11	O	O55	3.7125185	-1.0332779	-0.1256027
12	O	O42	2.3565182	-2.3942782	-1.9416031
13	O	O45	3.1095183	0.0607224	-2.4386032
14	Si	Si15	0.6025179	0.5027225	0.5103974
15	Si	Si4	-1.6354826	1.5367227	-1.295603
16	Si	Si12	-1.4164826	-0.9412778	2.2933978
17	Si	Si18	2.6035184	-0.9452778	-1.288603
18	H	H89	4.2490605	-1.8216307	-0.0340237
19	H	H95	2.8840665	-2.6791823	-2.6887891
20	H	H101	2.7063647	0.9248981	-2.5304273
21	H	H123	-1.4978514	0.915332	3.5568329
22	H	H127	-1.7187428	-2.6873841	3.6731028
23	H	H131	-3.0722182	-1.7820458	1.0277188
24	H	H140	-3.0006062	2.6536154	0.0949519

25	H	H146	-1.3549344	-0.1971817	-2.695789
26	H	H150	-1.5332962	3.4071469	-2.5365793
27	H	H1	2.2483565	1.1081523	1.913248

Mulliken bond orders in *Si4*

atom#1	atom#2	Bond Order	# electrons (BO x 2)
Si15	O39	0.88421	1.76843
Si15	O36	0.86215	1.72431
Si15	O52	0.84831	1.69662
Si15	O23	0.76951	1.53901
Si-O average		0.84105	1.68209
Si-O total		3.36418	**6.72837**
atom#1	atom#2	Bond Order	# electrons (BO x 2)
O23	O36	0.25877	0.51753
O36	O52	0.23329	0.46657
O23	O39	0.20989	0.41979
O23	O52	0.18439	0.36878
O52	O39	0.18010	0.36019
O36	O39	0.15893	0.31786
O-O average		0.20423	0.40845
O-O total		1.22536	**2.45072**
grand total bonding		4.58954	**9.17909**
percent Si-O		73.30107	**73.30107**
percent O-O		26.69893	**26.69893**
grand total non-bonding		3.41046	**6.82091**

Table S8. Computational results for *Si5* ($Si_5O_{16}H_{12}$).

Structure Labeled Structure

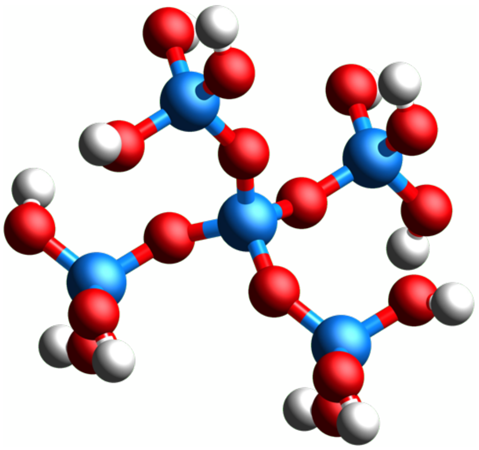

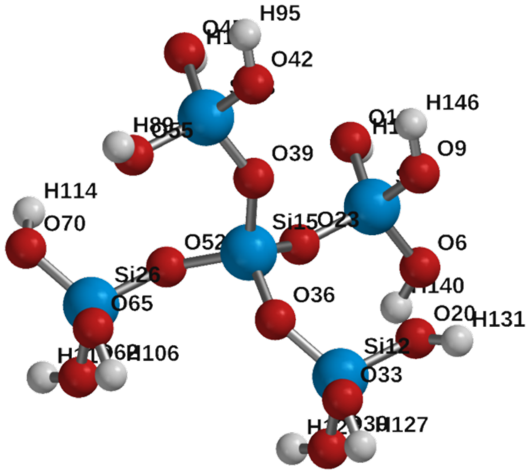

SPARTAN '18 Quantum Mechanics Program:	(x86/Darwin)	build 1.4.4
SPARTAN file name:	Si5.spartan	
Job type:	Single point.	
Method:	RB3LYP	
Basis set:	6-311++G**	
Number of basis functions:	586	
Number of electrons:	210	

Parallel Job:	3 threads
SCF total energy:	**-2659.8115700** hartrees
Reason for exit:	Successful completion
Quantum Calculation CPU Time:	38:39.88
Quantum Calculation Wall Time:	17:09.25

Cartesian Coordinates (Ångstroms)

atom#	type	label	X	Y	Z
1	O	O23	-1.1277166	1.1287942	-0.6422027
2	O	O36	-0.6317165	-0.7492063	1.1507977
3	O	O52	1.142284	1.1067942	0.6547975
4	O	O39	0.6222839	-0.7432063	-1.1592029
5	O	O9	-2.4837168	-0.2332061	-2.4582031
6	O	O11	-1.7317167	2.2217944	-2.9552032
7	O	O6	-3.6167171	1.7387943	-1.1662029
8	O	O30	-2.5117168	-0.2642061	2.945798
9	O	O33	-1.7887167	-2.7212067	2.4197979
10	O	O20	-3.1277169	-1.3222064	0.6197975
11	O	O70	3.6382845	1.6807942	1.1857977
12	O	O62	1.758284	2.1657944	2.980798
13	O	O65	2.4812842	-0.2912061	2.4557979
14	O	O55	3.1112844	-1.3542064	-0.6352027
15	O	O42	1.7552841	-2.7152068	-2.4512031
16	O	O45	2.5082842	-0.2602061	-2.9482032
17	Si	Si15	0.0012837	0.1817939	0.0007974
18	Si	Si4	-2.2367168	1.2157942	-1.805203
19	Si	Si12	-2.0177168	-1.2622064	1.7837977
20	Si	Si26	2.2512842	1.1677942	1.8187978
21	Si	Si18	2.0022842	-1.2662064	-1.798203
22	H	H89	3.6478264	-2.1425593	-0.5436237
23	H	H95	2.2828323	-3.0001108	-3.198389
24	H	H101	2.1051305	0.6039696	-3.0400273
25	H	H106	1.949288	-0.5787656	3.1988012
26	H	H110	2.1709499	3.0244468	3.0817293
27	H	H114	3.6310692	2.2834358	0.4411264
28	H	H123	-2.0990855	0.5944035	3.0472329
29	H	H127	-2.319977	-3.0083126	3.1635028
30	H	H131	-3.6734524	-2.1029744	0.5181188
31	H	H140	-3.6018403	2.3326869	-0.4146481
32	H	H146	-1.9561686	-0.5181102	-3.205389
33	H	H150	-2.1345303	3.0862184	-3.0461794

Mulliken bond orders in *Si5*

atom#1	atom#2	Bond Order	# electrons (BO x 2)
Si15	O23	0.65819	1.31639
Si15	O36	0.82422	1.64845
Si15	O39	0.82410	1.64820
Si15	O52	0.65721	1.31442
Si-O average		0.74093	1.48186
Si-O total		2.96373	**5.92745**
atom#1	atom#2	Bond Order	# electrons (BO x 2)
O23	O36	0.29540	0.59081
O23	O39	0.29128	0.58256
O23	O52	0.27316	0.54633
O36	O39	0.21831	0.43662
O36	O52	0.29043	0.58087
O39	O52	0.29638	0.59276
O-O average		0.27750	0.55499
O-O total		1.66497	**3.32994**
grand total bonding		4.62870	**9.25739**
percent Si-O		64.02938	**64.02938**
percent O-O		35.97062	**35.97062**
grand total non-bonding		3.37130	**6.74261**

Table S9. Computational results for *Si8* ($Si_8O_{24}H_{16}$).

Structure

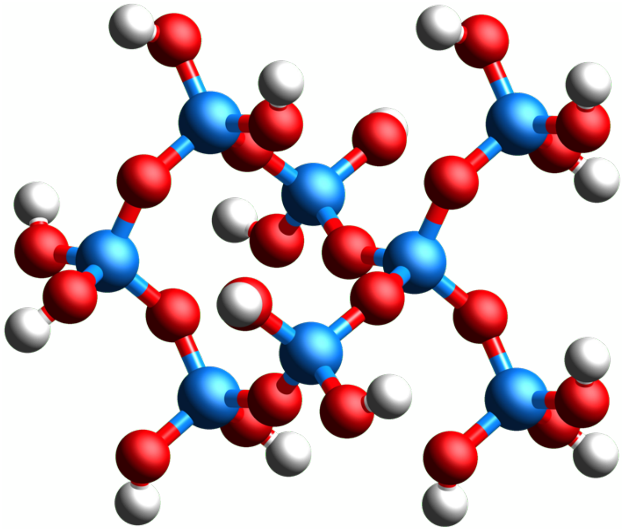

Labeled Structure

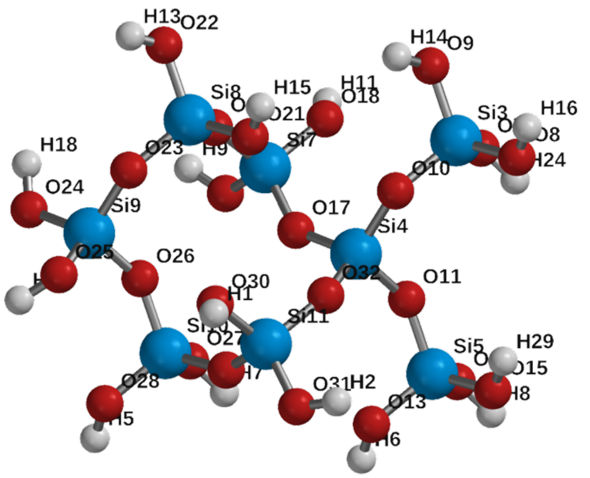

SPARTAN '18 Quantum Mechanics Program: (x86/Darwin) build 1.4.4
SPARTAN file name: Si8.spartan
Job type: Single point.
Method: RB3LYP
Basis set: 6-311++G**
Number of basis functions: 880
Number of electrons: 320
SCF total energy: **-4133.3797225** hartrees

Reason for exit: Successful completion
Quantum Calculation CPU Time: 2:22:52.28
Quantum Calculation Wall Time: 4:14:53.21
Cartesian Coordinates (Ångstroms)

atom#	type	label	X	Y	Z
1	O	O12	-3.90777	-3.2545322	-0.1860409
2	O	O15	-4.4077701	-1.4985319	-2.0610411
3	O	O7	-3.92677	1.0094684	2.2619595
4	O	O8	-4.4267701	2.7654687	0.3859592
5	O	O13	-2.0917698	-2.7475321	-2.0400412
6	O	O27	1.4972308	-3.2545322	-0.1440409
7	O	O19	0.9592307	-1.4865319	2.8969596
8	O	O18	-0.8457696	0.3874683	3.3679597
9	O	O11	-2.6207699	-0.9815318	-0.1820409
10	O	O17	-0.3127695	-0.3545318	0.9159593
11	O	O29	0.9972307	-1.4985319	-2.0200411
12	O	O10	-2.1107698	1.5174685	0.4069592
13	O	O32	-0.8087695	0.3754684	-1.549041
14	O	O20	1.4782308	1.0094684	2.3029595
15	O	O31	-0.2757695	-0.3665318	-4.0010414
16	O	O30	1.5162308	0.9974684	-2.6140413
17	O	O9	-2.6397699	3.2824687	2.2659595
18	O	O21	0.9782307	2.7654687	0.4269593
19	O	O28	3.313231	-2.7475321	-1.9990411
20	O	O26	2.784231	-0.9815318	-0.1400409
21	O	O24	5.0922313	-0.3545318	0.9569593
22	O	O23	3.294231	1.5174685	0.4479592
23	O	O25	4.5972312	0.3764683	-1.507041
24	O	O22	2.765231	3.2824687	2.3069595
25	Si	Si5	-3.2577699	-2.120532	-1.116041
26	Si	Si7	0.3192306	-0.1115317	2.3699595
27	Si	Si4	-1.4637697	0.1404683	-0.1030408
28	Si	Si11	0.3572306	-0.1235317	-2.5460412
29	Si	Si3	-3.2757699	2.1434686	1.3309594
30	Si	Si10	2.1482309	-2.120532	-1.075041
31	Si	Si9	3.9422311	0.1404683	-0.0610408
32	Si	Si8	2.1292309	2.1434686	1.3719594
33	H	H5	3.4081593	-3.6981069	-2.0708763
34	H	H17	5.3440158	-0.1542754	-1.7870195
35	H	H18	5.1805273	0.057991	1.8170715
36	H	H13	3.5127876	3.7948629	1.9965153
37	H	H11	-0.9464308	-0.0207084	4.2287867
38	H	H9	1.6998233	-1.4683462	3.5043771
39	H	H2	-1.0183824	0.1626167	-4.294797
40	H	H1	1.616154	1.5360817	-3.3999645
41	H	H16	-4.5210215	3.7156366	0.3081012
42	H	H14	-1.8922132	3.7948629	1.9555153
43	H	H15	0.8839792	3.7156366	0.3491012
44	H	H8	-4.6506326	-3.7682568	-0.5054255
45	H	H6	-1.997197	-3.6980866	-2.1126103
46	H	H7	0.7548367	-3.7687763	-0.4636786
47	H	H24	-4.669164	0.4952244	1.9423219
48	H	H29	-4.5016195	-0.5483418	-2.139112

Mulliken bond orders in *Si8*

atom#1	atom#2	Bond Order	# electrons (BO x 2)
Si4	O5	0.92179	1.84358
Si4	O6	0.91047	1.82095
Si4	O19	0.67041	1.34082
Si4	O24	0.62756	1.25511
Si-O average		0.78256	1.56511
Si-O total		3.13023	**6.26046**
atom#1	atom#2	Bond Order	# electrons (BO x 2)
O11	O10	0.35234	0.70468
O10	O32	0.34662	0.69324
O11	O32	0.33540	0.67079
O17	O32	0.32440	0.64880
O17	O10	0.30956	0.61912
O11	O17	0.19970	0.39940
O-O average		0.31134	0.62267
O-O total		1.86801	**3.73603**
grand total bonding		4.99824	**9.99649**
percent Si-O		62.62659	**62.62659**
percent O-O		37.37341	**37.37341**
grand total non-bonding		3.00176	**6.00351**

Table S10. Computational results for *Si11* ($Si_{11}O_{32}H_{20}$).

Structure

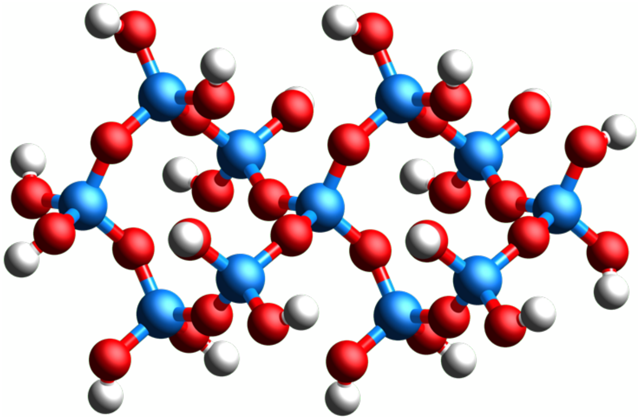

Labeled Structure

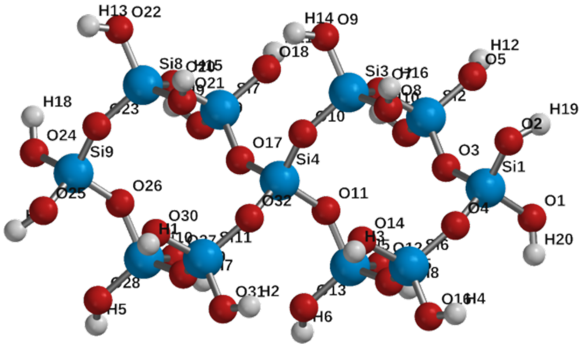

SPARTAN '18 Quantum Mechanics Program:	(x86/Darwin)	build 1.4.4
SPARTAN file name:	Si11.spartan	
Job type:	Single point.	
Method:	RB3LYP	
Basis set:	6-311++G**	
Number of basis functions:	1174	
Number of electrons:	430	
Parallel Job:	3 threads	
SCF total energy:	**-5606.9472951** hartrees	
Reason for exit:	Successful completion	
Quantum Calculation CPU Time:	6:45:54.06	
Quantum Calculation Wall Time:	2:21:21.62	

Cartesian Coordinates (Ångstroms)

atom#	type	label	X	Y	Z
1	O	O12	-2.4438539	-3.2582133	-0.1627726
2	O	O6	-2.9818539	-1.4902132	2.8782277
3	O	O5	-4.7878541	0.383787	3.3502277
4	O	O1	-6.5618542	-0.9852131	-0.1997726
5	O	O3	-4.254854	-0.3582131	0.8972275
6	O	O15	-2.9438539	-1.5022132	-2.0377727
7	O	O2	-6.0528542	1.513787	0.3892275
8	O	O4	-4.749854	0.371787	-1.5667727
9	O	O7	-2.4628539	1.005787	2.2852276
10	O	O16	-4.2168541	-0.3702131	-4.0187729
11	O	O14	-2.4258539	0.993787	-2.6317728
12	O	O8	-2.9628539	2.7617871	0.4092275
13	O	O13	-0.6278538	-2.7512133	-2.0167728
14	O	O27	2.9611465	-3.2582133	-0.1207726
15	O	O19	2.4231464	-1.4902132	2.9202277
16	O	O18	0.6181463	0.383787	3.3912277
17	O	O11	-1.1568538	-0.9852131	-0.1587726
18	O	O17	1.1511464	-0.3582131	0.9392275
19	O	O29	2.4611464	-1.5022132	-1.9967727
20	O	O10	-0.6468538	1.513787	0.4302275
21	O	O32	0.6551464	0.371787	-1.5257727
22	O	O20	2.9421465	1.005787	2.3262276
23	O	O31	1.1881464	-0.3702131	-3.9777728
24	O	O30	2.9801465	0.993787	-2.5907728
25	O	O9	-1.1758538	3.2787872	2.2892276
26	O	O21	2.4421464	2.7617871	0.4502275
27	O	O28	4.7771467	-2.7512133	-1.9757727
28	O	O26	4.2481466	-0.9852131	-0.1167726
29	O	O24	6.5561468	-0.3582131	0.9802275
30	O	O23	4.7581466	1.513787	0.4712275
31	O	O25	6.0611467	0.372787	-1.4837727
32	O	O22	4.2291466	3.2787872	2.3302276
33	Si	Si2	-3.621854	-0.1162131	2.3522276
34	Si	Si1	-5.4048541	0.1357869	-0.1207726
35	Si	Si6	-3.583854	-0.1282131	-2.5647727
36	Si	Si5	-1.7938538	-2.1242132	-1.0927726
37	Si	Si7	1.7831464	-0.1152131	2.3932276
38	Si	Si4	0.0001463	0.1367869	-0.0797726
39	Si	Si11	1.8211464	-0.127213	-2.5227727
40	Si	Si3	-1.8118538	2.1397871	1.3542276
41	Si	Si10	3.6121465	-2.1242132	-1.0517726
42	Si	Si9	5.4061466	0.1367869	-0.0377726
43	Si	Si8	3.5931465	2.1397871	1.3952275
44	H	H5	4.8720749	-3.7017881	-2.0476079
45	H	H17	6.8079313	-0.1579568	-1.7637511
46	H	H18	6.6444427	0.0543096	1.8403397
47	H	H13	4.9767032	3.7911812	2.0197835
48	H	H12	-4.8889977	-0.0240859	4.2111422
49	H	H10	-2.2418867	-1.4720429	3.4864073
50	H	H11	0.5174851	-0.0243898	4.2520547
51	H	H9	3.163739	-1.4720274	3.5276451
52	H	H4	-4.9596927	0.1589541	-4.311923
53	H	H3	-2.3247663	1.5314505	-3.4181972
54	H	H2	0.4455336	0.1589353	-4.2715284
55	H	H1	3.0800698	1.5324002	-3.376696

56	H	H19	-6.8025613	1.498794	0.9854477
57	H	H16	-3.0571053	3.711955	0.3313694
58	H	H14	-0.4282972	3.7911812	1.9787834
59	H	H15	2.347895	3.711955	0.3723695
60	H	H20	-6.6506129	-1.5275001	-0.9845088
61	H	H8	-3.1867163	-3.771938	-0.4821572
62	H	H6	-0.533281	-3.7017677	-2.0893419
63	H	H7	2.2187525	-3.7724574	-0.4404103

Mulliken bond orders in *Si11*

atom#1	atom#2	Bond Order	# electrons (BO x 2)
Si4	O11	0.97752	1.95504
Si4	O17	0.97033	1.94066
Si4	O32	0.63548	1.27096
Si4	O10	0.63512	1.27024
Si-O average		0.80461	1.60923
Si-O total		3.21845	**6.43691**
atom#1	atom#2	Bond Order	# electrons (BO x 2)
O11	O10	0.41568	0.83137
O17	O32	0.40880	0.81759
O10	O32	0.40243	0.80486
O11	O32	0.35467	0.70934
O17	O10	0.35440	0.70881
O11	O17	0.24202	0.48403
O-O average		0.36300	0.72600
O-O total		2.17800	**4.35600**
grand total bonding		5.39645	**10.79291**
percent Si-O		59.64016	**59.64016**
percent O-O		40.35984	**40.35984**
grand total non-bonding		2.60355	**5.20709**

Table S11. Computational results for *Si15* ($Si_{15}O_{42}H_{24}$).

Structure

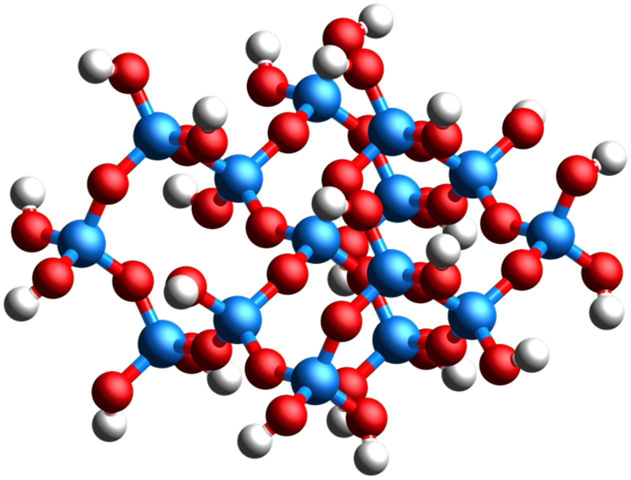

Labeled Structure

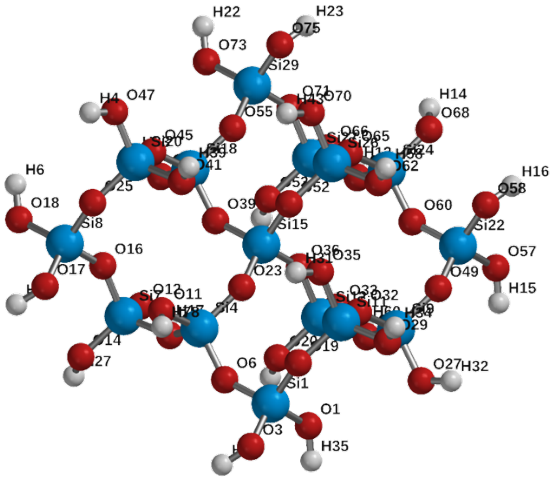

SPARTAN '18 Quantum Mechanics Program: (x86/Darwin) build 1.4.4
SPARTAN file name: Si15.spartan

Job type:	Single point.
Method:	RB3LYP
Basis set:	6-311++G**
Number of basis functions:	1542
Number of electrons:	570
Parallel Job:	3 threads
SCF total energy:	**-7520.7439293** hartrees
Reason for exit:	Successful completion
Quantum Calculation CPU Time:	17:43:15.93
Quantum Calculation Wall Time:	7:24:22.46

Cartesian Coordinates (Ångstroms)

atom#	type	label	X	Y	Z
1	O	O23	-1.1390375	1.0935969	-0.8425461
2	O	O36	-0.6430374	-0.7844032	0.950454
3	O	O52	1.1309627	1.0715969	0.454454
4	O	O39	0.6109627	-0.7784032	-1.3595462
5	O	O9	-2.4950375	-0.2684031	-2.6585463
6	O	O11	-1.7430375	2.1865971	-3.1555463
7	O	O6	-3.6280376	1.703597	-1.3665462
8	O	O30	-2.5230375	-0.2994031	2.7454541
9	O	O33	-1.8000375	-2.7564033	2.2194541
10	O	O20	-3.1390376	-1.3574032	0.419454
11	O	O70	3.6269629	1.645597	0.985454
12	O	O62	1.7469627	2.130597	2.7804541
13	O	O65	2.4699628	-0.3264031	2.2554541
14	O	O55	3.0999629	-1.3894032	-0.8355461
15	O	O42	1.7439628	-2.7504034	-2.6515463
16	O	O45	2.4969628	-0.2954031	-3.1485463
17	O	O16	-0.6150374	-0.7534032	-4.4535464
18	O	O12	-1.7730375	-2.7254033	-3.1835463
19	O	O14	-3.1110375	-1.3274032	-4.9835464
20	O	O3	-5.3790377	3.5755972	-0.8505461
21	O	O1	-4.8810377	1.697597	0.942454
22	O	O19	-3.1080375	3.5545971	0.447454
23	O	O49	-1.1660375	1.0625969	4.5614542
24	O	O32	-1.7700375	2.155597	2.2484541
25	O	O27	-3.6550376	1.672597	4.0374542
26	O	O68	3.0729629	-1.4194032	4.5684542
27	O	O63	1.7169627	-2.7814034	2.7524541
28	O	O60	0.5839627	-0.8094032	4.0444542
29	O	O71	3.5959629	-3.2674033	0.957454
30	O	O75	5.369963	-1.4104032	0.461454
31	O	O73	4.849963	-3.2614033	-1.3525462
32	O	O47	3.6539629	1.676597	-4.4185464
33	O	O41	1.7749628	2.161597	-2.6235463
34	O	O25	1.1579627	1.102597	-4.9485464
35	O	O17	-1.1120375	1.124597	-6.2465466
36	O	O18	0.6379627	-0.7474032	-6.7635466
37	O	O35	-0.6120374	4.1275972	0.978454
38	O	O29	-2.4920375	4.6125972	2.7734541
39	O	O57	-0.6700374	-0.8154032	6.3534544
40	O	O58	1.1029628	1.0405969	5.8584543
41	O	O53	1.0999627	-3.8404034	0.426454
42	O	O66	2.4399628	-5.2384035	2.2274541
43	Si	Si15	-0.0100373	0.1465969	-0.1995461
44	Si	Si4	-2.2480375	1.180597	-2.0055463
45	Si	Si12	-2.0290375	-1.2974032	1.583454

46	Si	Si26	2.2399628	1.132597	1.618454
47	Si	Si18	1.9909628	-1.3014032	-1.9985463
48	Si	Si7	-2.0020375	-1.2664032	-3.8205463
49	Si	Si1	-4.2490377	2.6285971	-0.2065461
50	Si	Si9	-2.2750375	1.149597	3.3974542
51	Si	Si24	1.9639628	-1.3324032	3.4054542
52	Si	Si29	4.228963	-2.3354033	-0.1925461
53	Si	Si20	2.2669628	1.163597	-3.7855463
54	Si	Si8	0.0169627	0.1775969	-5.6035465
55	Si	Si11	-1.9990375	3.6145972	1.611454
56	Si	Si22	-0.0370373	0.1155969	5.2044543
57	Si	Si27	2.2099628	-3.7794034	1.590454
58	H	H35	-5.8321795	1.6255081	1.0313167
59	H	H36	-5.8880022	3.2705822	-1.6026666
60	H	H27	-3.6568883	-2.1081932	-5.0844328
61	H	H5	-1.6216865	0.8197958	-6.99829
62	H	H6	1.5881773	-0.8303304	-6.8528743
63	H	H34	-2.0793717	5.4712496	2.8743854
64	H	H4	3.6467475	2.2796633	-5.1628737
65	H	H32	-3.6397557	2.2666781	4.7888517
66	H	H12	1.9073722	-5.5262996	2.9699009
67	H	H15	-1.6212444	-0.8867242	6.4422394
68	H	H22	5.8001696	-3.3436344	-1.4426
69	H	H16	1.6084438	0.7207907	6.6067704
70	H	H14	3.6094247	-2.2077249	4.6607674
71	H	H23	5.8748015	-1.7299736	1.2103044
72	H	H58	2.1596286	2.9892494	2.8813854
73	H	H60	-2.3312978	-3.0435092	2.9631589
74	H	H78	-2.1458511	3.051021	-3.2465225
75	H	H83	2.271511	-3.0353073	-3.3987322
76	H	H7	0.5541466	-4.6212101	0.3255096
77	H	H9	-3.6847729	-2.1381712	0.3177752
78	H	H17	-2.3050332	-3.0124674	-2.4403513
79	H	H31	-0.6192527	4.7302386	0.2337827
80	H	H39	2.187295	3.0205085	-2.5234601
81	H	H43	3.6197476	2.2482384	0.2407827

Mulliken bond orders in *Si15*

atom#1	atom#2	Bond Order	# electrons (BO x 2)
Si15	O36	0.95281	1.90563
Si15	O39	0.86722	1.73444
Si15	O23	0.78901	1.57801
Si15	O52	0.68253	1.36505
Si-O average		0.80461	1.60923
Si-O total		3.21845	**6.43691**
atom#1	atom#2	Bond Order	# electrons (BO x 2)
O23	O39	0.47519	0.95039
O23	O36	0.42399	0.84799
O23	O52	0.38190	0.76380
O52	O39	0.37887	0.75775
O36	O52	0.37000	0.73999
O36	O39	0.26513	0.53026
O-O average		0.38251	0.76503
O-O total		2.29509	**4.59017**
grand total bonding		5.58665	**11.17331**
percent Si-O		58.91841	**58.91841**
percent O-O		41.08159	**41.08159**
grand total non-bonding		2.41335	**4.82669**

Table S12. Computational results for *Si18* ($Si_{18}O_{50}H_{28}$).

Structure

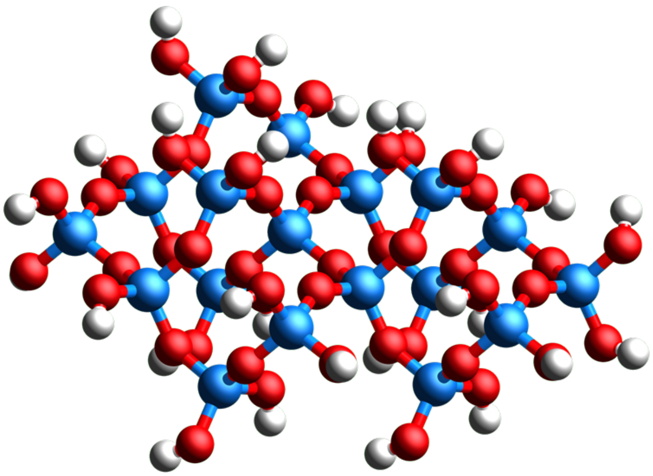

Labeled Structure

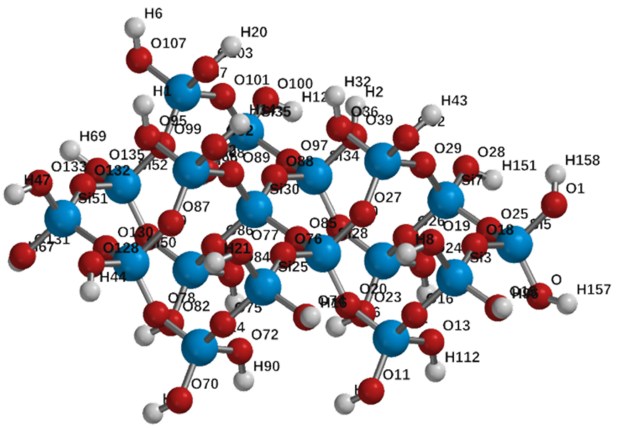

SPARTAN '18 Quantum Mechanics Program:	(x86/Darwin)	build 1.4.4
SPARTAN file name:	Si18.spartan	
Job type:	Single point.	
Method:	RB3LYP	
Basis set:	6-311++G**	
Number of basis functions:	1836	
Number of electrons:	680	
Parallel Job:	3 threads	

SCF total energy: **-8994.3151280** hartrees
Reason for exit: Successful completion
Quantum Calculation CPU Time: 32:57:56.77
Quantum Calculation Wall Time: 11:53:51.64
Cartesian Coordinates (Ångstroms)

atom#	type	label	X	Y	Z
1	O	O	-7.1389892	1.3820089	2.1616298
2	O	O1	-7.6599892	-1.0999913	1.5146298
3	O	O11	-2.2639888	3.0730091	4.13163
4	O	O13	-4.041989	2.609009	2.2306299
5	O	O15	-5.9009891	0.1800088	5.1046301
6	O	O16	-3.565989	0.8090089	4.06963
7	O	O18	-5.355989	-0.5119912	2.6406299
8	O	O19	-4.103989	-1.6949913	4.60363
9	O	O20	-1.7339887	1.346009	2.2386298
10	O	O23	-2.1929888	3.1690091	-0.7823704
11	O	O24	-3.9709889	2.705009	-2.6843705
12	O	O25	-5.8299891	0.2750088	0.1906297
13	O	O26	-3.4949889	0.9050089	-0.8453704
14	O	O27	-2.2559888	-1.1359913	1.5916298
15	O	O28	-5.2859891	-0.4169912	-2.2743705
16	O	O29	-4.033989	-1.5999913	-0.3103703
17	O	O30	-1.6639888	1.4410089	-2.6753705
18	O	O32	-3.5579889	-3.3989914	1.5286298
19	O	O36	-1.7259888	-2.8629914	-0.3023703
20	O	O39	-2.1849888	-1.0399912	-3.3233706
21	O	O70	3.1410116	3.037009	4.20863
22	O	O72	1.3630114	2.5730091	2.3066299
23	O	O74	-0.4959887	0.1440088	5.1816301
24	O	O75	1.8390115	0.7730089	4.14563
25	O	O76	0.0480114	-0.5479912	2.7176299
26	O	O77	1.3000114	-1.7309913	4.68063
27	O	O78	3.6700116	1.3100089	2.3156298
28	O	O82	3.2110116	3.133009	-0.7053704
29	O	O84	1.4330115	2.6690091	-2.6073705
30	O	O85	-0.4259887	0.2390088	0.2676297
31	O	O86	1.9090115	0.8690089	-0.7683703
32	O	O87	3.1490116	-1.1719913	1.6686298
33	O	O88	0.1190113	-0.4529912	-2.1973705
34	O	O89	1.3710115	-1.6349913	-0.2333703
35	O	O90	3.7410116	1.4060089	-2.5993705
36	O	O92	1.8470115	-3.4349914	1.6056298
37	O	O95	3.6790116	-2.8989914	-0.2253703
38	O	O97	-0.3549887	0.3350088	-4.6473707
39	O	O98	1.9800115	0.9650089	-5.6823708
40	O	O99	3.2190116	-1.0759912	-3.2463705
41	O	O100	0.1900114	-0.3569912	-7.1113708
42	O	O101	1.4410115	-1.5399913	-5.1483707
43	O	O103	1.9180115	-3.3399914	-3.3093706
44	O	O107	3.7490116	-2.8029914	-5.1393707
45	O	O128	5.4530117	-0.5839912	2.7946299
46	O	O130	4.9790117	0.2040088	0.3436297
47	O	O131	7.3140119	0.8330089	-0.6913703
48	O	O132	5.5230118	-0.4879912	-2.1203705
49	O	O133	6.7750118	-1.6709913	-0.1563703
50	O	O135	5.0490117	0.2990089	-4.5703707
51	Si	Si2	-2.9009889	1.959009	3.1686299

52	Si	Si3	-4.731989	-0.3059912	4.10363
53	Si	Si5	-6.4959891	0.0120088	1.6266298
54	Si	Si6	-2.8309889	2.055009	-1.7453704
55	Si	Si7	-4.660989	-0.2099912	-0.8103703
56	Si	Si8	-2.8929889	-2.2489913	0.6276297
57	Si	Si24	2.5030115	1.923009	3.2456299
58	Si	Si25	0.6730114	-0.3419912	4.18063
59	Si	Si28	-1.0919887	-0.0239912	1.7026298
60	Si	Si29	2.5740115	2.019009	-1.6693704
61	Si	Si30	0.7430114	-0.2459912	-0.7333703
62	Si	Si32	2.5110115	-2.2849913	0.7046297
63	Si	Si34	-1.0209887	0.0720089	-3.2113705
64	Si	Si35	0.8140114	-0.1499912	-5.6483708
65	Si	Si37	2.5820115	-2.1899913	-4.2093706
66	Si	Si50	4.3130116	-0.0599912	1.7796298
67	Si	Si51	6.1480118	-0.2819911	-0.6563703
68	Si	Si52	4.3830116	0.0360088	-3.1343705
69	H	H96	-6.011267	-0.2466144	5.9552742
70	H	H157	-7.8924137	1.3587298	2.7528775
71	H	H21	2.0365254	-1.731102	5.2932566
72	H	H44	5.5377005	-0.190784	3.664101
73	H	H47	7.5119529	-1.6710942	0.4557424
74	H	H67	7.4232893	1.3876929	-1.4647708
75	H	H69	5.794745	-0.2298659	-4.8566179
76	H	H99	2.0898081	1.5198399	-6.4555922
77	H	H124	-0.5475637	0.1828397	-7.3983025
78	H	H147	-4.0588778	3.657248	-2.7416476
79	H	H151	-6.0231385	0.1234257	-2.5612927
80	H	H158	-7.746597	-1.6241442	0.7174304
81	H	H1	3.7686138	-3.8483274	-0.3175479
82	H	H6	3.8389759	-3.752281	-5.2316732
83	H	H14	1.103271	-3.9369435	1.2699838
84	H	H20	1.1738163	-3.8412167	-3.645095
85	H	H32	-1.6363762	-3.8123563	-0.3942396
86	H	H43	-4.3015565	-3.9009093	1.1925497
87	H	H70	3.8935933	3.5511209	3.9135473
88	H	H79	3.9637831	3.6473201	-0.9996209
89	H	H90	1.2751299	3.5252481	2.2493442
90	H	H95	1.3458348	3.6212595	-2.6655371
91	H	H98	-1.5109164	3.5867783	3.8372029
92	H	H106	-1.440074	3.6827517	-1.0772465
93	H	H112	-4.1291656	3.5612595	2.1724632
94	H	H2	-2.2715966	-1.5641442	-4.12057
95	H	H8	-3.3680006	-1.6947171	5.2168879
96	H	H16	-0.6062666	-0.2826145	6.0322742

Mulliken bond orders in *Si18*

atom#1	atom#2	Bond Order	# electrons (BO x 2)
Si30	O86	0.97273	1.94545
Si30	O88	0.95046	1.90091
Si30	O85	0.69313	1.38625
Si30	O89	0.62362	1.24725
Si-O average		0.80998	1.61997
Si-O total		3.23993	**6.47987**

atom#1	atom#2	Bond Order	# electrons (BO x 2)
O85	O88	0.55992	1.11983
O85	O89	0.46286	0.92571
O88	O89	0.39193	0.78386
O86	O88	0.38260	0.76519
O86	O89	0.33133	0.66266
O85	O86	0.32354	0.64708
O-O average		0.40870	0.81739
O-O total		2.45217	**4.90434**
grand total bonding		5.69211	**11.38421**
percent Si-O		56.91978	**56.91978**
percent O-O		43.08022	**43.08022**
grand total non-bonding		2.30789	**4.61579**

Table S13. Computational results for *Si21* ($Si_{21}O_{56}H_{28}$).

Structure

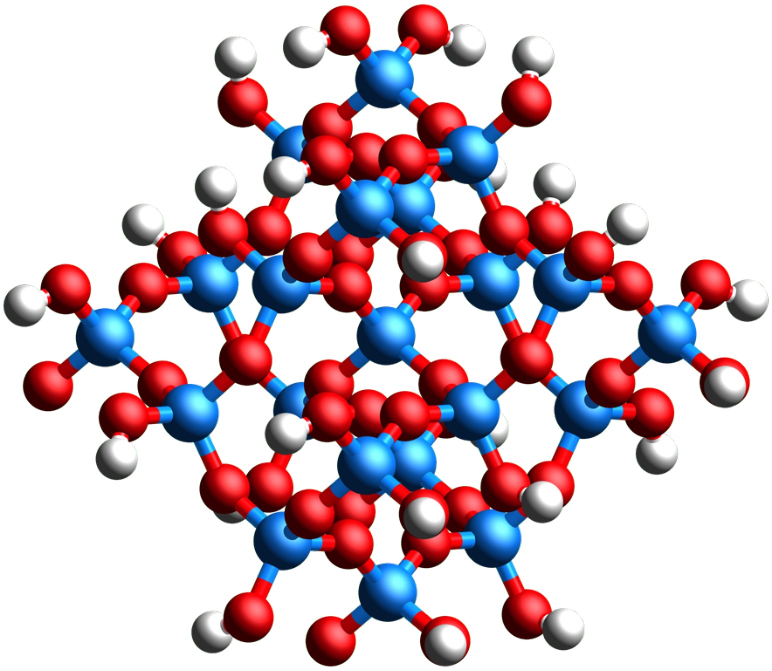

Labeled Structure

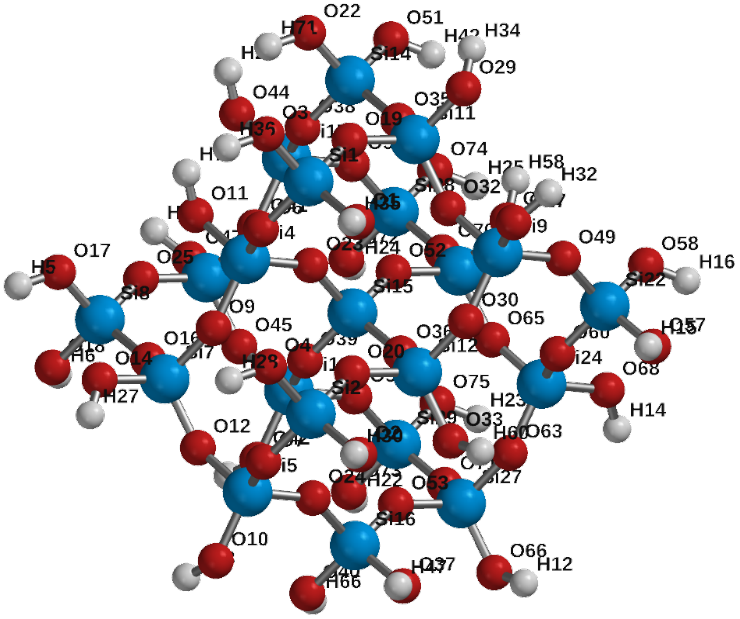

SPARTAN '18 Quantum Mechanics Program: (x86/Darwin) build 1.4.4
SPARTAN file name: Si21.spartan
Job type: Single point.
Method: RB3LYP
Basis set: 6-311++G**
Number of basis functions: 2058
Number of electrons: 770
Parallel Job: 4 threads
SCF total energy: **-10315.0041005** hartrees
Reason for exit: Successful completion
Quantum Calculation CPU Time: 55:13:35.32
Quantum Calculation Wall Time: 13:56:10.97
Cartesian Coordinates (Ångstroms)

atom#	type	label	X	Y	Z
1	O	O23	-1.1280702	1.0824692	-0.6423904
2	O	O36	-0.6320702	-0.7955309	1.1506098
3	O	O52	1.14193	1.0604692	0.6546097
4	O	O39	0.62193	-0.7895309	-1.1593905
5	O	O9	-2.4840702	-0.2795308	-2.4583905
6	O	O11	-1.7320702	2.1754694	-2.9553905
7	O	O6	-3.6170704	1.6924693	-1.1663904
8	O	O30	-2.5120702	-0.3105308	2.9456099
9	O	O33	-1.7890702	-2.767531	2.4196098
10	O	O20	-3.1280703	-1.3685309	0.6196097
11	O	O70	3.6379302	1.6344693	1.1856098
12	O	O62	1.75793	2.1194693	2.9806099
13	O	O65	2.4809301	-0.3375308	2.4556098
14	O	O55	3.1109301	-1.4005309	-0.6353904
15	O	O42	1.75493	-2.7615311	-2.4513905
16	O	O45	2.5079301	-0.3065308	-2.9483906
17	O	O16	-0.6040702	-0.7645309	-4.2533907
18	O	O12	-1.7620702	-2.736531	-2.9833906
19	O	O14	-3.1000703	-1.3385309	-4.7833907
20	O	O3	-5.3680705	3.5644695	-0.6503904

21	O	O1	-4.8700705	1.6864693	1.1426098
22	O	O19	-3.0970703	3.5434694	0.6476097
23	O	O49	-1.1550702	1.0514692	4.76161
24	O	O32	-1.7590702	2.1444693	2.4486098
25	O	O27	-3.6440704	1.6614693	4.23761
26	O	O4	-5.3980704	-1.3475309	-0.6783904
27	O	O2	-4.9010704	-3.226531	1.1146097
28	O	O7	-3.6470703	-3.220531	-1.1953905
29	O	O54	3.1409301	3.5114695	-0.6073904
30	O	O74	5.4119303	3.4904694	0.6906097
31	O	O72	4.8909303	1.6404693	-1.1243905
32	O	O68	3.0839301	-1.4305309	4.76861
33	O	O63	1.72793	-2.7925311	2.9526099
34	O	O60	0.59493	-0.8205309	4.24461
35	O	O71	3.6069302	-3.278531	1.1576098
36	O	O75	5.3809303	-1.4215309	0.6616097
37	O	O73	4.8609303	-3.272531	-1.1523905
38	O	O47	3.6649302	1.6654693	-4.2183907
39	O	O41	1.78593	2.1504693	-2.4233905
40	O	O25	1.16893	1.0914692	-4.7483906
41	O	O17	-1.1010702	1.1134693	-6.0463908
42	O	O18	0.64893	-0.7585309	-6.5633908
43	O	O10	-2.5150702	-5.1915312	-2.4863905
44	O	O24	-1.1580702	-3.8295311	-0.6713904
45	O	O22	-1.0980702	5.9954696	-0.6143904
46	O	O35	-0.6010702	4.1164695	1.1786098
47	O	O29	-2.4810702	4.6014695	2.9736098
48	O	O57	-0.6590702	-0.8265309	6.5536101
49	O	O58	1.11393	1.0294693	6.0586101
50	O	O37	-0.6620702	-5.7085312	1.1216098
51	O	O38	0.65193	4.1224695	-1.1313904
52	O	O44	2.5379301	4.6054695	-2.9203905
53	O	O51	1.17193	5.9734696	0.6826097
54	O	O53	1.11093	-3.8515311	0.6266097
55	O	O66	2.45093	-5.2495312	2.4276098
56	O	O40	0.5919299	-5.7025312	-1.1873905
57	Si	Si15	0.0009299	0.1354692	0.0006097
58	Si	Si4	-2.2370703	1.1694693	-1.8053905
59	Si	Si12	-2.0180703	-1.3085309	1.7836098
60	Si	Si26	2.25093	1.1214693	1.8186097
61	Si	Si18	2.0019301	-1.3125309	-1.7983905
62	Si	Si7	-1.9910703	-1.2775309	-3.6203906
63	Si	Si1	-4.2380704	2.6174694	-0.0063903
64	Si	Si9	-2.2640703	1.1384693	3.5976099
65	Si	Si2	-4.2680704	-2.294531	-0.0343903
66	Si	Si28	4.2699302	2.5654693	0.0356097
67	Si	Si24	1.9749301	-1.3435309	3.6056099
68	Si	Si29	4.2399302	-2.346531	0.0076097
69	Si	Si20	2.27793	1.1524693	-3.5853906
70	Si	Si8	0.0279299	0.1664692	-5.4033907
71	Si	Si5	-2.2670703	-3.7425311	-1.8343905
72	Si	Si11	-1.9880702	3.6034695	1.8116097
73	Si	Si22	-0.0260701	0.1044692	5.40461
74	Si	Si17	2.03193	3.5994695	-1.7703904
75	Si	Si27	2.2209301	-3.7905311	1.7906098
76	Si	Si16	-0.0290701	-4.7765312	-0.0273903
77	Si	Si14	0.0309299	5.0474696	0.0286097

78	H	H35	-5.8212123	1.6143804	1.2314725
79	H	H30	-5.8522594	-3.297603	1.203786
80	H	H36	-5.877035	3.2594545	-1.4025108
81	H	H28	-5.9070349	-1.6525459	-1.4305108
82	H	H27	-3.6459211	-2.1193209	-4.8842771
83	H	H5	-1.6107193	0.8086681	-6.7981342
84	H	H6	1.5991445	-0.841458	-6.6527185
85	H	H33	-1.9876624	-5.4773141	-3.2333397
86	H	H34	-2.0684045	5.4601219	3.0745411
87	H	H2	2.1347765	5.4696452	-3.0122146
88	H	H4	3.6577147	2.2685356	-4.962718
89	H	H32	-3.6287885	2.2555504	4.9890075
90	H	H12	1.9183395	-5.5374272	3.1700567
91	H	H15	-1.6102771	-0.8978519	6.6423952
92	H	H24	5.8411449	1.5575421	-1.2137181
93	H	H22	5.8111368	-3.3547621	-1.2424443
94	H	H16	1.619411	0.7096631	6.8069262
95	H	H14	3.620392	-2.2188526	4.8609232
96	H	H23	5.8857688	-1.7411013	1.4104601
97	H	H25	5.9160908	3.1706967	1.4398304
98	H	H42	1.6773258	5.6540695	1.4311567
99	H	H47	-1.6132597	-5.7801275	1.2103605
100	H	H58	2.1705958	2.9781218	3.0815411
101	H	H60	-2.3203305	-3.0546369	3.1633146
102	H	H66	1.5421352	-5.7848733	-1.2773564
103	H	H71	-1.6082684	5.6908393	-1.3658306
104	H	H78	-2.1348838	3.0398933	-3.0463667
105	H	H83	2.2824782	-3.046435	-3.1985764

Mulliken bond orders in *Si21*

atom#1	atom#2	Bond Order	# electrons (BO x 2)
Si15	O52	0.71251	1.42501
Si15	O23	0.71229	1.42458
Si15	O39	0.64199	1.28398
Si15	O36	0.64146	1.28291
Si-O average		0.67706	1.35412
Si-O total		2.70825	**5.41649**
atom#1	atom#2	Bond Order	# electrons (BO x 2)
O52	O39	0.60805	1.21611
O23	O36	0.60687	1.21374
O23	O39	0.41618	0.83237
O36	O52	0.41443	0.82885
O36	O39	0.36056	0.72112
O23	O52	0.35945	0.71889
O-O average		0.46092	0.92185
O-O total		2.76554	**5.53108**
grand total bonding		5.47379	**10.94757**
percent Si-O		49.47666	**49.47666**
percent O-O		50.52334	**50.52334**
grand total non-bonding		2.52621	**5.05243**

Table S14. Computational results for *Si25* ($Si_{25}O_{66}H_{32}$).

Structure

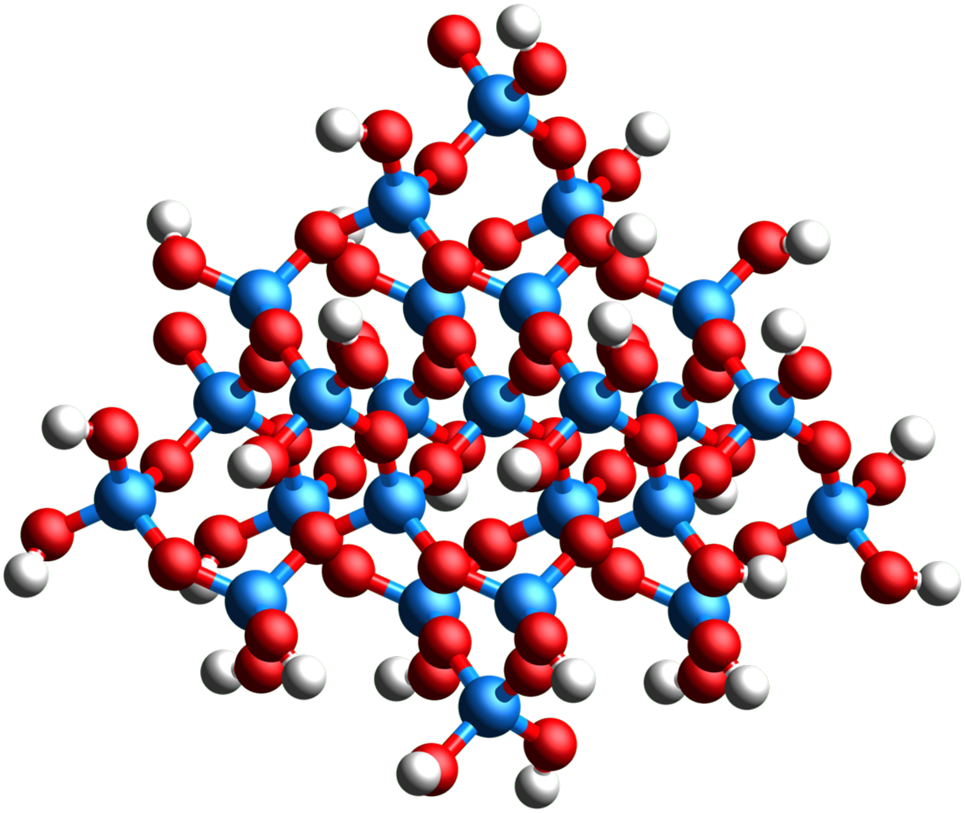

Labeled Structure

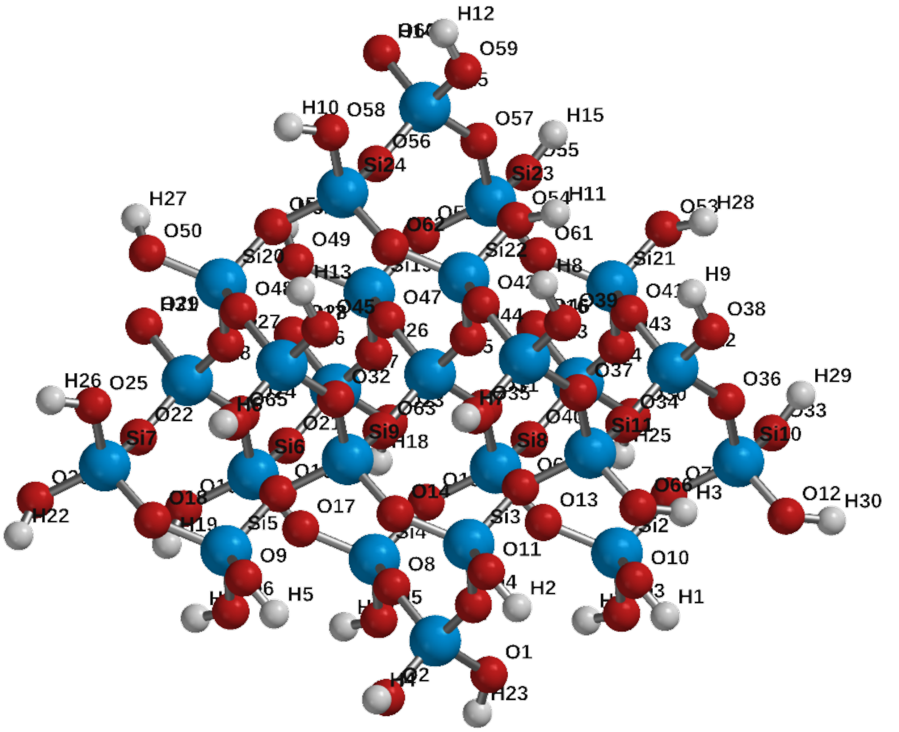

SPARTAN '18 Quantum Mechanics Program:	(x86/Darwin)	build 1.4.4
SPARTAN file name:	Si25.spartan	
Job type:	Single point.	
Method:	RB3LYP	
Basis set:	6-311++G**	
Number of basis functions:	2426	
Number of electrons:	910	
Parallel Job:	3 threads	
SCF total energy:	**-12228.8017947** hartrees	
Reason for exit:	Successful completion	
Quantum Calculation CPU Time:	102:59:06.20	
Quantum Calculation Wall Time:	37:13:37.28	

Cartesian Coordinates (Ångstroms)

atom#	type	label	X	Y	Z
1	O	O50	-3.5172052	3.0366853	4.5257832
2	O	O49	-3.3342052	2.8396853	-0.3862172
3	O	O52	-1.7002051	3.5186854	2.7027831
4	O	O58	0.111795	5.3846855	3.1867831
5	O	O60	-1.1582051	7.0096856	-0.4722171
6	O	O56	-0.520205	4.7546854	0.724783
7	O	O51	-1.517205	3.3226854	-2.2082172
8	O	O59	1.3277951	6.5806855	0.2297829
9	O	O57	0.294795	5.1876854	-1.7252172
10	O	O62	0.8297951	3.0426853	2.2237831
11	O	O55	-0.337205	4.5576854	-4.1862174
12	O	O61	1.0127951	2.8456853	-2.6882173
13	O	O54	2.6467953	3.5246853	0.400783
14	O	O53	2.8297952	3.3286853	-4.5102174
15	O	O20	-5.9212054	-1.8873151	5.2257833
16	O	O25	-4.1092053	-0.0213149	5.7097834
17	O	O29	-5.3792053	1.6036852	2.0507831
18	O	O22	-4.7422053	-0.651315	3.2477831
19	O	O19	-5.7382054	-2.084315	0.3147829
20	O	O27	-2.8942052	1.1746852	2.7527831
21	O	O24	-3.9262053	-0.2183149	0.797783
22	O	O18	-3.3912052	-2.3633151	4.7467832
23	O	O28	-5.1962054	1.4066852	-2.8602173
24	O	O21	-4.5592053	-0.8483149	-1.6632172
25	O	O26	-2.7112052	0.9776851	-2.1592173
26	O	O23	-3.7432052	-0.415315	-4.1132174
27	O	O17	-3.2082052	-2.5603151	-0.1652171
28	O	O48	-1.2152051	1.8056852	4.6607832
29	O	O65	-0.578205	-0.449315	5.8577833
30	O	O16	-1.5742051	-1.8813151	2.9237831
31	O	O45	1.2697952	1.3776852	5.3617833
32	O	O32	0.236795	-0.0153149	3.4077832
33	O	O47	-1.032205	1.6096852	-0.2512171
34	O	O63	-0.395205	-0.646315	0.9457829
35	O	O15	-1.3912051	-2.0783151	-1.9872173
36	O	O44	1.4527951	1.1806852	0.450783
37	O	O31	0.4197951	-0.2123149	-1.5042172
38	O	O14	0.9547951	-2.357315	2.4447831
39	O	O46	-0.849205	1.4126852	-5.1622175
40	O	O40	-0.2122049	-0.842315	-3.9652174
41	O	O43	1.6357951	0.9836852	-4.4612174
42	O	O30	0.6027951	-0.409315	-6.4152176

43	O	O13	1.1377952	-2.5543151	-2.4672173
44	O	O42	3.1307952	1.8116852	2.3587831
45	O	O35	3.7677953	-0.443315	3.5557832
46	O	O64	2.7717952	-1.8753151	0.621783
47	O	O39	5.6167955	1.3826852	3.0597831
48	O	O37	4.5837954	-0.0093149	1.105783
49	O	O41	3.3137953	1.6156852	-2.5532173
50	O	O34	3.9507953	-0.6403149	-1.3562172
51	O	O7	2.9547953	-2.072315	-4.2892174
52	O	O38	5.7997955	1.1866852	-1.8512172
53	O	O36	4.7667954	-0.2063149	-3.8062174
54	O	O66	5.3017954	-2.3513151	0.1427829
55	O	O33	4.1337953	-0.836315	-6.2672176
56	O	O12	5.4847954	-2.5483151	-4.7692175
57	O	O6	-2.7682052	-4.2253152	2.9737831
58	O	O5	-2.5852052	-4.4223152	-1.9382172
59	O	O9	-1.090205	-3.5943152	4.8807833
60	O	O8	-0.907205	-3.7913152	-0.0302171
61	O	O2	-0.2702049	-6.0463153	1.166783
62	O	O4	1.5777952	-4.2193152	0.671783
63	O	O1	0.5457951	-5.6123153	-1.2832172
64	O	O3	1.7607951	-4.4163152	-4.2402174
65	O	O11	3.2557953	-3.5883152	2.5787831
66	O	O10	3.4387953	-3.7853152	-2.3322172
67	Si	Si24	-0.3202049	4.1746854	2.2087831
68	Si	Si25	-0.013205	5.8836855	-0.3112171
69	Si	Si23	-0.137205	3.9786853	-2.7022173
70	Si	Si7	-4.5422053	-1.231315	4.7317832
71	Si	Si18	-4.2342053	0.4766851	2.211783
72	Si	Si6	-4.3592053	-1.427315	-0.1792171
73	Si	Si17	-4.0512053	0.2806851	-2.6992173
74	Si	Si20	-2.3312051	2.3846853	3.6617832
75	Si	Si16	-0.071205	0.6796852	4.8217833
76	Si	Si19	-2.1482051	2.1876853	-1.2502172
77	Si	Si9	-0.195205	-1.225315	2.4297831
78	Si	Si15	0.111795	0.4826852	-0.0902171
79	Si	Si8	-0.0122049	-1.422315	-2.4812172
80	Si	Si14	0.294795	0.2866851	-5.0012175
81	Si	Si22	2.0147952	2.3896852	1.359783
82	Si	Si13	4.2757954	0.6856851	2.519783
83	Si	Si21	2.1977952	2.1936852	-3.5522174
84	Si	Si11	4.1507954	-1.219315	0.1277829
85	Si	Si12	4.4587954	0.4886851	-2.3922172
86	Si	Si10	4.3337953	-1.416315	-4.7832174
87	Si	Si5	-2.2062051	-3.0163151	3.8827832
88	Si	Si4	-2.0232051	-3.2123151	-1.0292172
89	Si	Si1	0.2377951	-4.9173153	0.1307829
90	Si	Si3	2.1397952	-3.0103151	1.580783
91	Si	Si2	2.3227952	-3.2073151	-3.3312174
92	H	H14	-1.6706318	7.0596691	-1.2801061
93	H	H12	1.2720695	7.3487045	0.7997002
94	H	H10	-0.3321711	5.502935	4.0274229
95	H	H15	0.1848549	5.3038396	-4.483659
96	H	H27	-4.0349079	3.756184	4.1623694
97	H	H32	-3.851691	3.5594058	-0.7495007
98	H	H31	-5.891907	1.6536666	1.2430684
99	H	H17	-5.7086322	1.4566687	-3.6681062

100	H	H28	3.7798744	3.4432761	-4.5547449
101	H	H9	5.743658	1.9542344	-1.2807079
102	H	H29	4.657033	-0.0910312	-6.5647718
103	H	H30	6.0551215	-2.6609763	-5.5306633
104	H	H22	-5.9098535	-2.6072426	5.8577166
105	H	H26	-4.5533764	0.0976501	6.5502138
106	H	H6	-0.1984148	-0.4988645	6.7358883
107	H	H13	1.2139475	2.1450516	5.932567
108	H	H2	3.7973505	-4.3323567	2.3125422
109	H	H3	5.8722836	-2.4644852	-0.618466
110	H	H7	4.1475599	-0.4933372	4.4338725
111	H	H8	5.561359	2.1505215	3.6299747
112	H	H4	0.1095596	-6.0963375	2.0448723
113	H	H20	-3.5296362	-4.543459	-2.0437554
114	H	H23	0.0448878	-6.3812259	-1.5582311
115	H	H24	0.816324	-4.5378803	-4.3449088
116	H	H21	-3.7126678	-4.3464681	2.8685382
117	H	H5	-0.5486498	-4.3383567	4.6145423
118	H	H18	-4.2450913	-1.1835303	-4.3883896
119	H	H19	-5.7265148	-2.8036736	0.9473576
120	H	H25	0.1013017	-1.1778787	-6.6901328
121	H	H16	-1.3618782	1.4625934	-5.9699547
122	H	H11	3.5968402	3.6396035	0.3563702
123	H	H1	3.9806502	-4.5292441	-2.598163

Mulliken bond orders in *Si25*

atom#1	atom#2	Bond Order	# electrons (BO x 2)
Si15	O44	0.70197	1.40395
Si15	O31	0.65249	1.30498
Si15	O63	0.61089	1.22178
Si15	O47	0.57591	1.15182
Si-O average		0.63532	1.27063
Si-O total		2.54126	**5.08253**
atom#1	atom#2	Bond Order	# electrons (BO x 2)
O63	O44	0.59402	1.18805
O47	O31	0.51511	1.03023
O47	O44	0.42951	0.85902
O63	O31	0.42389	0.84778
O44	O31	0.41599	0.83198
O47	O63	0.36625	0.73250
O-O average		0.45746	0.91492
O-O total		2.74477	**5.48955**
grand total bonding		5.28604	**10.57207**
percent Si-O		48.07501	**48.07501**
percent O-O		51.92499	**51.92499**
grand total non-bonding		2.71396	**5.42793**

Table S15. Computational results for *Si29* ($Si_{29}O_{76}H_{36}$).

Structure

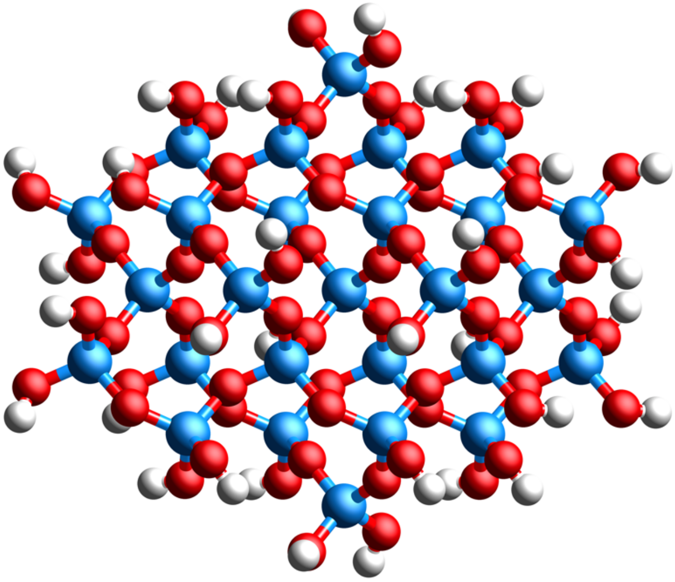

Rotated Structure

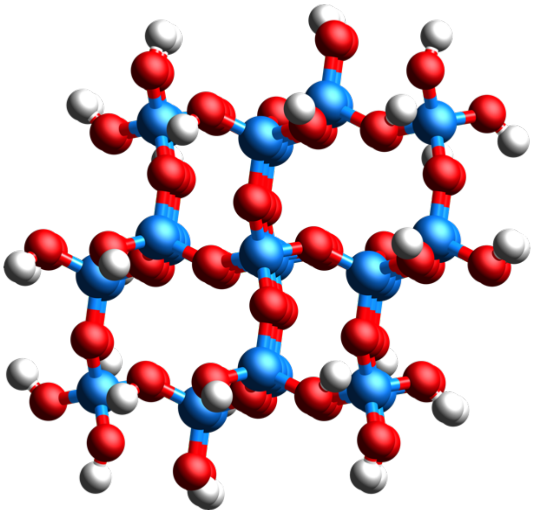

Labeled Structure

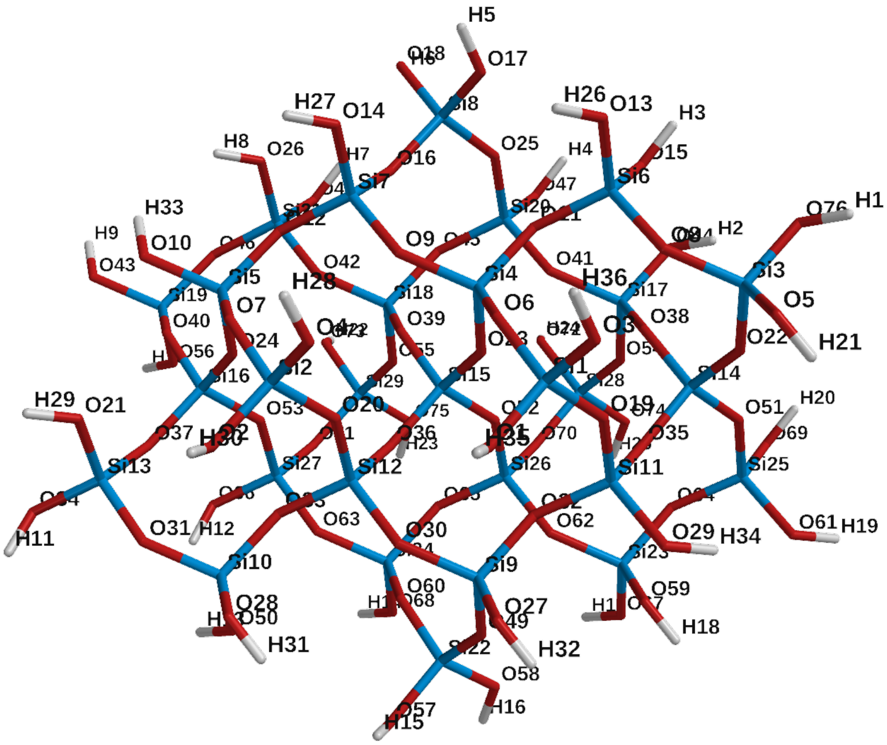

SPARTAN '18 Quantum Mechanics Program:	(x86/Darwin)	build 1.4.4
SPARTAN file name:	Si29.spartan	
Job type:	Single point.	
Method:	RB3LYP	
Basis set:	6-311++G**	
Number of basis functions:	2794	
Number of electrons:	1050	
Parallel Job:	3 threads	

SCF total energy: **-14142.5895089** hartrees
Reason for exit: Successful completion
Quantum Calculation CPU Time: 234:47:18.67
Quantum Calculation Wall Time: 88:07:32.53
Cartesian Coordinates (Ångstroms)

atom#	type	label	X	Y	Z
1	O	O23	-1.1280977	1.0873008	-0.6423689
2	O	O36	-0.6320977	-0.7906993	1.1506313
3	O	O52	1.1419025	1.0653008	0.6546312
4	O	O39	0.6219024	-0.7846993	-1.159369
5	O	O9	-2.4840978	-0.2746992	-2.458369
6	O	O11	-1.7320977	2.180301	-2.9553691
7	O	O6	-3.6170979	1.6973009	-1.1663689
8	O	O30	-2.5120977	-0.3056992	2.9456313
9	O	O33	-1.7890977	-2.7626994	2.4196313
10	O	O20	-3.1280978	-1.3636993	0.6196312
11	O	O70	3.6379027	1.6393009	1.1856313
12	O	O62	1.7579025	2.1243009	2.9806313
13	O	O65	2.4809025	-0.3326992	2.4556313
14	O	O55	3.1109026	-1.3956993	-0.6353689
15	O	O42	1.7549025	-2.7566995	-2.451369
16	O	O45	2.5079025	-0.3016992	-2.9483691
17	O	O16	-0.6040977	-0.7596993	-4.2533692
18	O	O12	-1.7620977	-2.7316994	-2.9833691
19	O	O14	-3.1000978	-1.3336993	-4.7833692
20	O	O15	-0.5740977	4.1523011	-4.2253692
21	O	O8	-2.4540978	4.6373011	-2.430369
22	O	O13	-3.0700978	3.5783011	-4.7553692
23	O	O3	-5.368098	3.5693011	-0.6503689
24	O	O1	-4.870098	1.6913009	1.1426313
25	O	O19	-3.0970978	3.548301	0.6476312
26	O	O49	-1.1550977	1.0563008	4.7616315
27	O	O32	-1.7590977	2.1493009	2.4486313
28	O	O27	-3.6440979	1.6663009	4.2376315
29	O	O50	-1.1850977	-3.8556995	4.7326315
30	O	O31	-2.5420978	-5.2176996	2.9176314
31	O	O28	-3.6740979	-3.2466994	4.2086314
32	O	O4	-5.398098	-1.3426993	-0.6783689
33	O	O2	-4.901098	-3.2216994	1.1146312
34	O	O7	-3.6470979	-3.2156994	-1.195369
35	O	O54	3.1409026	3.5163011	-0.6073689
36	O	O74	5.4119028	3.495301	0.6906312
37	O	O72	4.8909028	1.6453009	-1.124369
38	O	O67	3.1139026	3.4863011	4.7966315
39	O	O64	2.5109025	4.5793011	2.4836313
40	O	O59	0.6249024	4.0963011	4.2726315
41	O	O68	3.0839026	-1.4256993	4.7686315
42	O	O63	1.7279025	-2.7876995	2.9526314
43	O	O60	0.5949024	-0.8156993	4.2446315
44	O	O71	3.6069027	-3.2736994	1.1576313
45	O	O75	5.3809028	-1.4166993	0.6616312
46	O	O73	4.8609028	-3.2676994	-1.152369
47	O	O48	3.6349026	-3.2426994	-4.2463692
48	O	O46	2.4779025	-5.2136996	-2.9763691
49	O	O26	1.1389025	-3.8156995	-4.7763692
50	O	O47	3.6649027	1.6703009	-4.2183692
51	O	O41	1.7859025	2.1553009	-2.423369

52	O	O25	1.1689025	1.0963008	-4.7483692
53	O	O17	-1.1010977	1.1183009	-6.0463693
54	O	O18	0.6489024	-0.7536993	-6.5633693
55	O	O10	-2.5150978	-5.1866996	-2.486369
56	O	O24	-1.1580977	-3.8246995	-0.6713689
57	O	O5	-3.5870979	6.6093013	-1.138369
58	O	O22	-1.0980977	6.0003012	-0.6143689
59	O	O76	-1.7020977	7.0933013	-2.9273691
60	O	O35	-0.6010977	4.1213011	1.1786312
61	O	O29	-2.4810978	4.6063011	2.9736313
62	O	O57	-0.6590977	-0.8216993	6.5536316
63	O	O58	1.1139025	1.0343009	6.0586316
64	O	O21	-3.1580978	-6.2756997	0.5906312
65	O	O34	-1.8190977	-7.6756998	2.3916313
66	O	O37	-0.6620977	-5.7036996	1.1216312
67	O	O38	0.6519024	4.1273011	-1.1313689
68	O	O44	2.5379025	4.6103011	-2.9203691
69	O	O51	1.1719025	5.9783012	0.6826312
70	O	O69	3.6679027	6.5513013	1.2136312
71	O	O61	1.7889025	7.0373013	3.0096314
72	O	O53	1.1109025	-3.8466995	0.6266312
73	O	O66	2.4509025	-5.2446996	2.4276313
74	O	O40	0.5919024	-5.6976996	-1.187369
75	O	O43	1.7249025	-7.6696997	-2.479369
76	O	O56	3.0809026	-6.3076997	-0.6633689
77	Si	Si15	0.0009024	0.1403008	0.0006311
78	Si	Si4	-2.2370978	1.1743009	-1.805369
79	Si	Si12	-2.0180978	-1.3036993	1.7836312
80	Si	Si26	2.2509025	1.1263009	1.8186312
81	Si	Si18	2.0019026	-1.3076993	-1.798369
82	Si	Si7	-1.9910978	-1.2726993	-3.6203691
83	Si	Si6	-1.9610978	3.6393011	-3.5923691
84	Si	Si1	-4.2380979	2.622301	-0.0063689
85	Si	Si9	-2.2640978	1.1433009	3.5976314
86	Si	Si10	-2.2940978	-3.7686995	3.5696314
87	Si	Si2	-4.2680979	-2.2896994	-0.0343688
88	Si	Si28	4.2699027	2.570301	0.0356311
89	Si	Si23	2.0049025	3.5733011	3.6336314
90	Si	Si24	1.9749026	-1.3386993	3.6056314
91	Si	Si29	4.2399027	-2.3416994	0.0076312
92	Si	Si21	2.2479026	-3.7546995	-3.6133691
93	Si	Si20	2.2779025	1.1573009	-3.5853691
94	Si	Si8	0.0279024	0.1713008	-5.4033693
95	Si	Si5	-2.2670978	-3.7376995	-1.834369
96	Si	Si3	-2.2070978	6.0863012	-1.777369
97	Si	Si11	-1.9880978	3.6083011	1.8116312
98	Si	Si22	-0.0260976	0.1093008	5.4046315
99	Si	Si13	-2.0480978	-6.2166997	1.7546313
100	Si	Si17	2.0319025	3.6043011	-1.7703689
101	Si	Si25	2.2809025	6.0383012	1.8466313
102	Si	Si27	2.2209026	-3.7856995	1.7906312
103	Si	Si19	1.9719026	-6.2206997	-1.827369
104	Si	Si16	-0.0290976	-4.7716996	-0.0273688
105	Si	Si14	0.0309024	5.0523012	0.0286311
106	H	H35	-5.8212398	1.619212	1.2314939
107	H	H30	-5.852287	-3.2927714	1.2038075
108	H	H36	-5.8770625	3.2642861	-1.4024894

109	H	H28	-5.9070625	-1.6477143	-1.4304893
110	H	H26	-3.6159486	2.7975111	-4.8562556
111	H	H27	-3.6459486	-2.1144893	-4.8842556
112	H	H5	-1.6107468	0.8134997	-6.7981127
113	H	H21	-3.5722144	7.2037283	-0.3872371
114	H	H1	-2.1048624	7.9578095	-3.0177587
115	H	H3	-0.581313	4.7549425	-4.9700404
116	H	H6	1.599117	-0.8366264	-6.652697
117	H	H8	0.5930517	-4.5964895	-4.8772556
118	H	H33	-1.9876899	-5.4724825	-3.2333182
119	H	H29	-3.7037538	-7.0564272	0.4882192
120	H	H34	-2.068432	5.4649535	3.0745626
121	H	H2	2.134749	5.4744768	-3.0121932
122	H	H4	3.6576872	2.2733672	-4.9626965
123	H	H7	3.6280108	-2.6392909	-4.9904221
124	H	H9	2.2520853	-7.9548491	-3.2267192
125	H	H11	-2.3507226	-7.9625546	3.1351725
126	H	H31	-3.6595265	-2.6520204	4.9595699
127	H	H32	-3.628816	2.260382	4.989029
128	H	H10	3.6176195	-7.0958491	-0.5710677
129	H	H13	-0.6490028	-4.6442315	4.8252789
130	H	H12	1.918312	-5.5325956	3.1700782
131	H	H15	-1.6103046	-0.8930203	6.6424167
132	H	H18	0.6397792	4.6901936	5.0241861
133	H	H19	2.2009428	7.8964194	3.109145
134	H	H24	5.8411173	1.5623737	-1.2136966
135	H	H22	5.8111093	-3.3499305	-1.2424228
136	H	H20	3.6606873	7.1548914	0.4697287
137	H	H16	1.6193835	0.7144947	6.8069477
138	H	H14	3.6203645	-2.214021	4.8609447
139	H	H17	3.6503644	2.6979794	4.8889447
140	H	H23	5.8857412	-1.7362697	1.4104816
141	H	H25	5.9160633	3.1755283	1.4398519

This table is reports **Löwdin bond orders** in *Si29*—for comparison to Mulliken bond orders, given below.

Si29.spartan		-14142.5895089 hartrees	
Si29		**B3LYP**	**6-311++G****
atom#1	atom#2	**Löwdin Bond Order**	# electrons (BO x 2)
Si15	O23	1.07821	2.15642
Si15	O36	1.07782	2.15564
Si15	O39	1.07929	2.15859
Si15	O52	1.07794	2.15588
Si-O average		**1.07832**	2.15663
Si-O total		4.31326	**8.62652**
atom#1	atom#2	**Löwdin Bond Order**	# electrons (BO x 2)
O23	O36	0.09877	0.19753
O23	O39	0.10166	0.20332
O23	O52	0.09399	0.18797
O36	O39	0.09764	0.19529
O36	O52	0.10156	0.20311
O39	O52	0.09886	0.19771
O-O average		**0.09875**	0.19749
O-O total		0.59247	**1.18495**
grand total bonding		4.90573	**9.81147**
percent Si-O		87.92284	**87.92284**
percent O-O		12.07716	**12.07716**
grand total non-bonding		3.09427	**6.18853**

Labeled Si29 cluster center

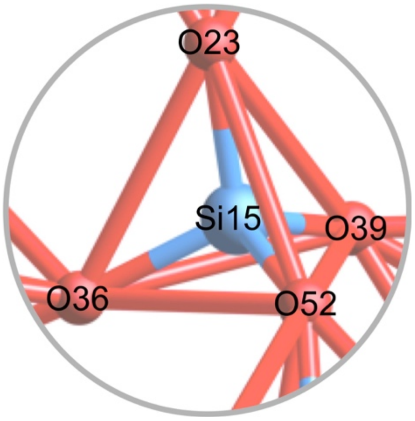

Center bond orders

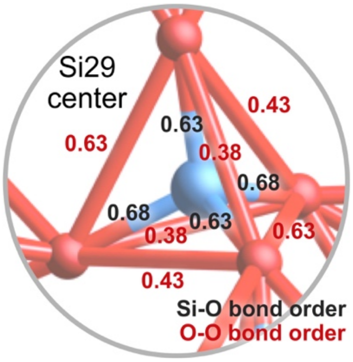

Center atomic charges (electrostatic)

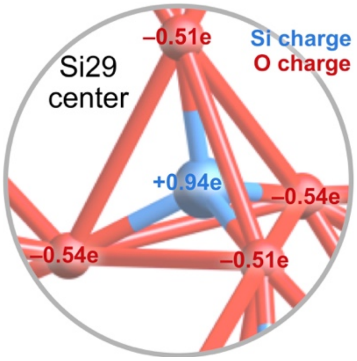

Mulliken bond orders in *Si29*

atom#1	atom#2	Bond Order	# electrons (BO x 2)
Si15	O23	0.63340	1.26680
Si15	O36	0.68037	1.36075
Si15	O39	0.67981	1.35963
Si15	O52	0.63258	1.26515
Si-O average		**0.65654**	1.31308
Si-O total		2.62617	**5.25233**
atom#1	atom#2	Bond Order	# electrons (BO x 2)
O23	O36	0.62605	1.25209
O23	O39	0.43299	0.86598
O23	O52	0.38712	0.77425
O36	O39	0.38141	0.76282
O36	O52	0.43107	0.86214
O39	O52	0.62886	1.25772
O-O average		**0.48125**	0.96250
O-O total		2.88751	**5.77501**
grand total bonding		5.51367	**11.02734**
percent Si-O		47.63008	**47.63008**
percent O-O		52.36992	**52.36992**
grand total non-bonding		2.48633	**4.97266**

Table S16. Computational results for *Si35* ($Si_{35}O_{90}H_{40}$).

Structure

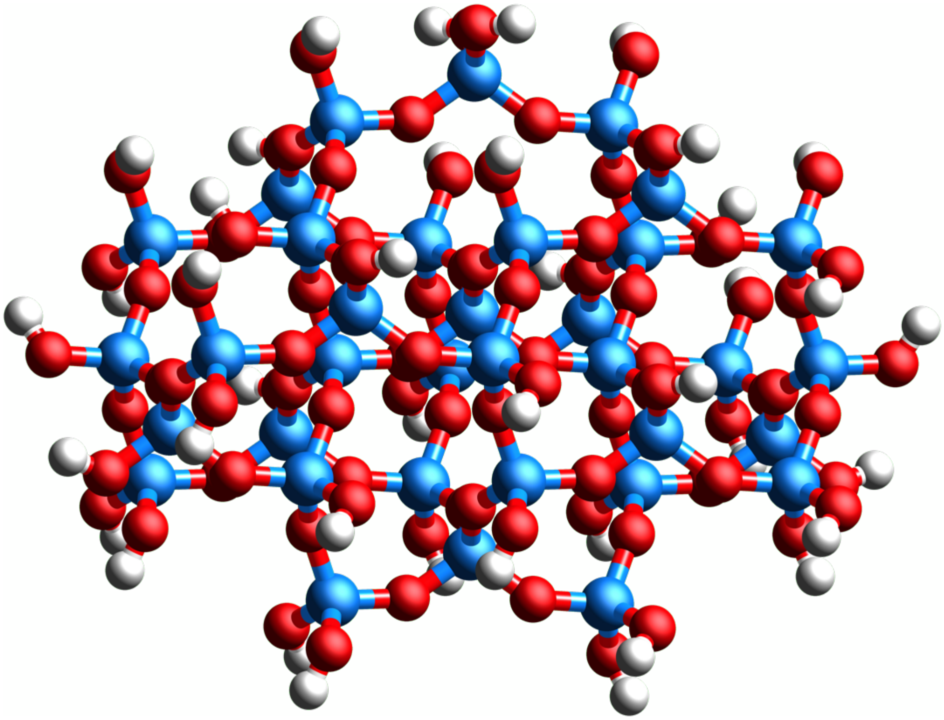

Labeled Structure

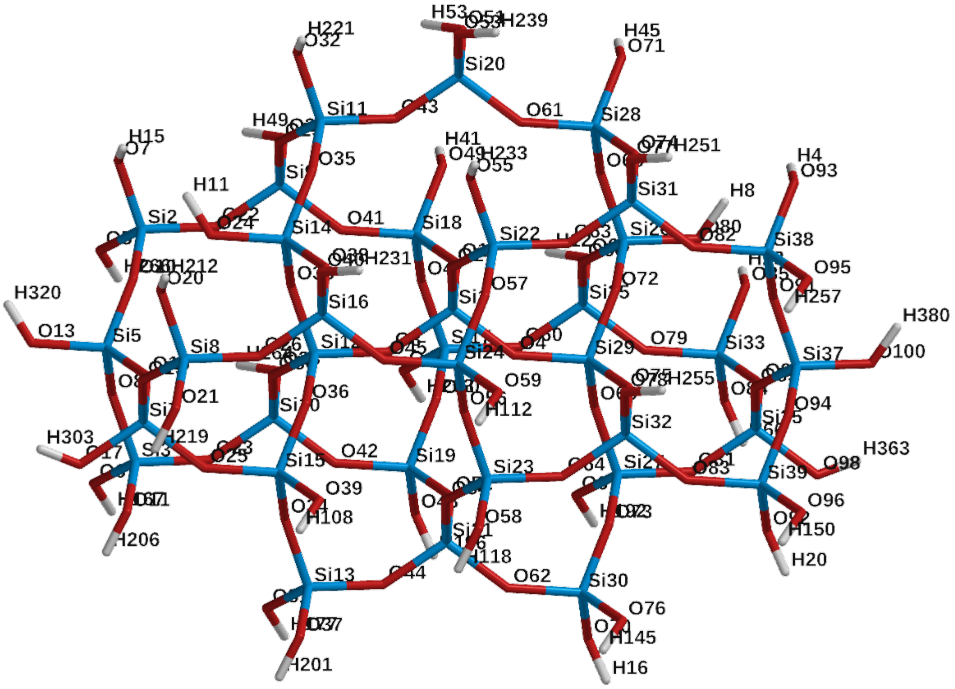

SPARTAN '18 Quantum Mechanics Program: (x86/Darwin) build 1.4.4
SPARTAN file name: Si35.spartan
Job type: Single point.
Method: RB3LYP
Basis set: 6-311++G**

Number of basis functions: 3310
Number of electrons: 1250
Parallel Job: 4 threads
SCF total energy: **-16936.8543296** hartrees
Reason for exit: Successful completion
Quantum Calculation CPU Time: 281:59:26.83
Quantum Calculation Wall Time: 72:31:45.34
Cartesian Coordinates (Ångstroms)

atom#	type	label	X	Y	Z
1	O	O85	-7.8127429	2.4471903	-1.5815209
2	O	O84	-8.3347429	-0.0338098	-2.2295209
3	O	O71	-2.4167425	6.6201907	1.0354793
4	O	O49	-2.4787425	2.3161904	3.4094795
5	O	O68	-2.9377425	4.1391905	0.3884793
6	O	O80	-4.7157426	3.6751905	-1.5135209
7	O	O93	-2.3457425	6.7161907	-3.878521
8	O	O65	-6.5747428	1.2451903	1.3614793
9	O	O66	-4.2397427	1.8751904	0.3254793
10	O	O47	-3.0007426	-0.1658098	2.7624794
11	O	O79	-6.0307427	0.5531903	-1.1035209
12	O	O60	-4.7787427	-0.6298098	0.8604793
13	O	O72	-2.4087425	2.4111904	-1.5045209
14	O	O46	-4.3027426	-2.42881	2.6994795
15	O	O50	-2.4707425	-1.89281	0.8684793
16	O	O91	-2.8677425	4.2341905	-4.5265211
17	O	O100	-4.6457426	3.7711904	-6.4275212
18	O	O87	-6.5047428	1.3411903	-3.5535211
19	O	O89	-4.1697427	1.9711904	-4.5885211
20	O	O69	-2.9297425	-0.0698098	-2.1525209
21	O	O98	-5.9597428	0.6491903	-6.0175212
22	O	O81	-4.7077426	-0.5338098	-4.0545211
23	O	O94	-2.3377425	2.5071904	-6.4195212
24	O	O67	-4.2317427	-2.3338099	-2.214521
25	O	O48	-2.9917425	-4.3738101	0.2214793
26	O	O73	-2.3997425	-1.7968099	-4.0455211
27	O	O92	-2.8597425	0.0261902	-7.0665213
28	O	O70	-2.9217425	-4.2788101	-4.6925211
29	O	O51	-1.1787424	5.4181906	3.9784795
30	O	O53	1.1562578	6.0481906	2.9434794
31	O	O32	2.3962578	4.0071905	5.3794796
32	O	O61	-0.6337424	4.7261905	1.5144794
33	O	O43	0.6182577	3.5431905	3.4774795
34	O	O27	-1.2407424	1.1141903	6.3524797
35	O	O29	1.0942577	1.7431903	5.3164796
36	O	O7	2.3332579	-0.2968098	7.7534798
37	O	O41	-0.6967424	0.4221903	3.8884795
38	O	O22	0.5552577	-0.7608098	5.8514797
39	O	O35	2.9252579	2.2801904	3.4864795
40	O	O5	1.0312577	-2.5608099	7.6904798
41	O	O10	2.8632579	-2.02481	5.8604796
42	O	O74	-1.1077425	5.5141906	-0.9355208
43	O	O77	1.2272578	6.1431906	-1.9715209
44	O	O55	2.4662578	4.1031905	0.4654793
45	O	O82	-0.5637424	4.8221906	-3.400521
46	O	O63	0.6882577	3.6391905	-1.4365209
47	O	O1	-1.1707424	1.2091903	1.4384793
48	O	O2	1.1642578	1.8391903	0.4024793

49	O	O33	2.4042578	-0.2018098	2.8394794
50	O	O4	-0.6257424	0.5171903	-1.0265208
51	O	O3	0.6262577	-0.6648098	0.9374793
52	O	O57	2.9962579	2.3761904	-1.4285209
53	O	O28	-1.2327424	-3.09481	3.8114795
54	O	O30	1.1022578	-2.4648099	2.7764795
55	O	O8	2.3412578	-4.5058101	5.2134796
56	O	O42	-0.6877424	-3.7868101	1.3474793
57	O	O23	0.5632577	-4.9698102	3.3114795
58	O	O36	2.9342579	-1.92881	0.9454793
59	O	O6	1.0402577	-6.7698103	5.1504796
60	O	O11	2.8712579	-6.2328102	3.3194795
61	O	O95	-1.0377424	5.6101906	-5.8505212
62	O	O75	-1.0997424	1.3051903	-3.4765211
63	O	O78	1.2352578	1.9351904	-4.5115211
64	O	O56	2.4742578	-0.1058098	-2.0755209
65	O	O83	-0.5547424	0.6131903	-5.9405212
66	O	O64	0.6962577	-0.5698099	-3.9775211
67	O	O52	-1.1627424	-2.99881	-1.1025209
68	O	O54	1.1732578	-2.3698099	-2.1385209
69	O	O34	2.4122578	-4.4098101	0.2984793
70	O	O62	-0.6177424	-3.6908101	-3.566521
71	O	O44	0.6342577	-4.8738101	-1.6035209
72	O	O58	3.0042579	-1.83281	-3.968521
73	O	O31	1.1102578	-6.6738103	0.2354792
74	O	O37	2.9422579	-6.1368102	-1.5945209
75	O	O96	-1.0297424	1.4011903	-8.3905214
76	O	O76	-1.0917424	-2.90281	-6.0175212
77	O	O24	4.708258	0.3861903	3.9654795
78	O	O13	4.646258	-3.9188101	6.3384797
79	O	O38	4.234258	1.1741903	1.5144794
80	O	O40	6.5692581	1.8031903	0.4794793
81	O	O20	7.8082582	-0.2378098	2.9164794
82	O	O45	4.778258	0.4821903	-0.9495208
83	O	O26	6.0302581	-0.7008099	1.0144793
84	O	O15	4.171258	-3.13081	3.8884795
85	O	O19	6.5062581	-2.50081	2.8534795
86	O	O25	4.716258	-3.82281	1.4244793
87	O	O17	5.9682581	-5.0058102	3.3874795
88	O	O21	8.3382582	-1.9648099	1.0224793
89	O	O59	4.304258	1.2691903	-3.3995211
90	O	O39	4.242258	-3.03481	-1.0255208
91	Si	Si26	-3.5757426	3.0251904	-0.5745208
92	Si	Si25	-5.4057427	0.7601903	0.3604792
93	Si	Si17	-3.6377426	-1.2788099	1.7984793
94	Si	Si33	-7.1707428	1.0781903	-2.1175209
95	Si	Si37	-3.5047426	3.1211904	-5.4895212
96	Si	Si35	-5.3357427	0.8561903	-4.5545211
97	Si	Si27	-3.5677426	-1.1838099	-3.115521
98	Si	Si20	-0.0097423	4.9331906	2.9774795
99	Si	Si11	1.7582578	2.8931904	4.4164796
100	Si	Si9	-0.0717424	0.6281903	5.3514796
101	Si	Si2	1.6962578	-1.4108099	6.7904797
102	Si	Si28	-1.7737424	5.2511906	0.5004793
103	Si	Si31	0.0612577	5.0291906	-1.9365209
104	Si	Si18	-1.8367425	0.9461902	2.8734795
105	Si	Si22	1.8292578	2.9891904	-0.4985208

106	Si	Si1	-0.0017423	0.7241903	0.4374793
107	Si	Si12	1.7662578	-1.3148099	1.8754794
108	Si	Si10	-0.0637423	-3.5798101	2.8114795
109	Si	Si3	1.7042578	-5.6198102	4.2494795
110	Si	Si38	-1.7037425	5.3461906	-4.4145211
111	Si	Si29	-1.7657425	1.0421902	-2.0405209
112	Si	Si32	0.0692577	0.8201903	-4.4775211
113	Si	Si19	-1.8287424	-3.26181	0.3334792
114	Si	Si23	1.8372578	-1.2198099	-3.038521
115	Si	Si21	0.0072577	-3.4838101	-2.1035209
116	Si	Si13	1.7752577	-5.5238102	-0.6645208
117	Si	Si39	-1.6957425	1.1381903	-6.9545212
118	Si	Si30	-1.7577425	-3.16681	-4.5815211
119	Si	Si14	3.5682579	0.9101903	2.9504795
120	Si	Si16	5.403258	0.6881902	0.5144793
121	Si	Si5	3.5052579	-3.3938101	5.3244796
122	Si	Si8	7.1712582	-1.3508099	1.9524794
123	Si	Si7	5.340258	-3.61581	2.8884795
124	Si	Si24	3.6382579	1.0061903	-1.9635209
125	Si	Si15	3.576258	-3.29781	0.4104793
126	H	H303	6.7040552	-5.0061726	4.0009667
127	H	H320	4.7314064	-3.5265104	7.2083158
128	H	H363	-6.6969638	1.189396	-6.304657
129	H	H380	-4.7329257	4.7234408	-6.4856801
130	H	H16	-3.0091166	-4.802849	-5.4897119
131	H	H20	-2.9462236	-0.4981033	-7.863642
132	H	H66	-8.4210164	-0.5583336	-3.0265126
133	H	H201	3.0318893	-7.08609	-1.6872476
134	H	H206	2.9616464	-7.1821119	3.2277173
135	H	H219	8.4278708	-2.9141749	0.9306101
136	H	H12	-6.6841765	0.8188781	2.2123885
137	H	H23	-8.5665321	2.423705	-0.9907465
138	H	H41	-3.2325195	2.2930154	4.0002817
139	H	H45	-3.1701779	6.596601	1.6267008
140	H	H49	-1.3510204	0.687567	7.2031238
141	H	H53	-1.2881761	4.9918784	4.8293887
142	H	H108	4.9879913	-3.5636848	-1.3117681
143	H	H112	5.0499912	0.7403155	-3.6857683
144	H	H118	3.0942221	-2.7820996	-4.0608236
145	H	H145	-0.3462999	-3.4317827	-6.3043444
146	H	H150	-0.2836675	0.872676	-8.6765442
147	H	H167	0.2967316	-7.2719907	4.8147001
148	H	H177	0.366603	-7.1753926	-0.1009087
149	H	H192	-4.9752687	-2.8359905	-2.5503004
150	H	H196	-3.0783608	-4.8977022	-0.5758905
151	H	H203	-5.0463102	-2.9307279	2.3633993
152	H	H212	8.5608585	0.2767561	2.6222365
153	H	H221	3.1488396	4.5213023	5.0843969
154	H	H231	6.6785356	2.3578743	-0.2939212
155	H	H233	3.2190293	4.6175015	0.1712287
156	H	H239	1.2660543	6.6030216	2.170258
157	H	H251	1.3362718	6.698361	-2.7446095
158	H	H255	1.3450544	2.4900213	-5.2847425
159	H	H257	-0.2925247	5.0809814	-6.1374921
160	H	H264	-1.3423773	-3.5207726	4.6625378
161	H	H266	0.2876029	-3.0623923	7.3540919
162	H	H4	-3.0998502	6.6930257	-3.2881405

163	H	H8	-4.8036315	4.6274294	-1.5707979
164	H	H11	4.7929467	0.7793974	4.8349507
165	H	H15	3.0863302	0.2169594	7.4590527

Mulliken bond orders in *Si35*

atom#1	atom#2	Bond Order	# electrons (BO x 2)
Si1	O4	0.69255	1.38511
Si1	O3	0.69028	1.38057
Si1	O2	0.50545	1.01091
Si1	O1	0.48307	0.96614
Si-O average		0.59284	1.18568
Si-O total		2.37136	**4.74272**
atom#1	atom#2	Bond Order	# electrons (BO x 2)
O1	O3	0.62845	1.25691
O2	O4	0.62553	1.25107
O2	O3	0.43466	0.86931
O1	O4	0.42613	0.85225
O4	O3	0.36848	0.73697
O1	O2	0.34586	0.69171
O-O average		0.47152	0.94304
O-O total		2.82911	**5.65822**
grand total bonding		5.20047	**10.40094**
percent Si-O		45.59896	**45.59896**
percent O-O		54.40104	**54.40104**
grand total non-bonding		2.79953	**5.59906**

Table S17. Silicon 3*d* atomic orbital contribution for the clusters *Si1*, *Si5*, and *Si29*. These contributions amount to 0.82%, 0.74%, and 0.36%, respectively, as shown by the MO analysis below where SUMSQ is the sum of the squares of the atomic orbital contributions to each valence MO. The Si 3*d* contribution includes the 3*d* atomic orbitals of all silicon atoms in each cluster (1, 5, or 29 silicon atoms). The maximum contribution to any MO is 2.31% (HOMO-3), 2.25% (HOMO-32), or 1.34% (HOMO-153) for the three clusters, respectively.

	Si1	*Si5*	*Si29*
max SUMSQ Si 3*d* / SUMSQ all (%)	2.30977	2.24664	1.33601
for HOMO-*n*	HOMO-3	HOMO-32	HOMO-153
average SUMSQ Si 3*d* / SUMSQ all (%)	**0.82340**	**0.74092**	**0.36058**

	SUMSQ Si 3*d* / SUMSQ all (%)		
valence HOMO-*n*			
HOMO	0.13896	0.12993	0.06796
HOMO-1	0.67167	0.17891	0.05746
HOMO-2	1.10484	0.15147	0.07464
HOMO-3	2.30977	0.29195	0.07478
HOMO-4	1.86701	0.47050	0.08073
HOMO-5	0.92665	0.36585	0.13131
HOMO-6	1.03466	0.68244	0.07006
HOMO-7	0.45078	0.74162	0.09285
HOMO-8	1.75359	0.58276	0.08786
HOMO-9	1.08968	0.81597	0.08190
HOMO-10	0.78241	0.93227	0.08021
HOMO-11	0.04378	1.26359	0.07863
HOMO-12	0.36913	1.47723	0.11424
HOMO-13	0.32213	1.33215	0.12248

HOMO-14	0.30636	1.15025	0.13983
HOMO-15	0.00294	1.67164	0.09944
HOMO-16		1.39642	0.16646
HOMO-17		1.30980	0.12898
HOMO-18		0.80721	0.15355
HOMO-19		1.10612	0.21705
HOMO-20		0.96830	0.15533
HOMO-21		0.93692	0.17950
HOMO-22		0.78179	0.13927
HOMO-23		0.87677	0.14007
HOMO-24		0.52653	0.19038
HOMO-25		0.68011	0.07761
HOMO-26		0.52243	0.16009
HOMO-27		1.76497	0.22078
HOMO-28		0.91503	0.22525
HOMO-29		1.60419	0.22013
HOMO-30		1.35833	0.20258
HOMO-31		0.96764	0.17681
HOMO-32		2.24664	0.25967
HOMO-33		1.66627	0.16409
HOMO-34		1.72293	0.22554
HOMO-35		1.42864	0.33346
HOMO-36		1.19899	0.25686
HOMO-37		1.05780	0.24120
HOMO-38		0.80894	0.23397
HOMO-39		0.97520	0.33409
HOMO-40		0.86889	0.26459
HOMO-41		0.85080	0.37391
HOMO-42		0.68737	0.30930
HOMO-43		0.46457	0.29696
HOMO-44		0.17036	0.28579
HOMO-45		0.17095	0.45506
HOMO-46		0.17985	0.39271
HOMO-47		0.11957	0.26099
HOMO-48		0.48169	0.39950
HOMO-49		0.42907	0.39268
HOMO-50		0.40856	0.37490
HOMO-51		0.29869	0.30950
HOMO-52		0.30924	0.29680
HOMO-53		0.31326	0.46909
HOMO-54		0.29963	0.30971
HOMO-55		0.29879	0.36091
HOMO-56		0.30628	0.27601
HOMO-57		0.29937	0.26767
HOMO-58		0.30030	0.36824
HOMO-59		0.17032	0.36319
HOMO-60		0.03595	0.43074
HOMO-61		0.03595	0.57036
HOMO-62		0.03457	0.39931
HOMO-63		0.01853	0.47689
HOMO-64			0.59263
HOMO-65			0.36932
HOMO-66			0.33390
HOMO-67			0.31088
HOMO-68			0.52239
HOMO-69			0.36051
HOMO-70			0.69572

HOMO-71	0.38844
HOMO-72	0.50632
HOMO-73	0.38748
HOMO-74	0.37995
HOMO-75	0.26508
HOMO-76	0.47055
HOMO-77	0.34405
HOMO-78	0.47803
HOMO-79	0.35403
HOMO-80	0.43626
HOMO-81	0.39723
HOMO-82	0.56554
HOMO-83	0.69380
HOMO-84	0.61306
HOMO-85	0.40834
HOMO-86	0.73542
HOMO-87	0.65073
HOMO-88	0.52307
HOMO-89	0.46630
HOMO-90	0.47123
HOMO-91	0.52819
HOMO-92	0.69895
HOMO-93	0.47717
HOMO-94	0.42980
HOMO-95	0.43671
HOMO-96	0.56628
HOMO-97	0.44625
HOMO-98	0.56017
HOMO-99	0.78034
HOMO-100	0.42174
HOMO-101	0.31128
HOMO-102	0.39069
HOMO-103	0.58959
HOMO-104	0.49840
HOMO-105	0.34636
HOMO-106	0.44274
HOMO-107	0.57398
HOMO-108	0.43076
HOMO-109	0.40770
HOMO-110	0.58270
HOMO-111	0.54991
HOMO-112	0.42783
HOMO-113	0.38820
HOMO-114	0.38873
HOMO-115	0.37176
HOMO-116	0.26433
HOMO-117	0.46370
HOMO-118	0.42406
HOMO-119	0.42172
HOMO-120	0.39760
HOMO-121	0.60494
HOMO-122	0.37307
HOMO-123	0.58755
HOMO-124	0.40654
HOMO-125	0.41213
HOMO-126	0.54103
HOMO-127	0.31150

HOMO-128	0.44730
HOMO-129	0.70866
HOMO-130	0.82619
HOMO-131	0.51804
HOMO-132	0.73761
HOMO-133	0.45549
HOMO-134	0.44930
HOMO-135	0.65167
HOMO-136	0.54648
HOMO-137	0.40275
HOMO-138	0.54004
HOMO-139	0.46774
HOMO-140	0.68015
HOMO-141	0.41848
HOMO-142	0.44498
HOMO-143	0.59905
HOMO-144	0.61788
HOMO-145	0.46660
HOMO-146	0.55531
HOMO-147	0.55210
HOMO-148	0.73245
HOMO-149	0.58920
HOMO-150	0.67896
HOMO-151	0.51064
HOMO-152	1.29881
HOMO-153	1.33601
HOMO-154	0.80498
HOMO-155	1.12263
HOMO-156	1.03053
HOMO-157	0.97854
HOMO-158	1.01754
HOMO-159	0.64556
HOMO-160	1.11402
HOMO-161	0.86093
HOMO-162	1.10166
HOMO-163	0.78815
HOMO-164	1.08046
HOMO-165	0.96008
HOMO-166	0.95714
HOMO-167	0.93084
HOMO-168	0.75627
HOMO-169	0.62033
HOMO-170	0.60471
HOMO-171	0.84552
HOMO-172	0.57602
HOMO-173	0.58490
HOMO-174	0.59741
HOMO-175	0.59783
HOMO-176	0.44727
HOMO-177	0.61638
HOMO-178	0.57459
HOMO-179	0.40440
HOMO-180	0.47668
HOMO-181	0.40131
HOMO-182	0.63827
HOMO-183	0.36757
HOMO-184	0.40985

HOMO-185	0.44579
HOMO-186	0.41987
HOMO-187	0.46751
HOMO-188	0.41680
HOMO-189	0.53907
HOMO-190	0.55285
HOMO-191	0.62170
HOMO-192	0.48328
HOMO-193	0.52889
HOMO-194	0.58899
HOMO-195	0.47598
HOMO-196	0.51838
HOMO-197	0.41903
HOMO-198	0.49243
HOMO-199	0.39755
HOMO-200	0.22050
HOMO-201	0.27820
HOMO-202	0.25276
HOMO-203	0.28448
HOMO-204	0.32284
HOMO-205	0.25801
HOMO-206	0.27734
HOMO-207	0.22156
HOMO-208	0.25082
HOMO-209	0.24818
HOMO-210	0.24578
HOMO-211	0.19238
HOMO-212	0.16274
HOMO-213	0.19152
HOMO-214	0.22106
HOMO-215	0.17878
HOMO-216	0.12980
HOMO-217	0.14122
HOMO-218	0.12560
HOMO-219	0.10793
HOMO-220	0.10360
HOMO-221	0.10336
HOMO-222	0.10518
HOMO-223	0.10784
HOMO-224	0.06284
HOMO-225	0.07876
HOMO-226	0.07594
HOMO-227	0.04396
HOMO-228	0.26141
HOMO-229	0.26295
HOMO-230	0.32428
HOMO-231	0.31073
HOMO-232	0.28396
HOMO-233	0.35491
HOMO-234	0.30654
HOMO-235	0.27593
HOMO-236	0.29155
HOMO-237	0.27675
HOMO-238	0.24440
HOMO-239	0.24251
HOMO-240	0.23627
HOMO-241	0.25524

HOMO-242	0.24679
HOMO-243	0.21772
HOMO-244	0.26606
HOMO-245	0.20560
HOMO-246	0.26791
HOMO-247	0.24615
HOMO-248	0.20470
HOMO-249	0.26336
HOMO-250	0.26057
HOMO-251	0.17287
HOMO-252	0.18384
HOMO-253	0.27155
HOMO-254	0.20176
HOMO-255	0.19913
HOMO-256	0.24734
HOMO-257	0.23275
HOMO-258	0.18532
HOMO-259	0.26726
HOMO-260	0.22141
HOMO-261	0.21838
HOMO-262	0.25467
HOMO-263	0.17823
HOMO-264	0.18301
HOMO-265	0.26753
HOMO-266	0.23457
HOMO-267	0.23545
HOMO-268	0.18189
HOMO-269	0.22161
HOMO-270	0.21763
HOMO-271	0.24116
HOMO-272	0.23008
HOMO-273	0.19315
HOMO-274	0.19210
HOMO-275	0.17257
HOMO-276	0.16011
HOMO-277	0.11886
HOMO-278	0.11376
HOMO-279	0.10996
HOMO-280	0.15677
HOMO-281	0.08749
HOMO-282	0.07195
HOMO-283	0.06688
HOMO-284	0.07111
HOMO-285	0.06624
HOMO-286	0.05755
HOMO-287	0.05242
HOMO-288	0.05125
HOMO-289	0.05387
HOMO-290	0.05738
HOMO-291	0.05419
HOMO-292	0.03902
HOMO-293	0.03499
HOMO-294	0.03948
HOMO-295	0.03226
HOMO-296	0.02361
HOMO-297	0.02728
HOMO-298	0.02885

HOMO-299	0.02411
HOMO-300	0.01598
HOMO-301	0.02220
HOMO-302	0.01538
HOMO-303	0.00973

Table S18. Computational results for Si(OSi)$_4$ (Si_5O_4).

Structure

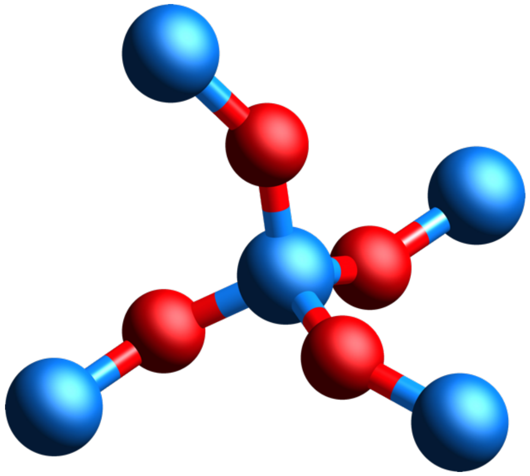

Labeled Structure

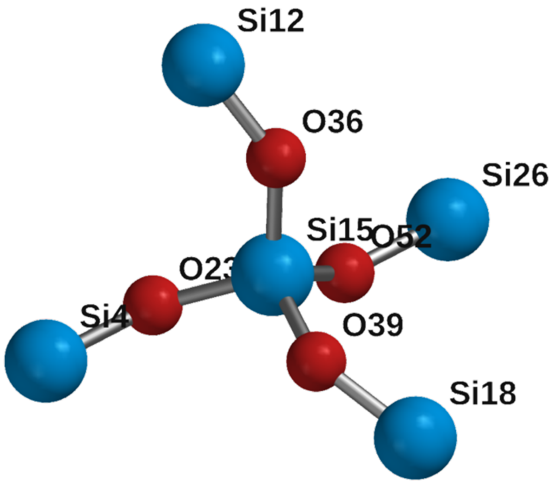

SPARTAN '18 Quantum Mechanics Program:	(x86/Darwin) build 1.4.4
SPARTAN file name:	Si5O4.spartan
Job type:	Single point.
Method:	RB3LYP
Basis set:	6-311++G**
Number of basis functions:	238
Number of electrons:	102
Parallel Job:	3 threads
SCF total energy:	**-1748.5979752** hartrees
Reason for exit:	Successful completion
Quantum Calculation CPU Time:	3:17.02
Quantum Calculation Wall Time:	1:23.23

Cartesian Coordinates (Ångstroms)

atom#	type	label	X	Y	Z
1	O	O23	-1.1283337	1.0421114	-0.6426668
2	O	O36	-0.6323335	-0.8358891	1.1503337
3	O	O52	1.141667	1.0201114	0.6543335
4	O	O39	0.6216669	-0.8298891	-1.1596671
5	Si	Si15	0.0006667	0.0951111	0.0003334
6	Si	Si4	-2.2373339	1.1291115	-1.8056672
7	Si	Si12	-2.0183339	-1.3488893	1.7833338
8	Si	Si26	2.2506673	1.0811114	1.8183338
9	Si	Si18	2.0016673	-1.3528893	-1.7986672

Mulliken bond orders in $Si(OSi)_4$

atom#1	atom#2	Bond Order	# electrons (BO x 2)
Si15	O39	0.88424	1.76849
Si16	O36	0.88309	1.76618
Si17	O23	0.79530	1.59059
Si18	O52	0.79480	1.58960
Si-O average		**0.83936**	1.67872
Si-O total		3.35743	**6.71487**
atom#1	atom#2	Bond Order	# electrons (BO x 2)
O36	O52	**0.13920**	0.27840
O23	O39	0.13917	0.27835
O52	O39	0.13368	0.26737
O23	O36	0.13333	0.26665
O36	O39	0.10746	0.21492
O23	O52	0.06936	0.13873
O-O average		**0.12037**	0.24074
O-O total		0.72221	**1.44441**
grand total bonding		4.07964	**8.15928**
percent Si-O		82.29732	**82.29732**
percent O-O		17.70268	**17.70268**
grand total non-bonding		3.92036	**7.84072**

Atomic orbital contributions for $Si(OSi)_4$. SUMSQ is the sum of the squares of the atomic orbital contributions to each valence MO. The numbers in bold appear in Figure 4 or are referenced in the text corresponding to Figure 4 (therein rounded).

average O (%)			**71.34**	
average O (%)	HOMO-13–9		**76.07**	
average Si (%)				**86.32**
valence HOMO-n	SUMSQ O	SUMSQ Si	SUMSQ O/ SUMSQ all (%)	SUMSQ Si/ SUMSQ all (%)
HOMO	0.069136181	0.573513628	10.76	89.24
HOMO-1	0.07626346	0.552109219	12.14	87.86
HOMO-2	0.131766118	0.748348593	14.97	85.03
HOMO-3	0.125855396	0.767763116	14.08	85.92
HOMO-4	0.124089582	0.828292746	13.03	86.97
HOMO-5	0.167999559	0.814547841	17.10	82.90
HOMO-6	0.513058817	0.093372846	**84.60**	15.40
HOMO-7	0.505430753	0.099336831	**83.57**	16.43
HOMO-8	0.446945181	0.144117392	**75.62**	24.38
HOMO-9	0.468623477	0.120061327	**79.61**	20.39
HOMO-10	0.406460755	0.144808638	**73.73**	26.27
HOMO-11	0.403452795	0.133129232	**75.19**	24.81
HOMO-12	0.453626172	0.111566946	**80.26**	19.74
HOMO-13	0.412754697	0.164075246	**71.56**	28.44
HOMO-14	0.280961144	0.312283672	**47.36**	52.64
HOMO-15	0.261961464	0.26397862	**49.81**	50.19
HOMO-16	0.249253288	0.261713767	**48.78**	51.22
HOMO-17	0.300082757	0.537820489	**35.81**	64.19
HOMO-18	0.636818295	0.063306698	**90.96**	9.04
HOMO-19	0.629204696	0.048117455	**92.90**	7.10
HOMO-20	0.694431711	0.090677989	**88.45**	11.55
HOMO-21	0.560855636	0.325865424	**63.25**	36.75

valence HOMO-n	SUMSQ O2*s*2*p* O#36	SUMSQ O2*s*2*p* O#52	SUMSQ Si3*s*3*p* Si#15	O2*s*2*p* (%) O#36	O2*s*2*p* (%) O#52	Si3*s*3*p* (%) Si#15
HOMO	0.018359356	0.016150399	0.047756022	2.86	2.51	7.43
HOMO-1	0.019401854	0.018653637	0.047152565	3.09	2.97	7.50
HOMO-2	0.023035603	0.042655452	0.011946652	2.62	4.85	1.36
HOMO-3	0.038172917	0.024542884	0.013077418	4.27	2.75	1.46
HOMO-4	0.051898241	0.010020417	0.014089145	5.45	1.05	1.48
HOMO-5	0.017676171	0.066194476	0.033511396	1.80	6.74	3.41
HOMO-6	0.119976059	0.135517646	0.057287426	**19.78**	**22.35**	**9.45**
HOMO-7	0.144085756	0.107858635	0.04289693	**23.82**	**17.83**	**7.09**
HOMO-8	0.085789529	0.138681963	0.053639772	**14.51**	**23.46**	**9.08**
HOMO-9	0.095370336	0.138845625	0.074572589	**16.20**	**23.59**	**12.67**
HOMO-10	0.109522755	0.093925011	0.080308	**19.87**	**17.04**	**14.57**
HOMO-11	0.068577895	0.132772925	0.044312553	**12.78**	**24.74**	**8.26**
HOMO-12	0.143174814	0.083642438	0.072006824	**25.33**	**14.80**	**12.74**
HOMO-13	0.12038825	0.08518978	0.069520647	**20.87**	**14.77**	**12.05**
HOMO-14	0.014854138	0.124983472	0.067690378	**2.50**	**21.07**	**11.41**
HOMO-15	0.116770852	0.014502105	0.060205985	**22.20**	**2.76**	**11.45**
HOMO-16	0.061573241	0.063011034	0.059779665	**12.05**	**12.33**	**11.70**
HOMO-17	0.070132825	0.079588707	0.389244795	**8.37**	**9.50**	**46.45**
HOMO-18	0.009474803	0.164612682	0.028901321	**1.35**	**23.51**	**4.13**
HOMO-19	0.12996641	0.315208852	0.01593141	**19.19**	**46.54**	**2.35**
HOMO-20	0.333790916	0.005118409	0.049972689	**42.52**	**0.65**	**6.37**
HOMO-21	0.151718648	0.121857158	0.299788728	**17.11**	**13.74**	**33.81**

Table S19. Computational results for geometry-optimized D_{6h} Si_6O_6.

Structure

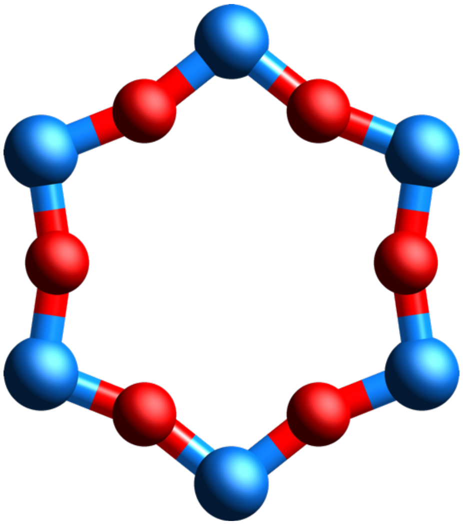

Labeled Structure

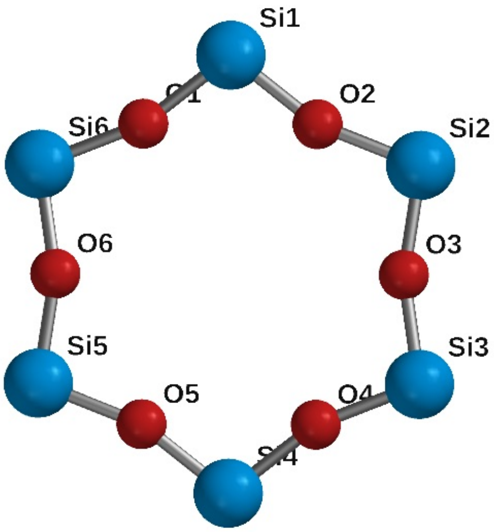

SPARTAN '18 Quantum Mechanics Program: (x86/Darwin) build 1.4.4
SPARTAN file name: Si6O6 D6h.spartan
Job type: Geometry optimization.
Method: RB3LYP
Basis set: 6-311++G**
Number of basis functions: 312
Number of electrons: 132
Parallel Job: 3 threads
Optimization:

Step	Energy	Max Grad.	Max Dist.	
1	-2188.509900	0.147008	0.283034	
2	-2188.556842	0.163590	0.296019	1
3	-2188.605829	0.173327	0.293817	1
4	-2188.658532	0.174445	0.296720	
5	-2188.710420	0.169077	0.298010	
6	-2188.759884	0.158789	0.298453	
7	-2188.805670	0.144956	0.298711	
8	-2188.846887	0.128740	0.299038	
9	-2188.882907	0.110675	0.299480	
10	-2188.913162	0.090631	0.299901	
10	-2188.913162	0.031937	0.235792	Switching to cartesian
11	-2188.932844	0.031727	0.239242	
12	-2188.962145	0.005551	0.246428	
13	-2188.961802	0.010962	0.093813	
14	-2188.964593	0.002793	0.020879	
15	-2188.964690	0.000685	0.006634	
16	**-2188.964702**	0.000006	0.000117	

Reason for exit: Successful completion
Quantum Calculation CPU Time: 39:27.26
Quantum Calculation Wall Time: 15:18.99

Cartesian Coordinates (Ångstroms)

atom#	type	label	X	Y	Z
1	O	O1	2.2668295	-1.3087546	0
2	Si	Si1	3.3044056	0	0
3	O	O2	2.2668295	1.3087546	0
4	Si	Si2	1.6522028	2.8616992	0
5	O	O3	0	2.6175093	0
6	Si	Si3	-1.6522028	2.8616992	0
7	O	O4	-2.2668295	1.3087546	0
8	Si	Si4	-3.3044056	0	0
9	O	O5	-2.2668295	-1.3087546	0
10	Si	Si5	-1.6522028	-2.8616992	0
11	O	O6	0	-2.6175093	0
12	Si	Si6	1.6522028	-2.8616992	0

Mulliken bond orders in Si_6O_6 D_{6h}

atom#1	atom#2	Bond Order
O1	Si1	0.41945
O4	Si4	0.41945
Si4	O5	0.41945
Si1	O2	0.41945
Si2	O3	0.41832
O3	Si3	0.41832
Si5	O6	0.41832
O6	Si6	0.41832
O2	Si2	0.41572
O1	Si6	0.41572
Si3	O4	0.41572
O5	Si5	0.41945
Si-O average		0.41783
Si-O total		5.01396

atom#1	atom#2	Bond Order
O2	O3	-0.05416
O3	O4	-0.05416
O1	O6	-0.05416
O5	O6	-0.05416
O1	O2	-0.05501
O4	O5	-0.05501
O-O average		**-0.05444**
O-O total		-0.32667

Atomic orbital contributions for Si_6O_6 D_{6h}. SUMSQ is the sum of the squares of the atomic orbital contributions to each valence MO. The numbers in bold appear in Figure 6a or are referenced in the text corresponding to Figure 6a (therein rounded).

average O (%) mostly 2p_y	HOMO-6,8,9,14,15,17 Figure 6a		76.49	
average O (%) mostly 2p_z	HOMO-7,10,11,12,13,16		91.11	
average Si (%)	HOMO-6,8,9,14,15,17 Figure 6a			23.51
average Si (%)	HOMO-7,10,11,12,13,16			8.89
valence HOMO-n	SUMSQ O	SUMSQ Si	SUMSQ O/ SUMSQ all (%)	SUMSQ Si/ SUMSQ all (%)
HOMO-6	0.495476196	0.043847381	**91.87**	8.13
HOMO-7	0.469846786	0.00784535	98.36	1.64
HOMO-8	0.41256569	0.118442897	**77.69**	22.31
HOMO-9	0.412247605	0.118089493	**77.73**	22.27
HOMO-10	0.413777012	0.029119562	93.43	6.57
HOMO-11	0.413778599	0.029138709	93.42	6.58
HOMO-12	0.347870348	0.048425427	87.78	12.22
HOMO-13	0.347883042	0.048418752	87.78	12.22
HOMO-14	0.338691654	0.139914456	**70.77**	29.23
HOMO-15	0.338240504	0.139818121	**70.75**	29.25
HOMO-16	0.325480489	0.053527134	85.88	14.12
HOMO-17	0.304236575	0.129623914	**70.12**	29.88

Table S20. Computational results for saddle-shaped Si_6O_6 (silica coordinates).

Structure

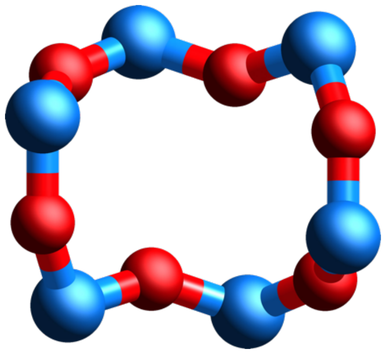

Labeled Structure

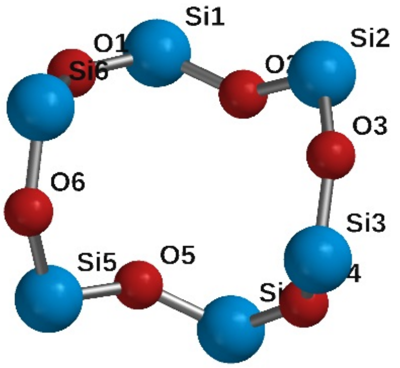

SPARTAN '18 Quantum Mechanics Program:	(x86/Darwin) build 1.4.4
SPARTAN file name:	Si6O6 silica.spartan
Job type:	Single point.
Method:	RB3LYP
Basis set:	6-311++G**
Number of basis functions:	312
Number of electrons:	132
Parallel Job:	3 threads
SCF total energy:	**-2188.9072168** hartrees
Reason for exit:	Successful completion
Quantum Calculation CPU Time:	3:58.30
Quantum Calculation Wall Time:	1:30.12

Cartesian Coordinates (Ångstroms)

atom#	type	label	X	Y	Z
1	O	O5	1.6350127	-0.3094386	-0.7830848
2	O	O4	1.1295921	2.2390502	-0.2986046
3	O	O6	1.1183898	-2.0694363	1.08154
4	O	O3	-1.1197146	2.0696032	1.0822096
5	O	O1	-1.1287322	-2.2390865	-0.298016
6	O	O2	-1.6350988	0.3103175	-0.7830815
7	Si	Si4	1.8382616	1.1987299	-1.3005081
8	Si	Si5	2.27442	-1.4576591	0.1451434
9	Si	Si3	0.457672	2.3824686	1.1548971
10	Si	Si6	-0.4573918	-2.3832015	1.1550906
11	Si	Si2	-2.274889	1.4575052	0.1456649
12	Si	Si1	-1.8375217	-1.1988527	-1.3012506

Möbius aromatic HOMO-13 for Si_6O_6 *silica* showing two half-twists (L_k = 2, IsoValue = 0.005 [e/bohr^3]$^{0.5}$)

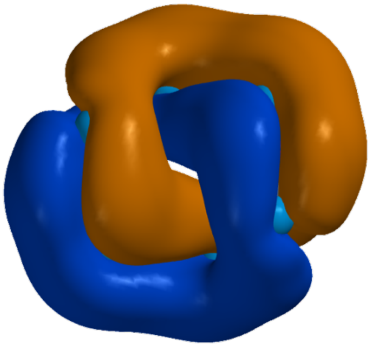

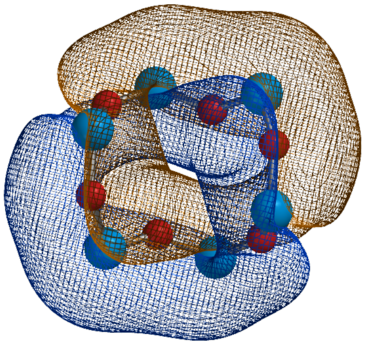

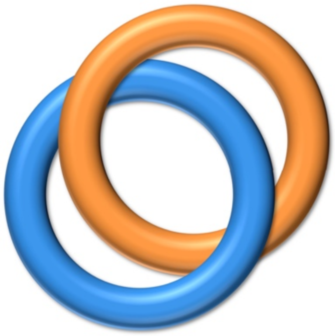

Mulliken bond orders in Si_6O_6 *silica*

atom#1	atom#2	Bond Order
O2	Si2	0.40608
O5	Si5	0.40605
O6	Si6	0.36463
O3	Si3	0.36454
O4	Si3	0.33528
O1	Si6	0.33524
O2	Si1	0.33042
O5	Si4	0.33011
O3	Si2	0.32083
O6	Si5	0.32042
O1	Si1	0.31612
O4	Si4	0.31542
Si-O average		0.34543
Si-O total		4.14513

atom#1	atom#2	Bond Order
O6	O1	**0.06795**
O4	O3	**0.06781**
O3	O2	**0.05859**
O5	O6	**0.05850**
O1	O2	**0.03599**
O5	O4	**0.03596**
O-O average		**0.05413**
O-O total		0.32479

Atomic orbital contributions for Si_6O_6 *silica*. SUMSQ is the sum of the squares of the atomic orbital contributions to each valence MO. The numbers in bold appear in Figure 6b or are referenced in the text corresponding to Figure 6b (therein rounded).

average O (%) mostly 2p_y	HOMO-6,8,11,12,13,17 Figure 6b		**84.18**	
average O (%) mostly 2p_z	HOMO-7,9,10,14,15,16		86.72	
average Si (%)	HOMO-6,8,11,12,13,17 Figure 6b			**15.82**
average Si (%)	HOMO-7,9,10,14,15,16			13.28
valence HOMO-n	SUMSQ O	SUMSQ Si	SUMSQ O/ SUMSQ all (%)	SUMSQ Si/ SUMSQ all (%)
HOMO-6	0.506926056	0.050718246	**90.90**	**9.10**
HOMO-7	0.487585967	0.028929386	94.40	5.60
HOMO-8	0.464653159	0.061379165	**88.33**	11.67
HOMO-9	0.453743542	0.064922195	87.48	12.52
HOMO-10	0.461686236	0.07708375	85.69	14.31
HOMO-11	0.425977837	0.079317359	**84.30**	15.70
HOMO-12	0.398160053	0.110281807	**78.31**	**21.69**
HOMO-13	0.374762318	0.091524835	**80.37**	19.63
HOMO-14	0.371364081	0.06861606	84.40	15.60
HOMO-15	0.388197478	0.061125663	86.40	13.60
HOMO-16	0.363748874	0.08015168	81.94	18.06
HOMO-17	0.34644759	0.071733807	**82.85**	17.15

Table S21. Computational results for $Si_{20}O_{24}$.

Structure

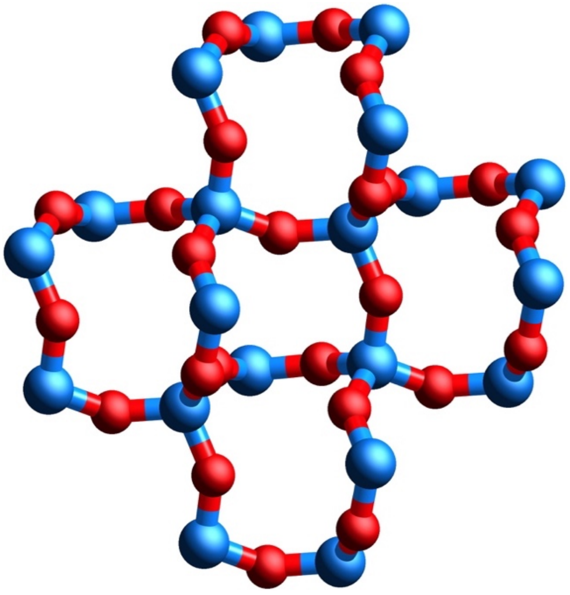

Labeled Structure

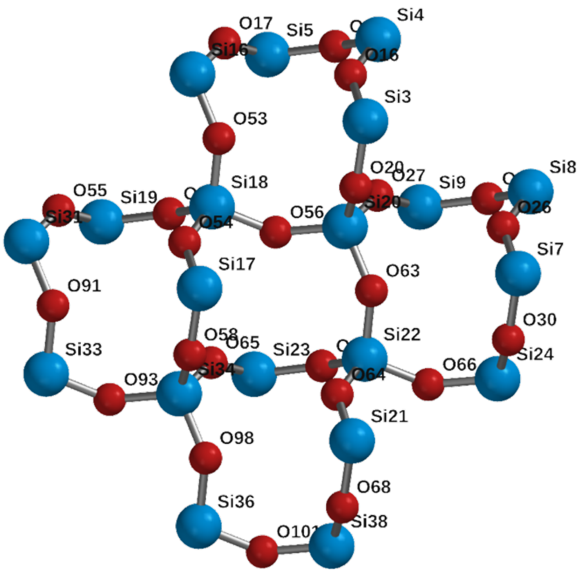

SPARTAN '18 Quantum Mechanics Program:	(x86/Darwin) build 1.4.4
SPARTAN file name:	Si20O24.spartan
Job type:	Single point.
Method:	RB3LYP
Basis set:	6-311++G**
Number of basis functions:	1128
Number of electrons:	472
Parallel Job:	3 threads
SCF total energy:	**-7597.9875621** hartrees
Reason for exit:	Successful completion

Quantum Calculation CPU Time: 7:33:40.59
Quantum Calculation Wall Time: 2:36:04.63
Cartesian Coordinates (Ångstroms)

atom#	type	label	X	Y	Z
1	O	O16	-4.2327506	0.9750228	2.2627958
2	O	O17	-2.9927504	-1.0659774	4.6987961
3	O	O19	-4.7707508	-1.5289775	2.7967959
4	O	O20	-2.4007503	1.512023	0.4317955
5	O	O26	-4.1617506	1.0710228	-2.652205
6	O	O27	-2.9227504	-0.9699774	-0.2152045
7	O	O29	-4.7007506	-1.4339775	-2.1172049
8	O	O30	-2.3307503	1.607023	-4.4822052
9	O	O53	-1.1627502	0.3100228	3.3747959
10	O	O54	1.1722502	0.9390229	2.3387958
11	O	O55	2.4112503	-1.1009774	4.7757961
12	O	O56	-0.6187501	-0.3819773	0.9107955
13	O	O57	0.6332501	-1.5649775	2.8737959
14	O	O58	3.0032504	1.4760229	0.5087956
15	O	O63	-1.0927502	0.4050228	-1.5392047
16	O	O64	1.2422502	1.0350229	-2.575205
17	O	O65	2.4822503	-1.0059774	-0.1382045
18	O	O66	-0.5477501	-0.2869773	-4.0042051
19	O	O67	0.7042501	-1.4689775	-2.0402049
20	O	O68	3.0742504	1.572023	-4.4062052
21	O	O91	4.2412507	0.2740227	3.451796
22	O	O93	4.7862507	-0.4179774	0.9877955
23	O	O98	4.3122506	0.3700228	-1.4632048
24	O	O101	4.8562507	-0.3219773	-3.9272051
25	Si	Si3	-3.5677505	2.125023	1.3617957
26	Si	Si4	-5.3987508	-0.1399773	2.2967958
27	Si	Si5	-3.6307505	-2.1789776	3.735796
28	Si	Si7	-3.4977505	2.221023	-3.5522051
29	Si	Si8	-5.3277507	-0.0439773	-2.6172049
30	Si	Si9	-3.5597505	-2.0829775	-1.1792048
31	Si	Si16	-1.8287503	0.0460228	4.8107961
32	Si	Si17	1.8362503	2.089023	1.4387957
33	Si	Si18	0.00625	-0.1759773	2.3737958
34	Si	Si19	1.7742502	-2.2149776	3.812796
35	Si	Si20	-1.7587502	0.1420228	-0.1042045
36	Si	Si21	1.9072503	2.185023	-3.4762051
37	Si	Si22	0.07625	-0.0799773	-2.5402049
38	Si	Si23	1.8442503	-2.1189776	-1.1022048
39	Si	Si24	-1.6877502	0.2380228	-5.0182053
40	Si	Si31	3.5752505	0.0110227	4.8877962
41	Si	Si33	5.4102507	-0.2109773	2.4507958
42	Si	Si34	3.6462505	0.1060228	-0.0272045
43	Si	Si36	5.4812508	-0.1159773	-2.4632048
44	Si	Si38	3.7162505	0.2020228	-4.9412053

Mulliken bond orders in $Si_{20}O_{24}$

atom#1	atom#2	Bond Order
O54	O56	**0.33497**
O56	O63	0.31039
O63	O67	0.28180
O58	O65	0.20049
O65	O67	0.13308
O54	O58	**0.12512**
O-O average		**0.23097**
O-O total		1.38585

Valence bonding vs. anti-bonding interactions for the central O_6 ring of $Si_{20}O_{24}$
O54••O56; O56••O63; O63••O67; O67••O65; O65••O58; O58••O54

**total**		**373**	**203**	for valence O 2*s* & O2*p*; ***be*$_{O-O}$ = 29.5%**
**HOMO-*n***	**energy (ev)**	**bonding**	**anti-bonding**	
HOMO{-16}	-10.22	0	6	bonding sum 2*p* only = 301
HOMO{-17}	-10.32	4	2	anti-bonding sum 2*p* only = 131
HOMO{-18}	-10.40	2	4	be_{2p} = 39.4%
HOMO{-19}	-10.47	3	3	
HOMO{-20}	-10.63	4	2	
HOMO{-21}	-10.69	5	1	
HOMO{-22}	-10.76	4	2	
HOMO{-23}	-10.80	2	4	
HOMO{-24}	-10.83	3	3	
HOMO{-25}	-10.89	3	3	
HOMO{-26}	-10.93	4	2	
HOMO{-27}	-10.97	2	4	
HOMO{-28}	-11.01	3	3	
HOMO{-29}	-11.03	4	2	
HOMO{-30}	-11.13	3	3	
HOMO{-31}	-11.22	2	4	
HOMO{-32}	-11.25	4	2	
HOMO{-33}	-11.34	5	1	
HOMO{-34}	-11.42	3	3	
HOMO{-35}	-11.45	3	3	
HOMO{-36}	-11.47	3	3	
HOMO{-37}	-11.61	5	1	
HOMO{-38}	-11.73	5	1	
HOMO{-39}	-11.78	6	0	
HOMO{-40}	-11.86	4	2	
HOMO{-41}	-11.94	6	0	
HOMO{-42}	-11.99	6	0	
HOMO{-43}	-12.04	5	1	
HOMO{-44}	-12.07	6	0	
HOMO{-45}	-12.13	2	4	
HOMO{-46}	-12.19	4	2	
HOMO{-47}	-12.21	4	2	
HOMO{-48}	-12.27	6	0	
HOMO{-49}	-12.32	5	1	
HOMO{-50}	-12.41	4	2	
HOMO{-51}	-12.44	6	0	
HOMO{-52}	-12.51	5	1	
HOMO{-53}	-12.58	5	1	
HOMO{-54}	-12.68	6	0	
HOMO{-55}	-12.72	6	0	
HOMO{-56}	-12.78	5	1	
HOMO{-57}	-12.87	3	3	

HOMO{-58}	-12.91	4	2	
HOMO{-59}	-13.00	5	1	
HOMO{-60}	-13.06	6	0	
HOMO{-61}	-13.11	5	1	
HOMO{-62}	-13.20	6	0	
HOMO{-63}	-13.23	6	0	
HOMO{-64}	-14.89	4	2	
HOMO{-65}	-14.94	5	1	
HOMO{-66}	-14.98	5	1	
HOMO{-67}	-15.04	3	3	
HOMO{-68}	-15.14	2	4	
HOMO{-69}	-15.34	3	3	
HOMO{-70}	-15.45	4	2	
HOMO{-71}	-15.58	6	0	
HOMO{-72}	-15.82	4	2	
HOMO{-73}	-15.95	4	2	
HOMO{-74}	-16.08	5	1	
HOMO{-75}	-16.19	4	2	
HOMO{-76}	-16.33	2	4	
HOMO{-77}	-16.57	4	2	
HOMO{-78}	-16.64	5	1	
HOMO{-79}	-16.92	5	1	
HOMO{-80}	-17.30	6	0	
HOMO{-81}	-17.41	4	2	
HOMO{-82}	-17.48	4	2	
HOMO{-83}	-17.59	2	4	
HOMO{-84}	-17.63	3	3	
HOMO{-85}	-18.21	5	1	
HOMO{-86}	-18.27	4	2	
HOMO{-87}	-18.78	6	0	
HOMO{-88}	-28.16	2	4	bonding sum 2*s* only = 72
HOMO{-89}	-28.17	2	4	anti-bonding sum 2*s* only = 72
HOMO{-90}	-28.19	2	4	be_{2s} = 0.0%
HOMO{-91}	-28.24	2	4	
HOMO{-92}	-28.25	0	6	
HOMO{-93}	-28.33	2	4	
HOMO{-94}	-28.38	2	4	
HOMO{-95}	-28.42	2	4	
HOMO{-96}	-28.54	2	4	
HOMO{-97}	-28.59	2	4	
HOMO{-98}	-28.60	2	4	
HOMO{-99}	-28.63	4	2	
HOMO{-100}	-28.74	2	4	
HOMO{-101}	-28.83	4	2	
HOMO{-102}	-28.89	4	2	
HOMO{-103}	-28.98	4	2	
HOMO{-104}	-29.14	6	0	
HOMO{-105}	-29.17	4	2	
HOMO{-106}	-29.23	4	2	
HOMO{-107}	-29.28	4	2	
HOMO{-108}	-29.48	2	4	
HOMO{-109}	-29.79	4	2	
HOMO{-110}	-29.80	4	2	
HOMO{-111}	-30.12	6	0	

Table S22. Computational results for $Si_6O_{18}H_{12}$.

Structure

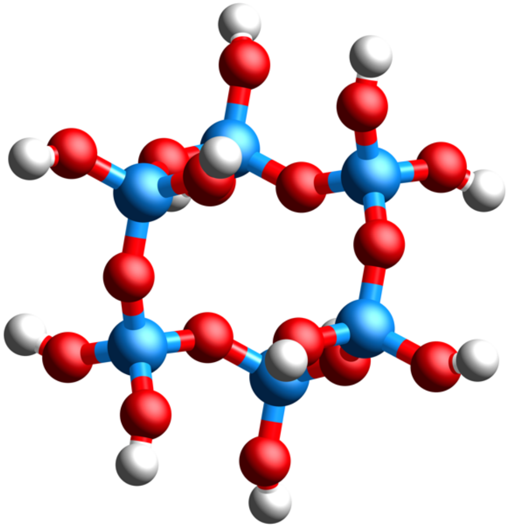

Labeled Structure

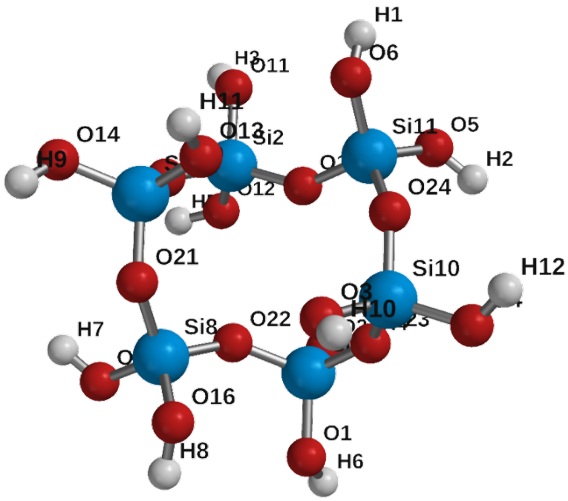

SPARTAN '18 Quantum Mechanics Program: (x86/Darwin) build 1.4.4
SPARTAN file name: Si6O18H12.spartan
Job type: Single point.
Method: RB3LYP
Basis set: 6-311++G**
Number of basis functions: 660
Number of electrons: 240
Parallel Job: 3 threads
SCF total energy: **-3100.0343896** hartrees
Reason for exit: Successful completion
Quantum Calculation CPU Time: 56:20.10
Quantum Calculation Wall Time: 21:06.49
Cartesian Coordinates (Ångstroms)

atom#	type	label	X	Y	Z
1	O	O22	1.6349975	-0.3093092	-0.7832243
2	O	O23	1.1295771	2.2391786	-0.2987443
3	O	O2	1.1663688	1.342132	-2.7536982
4	O	O1	3.4148704	1.5120859	-1.3741901
5	O	O16	3.429884	-0.846811	1.0816113
6	O	O15	2.9145231	-2.6060451	-0.7826807
7	O	O21	1.1183747	-2.0693064	1.0813997
8	O	O24	-1.1197289	2.0697317	1.0820694
9	O	O4	0.6600798	3.8917666	1.6729259
10	O	O3	1.1664464	1.3423635	2.1579912
11	O	O14	-0.6606321	-3.8911882	1.6733719
12	O	O13	-1.1660756	-1.3427514	2.1568536
13	O	O20	-1.1287464	-2.2389566	-0.2981557
14	O	O5	-2.9143331	2.6058031	-0.7837013
15	O	O19	-1.6351129	0.3104466	-0.783221
16	O	O6	-3.4309559	0.845806	1.0809228
17	O	O12	-1.1661532	-1.3429829	-2.7548358
18	O	O11	-3.4140787	-1.5121263	-1.3740697
19	Si	Si9	1.8382463	1.1988586	-1.3006475
20	Si	Si8	2.2744045	-1.4575294	0.1450035
21	Si	Si10	0.4576572	2.382597	1.1547569
22	Si	Si7	-0.4574064	-2.3830715	1.1549504

23	Si	Si11	-2.2749029	1.4576339	0.145525
24	Si	Si2	-1.8375357	-1.198723	-1.30139
25	H	H6	3.7864443	1.9184434	-2.1581358
26	H	H4	0.8209733	2.1884659	-3.0403776
27	H	H12	0.0224583	4.2821251	2.2719446
28	H	H2	-2.4806906	3.4565734	-0.860568
29	H	H1	-4.336298	0.8202274	0.7687279
30	H	H3	-3.7861961	-1.9176009	-2.1582146
31	H	H5	-0.8197826	-2.1892218	-3.0406182
32	H	H11	-1.4657295	-1.6357687	3.0183133
33	H	H9	-0.0223407	-4.2823098	2.2711783
34	H	H7	2.4805633	-3.4566169	-0.8599536
35	H	H8	4.3351883	-0.8197685	0.76943
36	H	H10	1.4656417	1.6358491	3.0194508

Mulliken bond orders in $Si_6O_{18}H_{12}$

atom#1	atom#2	Bond Order	O-O distance (Å)
O19	O20	0.246472	2.644
O20	O21	0.214802	2.642
O21	O22	0.212064	2.616
O22	O23	0.247615	2.643
O23	O24	0.213345	2.645
O24	O19	0.211827	2.615
O-O average		**0.224354**	2.634

Table S23. Computational results for $Si_{28}O_{76}H_{40}$.

Structure

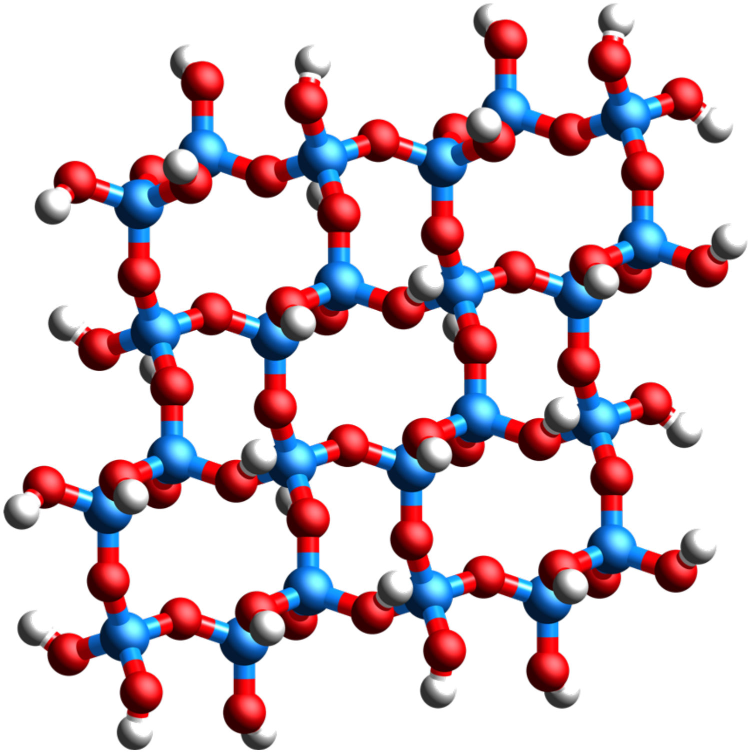

Labeled Structure

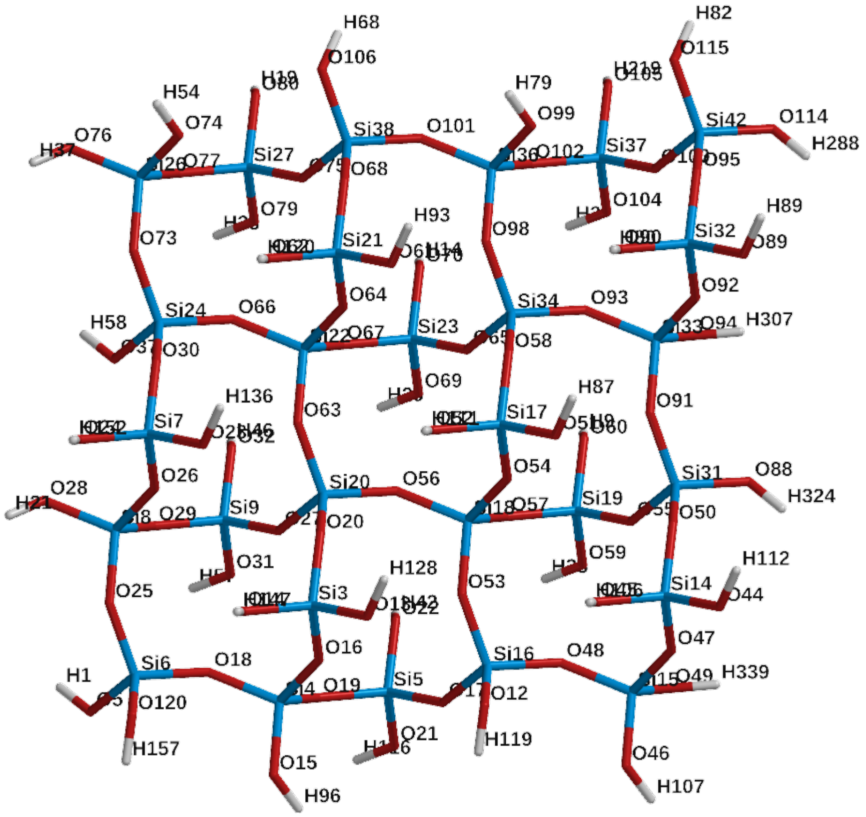

SPARTAN '18 Quantum Mechanics Program: (x86/Darwin) build 1.4.4
SPARTAN file name: Si28O76H40.spartan
Job type: Single point.
Method: RB3LYP
Basis set: 6-311++G**
Number of basis functions: 2792
Number of electrons: 1040
Parallel Job: 4 threads
SCF total energy: **-13855.2509440** hartrees
Reason for exit: Successful completion
Quantum Calculation CPU Time: 133:14:08.36
Quantum Calculation Wall Time: 33:36:46.62
Cartesian Coordinates (Ångstroms)

atom#	type	label	X	Y	Z
1	O	O120	-8.5221264	1.552946	0.3361049
2	O	O5	-9.0431265	-0.9290541	-0.3108951
3	O	O12	-3.188126	1.4209461	5.3271053
4	O	O13	-3.6471261	3.2439462	2.3061051
5	O	O14	-5.4251262	2.7799462	0.405105
6	O	O15	-7.2841264	0.350946	3.2791051
7	O	O16	-4.9491262	0.979946	2.2441051
8	O	O17	-3.7091261	-1.0610541	4.6801052
9	O	O18	-6.7391263	-0.341054	0.8151049
10	O	O19	-5.4871262	-1.5240541	2.7781051
11	O	O20	-3.117126	1.5169461	0.413105
12	O	O21	-5.0111262	-3.3240543	4.6171052
13	O	O22	-3.180126	-2.7880543	2.7871051
14	O	O23	-3.5761261	3.3399462	-2.6078953
15	O	O24	-5.3541262	2.8759462	-4.5098954

16	O	O25	-7.2131263	0.445946	-1.6348952
17	O	O26	-4.8781262	1.075946	-2.6708953
18	O	O27	-3.6391261	-0.9650541	-0.2338951
19	O	O28	-6.6691263	-0.246054	-4.0998954
20	O	O29	-5.4171262	-1.4290542	-2.1358953
21	O	O30	-3.047126	1.6119461	-4.5008954
22	O	O31	-4.9411262	-3.2280542	-0.2968951
23	O	O32	-3.1091261	-2.6920543	-2.1278952
24	O	O37	-3.5681261	-0.8690541	-5.1488955
25	O	O44	1.6868743	3.1119461	7.2981054
26	O	O45	-0.0911258	2.6479462	5.3961053
27	O	O46	-1.950126	0.218946	8.2701055
28	O	O47	0.3848742	0.848946	7.2351055
29	O	O48	-1.4051259	-0.4730541	5.8061053
30	O	O49	-0.1531258	-1.6560542	7.7701055
31	O	O50	2.2168744	1.3849461	5.4041053
32	O	O51	1.7578743	3.2079461	2.3831051
33	O	O52	-0.0201258	2.7439462	0.481105
34	O	O53	-1.879126	0.314946	3.3561052
35	O	O54	0.4558742	0.943946	2.3201051
36	O	O55	1.6948743	-1.0960541	4.7571052
37	O	O56	-1.3351259	-0.3770541	0.8921049
38	O	O57	-0.0831258	-1.5600542	2.8551051
39	O	O58	2.2868744	1.4809461	0.490105
40	O	O59	0.3928743	-3.3600543	4.6941052
41	O	O60	2.2248744	-2.8240542	2.8641051
42	O	O61	1.8278743	3.3039462	-2.5308952
43	O	O62	0.0498742	2.8399462	-4.4328954
44	O	O63	-1.8091259	0.409946	-1.5578952
45	O	O64	0.5258742	1.039946	-2.5938953
46	O	O65	1.7658743	-1.0010541	-0.1568951
47	O	O66	-1.2641259	-0.2820541	-4.0228954
48	O	O67	-0.0121258	-1.4640542	-2.0588953
49	O	O68	2.3578743	1.5769461	-4.4248954
50	O	O69	0.4638742	-3.2640543	-0.2198951
51	O	O70	2.2958743	-2.7280542	-2.0508952
52	O	O73	-1.7381259	0.505946	-6.4728956
53	O	O74	0.5968743	1.1359461	-7.5078956
54	O	O75	1.8358744	-0.9050541	-5.0718955
55	O	O76	-1.1931259	-0.1860541	-8.9368957
56	O	O77	0.0578742	-1.3690541	-6.9738956
57	O	O79	0.5348742	-3.1690543	-5.1348955
58	O	O80	2.3658743	-2.6320542	-6.9648956
59	O	O88	3.9988745	-0.5090541	5.8831053
60	O	O89	7.1618747	3.1719462	2.4601051
61	O	O90	5.3838746	2.7079462	0.558105
62	O	O91	3.5248745	0.2789459	3.4331052
63	O	O92	5.8598746	0.907946	2.3971051
64	O	O93	4.0698745	-0.4130541	0.9691049
65	O	O94	5.3218746	-1.5960541	2.9321051
66	O	O95	7.6918747	1.444946	0.567105
67	O	O98	3.5958745	0.374946	-1.4818952
68	O	O99	5.9308746	1.003946	-2.5168953
69	O	O100	7.1698747	-1.0370541	-0.0798951
70	O	O101	4.1398745	-0.3170541	-3.9458954
71	O	O102	5.3918746	-1.5000541	-1.9818953
72	O	O104	5.8678746	-3.3000543	-0.1428951

73	O	O105	7.6998748	-2.7640543	-1.9738952
74	O	O106	3.6658744	0.469946	-6.3958956
75	O	O114	9.4738749	-0.4490541	1.045105
76	O	O115	8.9998748	0.338946	-1.4048952
77	Si	Si3	-4.2841261	2.1299461	1.343105
78	Si	Si4	-6.1151263	-0.135054	2.2781051
79	Si	Si5	-4.3471261	-2.1740542	3.7171052
80	Si	Si6	-7.8791264	0.1829459	-0.1988951
81	Si	Si7	-4.2141261	2.2259461	-3.5708953
82	Si	Si8	-6.0441262	-0.039054	-2.6358953
83	Si	Si9	-4.2761261	-2.0780542	-1.1978952
84	Si	Si14	1.0488742	1.9989461	6.3341054
85	Si	Si15	-0.7811259	-0.266054	7.2701054
86	Si	Si16	-2.545126	0.050946	4.7921053
87	Si	Si17	1.1198743	2.0939461	1.420105
88	Si	Si18	-0.7101259	-0.1710541	2.3551051
89	Si	Si19	1.0578742	-2.2100542	3.7941052
90	Si	Si20	-2.475126	0.146946	-0.1228951
91	Si	Si21	1.1908743	2.1899461	-3.4948954
92	Si	Si22	-0.6401259	-0.0750541	-2.5588952
93	Si	Si23	1.1278743	-2.1140542	-1.1208952
94	Si	Si24	-2.404126	0.242946	-5.0368954
95	Si	Si26	-0.5691258	0.0209459	-7.4738956
96	Si	Si27	1.1988743	-2.0190542	-6.0348955
97	Si	Si31	2.8588744	0.015946	4.8691053
98	Si	Si32	6.5248747	2.0579461	1.497105
99	Si	Si33	4.6938745	-0.2060541	2.4321051
100	Si	Si34	2.9298744	0.110946	-0.0458951
101	Si	Si36	4.7648745	-0.111054	-2.4818952
102	Si	Si37	6.5328746	-2.1500542	-1.0438952
103	Si	Si38	2.9998744	0.206946	-4.9598954
104	Si	Si42	8.3338747	0.0749459	0.0311049
105	H	H107	-2.0597608	-0.2070166	9.1211638
106	H	H112	2.4393171	3.6264856	7.003414
107	H	H96	-7.3944043	-0.0756773	4.1297493
108	H	H116	-5.7549542	-3.8256708	4.2811517
109	H	H288	9.5589304	-0.0560366	1.9146261
110	H	H307	6.0578687	-1.5960784	3.5453561
111	H	H324	4.0844047	-0.1160923	6.752605
112	H	H339	0.583296	-1.6560699	8.3828427
113	H	H89	7.9149471	3.6857154	2.1656781
114	H	H119	-3.9415505	1.3976669	5.918353
115	H	H157	-9.2755509	1.5296669	0.9273526
116	H	H219	7.7894874	-3.7134192	-2.0657645
117	H	H2	5.124307	-3.8019722	-0.4789752
118	H	H9	2.3140621	-3.7734066	2.7716945
119	H	H14	2.3854765	-3.6773902	-2.1430728
120	H	H19	2.4558386	-3.5813439	-7.0571981
121	H	H25	-0.3507806	-3.8616366	4.3577174
122	H	H29	-0.2798663	-3.7660064	-0.5555412
123	H	H33	-0.2093209	-3.6702796	-5.4706199
124	H	H42	-3.0906048	-3.7374165	2.6951195
125	H	H46	-3.0195134	-3.6414191	-2.2197645
126	H	H57	-5.6846937	-3.7299721	-0.6329752
127	H	H80	5.2963791	3.6601758	0.5000767
128	H	H87	2.5104561	3.722058	2.0880224
129	H	H93	2.5806459	3.8182572	-2.8251458

130	H	H106	-0.1787421	3.6001541	5.337902
131	H	H111	-0.1080074	3.6961852	0.4238193
132	H	H120	-0.0373024	3.7921967	-4.491062
133	H	H128	-2.8940537	3.7577154	2.0116781
134	H	H136	-2.8232113	3.8536888	-2.9027715
135	H	H147	-5.5123029	3.7321966	0.3469383
136	H	H152	-5.442015	3.8281851	-4.5671725
137	H	H1	-9.1297342	-1.4532071	-1.1080946
138	H	H21	-7.4062758	0.2943628	-4.3868176
139	H	H37	-1.930701	0.3537768	-9.2238275
140	H	H54	0.7066708	1.690777	-8.2811171
141	H	H58	-3.6547338	-1.3932071	-5.9460949
142	H	H68	4.4116077	-0.0589288	-6.6821428
143	H	H79	6.040152	1.55863	-3.2902957
144	H	H82	9.7453173	-0.1900267	-1.6917184

Mulliken bond orders in $Si_{28}O_{76}H_{40}$

atom#1	atom#2	Bond Order	O-O distance (Å)
O67	O65	0.32504	2.644
O65	O58	0.39469	2.617
O58	O54	0.29250	2.644
O54	O56	0.42648	2.644
O56	O63	0.50669	2.617
O63	O67	0.52110	2.644
O-O average		**0.41108**	2.635

Table S24. Computational results for Al_8O_{12}.

Structure

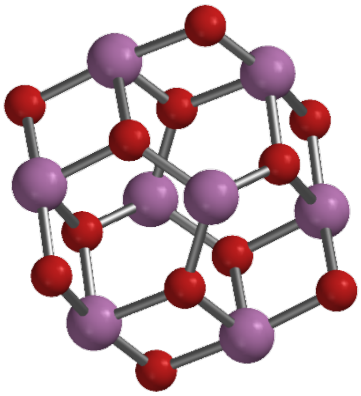

Labeled Structure

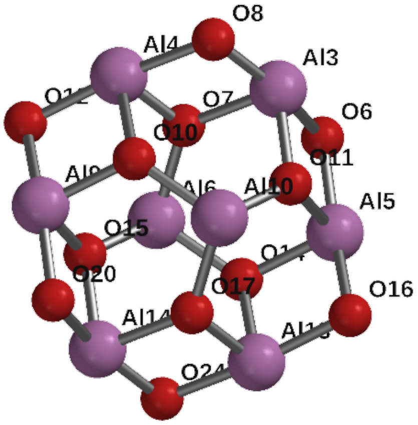

SPARTAN '18 Quantum Mechanics Program:	(x86/Darwin) build 1.4.4
SPARTAN file name:	Al8.spartan
Job type:	Single point.
Method:	RB3LYP
Basis set:	6-311++G**
Number of basis functions:	504
Number of electrons:	200
SCF total energy:	**-2843.5526649** hartrees
Reason for exit:	Successful completion
Quantum Calculation CPU Time:	25:25.46
Quantum Calculation Wall Time:	30:30.23

Cartesian Coordinates (Ångstroms)

atom#	type	label	X	Y	Z
1	Al	Al5	-0.4922	2.1494501	1.6537501
2	Al	Al13	0.4048001	2.5534501	-0.9562501
3	Al	Al14	0.1708	0.4074501	-2.7212502
4	Al	Al3	-0.1702	-0.40755	2.7207502
5	Al	Al10	1.8968002	-0.00755	0.29075
6	Al	Al4	-0.4042	-2.5535502	0.95575
7	Al	Al9	0.4918	-2.1495502	-1.6542501
8	Al	Al6	-1.8972001	0.00745	-0.29025
9	O	O24	-0.7042	2.0254502	-2.4962502
10	O	O11	0.8468001	0.70345	1.6407501
11	O	O14	-1.0832001	1.6524501	-0.03025
12	O	O16	0.9958	3.0504502	0.72875
13	O	O20	1.5088001	-1.03855	-2.7342502
14	O	O17	1.2788001	0.9354501	-1.1802501
15	O	O7	-1.2792001	-0.93555	1.1807501
16	O	O6	-1.5082001	1.0384501	2.7337502
17	O	O12	-0.9962	-3.0505502	-0.7282501
18	O	O8	0.7038001	-2.0255502	2.4967502
19	O	O15	-0.8462	-0.70355	-1.6412501
20	O	O10	1.0828001	-1.6515501	0.03075

Maximum Mulliken O-O bond orders in Al_8O_{12}

atom#1	atom#2	Bond Order
O11	O14	0.13439
O15	O10	0.13426
O17	O15	0.13360
O11	O7	0.13346
O14	O17	0.13343
O7	O10	0.13326

Table S25. Computational results for $Al_{12}O_{18}$.

Structure

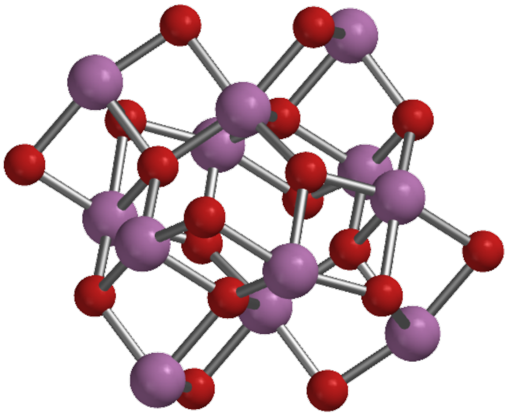

Labeled Structure

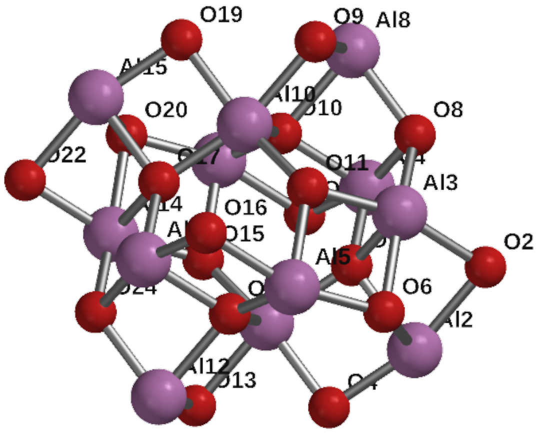

SPARTAN '18 Quantum Mechanics Program:	(x86/Darwin)	build 1.4.4
SPARTAN file name:	Al12.spartan	
Job type:	Single point.	
Method:	RB3LYP	
Basis set:	6-311++G**	
Number of basis functions:	756	
Number of electrons:	300	

SCF total energy:	**-4265.2433661** hartrees
Reason for exit:	Successful completion
Quantum Calculation CPU Time:	3:01:24.47
Quantum Calculation Wall Time:	3:12:00.95

Cartesian Coordinates (Ångstroms)

atom#	type	label	X	Y	Z
1	Al	Al12	-2.2181335	2.5644335	-1.3572668
2	Al	Al5	-0.4921334	2.1494335	1.6537335
3	Al	Al13	0.4048667	2.5534335	-0.9562667
4	Al	Al14	0.1708667	0.4074334	-2.7212669
5	Al	Al15	2.7928669	0.3964334	-2.3192668
6	Al	Al2	-2.7931335	-0.3965667	2.3197335
7	Al	Al3	-0.1701333	-0.4075667	2.7207336
8	Al	Al10	1.8968668	-0.0075667	0.2907333
9	Al	Al4	-0.4041334	-2.5535668	0.9557334
10	Al	Al9	0.4918667	-2.1495668	-1.6542668
11	Al	Al6	-1.8971335	0.0074334	-0.2902667
12	Al	Al8	2.2178668	-2.5645668	1.3577335
13	O	O24	-0.7041334	2.0254335	-2.4962668
14	O	O11	0.8468667	0.7034334	1.6407334
15	O	O14	-1.0831334	1.6524334	-0.0302667
16	O	O16	0.9958667	3.0504335	0.7287334
17	O	O20	1.5088668	-1.0385667	-2.7342669
18	O	O17	1.2788668	0.9354334	-1.1802668
19	O	O13	-3.0321336	0.9204334	-1.6172668
20	O	O22	1.6578668	1.3084335	-3.6462669
21	O	O7	-1.2791334	-0.9355667	1.1807335
22	O	O2	-1.6581335	-1.3085668	3.6457336
23	O	O4	-3.4111336	0.5464334	0.8487334
24	O	O6	-1.5081335	1.0384334	2.7337335
25	O	O19	3.4108669	-0.5465667	-0.8482667
26	O	O9	3.0318669	-0.9195667	1.6167334
27	O	O12	-0.9961334	-3.0505669	-0.7282667
28	O	O8	0.7038667	-2.0255668	2.4967335
29	O	O15	-0.8461334	-0.7035667	-1.6412668
30	O	O10	1.0828667	-1.6515668	0.0307333

Maximum Mulliken O-O bond orders in $Al_{12}O_{18}$

atom#1	atom#2	Bond Order
O15	O10	0.19783
O11	O14	0.19779
O19	O10	0.18271
O14	O4	0.18255

Table S26. Computational results for $Al_{16}O_{24}$.

Structure

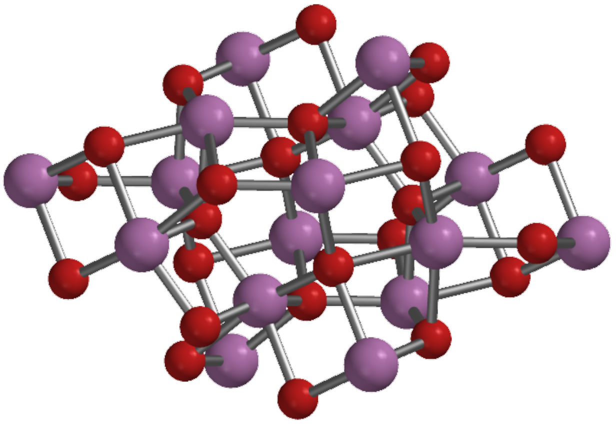

Labeled Structure

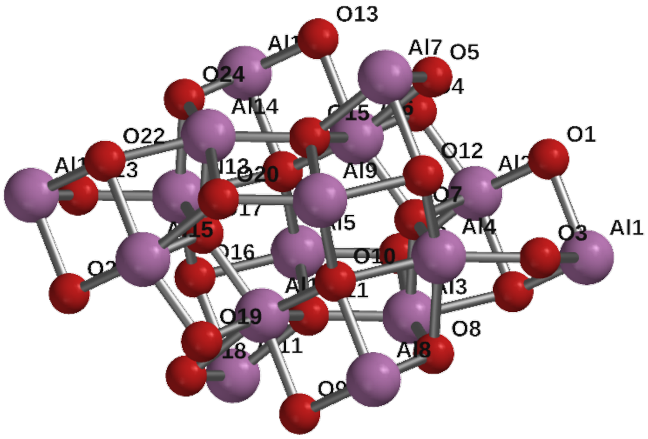

SPARTAN '18 Quantum Mechanics Program:	(x86/Darwin) build 1.4.4
SPARTAN file name:	Al16.spartan
Job type:	Single point.
Method:	RB3LYP
Basis set:	6-311++G**
Number of basis functions:	1008
Number of electrons:	400
Parallel Job:	3 threads
SCF total energy:	**-5686.8363238** hartrees
Reason for exit:	Successful completion
Quantum Calculation CPU Time:	19:54:45.35
Quantum Calculation Wall Time:	7:57:00.52

Cartesian Coordinates (Ångstroms)

atom#	type	label	X	Y	Z
1	Al	Al12	-2.2181002	2.5644001	-1.3572751
2	Al	Al5	-0.4921001	2.1494001	1.6537251
3	Al	Al13	0.4049001	2.5534001	-0.956275
4	Al	Al11	2.1309002	2.1384002	2.0557252
5	Al	Al16	2.4719002	2.9534002	-3.3862753
6	Al	Al14	0.1709	0.4074001	-2.7212752
7	Al	Al15	2.7929002	0.3964001	-2.3192751
8	Al	Al2	-2.7931002	-0.3966	2.3197251
9	Al	Al3	-0.1701	-0.4076	2.7207252
10	Al	Al10	1.8969001	-0.0076	0.290725
11	Al	Al7	-2.1311002	-2.1386002	-2.0552752
12	Al	Al4	-0.4041001	-2.5536002	0.9557251
13	Al	Al9	0.4919	-2.1496002	-1.6542751
14	Al	Al6	-1.8971002	0.0074	-0.290275
15	Al	Al8	2.2179002	-2.5646002	1.3577251
16	Al	Al1	-2.4721002	-2.9526002	3.3867252
17	O	O23	1.4209001	3.6644003	-2.0362752
18	O	O24	-0.7041001	2.0254001	-2.4962751
19	O	O11	0.8469	0.7034001	1.6407251
20	O	O14	-1.0831	1.6524001	-0.030275
21	O	O18	3.1819002	1.4274001	0.704725
22	O	O16	0.9959	3.0504002	0.7287251
23	O	O20	1.5089001	-1.0386	-2.7342752
24	O	O21	3.6069003	2.0404002	-2.0592752
25	O	O17	1.2789001	0.9354001	-1.1802751

26	O	O13	-3.0321002	0.9204001	-1.6172751
27	O	O22	1.6579001	1.3084001	-3.6462752
28	O	O7	-1.2791001	-0.9356	1.1807251
29	O	O2	-1.6581001	-1.3086001	3.6457253
30	O	O4	-3.4111003	0.5464001	0.8487251
31	O	O6	-1.5081001	1.0384001	2.7337252
32	O	O19	3.4109003	-0.5466	-0.848275
33	O	O5	-3.1821002	-1.4266001	-0.7052751
34	O	O9	3.0319002	-0.9196	1.6167251
35	O	O12	-0.9961001	-3.0506002	-0.7282751
36	O	O8	0.7039	-2.0256002	2.4967251
37	O	O15	-0.8461001	-0.7036	-1.6412751
38	O	O1	-3.6071003	-2.0406002	2.0597252
39	O	O10	1.0829001	-1.6516001	0.030725
40	O	O3	-1.4211001	-3.6646003	2.0357251

Maximum Mulliken O-O bond orders in $Al_{12}O_{18}$

atom#1	atom#2	Bond Order
O11	O7	0.31564
O17	O15	0.31550

Table S27. Computational results for α-sulfur S_8.

Structure

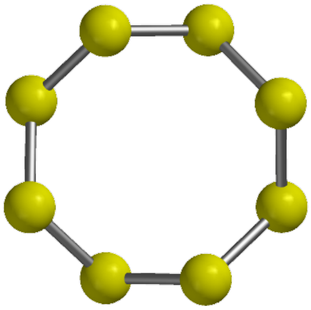

Labeled Structure

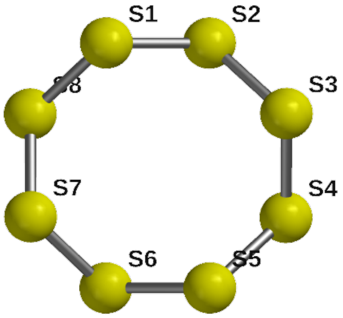

Rotated Structure

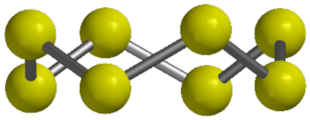

Rotated Labeled Structure

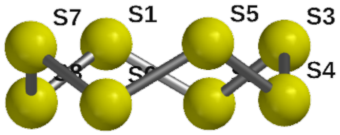

SPARTAN '18 Quantum Mechanics Program:	(x86/Darwin) build 1.4.4
SPARTAN file name:	S8 refined.spartan
Job type:	Single point.
Method:	RB3LYP
Basis set:	6-311++G**
Number of basis functions:	240
Number of electrons:	128
Parallel Job:	3 threads
SCF total energy:	**-3185.7523781** hartrees
Reason for exit:	Successful completion
Quantum Calculation CPU Time:	1:41.27
Quantum Calculation Wall Time:	49.66

Cartesian Coordinates (Ångstroms)

atom#	type	label	X	Y	Z
1	S	S8	-1.6628751	-0.338875	-1.7013751
2	S	S7	-2.3298751	0.590125	-0.013375
3	S	S1	0.062125	0.585125	-2.3103752
4	S	S6	-1.6888751	-0.482875	1.6116251
5	S	S2	1.6301251	-0.5488751	-1.6453751
6	S	S5	-0.017875	0.447125	2.3386251
7	S	S3	2.3871252	0.341125	0.035625
8	S	S4	1.6201251	-0.5928751	1.6846251

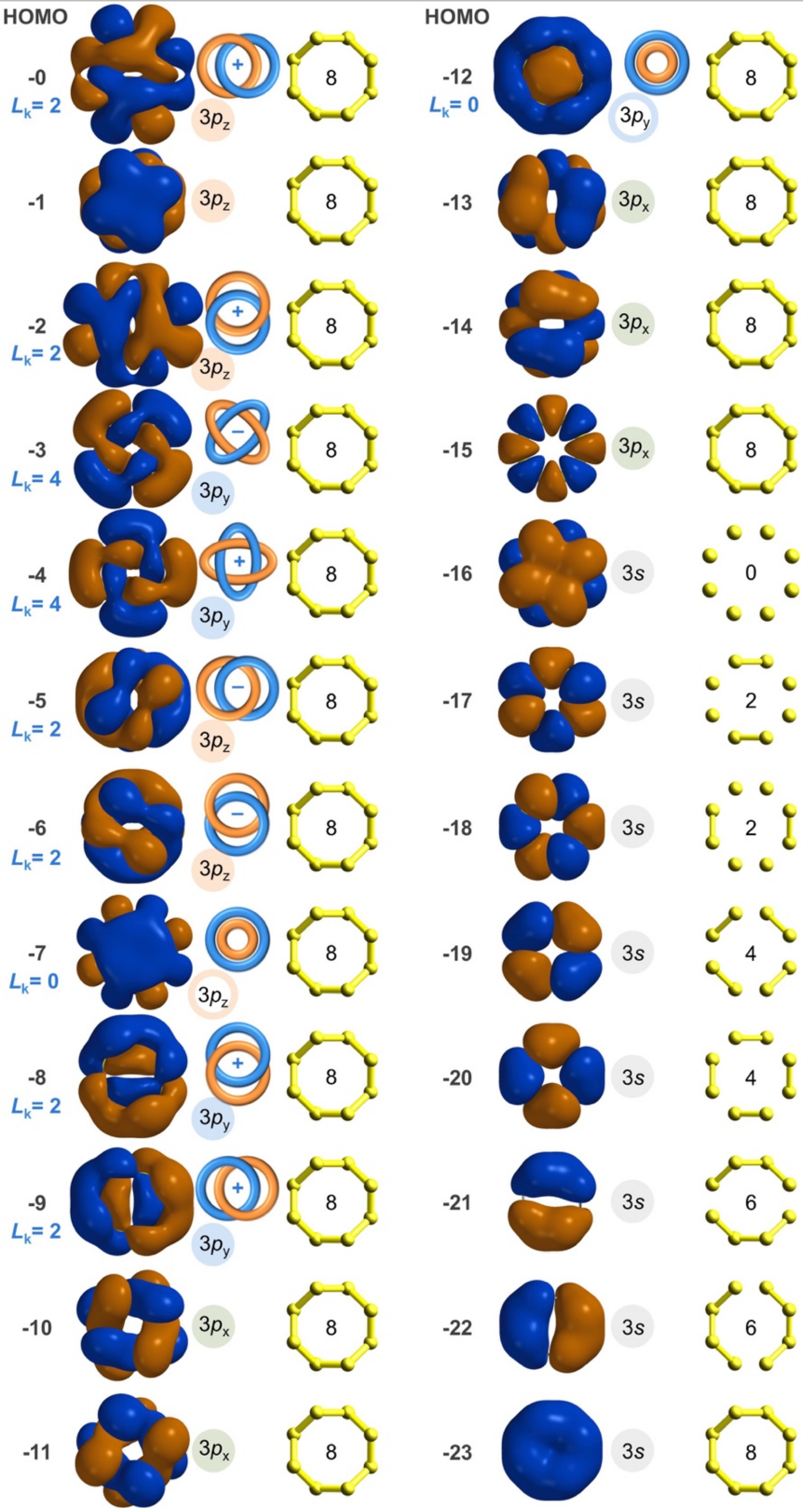

Figure S5. Valence molecular orbitals HOMO-23 through HOMO for α-sulfur S_8. MOs with contiguous *p* orbitals are shown with both Hückel (L_k = 0) and Möbius (L_k = 2 or 4) aromaticity. These 24 MOs are built primarily from sulfur 3*s*, 3p_x, 3p_y, or 3p_z atomic orbitals, as designated. MO surfaces are illustrated with IsoValue = 0.005 [e/bohr^3]$^{0.5}$, except for HOMO-2 and HOMO, which are illustrated with IsoValue = 0.003 [e/bohr^3]$^{0.5}$. S-S bonding interactions per MO provide the *bonding excess* parameters be_{3s} = 0%, be_{3p} = 100%, and collectively, *be* = 67%, as described in the main text.

Mulliken bond orders in S_8

atom#1	atom#2	Bond Order	S-S distance (Å)
S1	S2	1.18097	2.046
S2	S3	1.15178	2.047
S3	S4	1.13199	2.044
S4	S5	1.15150	2.048
S5	S6	1.18087	2.046
S6	S7	1.14876	2.050
S7	S8	1.14790	2.039
S8	S1	1.14881	2.049

Table S28. Computational results for HOON.

Structure

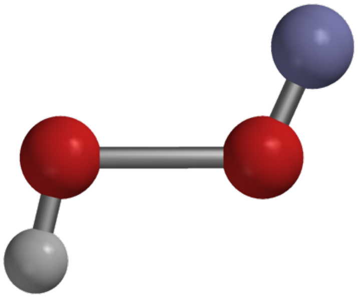

Labeled Structure

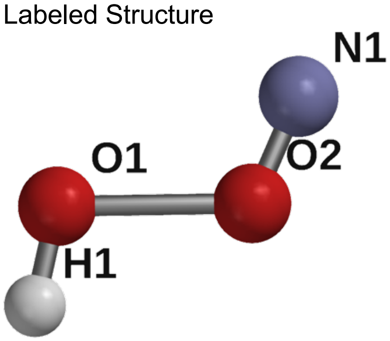

SPARTAN '18 Quantum Mechanics Program:	(x86/Darwin)	build 1.4.4
SPARTAN file name:	HOON.spartan	
Job type:	Geometry optimization.	
Method:	RB3LYP	
Basis set:	6-311++G**	
Number of basis functions:	73	
Number of electrons:	24	
Parallel Job:	3 threads	

Optimization:

Step	Energy	Max Grad.	Max Dist.
1	-205.594943	0.112912	0.214161
2	-205.639360	0.095844	0.203056
3	-205.671155	0.067228	0.090000
4	-205.678460	0.057573	0.264970
5	-205.692146	0.026844	0.167356
6	-205.695896	0.012863	0.086280
7	-205.696849	0.006841	0.043437
8	-205.697138	0.004206	0.045948
9	-205.697306	0.002553	0.035451
10	-205.697388	0.001367	0.014837
11	**-205.697403**	0.000524	0.004182

Reason for exit:	Successful completion
Quantum Calculation CPU Time:	38.16
Quantum Calculation Wall Time:	18.81

Cartesian Coordinates (Ångstroms)

atom#	type	label	X	Y	Z
1	O	O1	-0.4844265	0	-0.8753083
2	O	O2	0.4550681	0	0.7562842
3	N	N1	-0.1886529	0	1.661619
4	H	H1	0.2180112	0	-1.542595

Mulliken bond order in HOON

atom#1	atom#2	Bond Order	O-O distance (Å)
O1	O2	**0.405707**	1.883

Table S29. Computational results for ozone (O_3).

Structure

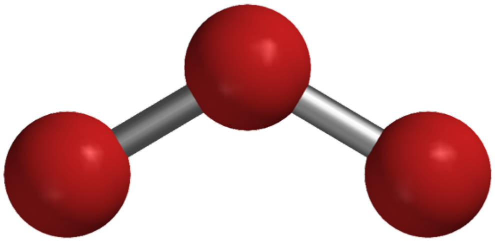

Labeled Structure

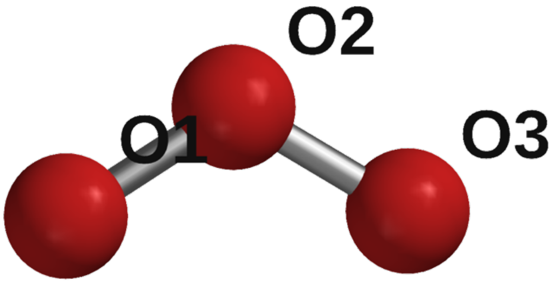

SPARTAN '18 Quantum Mechanics Program: (x86/Darwin) build 1.4.4
SPARTAN file name: ozone.spartan
Job type: Geometry optimization.
Method: RB3LYP
Basis set: 6-311++G**
Number of basis functions: 66
Number of electrons: 24
Parallel Job: 3 threads
Optimization:

Step	Energy	Max Grad.	Max Dist.
1	-225.423051	0.162705	0.296035
2	-225.463155	0.105451	0.299470
3	-225.478919	0.044237	0.053907
4	-225.480491	0.006434	0.014536
5	-225.480570	0.000767	0.001461
6	**-225.480571**	0.000026	0.000038

Reason for exit: Successful completion
Quantum Calculation CPU Time: 14.11
Quantum Calculation Wall Time: 6.96
Cartesian Coordinates (Ångstroms)

atom#	type	label	X	Y	Z
1	O	O1	1.0793009	0	0.2142479
2	O	O2	0.0000001	0	-0.4284959
3	O	O3	-1.079301	0	0.2142479

Mulliken bond orders in ozone

atom#1	atom#2	Bond Order	O-O distance (Å)
O1	O2	**1.442438**	**1.256**
O2	O3	1.442438	1.256
O1	O3	**0.590359**	**2.159**

Table S30. For various computational methods and basis sets, this table provides average computed Mulliken bond orders for the central SiO_4 unit within the *Si5* ($Si_5O_{16}H_{12}$), *Si11* ($Si_{11}O_{32}H_{20}$), and *Si21* ($Si_{21}O_{56}H_{28}$), clusters. Four bond order values are averaged for Si-O and six bond order values are averaged for O-O. (Not all computations converged.).

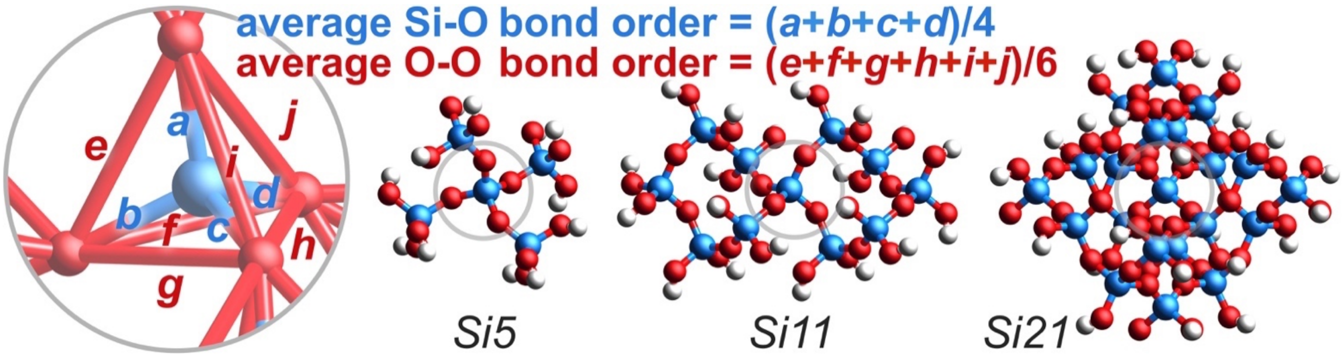

method	basis set	Si5		file	Si11		file	Si21		file
		Si-O	O-O		Si-O	O-O		Si-O	O-O	
B3LYP	6-311++G**	0.74	0.28	Si5 A.spartan	0.80	0.36	Si11 A.spartan	0.68	0.46	Si21 A.spartan
B3LYP-D3	6-311++G**	0.74	0.28	Si5 2I.spartan	0.80	0.36	Si11 2A.spartan	0.68	0.46	Si21 2A.spartan
HF	6-311++G**	0.65	0.33	Si5 F.spartan	0.77	0.41	Si11 F.spartan	0.63	0.50	Si21 F.spartan
ωB97X	6-311++G**	0.74	0.26	Si5 S.spartan	0.80	0.34	Si11 R.spartan	0.70	0.42	Si21 O.spartan
ωB97X-D	6-311++G**	0.76	0.27	Si5 B.spartan	0.83	0.35	Si11 Q.spartan	0.71	0.44	Si21 V.spartan
ωB97X-V	6-311++G**	0.82	0.23	Si5 X.spartan	0.91	0.30	Si11 P.spartan	0.78	0.38	Si21 K3.spartan
MP2	6-311++G**	0.65	0.33	Si5 E.spartan	0.78	0.41	Si11 T.spartan			
EDF2	6-311++G**	0.75	0.27	Si5 C.spartan	0.81	0.36	Si11 V.spartan	0.68	0.46	Si21 P.spartan
M06-2X	6-311++G**	0.74	0.27	Si5 D.spartan	0.75	0.35	Si11 W.spartan	0.59	0.46	Si21 Q.spartan
VV10	6-311++G**	0.79	0.25	Si5 2K.spartan	0.81	0.33	Si11 2C.spartan	0.67	0.43	Si21 2B.spartan
B97-D2	6-311++G**	0.80	0.27	Si5 2L.spartan	0.89	0.34	Si11 2D.spartan	0.73	0.44	Si21 2C.spartan
ωB97X-V	6-311++G(2df,2p)	1.23	0.10	Si5 2A.spartan	1.38	0.16	Si11 K.spartan	1.25	0.23	Si21 H.spartan
B3LYP	6-31+G	-0.13	0.52	Si5 N.spartan	0.55	0.36	Si11 B.spartan	0.79	0.13	Si21 D.spartan
B3LYP	6-31++G**	0.01	0.57	Si5 M.spartan	0.83	0.42	Si11 D.spartan	1.22	0.13	Si21 G.spartan
B3LYP	6-311++G(2d,p)	0.37	0.34	Si5 V.spartan	0.37	0.53	Si11 E.spartan	0.28	0.62	Si21 E.spartan
B3LYP	6-311++G(2df,2p)	1.25	0.10	Si5 Z.spartan	1.38	0.18	Si11 J.spartan	1.24	0.25	Si21 J.spartan
B3LYP	6-311++G(3df,2p)	1.06	0.04	Si5 O.spartan	0.94	0.12	Si11 M.spartan			
B3LYP	aug-cc-pVDZ	-0.48	0.00	Si5 H.spartan	-0.27	0.06	Si11 G.spartan	-0.01	0.30	Si21 C.spartan
B3LYP	aug-cc-pVTZ	0.41	0.22	Si5 L.spartan	0.53	0.07	Si11 C.spartan			
B3LYP	aug-cc-pVQZ	0.43	0.12	Si5 R.spartan						
B3LYP	def2-SVPD	-2.61	1.59	Si5 J.spartan	0.89	0.63	Si11 H.spartan	0.50	0.21	Si21 B.spartan
B3LYP	def2-TZVPD	0.80	0.01	Si5 U.spartan	0.63	0.11	Si11 L.spartan			
B3LYP	def2-QZVPD	1.16	0.00	Si5 Y.spartan						
NO diffuse functionals										
B3LYP	6-31G*	1.02	0.00	Si5 2O.spartan	0.99	0.00	Si11 2F.spartan	0.96	0.00	Si21 2F.spartan
B3LYP	cc-pVDZ	1.13	0.00	Si5 G.spartan	1.11	0.00	Si11 S.spartan	1.09	0.00	Si21 2E.spartan
B3LYP	cc-pVTZ	1.09	0.00	Si5 K.spartan	1.04	0.00	Si11 X.spartan			
B3LYP	cc-pVQZ	1.03	0.00	Si5 Q.spartan						
B3LYP	def2-SV(P)	1.08	0.00	Si5 I.spartan	1.06	0.00	Si11 I.spartan	1.05	0.00	Si21 2G.spartan
B3LYP	def2-TZVP	1.08	0.00	Si5 T.spartan	1.05	0.00	Si11 Y.spartan			
B3LYP	def2-QZVP	1.03	-0.01	Si5 W.spartan						

Table S31. For various computational methods and basis sets, this table provides computed Mulliken bond orders for the central SiO_4 unit within the *Si5* ($Si_5O_{16}H_{12}$), *Si11* ($Si_{11}O_{32}H_{20}$), and *Si21* ($Si_{21}O_{56}H_{28}$), clusters. The Si-O and O-O bond order averages (bold) are summarized in Table S30.

Si5 A.spartan		-2659.8115700 hartrees	
Si5	**DFT**	**B3LYP**	**6-311++G****
atom#1	atom#2	Bond Order	# electrons (BO x 2)
Si15	O23	0.65819	1.31639
Si15	O36	0.82422	1.64845
Si15	O39	0.82410	1.64820
Si15	O52	0.65721	1.31442
Si-O average		**0.74093**	1.48186
Si-O total		2.96373	**5.92745**
atom#1	atom#2	Bond Order	# electrons (BO x 2)
O23	O36	0.29540	0.59081
O23	O39	0.29128	0.58256
O23	O52	0.27316	0.54633
O36	O39	0.21831	0.43662
O36	O52	0.29043	0.58087
O39	O52	0.29638	0.59276
O-O average		**0.27750**	0.55499
O-O total		1.66497	**3.32994**
grand total bonding		4.62870	**9.25739**
percent Si-O		64.02938	**64.02938**
percent O-O		35.97062	**35.97062**
grand total non-bonding		3.37130	**6.74261**

Si11 A.spartan		-5606.9472951 hartrees	
Si11	**DFT**	**B3LYP**	**6-311++G****
atom#1	atom#2	Bond Order	# electrons (BO x 2)
Si15	O10	0.63512	1.27024
Si15	O11	0.97752	1.95504
Si15	O17	0.97033	1.94066
Si15	O32	0.63548	1.27096
Si-O average		**0.80461**	1.60923
Si-O total		3.21845	**6.43691**
atom#1	atom#2	Bond Order	# electrons (BO x 2)
O10	O11	0.41568	0.83137
O10	O17	0.35440	0.70881
O10	O32	0.40243	0.80486
O11	O17	0.24202	0.48403
O11	O32	0.35467	0.70934
O17	O32	0.40880	0.81759
O-O average		**0.36300**	0.72600
O-O total		2.17800	**4.35600**
grand total bonding		5.39645	**10.79291**
percent Si-O		59.64016	**59.64016**
percent O-O		40.35984	**40.35984**
grand total non-bonding		2.60355	**5.20709**

Si21 A.spartan		-10315.0041005 hartrees	
Si21	**DFT**	**B3LYP**	**6-311++G****
atom#1	atom#2	Bond Order	# electrons (BO x 2)
Si15	O23	0.71229	1.42458
Si15	O36	0.64146	1.28291
Si15	O39	0.64199	1.28398
Si15	O52	0.71251	1.42501
Si-O average		**0.67706**	1.35412
Si-O total		2.70825	**5.41649**
atom#1	atom#2	Bond Order	# electrons (BO x 2)
O23	O36	0.60687	1.21374
O23	O39	0.41618	0.83237
O23	O52	0.35945	0.71889
O36	O39	0.36056	0.72112
O36	O52	0.41443	0.82885
O39	O52	0.60805	1.21611
O-O average		**0.46092**	0.92185
O-O total		2.76554	**5.53108**
grand total bonding		5.47379	**10.94757**
percent Si-O		49.47666	**49.47666**
percent O-O		50.52334	**50.52334**
grand total non-bonding		2.52621	**5.05243**

Si5 2I.spartan		-2659.8479510 hartrees	
Si5	**DFT**	**B3LYP-D3**	**6-311++G****
atom#1	atom#2	Bond Order	# electrons (BO x 2)
Si15	O23	0.65819	1.31639
Si15	O36	0.82422	1.64845
Si15	O39	0.82410	1.64819
Si15	O52	0.65721	1.31442
Si-O average		**0.74093**	1.48186
Si-O total		2.96373	**5.92745**
atom#1	atom#2	Bond Order	# electrons (BO x 2)
O23	O36	0.29540	0.59081
O23	O39	0.29128	0.58256
O23	O52	0.27316	0.54633
O36	O39	0.21831	0.43662
O36	O52	0.29043	0.58087
O39	O52	0.29638	0.59276
O-O average		**0.27749**	0.55499
O-O total		1.66497	**3.32994**
grand total bonding		4.62870	**9.25739**
percent Si-O		64.02940	**64.02940**
percent O-O		35.97060	**35.97060**
grand total non-bonding		3.37130	**6.74261**

Si11 2A.spartan		-5607.0503679 hartrees	
Si11	**DFT**	**B3LYP-D3**	**6-311++G****
atom#1	atom#2	Bond Order	# electrons (BO x 2)
Si15	O10	0.63512	1.27025
Si15	O11	0.97752	1.95503
Si15	O17	0.97033	1.94066
Si15	O32	0.63548	1.27097
Si-O average		**0.80461**	1.60923
Si-O total		3.21845	**6.43691**
atom#1	atom#2	Bond Order	# electrons (BO x 2)
O10	O11	0.41568	0.83137
O10	O17	0.35440	0.70881
O10	O32	0.40243	0.80486
O11	O17	0.24202	0.48403
O11	O32	0.35467	0.70934
O17	O32	0.40879	0.81759
O-O average		**0.36300**	0.72600
O-O total		2.17800	**4.35599**
grand total bonding		5.39645	**10.79290**
percent Si-O		59.64019	**59.64019**
percent O-O		40.35981	**40.35981**
grand total non-bonding		2.60355	**5.20710**

Si21 2A.spartan		-10315.2322887 hartrees	
Si21	**DFT**	**B3LYP-D3**	**6-311++G****
atom#1	atom#2	Bond Order	# electrons (BO x 2)
Si15	O23	0.71229	1.42458
Si15	O36	0.64146	1.28292
Si15	O39	0.64199	1.28399
Si15	O52	0.71251	1.42501
Si-O average		**0.67706**	1.35413
Si-O total		2.70825	**5.41650**
atom#1	atom#2	Bond Order	# electrons (BO x 2)
O23	O36	0.60687	1.21374
O23	O39	0.41618	0.83237
O23	O52	0.35945	0.71889
O36	O39	0.36056	0.72112
O36	O52	0.41442	0.82885
O39	O52	0.60805	1.21611
O-O average		**0.46092**	0.92185
O-O total		2.76554	**5.53108**
grand total bonding		5.47379	**10.94758**
percent Si-O		49.47670	**49.47670**
percent O-O		50.52330	**50.52330**
grand total non-bonding		2.52621	**5.05242**

Si5 F.spartan		-2650.9716317 hartrees	
Si5		**HF**	**6-311++G****
atom#1	atom#2	Bond Order	# electrons (BO x 2)
Si15	O23	0.56158	1.12315
Si15	O36	0.73925	1.47849
Si15	O39	0.73981	1.47963
Si15	O52	0.56076	1.12153
Si-O average		**0.65035**	1.30070
Si-O total		2.60140	**5.20280**
atom#1	atom#2	Bond Order	# electrons (BO x 2)
O23	O36	0.34590	0.69181
O23	O39	0.34009	0.68018
O23	O52	0.33396	0.66793
O36	O39	0.28199	0.56399
O36	O52	0.33943	0.67887
O39	O52	0.34665	0.69329
O-O average		**0.33134**	0.66268
O-O total		1.98803	**3.97606**
grand total bonding		4.58943	**9.17886**
percent Si-O		56.68239	**56.68239**
percent O-O		43.31761	**43.31761**
grand total non-bonding		3.41057	**6.82114**

Si11 F.spartan		-5588.8082366 hartrees	
Si11		**HF**	**6-311++G****
atom#1	atom#2	Bond Order	# electrons (BO x 2)
Si15	O10	0.58677	1.17354
Si15	O11	0.96441	1.92881
Si15	O17	0.96434	1.92868
Si15	O32	0.58596	1.17193
Si-O average		**0.77537**	1.55074
Si-O total		3.10148	**6.20296**
atom#1	atom#2	Bond Order	# electrons (BO x 2)
O10	O11	0.46966	0.93932
O10	O17	0.38999	0.77998
O10	O32	0.44322	0.88643
O11	O17	0.30785	0.61571
O11	O32	0.39133	0.78266
O17	O32	0.46883	0.93766
O-O average		**0.41181**	0.82363
O-O total		2.47088	**4.94177**
grand total bonding		5.57236	**11.14473**
percent Si-O		55.65825	**55.65825**
percent O-O		44.34175	**44.34175**
grand total non-bonding		2.42764	**4.85527**

Si21 F.spartan		-10282.4618404 hartrees	
Si21		**HF**	**6-311++G****
atom#1	atom#2	Bond Order	# electrons (BO x 2)
Si15	O23	0.67361	1.34722
Si15	O36	0.61136	1.22272
Si15	O39	0.61138	1.22275
Si15	O52	0.61138	1.22275
Si-O average		**0.62693**	1.25386
Si-O total		2.50773	**5.01545**
atom#1	atom#2	Bond Order	# electrons (BO x 2)
O23	O36	0.66017	1.32034
O23	O39	0.43788	0.87577
O23	O52	0.38071	0.76142
O36	O39	0.44175	0.88350
O36	O52	0.43657	0.87314
O39	O52	0.66137	1.32274
O-O average		**0.50307**	1.00615
O-O total		3.01845	**6.03690**
grand total bonding		5.52617	**11.05235**
percent Si-O		45.37905	**45.37905**
percent O-O		54.62095	**54.62095**
grand total non-bonding		2.47383	**4.94765**

Si5 S.spartan		-2659.4018805 hartrees	
Si5		**ωB97X**	**6-311++G****
atom#1	atom#2	Bond Order	# electrons (BO x 2)
Si15	O23	0.66448	1.32896
Si15	O36	0.80965	1.61929
Si15	O39	0.81022	1.62043
Si15	O52	0.66361	1.32723
Si-O average		**0.73699**	1.47398
Si-O total		2.94796	**5.89591**
atom#1	atom#2	Bond Order	# electrons (BO x 2)
O23	O36	0.28440	0.56880
O23	O39		0.00000
O23	O52	0.24347	0.48694
O36	O39		0.00000
O36	O52		0.00000
O39	O52		0.00000
O-O average		**0.26394**	0.17596
O-O total		0.52787	**1.05574**
grand total bonding		3.47583	**6.95165**
percent Si-O		84.81308	**84.81308**
percent O-O		15.18692	**15.18692**
grand total non-bonding		4.52417	**9.04835**

Si11 R.spartan		-5606.1323509 hartrees	
Si11	**DFT**	**ωB97X**	**6-311++G****
atom#1	atom#2	Bond Order	# electrons (BO x 2)
Si15	O10	0.64245	1.28491
Si15	O11	0.95552	1.91103
Si15	O17	0.95575	1.91150
Si15	O32	0.64196	1.28393
Si-O average		**0.79892**	1.59784
Si-O total		3.19568	**6.39136**
atom#1	atom#2	Bond Order	# electrons (BO x 2)
O10	O11	0.38666	0.77332
O10	O17	0.32886	0.65772
O10	O32	0.36522	0.73045
O11	O17	0.21534	0.43067
O11	O32	0.33015	0.66030
O17	O32	0.38581	0.77161
O-O average		**0.33534**	0.67068
O-O total		2.01204	**4.02408**
grand total bonding		5.20772	**10.41544**
percent Si-O		61.36432	**61.36432**
percent O-O		38.63568	**38.63568**
grand total non-bonding		2.79228	**5.58456**

Si21 O.spartan		-10313.5778516 hartrees	
Si21	**DFT**	**ωB97X**	**6-311++G****
atom#1	atom#2	Bond Order	# electrons (BO x 2)
Si15	O23	0.72407	1.44813
Si15	O36	0.68010	1.36019
Si15	O39	0.68020	1.36040
Si15	O52	0.72427	1.44854
Si-O average		**0.70216**	1.40431
Si-O total		2.80863	**5.61726**
atom#1	atom#2	Bond Order	# electrons (BO x 2)
O23	O36	0.56421	1.12843
O23	O39	0.38254	0.76509
O23	O52	0.31363	0.62726
O36	O39	0.32285	0.64569
O36	O52	0.38141	0.76282
O39	O52	0.56521	1.13042
O-O average		**0.42164**	0.84328
O-O total		2.52985	**5.05969**
grand total bonding		5.33847	**10.67695**
percent Si-O		52.61106	**52.61106**
percent O-O		47.38894	**47.38894**
grand total non-bonding		2.66153	**5.32305**

Si5 B.spartan		-2659.3122429 hartrees	
Si5	**DFT**	**ωB97X-D**	**6-311++G****
atom#1	atom#2	Bond Order	# electrons (BO x 2)
Si15	O23	0.68005	1.36011
Si15	O36	0.84501	1.69003
Si15	O39	0.84556	1.69112
Si15	O52	0.67932	1.35865
Si-O average		**0.76249**	1.52497
Si-O total		3.04995	**6.09990**
atom#1	atom#2	Bond Order	# electrons (BO x 2)
O23	O36	0.29132	0.58263
O23	O39	0.28355	0.56710
O23	O52	0.25594	0.51189
O36	O39	0.20426	0.40853
O36	O52	0.28288	0.56577
O39	O52	0.29201	0.58402
O-O average		**0.26833**	0.53666
O-O total		1.60997	**3.21993**
grand total bonding		4.65991	**9.31983**
percent Si-O		65.45075	**65.45075**
percent O-O		34.54925	**34.54925**
grand total non-bonding		3.34009	**6.68017**

Si11 Q.spartan		-5605.9496482 hartrees	
Si11	**DFT**	**ωB97X-D**	**6-311++G****
atom#1	atom#2	Bond Order	# electrons (BO x 2)
Si15	O10	0.66992	1.33984
Si15	O11	0.98231	1.96463
Si15	O17	0.98290	1.96579
Si15	O32	0.66901	1.33803
Si-O average		**0.82604**	1.65207
Si-O total		3.30414	**6.60829**
atom#1	atom#2	Bond Order	# electrons (BO x 2)
O10	O11	0.39965	0.79930
O10	O17	0.34117	0.68233
O10	O32	0.37785	0.75569
O11	O17	0.22771	0.45542
O11	O32	0.34274	0.68548
O17	O32	0.39849	0.79699
O-O average		**0.34793**	0.69587
O-O total		2.08761	**4.17521**
grand total bonding		5.39175	**10.78350**
percent Si-O		61.28149	**61.28149**
percent O-O		38.71851	**38.71851**
grand total non-bonding		2.60825	**5.21650**

Si21 V.spartan		-10313.2576136 hartrees	
Si21	**DFT**	**ωB97X-D**	**6-311++G****
atom#1	atom#2	Bond Order	# electrons (BO x 2)
Si15	O23	0.73804	1.47608
Si15	O36	0.67990	1.35980
Si15	O39	0.67966	1.35932
Si15	O52	0.73822	1.47645
Si-O average		**0.70896**	1.41791
Si-O total		2.83582	**5.67164**
atom#1	atom#2	Bond Order	# electrons (BO x 2)
O23	O36	0.58448	1.16896
O23	O39	0.39653	0.79306
O23	O52	0.33395	0.66789
O36	O39	0.34235	0.68471
O36	O52	0.39521	0.79043
O39	O52	0.58557	1.17114
O-O average		**0.43968**	0.87936
O-O total		2.63809	**5.27618**
grand total bonding		5.47391	**10.94783**
percent Si-O		51.80611	**51.80611**
percent O-O		48.19389	**48.19389**
grand total non-bonding		2.52609	**5.05217**

Si5 X.spartan		-2659.0949208 hartrees	
Si5	**DFT**	**ωB97X-V**	**6-311++G****
atom#1	atom#2	Bond Order	# electrons (BO x 2)
Si15	O23	0.74991	1.49982
Si15	O36	0.89749	1.79497
Si15	O39	0.89811	1.79623
Si15	O52	0.74911	1.49822
Si-O average		**0.82366**	1.64731
Si-O total		3.29462	**6.58924**
atom#1	atom#2	Bond Order	# electrons (BO x 2)
O23	O36	0.25663	0.51326
O23	O39	0.24765	0.49530
O23	O52	0.21907	0.43814
O36	O39	0.18048	0.36095
O36	O52	0.24708	0.49415
O39	O52	0.25728	0.51456
O-O average		**0.23470**	0.46939
O-O total		1.40818	**2.81637**
grand total bonding		4.70280	**9.40561**
percent Si-O		70.05652	**70.05652**
percent O-O		29.94348	**29.94348**
grand total non-bonding		3.29720	**6.59439**

Si11 P.spartan		-5605.4964849 hartrees	
Si11	**DFT**	**ωB97X-V**	**6-311++G****
atom#1	atom#2	Bond Order	# electrons (BO x 2)
Si15	O10	0.76370	1.52740
Si15	O11	1.05350	2.10700
Si15	O17	1.05325	2.10649
Si15	O32	0.76336	1.52673
Si-O average		**0.90845**	1.81691
Si-O total		3.63381	**7.26762**
atom#1	atom#2	Bond Order	# electrons (BO x 2)
O10	O11	0.35377	0.70753
O10	O17	0.29538	0.59075
O10	O32	0.32373	0.64746
O11	O17	0.18349	0.36698
O11	O32	0.29630	0.59259
O17	O32	0.35316	0.70633
O-O average		**0.30097**	0.60194
O-O total		1.80583	**3.61166**
grand total bonding		5.43964	**10.87928**
percent Si-O		66.80243	**66.80243**
percent O-O		33.19757	**33.19757**
grand total non-bonding		2.56036	**5.12072**

Si21 K3.spartan		-10312.4264021 hartrees	
Si21	**DFT**	**ωB97X-V**	**6-311++G****
atom#1	atom#2	Bond Order	# electrons (BO x 2)
Si15	O23	0.81530	1.63060
Si15	O36	0.75254	1.50507
Si15	O39	0.75290	1.50579
Si15	O52	0.81537	1.63073
Si-O average		**0.78403**	1.56805
Si-O total		3.13610	**6.27220**
atom#1	atom#2	Bond Order	# electrons (BO x 2)
O23	O36	0.52468	1.04937
O23	O39	0.34429	0.68858
O23	O52	0.28300	0.56600
O36	O39	0.28789	0.57579
O36	O52	0.34322	0.68643
O39	O52	0.52571	1.05142
O-O average		**0.38480**	0.76960
O-O total		2.30880	**4.61759**
grand total bonding		5.44490	**10.88979**
percent Si-O		57.59708	**57.59708**
percent O-O		42.40292	**42.40292**
grand total non-bonding		2.55510	**5.11021**

Si5 E.spartan		-2650.9716316 hartrees	
Si5	**MP**	**MP2**	**6-311++G****
atom#1	atom#2	Bond Order	# electrons (BO x 2)
Si15	O23	0.56157	1.12315
Si15	O36	0.73924	1.47849
Si15	O39	0.73981	1.47962
Si15	O52	0.56076	1.12152
Si-O average		**0.65035**	1.30070
Si-O total		2.60139	**5.20278**
atom#1	atom#2	Bond Order	# electrons (BO x 2)
O23	O36	0.34591	0.69181
O23	O39	0.34009	0.68019
O23	O52	0.33396	0.66793
O36	O39	0.28199	0.56399
O36	O52	0.33943	0.67887
O39	O52	0.34665	0.69329
O-O average		**0.33134**	0.66268
O-O total		1.98804	**3.97608**
grand total bonding		4.58943	**9.17886**
percent Si-O		56.68224	**56.68224**
percent O-O		43.31776	**43.31776**
grand total non-bonding		3.41057	**6.82114**

Si11 T.spartan		-5588.8082366 hartrees	
Si11	**MP**	**MP2**	**6-311++G****
atom#1	atom#2	Bond Order	# electrons (BO x 2)
Si15	O10	0.58677	1.17354
Si15	O11	0.96440	1.92880
Si15	O17	0.96434	1.92869
Si15	O32	0.58597	1.17194
Si-O average		**0.77537**	1.55074
Si-O total		3.10149	**6.20297**
atom#1	atom#2	Bond Order	# electrons (BO x 2)
O10	O11	0.46966	0.93933
O10	O17	0.38999	0.77998
O10	O32	0.44321	0.88643
O11	O17	0.30785	0.61571
O11	O32	0.39133	0.78266
O17	O32	0.46883	0.93766
O-O average		**0.41181**	0.82363
O-O total		2.47088	**4.94176**
grand total bonding		5.57236	**11.14473**
percent Si-O		55.65834	**55.65834**
percent O-O		44.34166	**44.34166**
grand total non-bonding		2.42764	**4.85527**

Si5 C.spartan		-2659.2724113 hartrees	
Si5	**DFT**	**EDF2**	**6-311++G****
atom#1	atom#2	Bond Order	# electrons (BO x 2)
Si15	O23	0.66681	1.33362
Si15	O36	0.83308	1.66615
Si15	O39	0.83297	1.66593
Si15	O52	0.66583	1.33166
Si-O average		**0.74967**	1.49934
Si-O total		2.99868	**5.99736**
atom#1	atom#2	Bond Order	# electrons (BO x 2)
O23	O36	0.29250	0.58501
O23	O39	0.28877	0.57755
O23	O52	0.27079	0.54158
O36	O39	0.21251	0.42502
O36	O52	0.28793	0.57587
O39	O52	0.29348	0.58696
O-O average		**0.27433**	0.54866
O-O total		1.64599	**3.29199**
grand total bonding		4.64467	**9.28935**
percent Si-O		64.56171	**64.56171**
percent O-O		35.43829	**35.43829**
grand total non-bonding		3.35533	**6.71065**

Si11 V.spartan		-5605.8880015 hartrees	
Si11	**DFT**	**EDF2**	**6-311++G****
atom#1	atom#2	Bond Order	# electrons (BO x 2)
Si15	O10	0.63811	1.27623
Si15	O11	0.97907	1.95813
Si15	O17	0.97193	1.94386
Si15	O32	0.63905	1.27810
Si-O average		**0.80704**	1.61408
Si-O total		3.22816	**6.45632**
atom#1	atom#2	Bond Order	# electrons (BO x 2)
O10	O11	0.41266	0.82532
O10	O17	0.35304	0.70608
O10	O32	0.40249	0.80499
O11	O17	0.23708	0.47416
O11	O32	0.35321	0.70642
O17	O32	0.40573	0.81146
O-O average		**0.36070**	0.72141
O-O total		2.16422	**4.32843**
grand total bonding		5.39238	**10.78475**
percent Si-O		59.86527	**59.86527**
percent O-O		40.13473	**40.13473**
grand total non-bonding		2.60762	**5.21525**

Si21 P.spartan		-10313.1832117 hartrees	
Si21	**DFT**	**EDF2**	**6-311++G****
atom#1	atom#2	Bond Order	# electrons (BO x 2)
Si15	O23	0.71228	1.42456
Si15	O36	0.64219	1.28438
Si15	O39	0.64266	1.28532
Si15	O52	0.71239	1.42478
Si-O average		**0.67738**	1.35476
Si-O total		2.70952	**5.41903**
atom#1	atom#2	Bond Order	# electrons (BO x 2)
O23	O36	0.60534	1.21068
O23	O39	0.41719	0.83439
O23	O52	0.36190	0.72379
O36	O39	0.35616	0.71232
O36	O52	0.41546	0.83092
O39	O52	0.60656	1.21312
O-O average		**0.46044**	0.92087
O-O total		2.76262	**5.52524**
grand total bonding		5.47214	**10.94427**
percent Si-O		49.51480	**49.51480**
percent O-O		50.48520	**50.48520**
grand total non-bonding		2.52786	**5.05573**

Si5 D.spartan		-2659.2536265 hartrees	
Si5	**DFT**	**M06-2X**	**6-311++G****
atom#1	atom#2	Bond Order	# electrons (BO x 2)
Si15	O23	0.65273	1.30545
Si15	O36	0.82247	1.64494
Si15	O39	0.82288	1.64576
Si15	O52	0.65191	1.30381
Si-O average		**0.73750**	1.47499
Si-O total		2.94999	**5.89997**
atom#1	atom#2	Bond Order	# electrons (BO x 2)
O23	O36	0.29278	0.58557
O23	O39	0.28164	0.56328
O23	O52	0.26826	0.53651
O36	O39	0.19378	0.38757
O36	O52	0.28112	0.56224
O39	O52	0.29355	0.58710
O-O average		**0.26852**	0.53704
O-O total		1.61113	**3.22226**
grand total bonding		4.56112	**9.12223**
percent Si-O		64.67684	**64.67684**
percent O-O		35.32316	**35.32316**
grand total non-bonding		3.43888	**6.87777**

Si11 W.spartan		-5605.8544464 hartrees	
Si11	**DFT**	**M06-2X**	**6-311++G****
atom#1	atom#2	Bond Order	# electrons (BO x 2)
Si15	O10	0.62969	1.25938
Si15	O11	0.87979	1.75959
Si15	O17	0.88192	1.76383
Si15	O32	0.62843	1.25687
Si-O average		**0.75496**	1.50992
Si-O total		3.01984	**6.03967**
atom#1	atom#2	Bond Order	# electrons (BO x 2)
O10	O11	0.40046	0.80091
O10	O17	0.34265	0.68531
O10	O32	0.38790	0.77579
O11	O17	0.22409	0.44818
O11	O32	0.34398	0.68795
O17	O32	0.39985	0.79971
O-O average		**0.34982**	0.69964
O-O total		2.09892	**4.19785**
grand total bonding		5.11876	**10.23752**
percent Si-O		58.99544	**58.99544**
percent O-O		41.00456	**41.00456**
grand total non-bonding		2.88124	**5.76248**

Si21 Q.spartan		-10313.1229884 hartrees	
Si21	**DFT**	**M06-2X**	**6-311++G****
atom#1	atom#2	Bond Order	# electrons (BO x 2)
Si15	O23	0.64533	1.29066
Si15	O36	0.53757	1.07514
Si15	O39	0.53801	1.07602
Si15	O52	0.64579	1.29158
Si-O average		**0.59168**	1.18335
Si-O total		2.36671	**4.73341**
atom#1	atom#2	Bond Order	# electrons (BO x 2)
O23	O36	0.59202	1.18404
O23	O39	0.41260	0.82520
O23	O52	0.36473	0.72945
O36	O39	0.35693	0.71386
O36	O52	0.41138	0.82277
O39	O52	0.59312	1.18625
O-O average		**0.45513**	0.91026
O-O total		2.73078	**5.46157**
grand total bonding		5.09749	**10.19498**
percent Si-O		46.42886	**46.42886**
percent O-O		53.57114	**53.57114**
grand total non-bonding		2.90251	**5.80502**

Si5 2K.spartan		-2662.4686609 hartrees	
Si5	**DFT**	**VV10**	**6-311++G****
atom#1	atom#2	Bond Order	# electrons (BO x 2)
Si15	O23	0.70619	1.41238
Si15	O36	0.86456	1.72913
Si15	O39	0.86515	1.73029
Si15	O52	0.70573	1.41146
Si-O average		**0.78541**	1.57082
Si-O total		3.14163	**6.28327**
atom#1	atom#2	Bond Order	# electrons (BO x 2)
O23	O36	0.26811	0.53621
O23	O39	0.26090	0.52181
O23	O52	0.23815	0.47631
O36	O39	0.18004	0.36007
O36	O52	0.26016	0.52033
O39	O52	0.26867	0.53735
O-O average		**0.24601**	0.49201
O-O total		1.47604	**2.95207**
grand total bonding		4.61767	**9.23534**
percent Si-O		68.03503	**68.03503**
percent O-O		31.96497	**31.96497**
grand total non-bonding		3.38233	**6.76466**

Si11 2C.spartan		-5612.5079278 hartrees	
Si11	**DFT**	**VV10**	**6-311++G****
atom#1	atom#2	Bond Order	# electrons (BO x 2)
Si15	O10	0.65694	1.31389
Si15	O11	0.95946	1.91892
Si15	O17	0.95915	1.91829
Si15	O32	0.65708	1.31416
Si-O average		**0.80816**	1.61631
Si-O total		3.23263	**6.46526**
atom#1	atom#2	Bond Order	# electrons (BO x 2)
O10	O11	0.38287	0.76574
O10	O17	0.32817	0.65634
O10	O32	0.36855	0.73710
O11	O17	0.20597	0.41193
O11	O32	0.32935	0.65869
O17	O32	0.38128	0.76257
O-O average		**0.33270**	0.66540
O-O total		1.99619	**3.99238**
grand total bonding		5.22882	**10.45764**
percent Si-O		61.82331	**61.82331**
percent O-O		38.17669	**38.17669**
grand total non-bonding		2.77118	**5.54236**

Si21 2B.spartan		-10325.1551452 hartrees	
Si21	**DFT**	**VV10**	**6-311++G****
atom#1	atom#2	Bond Order	# electrons (BO x 2)
Si15	O23	0.70893	1.41785
Si15	O36	0.63017	1.26033
Si15	O39	0.63043	1.26085
Si15	O52	0.70953	1.41905
Si-O average		**0.66976**	1.33952
Si-O total		2.67904	**5.35809**
atom#1	atom#2	Bond Order	# electrons (BO x 2)
O23	O36	0.57369	1.14738
O23	O39	0.39527	0.79054
O23	O52	0.33584	0.67168
O36	O39	0.31825	0.63649
O36	O52	0.39370	0.78740
O39	O52	0.57445	1.14890
O-O average		**0.43187**	0.86373
O-O total		2.59120	**5.18240**
grand total bonding		5.27024	**10.54049**
percent Si-O		50.83343	**50.83343**
percent O-O		49.16657	**49.16657**
grand total non-bonding		2.72976	**5.45951**

Si5 2L.spartan		-2659.0236276 hartrees	
Si5	**DFT**	**B97-D2**	**6-311++G****
atom#1	atom#2	Bond Order	# electrons (BO x 2)
Si15	O23	0.71646	1.43292
Si15	O36	0.88776	1.77552
Si15	O39	0.88848	1.77697
Si15	O52	0.71539	1.43078
Si-O average		**0.80202**	1.60405
Si-O total		3.20809	**6.41618**
atom#1	atom#2	Bond Order	# electrons (BO x 2)
O23	O36	0.28111	0.56223
O23	O39	0.27831	0.55663
O23	O52	0.26345	0.52690
O36	O39	0.21257	0.42514
O36	O52	0.27780	0.55560
O39	O52	0.28189	0.56378
O-O average		**0.26586**	0.53171
O-O total		1.59513	**3.19027**
grand total bonding		4.80323	**9.60645**
percent Si-O		66.79036	**66.79036**
percent O-O		33.20964	**33.20964**
grand total non-bonding		3.19677	**6.39355**

Si11 2D.spartan		-5605.3339441 hartrees	
Si11	**DFT**	**B97-D2**	**6-311++G****
atom#1	atom#2	Bond Order	# electrons (BO x 2)
Si15	O10	0.72252	1.44505
Si15	O11	1.05418	2.10835
Si15	O17	1.05461	2.10922
Si15	O32	0.71544	1.43087
Si-O average		**0.88669**	1.77337
Si-O total		3.54674	**7.09349**
atom#1	atom#2	Bond Order	# electrons (BO x 2)
O10	O11	0.39396	0.78793
O10	O17	0.32857	0.65713
O10	O32	0.38179	0.76359
O11	O17	0.23151	0.46301
O11	O32	0.33233	0.66466
O17	O32	0.39627	0.79254
O-O average		**0.34407**	0.68814
O-O total		2.06443	**4.12886**
grand total bonding		5.61117	**11.22234**
percent Si-O		63.20860	**63.20860**
percent O-O		36.79140	**36.79140**
grand total non-bonding		2.38883	**4.77766**

Si21 2C.spartan		-10312.1070664 hartrees	
Si21	**DFT**	**B97-D2**	**6-311++G****
atom#1	atom#2	Bond Order	# electrons (BO x 2)
Si15	O23	0.77281	1.54563
Si15	O36	0.68024	1.36048
Si15	O39	0.68050	1.36099
Si15	O52	0.77255	1.54510
Si-O average		**0.72653**	1.45305
Si-O total		2.90610	**5.81220**
atom#1	atom#2	Bond Order	# electrons (BO x 2)
O23	O36	0.58140	1.16280
O23	O39	0.38336	0.76673
O23	O52	0.33778	0.67556
O36	O39	0.34957	0.69914
O36	O52	0.38228	0.76457
O39	O52	0.58260	1.16520
O-O average		**0.43617**	0.87233
O-O total		2.61700	**5.23399**
grand total bonding		5.52310	**11.04620**
percent Si-O		52.61723	**52.61723**
percent O-O		47.38277	**47.38277**
grand total non-bonding		2.47690	**4.95380**

Si5 2A.spartan		-2659.2424561 hartrees	
Si5	**DFT**	**wB97X-V**	**6-311++G(2df,2p)**
atom#1	atom#2	Bond Order	# electrons (BO x 2)
Si15	O23	1.24922	2.49845
Si15	O36	1.20379	2.40759
Si15	O39	1.20448	2.40897
Si15	O52	1.24919	2.49838
Si-O average		**1.22667**	2.45334
Si-O total		4.90669	**9.81338**
atom#1	atom#2	Bond Order	# electrons (BO x 2)
O23	O36	0.08523	0.17046
O23	O39	0.08305	0.16610
O23	O52	0.18234	0.36467
O36	O39	0.06964	0.13928
O36	O52	0.08291	0.16583
O39	O52	0.08527	0.17053
O-O average		**0.09807**	0.19615
O-O total		0.58844	**1.17688**
grand total bonding		5.49513	**10.99026**
percent Si-O		89.29158	**89.29158**
percent O-O		10.70842	**10.70842**
grand total non-bonding		2.50487	**5.00974**

Si11 K.spartan		-5605.8628567 hartrees	
Si11	**DFT**	**wB97X-V**	**6-311++G(2df,2p)**
atom#1	atom#2	Bond Order	# electrons (BO x 2)
Si15	O10	1.25347	2.50695
Si15	O11	1.50488	3.00976
Si15	O17	1.50118	3.00237
Si15	O32	1.25381	2.50761
Si-O average		**1.37834**	2.75667
Si-O total		5.51334	**11.02669**
atom#1	atom#2	Bond Order	# electrons (BO x 2)
O10	O11	0.14825	0.29651
O10	O17	0.14780	0.29561
O10	O32	0.20323	0.40647
O11	O17	0.17094	0.34188
O11	O32	0.14767	0.29534
O17	O32	0.14790	0.29579
O-O average		**0.16097**	0.32193
O-O total		0.96579	**1.93159**
grand total bonding		6.47914	**12.95827**
percent Si-O		85.09378	**85.09378**
percent O-O		14.90622	**14.90622**
grand total non-bonding		1.52086	**3.04173**

Si21 H.spartan		-10313.0877363 hartrees	
Si21	**DFT**	**wB97X-V**	**6-311++G(2df,2p)**
atom#1	atom#2	Bond Order	# electrons (BO x 2)
Si15	O23	1.08882	2.17764
Si15	O36	1.40956	2.81913
Si15	O39	1.41155	2.82311
Si15	O52	1.08995	2.17990
Si-O average		**1.24997**	2.49994
Si-O total		4.99989	**9.99977**
atom#1	atom#2	Bond Order	# electrons (BO x 2)
O23	O36	0.28286	0.56572
O23	O39	0.32796	0.65592
O23	O52	0.17277	0.34554
O36	O39	-0.03530	-0.07060
O36	O52	0.32689	0.65378
O39	O52	0.28521	0.57042
O-O average		**0.22673**	0.45346
O-O total		1.36039	**2.72079**
grand total bonding		6.36028	**12.72056**
percent Si-O		78.61110	**78.61110**
percent O-O		21.38890	**21.38890**
grand total non-bonding		1.63972	**3.27944**

Si5 N.spartan		-2658.4967452 hartrees	
Si5	**DFT**	**B3LYP**	**6-31+G**
atom#1	atom#2	Bond Order	# electrons (BO x 2)
Si15	O23	-0.22138	-0.44276
Si15	O36	-0.03239	-0.06479
Si15	O39	-0.03551	-0.07103
Si15	O52	-0.22106	-0.44211
Si-O average		**-0.12759**	-0.25517
Si-O total		-0.51035	**-1.02070**
atom#1	atom#2	Bond Order	# electrons (BO x 2)
O23	O36	0.49128	0.98256
O23	O39	0.47268	0.94536
O23	O52	0.50500	1.01000
O36	O39	0.70985	1.41971
O36	O52	0.47063	0.94126
O39	O52	0.49384	0.98768
O-O average		**0.52388**	1.04776
O-O total		3.14329	**6.28657**
grand total bonding		2.63294	**5.26588**
percent Si-O		-19.38319	**-19.38319**
percent O-O		119.38319	**119.38319**
grand total non-bonding		5.36706	**10.73412**

Si11 B.spartan		-5604.2964302 hartrees	
Si11	**DFT**	**B3LYP**	**6-31+G**
atom#1	atom#2	Bond Order	# electrons (BO x 2)
Si15	O10	0.12653	0.25307
Si15	O11	0.97853	1.95707
Si15	O17	0.97623	1.95245
Si15	O32	0.13189	0.26378
Si-O average		**0.55330**	1.10659
Si-O total		2.21318	**4.42636**
atom#1	atom#2	Bond Order	# electrons (BO x 2)
O10	O11	0.27450	0.54900
O10	O17	0.26331	0.52662
O10	O32	0.59989	1.19977
O11	O17	0.50992	1.01983
O11	O32	0.26628	0.53255
O17	O32	0.26599	0.53198
O-O average		**0.36331**	0.72663
O-O total		2.17988	**4.35975**
grand total bonding		4.39306	**8.78612**
percent Si-O		50.37907	**50.37907**
percent O-O		49.62093	**49.62093**
grand total non-bonding		3.60694	**7.21388**

Si21 D.spartan		-10310.3387677 hartrees	
Si21	**DFT**	**B3LYP**	**6-31+G**
atom#1	atom#2	Bond Order	# electrons (BO x 2)
Si15	O23	0.75049	1.50097
Si15	O36	0.81867	1.63735
Si15	O39	0.82023	1.64046
Si15	O52	0.75395	1.50790
Si-O average		**0.78584**	1.57167
Si-O total		3.14334	**6.28668**
atom#1	atom#2	Bond Order	# electrons (BO x 2)
O23	O36	0.03520	0.07039
O23	O39	0.15697	0.31393
O23	O52	0.32416	0.64832
O36	O39	0.07270	0.14540
O36	O52	0.15335	0.30670
O39	O52	0.03622	0.07244
O-O average		**0.12976**	0.25953
O-O total		0.77859	**1.55718**
grand total bonding		3.92193	**7.84386**
percent Si-O		80.14780	**80.14780**
percent O-O		19.85220	**19.85220**
grand total non-bonding		4.07807	**8.15614**

Si5 M.spartan		-2659.3424302 hartrees	
Si5	**DFT**	**B3LYP**	**6-31++G****
atom#1	atom#2	Bond Order	# electrons (BO x 2)
Si15	O23	-0.03936	-0.07872
Si15	O36	0.05455	0.10909
Si15	O39	0.05372	0.10744
Si15	O52	-0.04106	-0.08212
Si-O average		**0.00696**	0.01392
Si-O total		0.02785	**0.05569**
atom#1	atom#2	Bond Order	# electrons (BO x 2)
O23	O36	0.59932	1.19865
O23	O39	0.51934	1.03868
O23	O52	0.45161	0.90322
O36	O39	0.73126	1.46251
O36	O52	0.51849	1.03698
O39	O52	0.60100	1.20200
O-O average		**0.57017**	1.14034
O-O total		3.42102	**6.84204**
grand total bonding		3.44887	**6.89773**
percent Si-O		0.80740	**0.80740**
percent O-O		99.19260	**99.19260**
grand total non-bonding		4.55113	**9.10227**

Si11 D.spartan		-5605.9912622 hartrees	
Si11	**DFT**	**B3LYP**	**6-31++G****
atom#1	atom#2	Bond Order	# electrons (BO x 2)
Si15	O10	0.48903	0.97806
Si15	O11	1.17134	2.34268
Si15	O17	1.16684	2.33368
Si15	O32	0.49470	0.98940
Si-O average		**0.83048**	1.66096
Si-O total		3.32191	**6.64382**
atom#1	atom#2	Bond Order	# electrons (BO x 2)
O10	O11	0.29843	0.59686
O10	O17	0.44814	0.89628
O10	O32	0.57122	1.14243
O11	O17	0.49018	0.98036
O11	O32	0.45002	0.90005
O17	O32	0.28905	0.57810
O-O average		**0.42451**	0.84901
O-O total		2.54704	**5.09409**
grand total bonding		5.86896	**11.73791**
percent Si-O		56.60143	**56.60143**
percent O-O		43.39857	**43.39857**
grand total non-bonding		2.13104	**4.26209**

Si21 G.spartan		-10313.2985398 hartrees	
Si21	**DFT**	**B3LYP**	**6-31++G****
atom#1	atom#2	Bond Order	# electrons (BO x 2)
Si15	O23	1.14424	2.28849
Si15	O36	1.28785	2.57569
Si15	O39	1.28936	2.57872
Si15	O52	1.14603	2.29207
Si-O average		**1.21687**	2.43374
Si-O total		4.86748	**9.73497**
atom#1	atom#2	Bond Order	# electrons (BO x 2)
O23	O36	0.14215	0.28429
O23	O39	0.05377	0.10753
O23	O52	0.20011	0.40021
O36	O39	0.15936	0.31873
O36	O52	0.05321	0.10643
O39	O52	0.14155	0.28310
O-O average		**0.12502**	0.25005
O-O total		0.75015	**1.50030**
grand total bonding		5.61763	**11.23526**
percent Si-O		86.64655	**86.64655**
percent O-O		13.35345	**13.35345**
grand total non-bonding		2.38237	**4.76474**

Si5 V.spartan		-2659.9106885 hartrees	
Si5	**DFT**	**B3LYP**	**6-311++G(2d,p)**
atom#1	atom#2	Bond Order	# electrons (BO x 2)
Si15	O23	0.32522	0.65043
Si15	O36	0.41664	0.83328
Si15	O39	0.41452	0.82905
Si15	O52	0.32433	0.64865
Si-O average		**0.37018**	0.74035
Si-O total		1.48071	**2.96142**
atom#1	atom#2	Bond Order	# electrons (BO x 2)
O23	O36	0.31635	0.63270
O23	O39	0.35093	0.70187
O23	O52	0.43905	0.87811
O36	O39	0.28099	0.56197
O36	O52	0.35023	0.70046
O39	O52	0.31715	0.63431
O-O average		**0.34245**	0.68490
O-O total		2.05471	**4.10942**
grand total bonding		3.53542	**7.07083**
percent Si-O		41.88213	**41.88213**
percent O-O		58.11787	**58.11787**
grand total non-bonding		4.46458	**8.92917**

Si11 E.spartan		-5607.1518805 hartrees	
Si11	**DFT**	**B3LYP**	**6-311++G(2d,p)**
atom#1	atom#2	Bond Order	# electrons (BO x 2)
Si15	O10	0.08617	0.17235
Si15	O11	0.66182	1.32364
Si15	O17	0.65666	1.31332
Si15	O32	0.08896	0.17792
Si-O average		**0.37340**	0.74681
Si-O total		1.49361	**2.98722**
atom#1	atom#2	Bond Order	# electrons (BO x 2)
O10	O11	0.55831	1.11662
O10	O17	0.48924	0.97848
O10	O32	0.65251	1.30501
O11	O17	0.45812	0.91623
O11	O32	0.48910	0.97820
O17	O32	0.55075	1.10150
O-O average		**0.53300**	1.06601
O-O total		3.19802	**6.39604**
grand total bonding		4.69163	**9.38326**
percent Si-O		31.83566	**31.83566**
percent O-O		68.16434	**68.16434**
grand total non-bonding		3.30837	**6.61674**

Si21 E.spartan		-10315.3759569 hartrees	
Si21	**DFT**	**B3LYP**	**6-311++G(2d,p)**
atom#1	atom#2	Bond Order	# electrons (BO x 2)
Si15	O23	0.24169	0.48338
Si15	O36	0.32215	0.64430
Si15	O39	0.31973	0.63947
Si15	O52	0.24268	0.48536
Si-O average		**0.28156**	0.56313
Si-O total		1.12626	**2.25251**
atom#1	atom#2	Bond Order	# electrons (BO x 2)
O23	O36	0.75662	1.51324
O23	O39	0.52087	1.04173
O23	O52	0.61979	1.23958
O36	O39	0.57099	1.14198
O36	O52	0.51852	1.03704
O39	O52	0.75787	1.51574
O-O average		**0.62411**	1.24822
O-O total		3.74465	**7.48931**
grand total bonding		4.87091	**9.74182**
percent Si-O		23.12210	**23.12210**
percent O-O		76.87790	**76.87790**
grand total non-bonding		3.12909	**6.25818**

Si5 Z.spartan		-2659.9863655 hartrees	
**Si5**	**DFT**	**B3LYP**	**6-311++G(2df,2p)**
atom#1	atom#2	Bond Order	# electrons (BO x 2)
Si15	O23	1.25249	2.50497
Si15	O36	1.25418	2.50836
Si15	O39	1.25438	2.50876
Si15	O52	1.25241	2.50483
Si-O average		**1.25337**	2.50673
Si-O total		5.01346	**10.02692**
atom#1	atom#2	Bond Order	# electrons (BO x 2)
O23	O36	0.08200	0.16400
O23	O39	0.08305	0.16609
O23	O52	0.19665	0.39330
O36	O39	0.06779	0.13559
O36	O52	0.08292	0.16584
O39	O52	0.08217	0.16435
O-O average		**0.09910**	0.19819
O-O total		0.59458	**1.18916**
grand total bonding		5.60804	**11.21608**
percent Si-O		89.39771	**89.39771**
percent O-O		10.60229	**10.60229**
grand total non-bonding		2.39196	**4.78392**

Si11 J.spartan		-5607.3098332 hartrees	
Si11	**DFT**	**B3LYP**	**6-311++G(2df,2p)**
atom#1	atom#2	Bond Order	# electrons (BO x 2)
Si15	O10	1.22528	2.45057
Si15	O11	1.54873	3.09746
Si15	O17	1.52960	3.05920
Si15	O32	1.21256	2.42511
Si-O average		**1.37904**	2.75808
Si-O total		5.51617	**11.03234**
atom#1	atom#2	Bond Order	# electrons (BO x 2)
O10	O11	0.16091	0.32182
O10	O17	0.16721	0.33441
O10	O32	0.24471	0.48943
O11	O17	0.19568	0.39136
O11	O32	0.16689	0.33378
O17	O32	0.15958	0.31916
O-O average		**0.18250**	0.36499
O-O total		1.09498	**2.18996**
grand total bonding		6.61115	**13.22230**
percent Si-O		83.43737	**83.43737**
percent O-O		16.56263	**16.56263**
grand total non-bonding		1.38885	**2.77770**

Si21 J.spartan		-10315.6604769 hartrees	
Si21	**DFT**	**B3LYP**	**6-311++G(2df,2p)**
atom#1	atom#2	Bond Order	# electrons (BO x 2)
Si15	O23	1.04811	2.09621
Si15	O36	1.43141	2.86283
Si15	O39	1.43397	2.86794
Si15	O52	1.04876	2.09753
Si-O average		**1.24056**	2.48113
Si-O total		4.96225	**9.92450**
atom#1	atom#2	Bond Order	# electrons (BO x 2)
O23	O36	0.30801	0.61602
O23	O39	0.34898	0.69795
O23	O52	0.21231	0.42463
O36	O39	0.00000	0.00000
O36	O52	0.34823	0.69645
O39	O52	0.31022	0.62043
O-O average		**0.25462**	0.50925
O-O total		1.52774	**3.05549**
grand total bonding		6.48999	**12.97999**
percent Si-O		76.46002	**76.46002**
percent O-O		23.53998	**23.53998**
grand total non-bonding		1.51001	**3.02001**

Si5 O.spartan		-2660.0193934 hartrees	
Si5	**DFT**	**B3LYP**	**6-311++G(3df,2p)**
atom#1	atom#2	Bond Order	# electrons (BO x 2)
Si15	O23	0.99127	1.98255
Si15	O36	1.12189	2.24378
Si15	O39	1.12248	2.24496
Si15	O52	0.99112	1.98224
Si-O average		**1.05669**	2.11338
Si-O total		4.22676	**8.45352**
atom#1	atom#2	Bond Order	# electrons (BO x 2)
O23	O36	0.00000	0.00000
O23	O39	0.00000	0.00000
O23	O52	0.18688	0.37376
O36	O39	0.02738	0.05476
O36	O52	0.00000	0.00000
O39	O52	0.00000	0.00000
O-O average		**0.03571**	0.07142
O-O total		0.21426	**0.42852**
grand total bonding		4.44102	**8.88205**
percent Si-O		95.17542	**95.17542**
percent O-O		4.82458	**4.82458**
grand total non-bonding		3.55898	**7.11795**

Si11 M.spartan		-5607.3795164 hartrees	
Si11	**DFT**	**B3LYP**	**6-311++G(3df,2p)**
atom#1	atom#2	Bond Order	# electrons (BO x 2)
Si15	O10	0.76216	1.52433
Si15	O11	1.11498	2.22995
Si15	O17	1.11064	2.22129
Si15	O32	0.76096	1.52193
Si-O average		**0.93719**	1.87437
Si-O total		3.74875	**7.49749**
atom#1	atom#2	Bond Order	# electrons (BO x 2)
O10	O11	0.08430	0.16860
O10	O17	0.11275	0.22550
O10	O32	0.26224	0.52448
O11	O17	0.08681	0.17361
O11	O32	0.11375	0.22751
O17	O32	0.08174	0.16349
O-O average		**0.12360**	0.24720
O-O total		0.74159	**1.48319**
grand total bonding		4.49034	**8.98068**
percent Si-O		83.48469	**83.48469**
percent O-O		16.51531	**16.51531**
grand total non-bonding		3.50966	**7.01932**

Si5 H.spartan		-2659.4548595 hartrees	
Si5	**DFT**	**B3LYP**	**aug-cc-pVDZ**
atom#1	atom#2	Bond Order	# electrons (BO x 2)
Si15	O23	-0.35884	-0.71768
Si15	O36	-0.59956	-1.19911
Si15	O39	-0.60149	-1.20297
Si15	O52	-0.35896	-0.71792
Si-O average		**-0.47971**	-0.95942
Si-O total		-1.91885	**-3.83769**
atom#1	atom#2	Bond Order	# electrons (BO x 2)
O23	O36	0.04481	0.08963
O23	O39	0.06507	0.13013
O23	O52	0.00000	0.00000
O36	O39	-0.24846	-0.49692
O36	O52	0.06491	0.12983
O39	O52	0.04492	0.08984
O-O average		**-0.00479**	-0.00958
O-O total		-0.02875	**-0.05750**
grand total bonding		-1.94759	**-3.89519**
percent Si-O		98.52394	**98.52394**
percent O-O		1.47606	**1.47606**
grand total non-bonding		9.94759	**19.89519**

Si11 G.spartan		-5606.2035580 hartrees	
Si11	**DFT**	**B3LYP**	**aug-cc-pVDZ**
atom#1	atom#2	Bond Order	# electrons (BO x 2)
Si15	O10	-0.82009	-1.64018
Si15	O11	0.27987	0.55973
Si15	O17	0.27923	0.55847
Si15	O32	-0.82949	-1.65898
Si-O average		**-0.27262**	-0.54524
Si-O total		-1.09048	**-2.18096**
atom#1	atom#2	Bond Order	# electrons (BO x 2)
O10	O11	-0.32131	-0.64261
O10	O17	0.22085	0.44171
O10	O32	-0.13182	-0.26364
O11	O17	0.70111	1.40223
O11	O32	0.22832	0.45663
O17	O32	-0.32470	-0.64940
O-O average		**0.06208**	0.12415
O-O total		0.37246	**0.74492**
grand total bonding		-0.71802	**-1.43604**
percent Si-O		151.87275	**151.87275**
percent O-O		-51.87275	**-51.87275**
grand total non-bonding		8.71802	**17.43604**

Si21 C.spartan		-10313.6443841 hartrees	
Si21	**DFT**	**B3LYP**	**aug-cc-pVDZ**
atom#1	atom#2	Bond Order	# electrons (BO x 2)
Si15	O23	-0.16933	-0.33866
Si15	O36	0.14144	0.28287
Si15	O39	0.14059	0.28119
Si15	O52	-0.17048	-0.34096
Si-O average		**-0.01445**	-0.02889
Si-O total		-0.05778	**-0.11557**
atom#1	atom#2	Bond Order	# electrons (BO x 2)
O23	O36	0.27609	0.55219
O23	O39	0.46678	0.93355
O23	O52	0.26385	0.52771
O36	O39	0.07370	0.14740
O36	O52	0.46794	0.93587
O39	O52	0.27714	0.55427
O-O average		**0.30425**	0.60850
O-O total		1.82550	**3.65099**
grand total bonding		1.76771	**3.53542**
percent Si-O		-3.26886	**-3.26886**
percent O-O		103.26886	**103.26886**
grand total non-bonding		6.23229	**12.46458**

Si5 L.spartan		-2660.0211570 hartrees	
Si5	**DFT**	**B3LYP**	**aug-cc-pVTZ**
atom#1	atom#2	Bond Order	# electrons (BO x 2)
Si15	O23	0.32689	0.65377
Si15	O36	0.49967	0.99935
Si15	O39	0.50113	1.00227
Si15	O52	0.32590	0.65180
Si-O average		**0.41340**	0.82680
Si-O total		1.65359	**3.30719**
atom#1	atom#2	Bond Order	# electrons (BO x 2)
O23	O36	0.04813	0.09627
O23	O39	0.27194	0.54389
O23	O52	0.35366	0.70731
O36	O39	0.31248	0.62497
O36	O52	0.27258	0.54516
O39	O52	0.04772	0.09544
O-O average		**0.21775**	0.43551
O-O total		1.30652	**2.61304**
grand total bonding		2.96011	**5.92022**
percent Si-O		55.86254	**55.86254**
percent O-O		44.13746	**44.13746**
grand total non-bonding		5.03989	**10.07978**

Si11 C.spartan		-5607.3736739 hartrees	
Si11	**DFT**	**B3LYP**	**aug-cc-pVTZ**
atom#1	atom#2	Bond Order	# electrons (BO x 2)
Si15	O10	0.54215	1.08430
Si15	O11	0.51988	1.03976
Si15	O17	0.51072	1.02145
Si15	O32	0.53954	1.07907
Si-O average		**0.52807**	1.05615
Si-O total		2.11229	**4.22459**
atom#1	atom#2	Bond Order	# electrons (BO x 2)
O10	O11	0.07258	0.14516
O10	O17	0.05440	0.10881
O10	O32	0.07518	0.15036
O11	O17	0.11552	0.23105
O11	O32	0.05274	0.10549
O17	O32	0.07130	0.14261
O-O average		**0.07362**	0.14725
O-O total		0.44174	**0.88347**
grand total bonding		2.55403	**5.10806**
percent Si-O		82.70436	**82.70436**
percent O-O		17.29564	**17.29564**
grand total non-bonding		5.44597	**10.89194**

Si5 R.spartan		-2660.1681231 hartrees	
Si5	**DFT**	**B3LYP**	**aug-cc-pVQZ**
atom#1	atom#2	Bond Order	# electrons (BO x 2)
Si15	O23	0.47163	0.94326
Si15	O36	0.38584	0.77168
Si15	O39	0.38710	0.77420
Si15	O52	0.47000	0.94000
Si-O average		**0.42864**	0.85729
Si-O total		1.71457	**3.42914**
atom#1	atom#2	Bond Order	# electrons (BO x 2)
O23	O36	0.05673	0.11346
O23	O39	0.13559	0.27117
O23	O52	0.15432	0.30864
O36	O39	0.17024	0.34048
O36	O52	0.13528	0.27056
O39	O52	0.05655	0.11310
O-O average		**0.11812**	0.23624
O-O total		0.70871	**1.41741**
grand total bonding		2.42328	**4.84655**
percent Si-O		70.75423	**70.75423**
percent O-O		29.24577	**29.24577**
grand total non-bonding		5.57672	**11.15345**

Si5 J.spartan		-2658.0916747 hartrees	
Si5	**DFT**	**B3LYP**	**DEF2-SVPD**
atom#1	atom#2	Bond Order	# electrons (BO x 2)
Si15	O23	-2.15915	-4.31829
Si15	O36	-3.06274	-6.12549
Si15	O39	-3.06471	-6.12942
Si15	O52	-2.16144	-4.32288
Si-O average		**-2.61201**	-5.22402
Si-O total		-10.44804	**-20.89608**
atom#1	atom#2	Bond Order	# electrons (BO x 2)
O23	O36	1.56548	3.13096
O23	O39	1.57518	3.15036
O23	O52	0.97488	1.94976
O36	O39	2.27818	4.55635
O36	O52	1.57482	3.14965
O39	O52	1.56984	3.13968
O-O average		**1.58973**	3.17946
O-O total		9.53838	**19.07676**
grand total bonding		-0.90966	**-1.81931**
percent Si-O		1148.56889	**1148.56889**
percent O-O		-1048.5689	**-1048.5689**
grand total non-bonding		8.90966	**17.81931**

Si11 H.spartan		-5603.4153081 hartrees	
Si11	**DFT**	**B3LYP**	**DEF2-SVPD**
atom#1	atom#2	Bond Order	# electrons (BO x 2)
Si15	O10	0.81297	1.62594
Si15	O11	0.96354	1.92707
Si15	O17	0.96522	1.93044
Si15	O32	0.81240	1.62480
Si-O average		**0.88853**	1.77706
Si-O total		3.55413	**7.10825**
atom#1	atom#2	Bond Order	# electrons (BO x 2)
O10	O11	0.65871	1.31742
O10	O17	0.45593	0.91185
O10	O32	0.38181	0.76361
O11	O17	1.17218	2.34436
O11	O32	0.45357	0.90714
O17	O32	0.65233	1.30466
O-O average		**0.62909**	1.25817
O-O total		3.77452	**7.54904**
grand total bonding		7.32864	**14.65729**
percent Si-O		48.49635	**48.49635**
percent O-O		51.50365	**51.50365**
grand total non-bonding		0.67136	**1.34271**

Si21 B.spartan		-10308.6537382 hartrees	
Si21	**DFT**	**B3LYP**	**DEF2-SVPD**
atom#1	atom#2	Bond Order	# electrons (BO x 2)
Si15	O23	0.51249	1.02498
Si15	O36	0.49346	0.98693
Si15	O39	0.49504	0.99009
Si15	O52	0.51173	1.02346
Si-O average		**0.50318**	1.00636
Si-O total		2.01273	**4.02545**
atom#1	atom#2	Bond Order	# electrons (BO x 2)
O23	O36	0.00000	0.00000
O23	O39	0.37562	0.75123
O23	O52	0.18477	0.36953
O36	O39	0.30489	0.60978
O36	O52	0.37498	0.74995
O39	O52	0.00000	0.00000
O-O average		**0.20671**	0.41342
O-O total		1.24025	**2.48050**
grand total bonding		3.25298	**6.50595**
percent Si-O		61.87342	**61.87342**
percent O-O		38.12658	**38.12658**
grand total non-bonding		4.74702	**9.49405**

Si5 U.spartan		-2660.0835977 hartrees	
Si5	**DFT**	**B3LYP**	**def2-TZVPD**
atom#1	atom#2	Bond Order	# electrons (BO x 2)
Si15	O23	1.03074	2.06149
Si15	O36	0.56568	1.13135
Si15	O39	0.56678	1.13357
Si15	O52	1.02906	2.05812
Si-O average		**0.79807**	1.59613
Si-O total		3.19227	**6.38453**
atom#1	atom#2	Bond Order	# electrons (BO x 2)
O23	O36	0.00000	0.00000
O23	O39	0.02989	0.05977
O23	O52	-0.04983	-0.09966
O36	O39	0.07747	0.15494
O36	O52	0.02966	0.05932
O39	O52	0.00000	0.00000
O-O average		**0.01453**	0.02906
O-O total		0.08719	**0.17438**
grand total bonding		3.27945	**6.55891**
percent Si-O		97.34138	**97.34138**
percent O-O		2.65862	**2.65862**
grand total non-bonding		4.72055	**9.44109**

Si11 L.spartan		-5607.5080485 hartrees	
Si11	**DFT**	**B3LYP**	**def2-TZVPD**
atom#1	atom#2	Bond Order	# electrons (BO x 2)
Si15	O10	0.59764	1.19529
Si15	O11	0.65719	1.31438
Si15	O17	0.64756	1.29511
Si15	O32	0.60094	1.20189
Si-O average		**0.62583**	1.25167
Si-O total		2.50333	**5.00667**
atom#1	atom#2	Bond Order	# electrons (BO x 2)
O10	O11	0.08982	0.17965
O10	O17	0.08521	0.17043
O10	O32	0.18788	0.37575
O11	O17	0.12500	0.24999
O11	O32	0.08533	0.17067
O17	O32	0.08613	0.17227
O-O average		**0.10990**	0.21979
O-O total		0.65938	**1.31875**
grand total bonding		3.16271	**6.32542**
percent Si-O		79.15154	**79.15154**
percent O-O		20.84846	**20.84846**
grand total non-bonding		4.83729	**9.67458**

Si5 Y.spartan		-2660.2291794 hartrees	
Si5	**DFT**	**B3LYP**	**def2-QZVPD**
atom#1	atom#2	Bond Order	# electrons (BO x 2)
Si15	O23	1.12745	2.25490
Si15	O36	1.18483	2.36967
Si15	O39	1.18752	2.37504
Si15	O52	1.12713	2.25426
Si-O average		**1.15673**	2.31347
Si-O total		4.62694	**9.25387**
atom#1	atom#2	Bond Order	# electrons (BO x 2)
O23	O36	0.00000	0.00000
O23	O39	0.00000	0.00000
O23	O52	0.02923	0.05846
O36	O39	0.00000	0.00000
O36	O52	0.00000	0.00000
O39	O52	0.00000	0.00000
O-O average		**0.00487**	0.00974
O-O total		0.02923	**0.05846**
grand total bonding		4.65617	**9.31233**
percent Si-O		99.37223	**99.37223**
percent O-O		0.62777	**0.62777**
grand total non-bonding		3.34383	**6.68767**

Si5 2O.spartan		-2659.1749154 hartrees	
Si5	**DFT**	**B3LYP**	**6-31G***
atom#1	atom#2	Bond Order	# electrons (BO x 2)
Si15	O23	1.03041	2.06082
Si15	O36	1.00462	2.00923
Si15	O39	1.00579	2.01158
Si15	O52	1.02995	2.05991
Si-O average		**1.01769**	2.03538
Si-O total		4.07077	**8.14153**
atom#1	atom#2	Bond Order	# electrons (BO x 2)
O23	O36	0.00000	0.00000
O23	O39	0.00000	0.00000
O23	O52	0.00000	0.00000
O36	O39	0.00000	0.00000
O36	O52	0.00000	0.00000
O39	O52	0.00000	0.00000
O-O average		**0.00000**	0.00000
O-O total		0.00000	**0.00000**
grand total bonding		4.07077	**8.14153**
percent Si-O		100.00000	**100.00000**
percent O-O		0.00000	**0.00000**
grand total non-bonding		3.92923	**7.85847**

Si11 2F.spartan		-5605.6958025 hartrees	
Si11	**DFT**	**B3LYP**	**6-31G***
atom#1	atom#2	Bond Order	# electrons (BO x 2)
Si15	O10	1.00112	2.00225
Si15	O11	0.97222	1.94444
Si15	O17	0.97010	1.94020
Si15	O32	1.00049	2.00098
Si-O average		**0.98598**	1.97197
Si-O total		3.94394	**7.88787**
atom#1	atom#2	Bond Order	# electrons (BO x 2)
O10	O11	0.00000	0.00000
O10	O17	0.00000	0.00000
O10	O32	0.00000	0.00000
O11	O17	0.00000	0.00000
O11	O32	0.00000	0.00000
O17	O32	0.00000	0.00000
O-O average		**0.00000**	0.00000
O-O total		0.00000	**0.00000**
grand total bonding		3.94394	**7.88787**
percent Si-O		100.00000	**100.00000**
percent O-O		0.00000	**0.00000**
grand total non-bonding		4.05606	**8.11213**

Si21 2F.spartan		-10312.8503897 hartrees	
Si21	**DFT**	**B3LYP**	**6-31G***
atom#1	atom#2	Bond Order	# electrons (BO x 2)
Si15	O23	0.94516	1.89033
Si15	O36	0.97864	1.95728
Si15	O39	0.98001	1.96001
Si15	O52	0.94495	1.88989
Si-O average		**0.96219**	1.92438
Si-O total		3.84876	**7.69751**
atom#1	atom#2	Bond Order	# electrons (BO x 2)
O23	O36	0.00000	0.00000
O23	O39	0.00000	0.00000
O23	O52	0.00000	0.00000
O36	O39	0.00000	0.00000
O36	O52	0.00000	0.00000
O39	O52	0.00000	0.00000
O-O average		**0.00000**	0.00000
O-O total		0.00000	**0.00000**
grand total bonding		3.84876	**7.69751**
percent Si-O		100.00000	**100.00000**
percent O-O		0.00000	**0.00000**
grand total non-bonding		4.15124	**8.30249**

Si5 G.spartan		-2659.2568542 hartrees	
Si5	**DFT**	**B3LYP**	**cc-pVDZ**
atom#1	atom#2	Bond Order	# electrons (BO x 2)
Si15	O23	1.14448	2.28896
Si15	O36	1.11425	2.22850
Si15	O39	1.11538	2.23076
Si15	O52	1.14400	2.28800
Si-O average		**1.12953**	2.25906
Si-O total		4.51811	**9.03622**
atom#1	atom#2	Bond Order	# electrons (BO x 2)
O23	O36	0.00000	0.00000
O23	O39	0.00000	0.00000
O23	O52	0.00000	0.00000
O36	O39	0.00000	0.00000
O36	O52	0.00000	0.00000
O39	O52	0.00000	0.00000
O-O average		**0.00000**	0.00000
O-O total		0.00000	**0.00000**
grand total bonding		4.51811	**9.03622**
percent Si-O		100.00000	**100.00000**
percent O-O		0.00000	**0.00000**
grand total non-bonding		3.48189	**6.96378**

Si11 S.spartan		-5605.8322022 hartrees	
Si11	**DFT**	**B3LYP**	**cc-pVDZ**
atom#1	atom#2	Bond Order	# electrons (BO x 2)
Si15	O10	1.12665	2.25329
Si15	O11	1.09044	2.18089
Si15	O17	1.08840	2.17680
Si15	O32	1.12597	2.25194
Si-O average		**1.10787**	2.21573
Si-O total		4.43146	**8.86292**
atom#1	atom#2	Bond Order	# electrons (BO x 2)
O10	O11	0.00000	0.00000
O10	O17	0.00000	0.00000
O10	O32	0.00000	0.00000
O11	O17	0.00000	0.00000
O11	O32	0.00000	0.00000
O17	O32	0.00000	0.00000
O-O average		**0.00000**	0.00000
O-O total		0.00000	**0.00000**
grand total bonding		4.43146	**8.86292**
percent Si-O		100.00000	**100.00000**
percent O-O		0.00000	**0.00000**
grand total non-bonding		3.56854	**7.13708**

Si21 2E.spartan		-10313.0264632 hartrees	
Si21	**DFT**	**B3LYP**	**cc-pVDZ**
atom#1	atom#2	Bond Order	# electrons (BO x 2)
Si15	O23	1.08710	2.17421
Si15	O36	1.09846	2.19693
Si15	O39	1.09975	2.19950
Si15	O52	1.08689	2.17378
Si-O average		**1.09305**	2.18610
Si-O total		4.37220	**8.74440**
atom#1	atom#2	Bond Order	# electrons (BO x 2)
O23	O36	0.00000	0.00000
O23	O39	0.00000	0.00000
O23	O52	0.00000	0.00000
O36	O39	0.00000	0.00000
O36	O52	0.00000	0.00000
O39	O52	0.00000	0.00000
O-O average		**0.00000**	0.00000
O-O total		0.00000	**0.00000**
grand total bonding		4.37220	**8.74440**
percent Si-O		100.00000	**100.00000**
percent O-O		0.00000	**0.00000**
grand total non-bonding		3.62780	**7.25560**

Si5 K.spartan		-2659.9817894 hartrees	
Si5	**DFT**	**B3LYP**	**cc-pVTZ**
atom#1	atom#2	Bond Order	# electrons (BO x 2)
Si15	O23	1.11054	2.22107
Si15	O36	1.06795	2.13590
Si15	O39	1.06917	2.13834
Si15	O52	1.11004	2.22007
Si-O average		**1.08942**	2.17885
Si-O total		4.35770	**8.71539**
atom#1	atom#2	Bond Order	# electrons (BO x 2)
O23	O36	0.00000	0.00000
O23	O39	0.00000	0.00000
O23	O52	0.00000	0.00000
O36	O39	0.00000	0.00000
O36	O52	0.00000	0.00000
O39	O52	0.00000	0.00000
O-O average		**0.00000**	0.00000
O-O total		0.00000	**0.00000**
grand total bonding		4.35770	**8.71539**
percent Si-O		100.00000	**100.00000**
percent O-O		0.00000	**0.00000**
grand total non-bonding		3.64230	**7.28461**

Si11 X.spartan		-5607.3013631 hartrees	
Si11	**DFT**	**B3LYP**	**cc-pVTZ**
atom#1	atom#2	Bond Order	# electrons (BO x 2)
Si15	O10	1.06284	2.12568
Si15	O11	1.02373	2.04746
Si15	O17	1.02075	2.04149
Si15	O32	1.06221	2.12443
Si-O average		1.04238	2.08476
Si-O total		4.16953	**8.33906**
atom#1	atom#2	Bond Order	# electrons (BO x 2)
O10	O11	0.00000	0.00000
O10	O17	0.00000	0.00000
O10	O32	0.00000	0.00000
O11	O17	0.00000	0.00000
O11	O32	0.00000	0.00000
O17	O32	0.00000	0.00000
O-O average		0.00000	0.00000
O-O total		0.00000	**0.00000**
grand total bonding		4.16953	**8.33906**
percent Si-O		100.00000	**100.00000**
percent O-O		0.00000	**0.00000**
grand total non-bonding		3.83047	**7.66094**

Si5 Q.spartan		-2660.1572389 hartrees	
Si5	**DFT**	**B3LYP**	**cc-pVQZ**
atom#1	atom#2	Bond Order	# electrons (BO x 2)
Si15	O23	1.06732	2.13465
Si15	O36	1.00107	2.00214
Si15	O39	1.06694	2.13387
Si15	O52	1.00301	2.00602
Si-O average		**1.03458**	2.06917
Si-O total		4.13834	**8.27668**
atom#1	atom#2	Bond Order	# electrons (BO x 2)
O23	O36	0.00000	0.00000
O23	O39	0.00000	0.00000
O23	O52	0.00000	0.00000
O36	O39	0.00000	0.00000
O36	O52	0.00000	0.00000
O39	O52	0.00000	0.00000
O-O average		**0.00000**	0.00000
O-O total		0.00000	**0.00000**
grand total bonding		4.13834	**8.27668**
percent Si-O		100.00000	**100.00000**
percent O-O		0.00000	**0.00000**
grand total non-bonding		3.86166	**7.72332**

Si5 I.spartan		-2657.7995967 hartrees	
Si5	**DFT**	**B3LYP**	**DEF2-SV(P)**
atom#1	atom#2	Bond Order	# electrons (BO x 2)
Si15	O23	1.08760	2.17521
Si15	O36	1.06244	2.12487
Si15	O39	1.06366	2.12732
Si15	O52	1.08714	2.17429
Si-O average		**1.07521**	2.15042
Si-O total		4.30084	**8.60169**
atom#1	atom#2	Bond Order	# electrons (BO x 2)
O23	O36	0.00000	0.00000
O23	O39	0.00000	0.00000
O23	O52	0.00000	0.00000
O36	O39	0.00000	0.00000
O36	O52	0.00000	0.00000
O39	O52	0.00000	0.00000
O-O average		**0.00000**	0.00000
O-O total		0.00000	**0.00000**
grand total bonding		4.30084	**8.60169**
percent Si-O		100.00000	**100.00000**
percent O-O		0.00000	**0.00000**
grand total non-bonding		3.69916	**7.39831**

Si11 I.spartan		-5602.8926301 hartrees	
Si11	**DFT**	**B3LYP**	**DEF2-SV(P)**
atom#1	atom#2	Bond Order	# electrons (BO x 2)
Si15	O10	1.07567	2.15135
Si15	O11	1.04321	2.08643
Si15	O17	1.04102	2.08204
Si15	O32	1.07503	2.15005
Si-O average		**1.05873**	2.11747
Si-O total		4.23493	**8.46987**
atom#1	atom#2	Bond Order	# electrons (BO x 2)
O10	O11	0.00000	0.00000
O10	O17	0.00000	0.00000
O10	O32	0.00000	0.00000
O11	O17	0.00000	0.00000
O11	O32	0.00000	0.00000
O17	O32	0.00000	0.00000
O-O average		**0.00000**	0.00000
O-O total		0.00000	**0.00000**
grand total bonding		4.23493	**8.46987**
percent Si-O		100.00000	**100.00000**
percent O-O		0.00000	**0.00000**
grand total non-bonding		3.76507	**7.53013**

Si21 2G.spartan		-10307.8439200 hartrees	
Si21	**DFT**	**B3LYP**	**DEF2-SV(P)**
atom#1	atom#2	Bond Order	# electrons (BO x 2)
Si15	O23	1.04159	2.08318
Si15	O36	1.05547	2.11093
Si15	O39	1.05692	2.11384
Si15	O52	1.04138	2.08275
Si-O average		**1.04884**	2.09768
Si-O total		4.19535	**8.39070**
atom#1	atom#2	Bond Order	# electrons (BO x 2)
O23	O36	0.00000	0.00000
O23	O39	0.00000	0.00000
O23	O52	0.00000	0.00000
O36	O39	0.00000	0.00000
O36	O52	0.00000	0.00000
O39	O52	0.00000	0.00000
O-O average		**0.00000**	0.00000
O-O total		0.00000	**0.00000**
grand total bonding		4.19535	**8.39070**
percent Si-O		100.00000	**100.00000**
percent O-O		0.00000	**0.00000**
grand total non-bonding		3.80465	**7.60930**

Si5 T.spartan		-2660.0674934 hartrees	
Si5	**DFT**	**B3LYP**	**def2-TZVP**
atom#1	atom#2	Bond Order	# electrons (BO x 2)
Si15	O23	1.10429	2.20857
Si15	O36	1.06429	2.12858
Si15	O39	1.06538	2.13075
Si15	O52	1.10387	2.20774
Si-O average		**1.08446**	2.16891
Si-O total		4.33783	**8.67565**
atom#1	atom#2	Bond Order	# electrons (BO x 2)
O23	O36	0.00000	0.00000
O23	O39	0.00000	0.00000
O23	O52	0.00000	0.00000
O36	O39	0.00000	0.00000
O36	O52	0.00000	0.00000
O39	O52	0.00000	0.00000
O-O average		**0.00000**	0.00000
O-O total		0.00000	**0.00000**
grand total bonding		4.33783	**8.67565**
percent Si-O		100.00000	**100.00000**
percent O-O		0.00000	**0.00000**
grand total non-bonding		3.66217	**7.32435**

Si11 Y.spartan		-5607.4791648 hartrees	
Si11	**DFT**	**B3LYP**	**def2-TZVP**
atom#1	atom#2	Bond Order	# electrons (BO x 2)
Si15	O10	1.06617	2.13235
Si15	O11	1.02808	2.05615
Si15	O17	1.02574	2.05148
Si15	O32	1.06611	2.13222
Si-O average		**1.04652**	2.09305
Si-O total		4.18610	**8.37220**
atom#1	atom#2	Bond Order	# electrons (BO x 2)
O10	O11	0.00000	0.00000
O10	O17	0.00000	0.00000
O10	O32	0.00000	0.00000
O11	O17	0.00000	0.00000
O11	O32	0.00000	0.00000
O17	O32	0.00000	0.00000
O-O average		**0.00000**	0.00000
O-O total		0.00000	**0.00000**
grand total bonding		4.18610	**8.37220**
percent Si-O		100.00000	**100.00000**
percent O-O		0.00000	**0.00000**
grand total non-bonding		3.81390	**7.62780**

Si5 W.spartan		-2660.2277465 hartrees	
Si5	**DFT**	**B3LYP**	**def2-QZVP**
atom#1	atom#2	Bond Order	# electrons (BO x 2)
Si15	O23	1.05760	2.11521
Si15	O36	1.01079	2.02157
Si15	O39	1.01248	2.02496
Si15	O52	1.05684	2.11368
Si-O average		**1.03443**	2.06885
Si-O total		4.13771	**8.27541**
atom#1	atom#2	Bond Order	# electrons (BO x 2)
O23	O36	0.00000	0.00000
O23	O39	0.00000	0.00000
O23	O52	-0.03436	-0.06873
O36	O39	0.00000	0.00000
O36	O52	0.00000	0.00000
O39	O52	0.00000	0.00000
O-O average		**-0.00573**	-0.01145
O-O total		-0.03436	**-0.06873**
grand total bonding		4.10334	**8.20669**
percent Si-O		100.83743	**100.83743**
percent O-O		-0.83743	**-0.83743**
grand total non-bonding		3.89666	**7.79331**

Table S32. Average computed **Wiberg** and **Mayer** bond orders, along with 3-center 2-electron (**3c2e**) and 4-center 2-electron (**4c2e**) bond indices for the central SiO_4 unit within various silica clusters. Four bond order values are averaged for Si-O and six bond order values are averaged for O-O. Also included are the silicon (Si_{AV}) and oxygen (O_{AV}) atomic valencies computed for the central SiO_4 unit (four oxygen values averaged) according to the **Mayer** (*AOMix*) and Mulliken (*Spartan*) methods. *Spartan* Output files (Si*xx*.txt via B3LYP/6-311++G**) were processed with the *AOMix* software package (via Windows XP, running on an iMac with the Parallels Desktop 17 for Mac emulator) along with fragment files (Si*xx*frag.txt) that instruct *AOMix* to treat each atom as a compilation of all corresponding basis functions (22 for oxygen and 30 for silicon at this level of theory).[5] The *Spartan 18* Output files were parsed to mimic compatible *Spartan 08* Output files by only including the number of basis functions, the number of electrons, the atomic Cartesian coordinates, the overlap matrix, and the molecular (atomic) orbital coefficients. Additionally, the *AOMix* code was rewritten by author Serge Gorelsky to accommodate large *Spartan* Output files with more than 999 basis functions (*Si11* and greater).

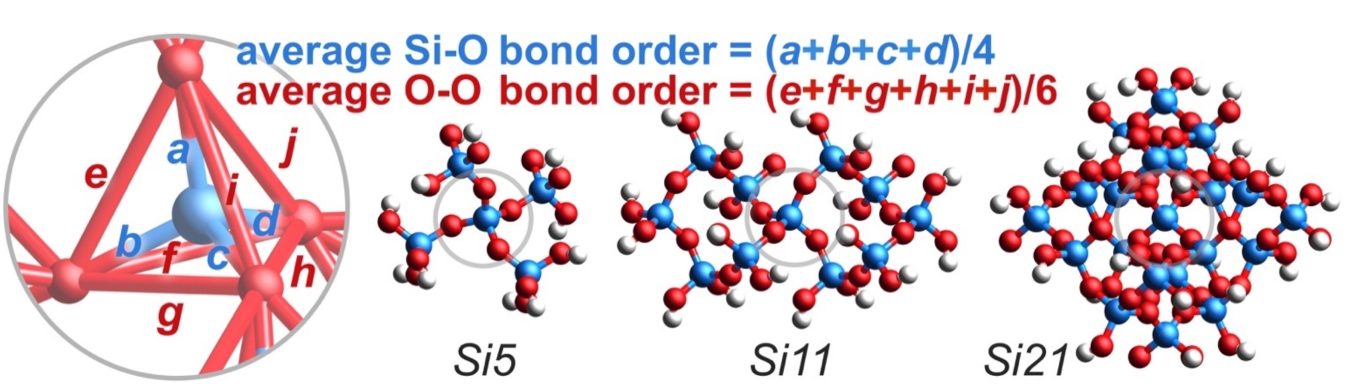

cluster	Wiberg bond order (AOMix)		Mayer bond order (AOMix)		Mulliken bond order (Spartan)		3c2e bond (AOMix)	4c2e bond (AOMix)	3c2e bond (AOMix)	4c2e bond (AOMix)	Mayer atomic valence		Mulliken atomic valence (Spartan)	
	Si-O	O-O	Si-O	O-O	Si-O	O-O	max I_{OOO}	max I_{OOOO}	max I_{OSiO}	max I_{OSiOO}	Si_{AV}	O_{AV}	Si_{AV}	O_{AV}
Si1	1.024	0.046	1.024	0.046	1.027	0.046	0.053	0.019	< 0.01	< 0.01	4.328	2.056	4.327	2.049
Si2	0.986	0.087	0.986	0.087	0.984	0.091	0.078	0.028	< 0.01	< 0.01	4.160	2.229	4.159	2.220
Si3	0.912	0.145	0.912	0.145	0.914	0.145	0.119	0.044	< 0.01	< 0.01	3.856	2.402	3.855	2.393
Si4	0.838	0.204	0.838	0.204	0.841	0.204	0.164	0.065	< 0.01	< 0.01	3.480	2.638	3.478	2.627
Si5	0.739	0.276	0.739	0.276	0.741	0.278	0.237	0.116	< 0.01	< 0.01	2.994	2.936	2.989	2.925
Si8	0.780	0.310	0.780	0.310	0.783	0.311	0.281	0.155	< 0.01	< 0.01	3.296	3.153	3.295	3.142
Si11	0.802	0.361	0.802	0.361	0.805	0.363	**0.319**	**0.209**	< 0.01	< 0.01	4.112	3.376	4.106	3.365
Si15	0.819	0.380	0.819	0.380	0.823	0.383	**0.320**	**0.123**	< 0.01	< 0.01	3.771	3.737	3.762	3.726
Si18	0.806	0.406	0.806	0.406	0.810	0.409	**0.383**	**0.143**	< 0.01	**0.023**	3.842	3.811	3.832	3.801
Si21	0.672	0.459	0.672	0.459	0.677	0.461	**0.321**	**0.130**	< 0.01	< 0.01	3.572	4.099	3.566	4.090
Si25	0.629	0.455	0.629	0.455	0.635	0.457	**0.349**	**0.169**	< 0.01	**0.038**	3.342	4.172	3.335	4.164
Si29	0.653	0.478	0.653	0.478	0.657	0.481	**0.347**	**0.110**	< 0.01	< 0.01	**3.148**	**4.361**	**3.135**	**4.350**
Si35	0.585	0.470	0.585	0.470	0.593	0.472	**0.319**	**0.120**	< 0.01	**0.141**	3.255	4.187	3.253	4.176
Si6 (ring)		0.223		0.223		0.224								
AVE 6 O-O														

Table S33. The compilation of *AOmix* software output data for the silica clusters summarized in Table S32.

Si1.spartan		-593.1208008	hartrees		
Si1	DFT	B3LYP	6-311++G**		
		Wiberg	= Mayer		
atom#1	atom#2	Bond Order	# e (BO x 2)	AOMix Atoms/Fragments	
Si2	O20	1.041	2.08200	5	1
Si2	O19	1.006	2.01200	5	2
Si2	O12	1.008	2.01600	5	3
Si2	O11	1.042	2.08400	5	4
Si-O average		**1.02425**	2.04850		
Si-O total		4.09700	**8.19400**		
atom#1	atom#2	Bond Order	# e (BO x 2)		
O20	O19	0.054	0.10800	1	2
O20	O12	0.037	0.07400	1	3
O20	O11	0.036	0.07200	1	4
O19	O12	0.056	0.11200	2	3
O19	O11	0.037	0.07400	2	4
O12	O11	0.054	0.10800	3	4
O-O average		**0.04567**	0.09133		
O-O total		0.27400	**0.54800**		
grand total bonding		4.37100	**8.74200**		
percent Si-O		93.73141	**93.73141**		
percent O-O		6.26859	**6.26859**		
grand total non-bonding		3.62900	**7.25800**		

(AOMix) A	B	Wiberg (P*S)(P*S)	Mayer bond orders B(AB)	B(alpha)	B(beta)
1	2	0.054	0.054	0.027	0.027
1	3	0.037	0.037	0.019	0.019
1	4	0.036	0.036	0.018	0.018
1	5	1.041	1.041	0.521	0.521
2	3	0.056	0.056	0.028	0.028
2	4	0.037	0.037	0.019	0.019
2	5	1.006	1.006	0.503	0.503
3	4	0.054	0.054	0.027	0.027
3	5	1.008	1.008	0.504	0.504
4	5	1.042	1.042	0.521	0.521

(AOMix)	A	B	C	3-center BO	O-O-O-O (AOMix)	
O-O-O	1	2	3	0.047	4-CENTER BO index	
O-O-O	1	3	2	0.059	B(ABCD)=	**0.019**
O-O-O	2	1	3	0.059		
O-O-O	2	3	1	0.047		
O-O-O	3	1	2	0.047		
O-O-O	3	2	1	0.059		
Average				**0.053**		

Fragment			Mayer Total Valences (AOMix)	Mulliken Atomic Valences	(Spartan)
O	1	:	2.052	O20	2.044732
O	2	:	2.059	O19	2.050958
O	3	:	2.06	O12	2.053059
O	4	:	2.052	O11	2.045672
Si	5	:	**4.328**	Si2	**4.32682**
O average			**2.056**		**2.04861**

Si2.spartan		-1109.794721	hartrees		
Si2	**DFT**	**B3LYP**	**6-311++G****		
		Wiberg	= Mayer		
atom#1	atom#2	Bond Order	# e (BO x 2)	AOMix Atoms/Fragments	
Si2	O20	0.864	1.72800	5	1
Si2	O19	1.01	2.02000	5	2
Si2	O12	1.011	2.02200	5	3
Si2	O11	1.06	2.12000	5	4
Si-O average		**0.98625**	1.97250		
Si-O total		3.94500	**7.89000**		
atom#1	atom#2	Bond Order	# e (BO x 2)		
O20	O19	0.118	0.23600	1	2
O20	O12	0.166	0.33200	1	3
O20	O11	0.074	0.14800	1	4
O19	O12	0.068	0.13600	2	3
O19	O11	0.033	0.06600	2	4
O12	O11	0.062	0.12400	3	4
O-O average		**0.08683**	0.17367		
O-O total		0.52100	**1.04200**		
grand total bonding		4.46600	**8.93200**		
percent Si-O		88.33408	**88.33408**		
percent O-O		11.66592	**11.66592**		
grand total non-bonding		3.53400	**7.06800**		

(AOMix) A	B	Wiberg (P*S)(P*S)	Mayer bond orders B(AB)	B(alpha)	B(beta)
1	2	0.118	0.118	0.059	0.059
1	3	0.166	0.166	0.083	0.083
1	4	0.074	0.074	0.037	0.037
1	5	0.864	0.864	0.432	0.432
2	3	0.068	0.068	0.034	0.034
2	4	0.033	0.033	0.017	0.017
2	5	1.01	1.01	0.505	0.505
3	4	0.062	0.062	0.031	0.031
3	5	1.011	1.011	0.506	0.506
4	5	1.06	1.06	0.53	0.53

(AOMix)	A	B	C	3-center BO	O-O-O-O	(AOMix)
O-O-O	1	2	3	0.079	4-CENTER BO index	
O-O-O	1	3	2	0.077	B(ABCD)=	**0.028**
O-O-O	2	1	3	0.077		
O-O-O	2	3	1	0.079		
O-O-O	3	1	2	0.079		
O-O-O	3	2	1	0.077		
Average				**0.078**		

Fragment			Mayer Total Valences (AOMix)	Mulliken Atomic Valences	(Spartan)
O	1	:	2.469	O20	2.459715
O	2	:	2.169	O19	2.162165
O	3	:	2.19	O12	2.181505
O	4	:	2.087	O11	2.078491
Si	5	:	**4.16**	Si2	**4.159067**
O average			**2.229**		**2.22047**

Si3.spartan		-1626.4690133	hartrees		
Si3	**DFT**	**B3LYP**	**6-311++G****		
		Wiberg	= Mayer		
atom#1	atom#2	Bond Order	# e (BO x 2)	AOMix Atoms/Fragments	
Si11	O5	1.052	2.10400	5	1
Si11	O6	0.92	1.84000	5	2
Si11	O19	0.852	1.70400	5	3
Si11	O24	0.823	1.64600	5	4
Si-O average		**0.91175**	1.82350		
Si-O total		3.64700	**7.29400**		
atom#1	atom#2	Bond Order	# e (BO x 2)		
O5	O6	0.077	0.15400	1	2
O5	O19	0.079	0.15800	1	3
O5	O24	0.212	0.42400	1	4
O6	O19	0.153	0.30600	2	3
O6	O24	0.137	0.27400	2	4
O19	O24	0.211	0.42200	3	4
O-O average		**0.14483**	0.28967		
O-O total		0.86900	**1.73800**		
grand total bonding		4.51600	**9.03200**		
percent Si-O		80.75731	**80.75731**		
percent O-O		19.24269	**19.24269**		
grand total non-bonding		3.48400	**6.96800**		

(AOMix) A	B	Wiberg (P*S)(P*S)	Mayer bond orders B(AB)	B(alpha)	B(beta)
1	2	0.077	0.077	0.038	0.038
1	3	0.079	0.079	0.04	0.04
1	4	0.212	0.212	0.106	0.106
1	5	1.052	1.052	0.526	0.526
2	3	0.153	0.153	0.076	0.076
2	4	0.137	0.137	0.068	0.068
2	5	0.92	0.92	0.46	0.46
3	4	0.211	0.211	0.105	0.105
3	5	0.852	0.852	0.426	0.426
4	5	0.823	0.823	0.412	0.412

(AOMix)	A	B	C	3-center BO	O-O-O-O	(AOMix)
O-O-O	2	3	4	0.11	4-CENTER BO index	
O-O-O	2	4	3	0.128	B(ABCD)=	**0.044**
O-O-O	3	2	4	0.128		
O-O-O	3	4	2	0.11		
O-O-O	4	2	3	0.11		
O-O-O	4	3	2	0.128		
Average				**0.119**		

Fragment			Mayer Total Valences (AOMix)	Mulliken Atomic Valences	(Spartan)
O	1	:	2.243	O5	2.233369
O	2	:	2.209	O6	2.201431
O	3	:	2.577	O19	2.568629
O	4	:	2.578	O24	2.566725
Si	5	:	**3.856**	Si11	**3.854945**
O average			**2.402**		**2.39254**

S4.spartan		-2143.1403630	hartrees		
Si4	**DFT**	**B3LYP**	**6-311++G****		
		Wiberg	= Mayer		
atom#1	atom#2	Bond Order	# e (BO x 2)	AOMix Atoms/Fragments	
Si15	O23	0.767	1.53400	5	1
Si15	O36	0.86	1.72000	5	2
Si15	O52	0.844	1.68800	5	3
Si15	O39	0.882	1.76400	5	4
Si-O average		**0.83825**	1.67650		
Si-O total		3.35300	**6.70600**		
atom#1	atom#2	Bond Order	# e (BO x 2)		
O23	O36	0.258	0.51600	1	2
O23	O52	0.184	0.36800	1	3
O23	O39	0.209	0.41800	1	4
O36	O52	0.233	0.46600	2	3
O36	O39	0.158	0.31600	2	4
O52	O39	0.18	0.36000	3	4
O-O average		**0.20367**	0.40733		
O-O total		1.22200	**2.44400**		
grand total bonding		4.57500	**9.15000**		
percent Si-O		73.28962	**73.28962**		
percent O-O		26.71038	**26.71038**		
grand total non-bonding		3.42500	**6.85000**		

(AOMix) A	B	Wiberg (P*S)(P*S)	Mayer bond orders B(AB)	B(alpha)	B(beta)
1	2	0.258	0.258	0.129	0.129
1	3	0.184	0.184	0.092	0.092
1	4	0.209	0.209	0.104	0.104
1	5	0.767	0.767	0.384	0.384
2	3	0.233	0.233	0.116	0.116
2	4	0.158	0.158	0.079	0.079
2	5	0.86	0.86	0.43	0.43
3	4	0.18	0.18	0.09	0.09
3	5	0.844	0.844	0.422	0.422
4	5	0.882	0.882	0.441	0.441

(AOMix)	A	B	C	3-center BO	O-O-O-O	(AOMix)
O-O-O	1	2	4	0.141	4-CENTER BO index	
O-O-O	1	4	2	0.187	B(ABCD)=	**0.065**
O-O-O	2	1	4	0.187		
O-O-O	2	4	1	0.141		
O-O-O	4	1	2	0.141		
O-O-O	4	2	1	0.187		
Average				**0.164**		

Fragment			Mayer Total Valences (AOMix)	Mulliken Atomic Valences	(Spartan)
O	1	:	2.79	O23	2.779768
O	2	:	2.74	O36	2.729262
O	3	:	2.311	O52	2.30009
O	4	:	2.71	O39	2.700461
Si	5	:	**3.48**	Si15	**3.47782**
O average			**2.638**		**2.62740**

Si5.spartan		-2659.81157	hartrees		
Si5	DFT	B3LYP	6-311++G**		
		Wiberg	= Mayer		
atom#1	atom#2	Bond Order	# e (BO x 2)	AOMix Atoms/Fragments	
Si15	O23	0.656	1.31200	17	1
Si15	O36	0.822	1.64400	17	2
Si15	O39	0.822	1.64400	17	4
Si15	O52	0.656	1.31200	17	3
Si-O average		**0.73900**	1.47800		
Si-O total		2.95600	**5.91200**		
atom#1	atom#2	Bond Order	# e (BO x 2)		
O23	O36	0.294	0.58800	1	2
O23	O39	0.29	0.58000	1	4
O23	O52	0.272	0.54400	1	3
O36	O39	0.217	0.43400	2	4
O36	O52	0.289	0.57800	2	3
O39	O52	0.295	0.59000	4	3
O-O average		**0.27617**	0.55233		
O-O total		1.65700	**3.31400**		
grand total bonding		4.61300	**9.22600**		
percent Si-O		64.07977	**64.07977**		
percent O-O		35.92023	**35.92023**		
grand total non-bonding		3.38700	**6.77400**		

(AOMix) A	B	Wiberg (P*S)(P*S)	Mayer bond orders B(AB)	B(alpha)	B(beta)
1	2	0.294	0.294	0.147	0.147
1	3	0.272	0.272	0.136	0.136
1	4	0.29	0.29	0.145	0.145
1	17	0.656	0.656	0.328	0.328
2	3	0.289	0.289	0.145	0.145
2	4	0.217	0.217	0.109	0.109
2	17	0.822	0.822	0.411	0.411
3	4	0.295	0.295	0.147	0.147
3	17	0.656	0.656	0.328	0.328
4	17	0.822	0.822	0.411	0.411

(AOMix)	A	B	C	3-center BO	O-O-O-O	(AOMix)
O-O-O	1	3	4	0.206	4-CENTER BO index	
O-O-O	1	4	3	0.267	B(ABCD)=	**0.116**
O-O-O	3	1	4	0.267		
O-O-O	3	4	1	0.206		
O-O-O	4	1	3	0.206		
O-O-O	4	3	1	0.267		
Average				**0.2365**		

Fragment			Mayer Total Valences (AOMix)	Mulliken Atomic Valences	(Spartan)
O	1	:	2.969	O23	2.957572
O	2	:	2.901	O36	2.890876
O	3	:	2.97	O52	2.9588
O	4	:	2.902	O39	2.891583
Si	17	:	**2.994**	Si15	**2.989127**
O average			**2.936**		**2.92471**

Si8.spartan		-4133.3797225	hartrees	AOMix Atoms/Fragments	
Si8	DFT	B3LYP	6-311++G**		
		Wiberg	= Mayer		
atom#1	atom#2	Bond Order	# e (BO x 2)		
Si4	O10	0.667	1.33400	5	1
Si4	O11	0.907	1.81400	5	2
Si4	O17	0.919	1.83800	5	3
Si4	O32	0.625	1.25000	5	4
Si-O average		**0.77950**	1.55900		
Si-O total		3.11800	**6.23600**		
atom#1	atom#2	Bond Order	# e (BO x 2)		
O10	O11	0.35	0.70000	1	2
O10	O17	0.308	0.61600	1	3
O10	O32	0.346	0.69200	1	4
O11	O17	0.199	0.39800	2	3
O11	O32	0.334	0.66800	2	4
O17	O32	0.323	0.64600	3	4
O-O average		**0.31000**	0.62000		
O-O total		1.86000	**3.72000**		
grand total bonding		4.97800	**9.95600**		
percent Si-O		62.63560	**62.63560**		
percent O-O		37.36440	**37.36440**		
grand total non-bonding		3.02200	**6.04400**		

(AOMix) A	B	Wiberg (P*S)(P*S)	Mayer bond orders B(AB)	B(alpha)	B(beta)
1	2	0.35	0.35	0.175	0.175
1	3	0.308	0.308	0.154	0.154
1	4	0.346	0.346	0.173	0.173
1	5	0.667	0.667	0.334	0.334
2	3	0.199	0.199	0.099	0.099
2	4	0.334	0.334	0.167	0.167
2	5	0.907	0.907	0.454	0.454
3	4	0.323	0.323	0.162	0.162
3	5	0.919	0.919	0.46	0.46
4	5	0.625	0.625	0.312	0.312

(AOMix)	A	B	C	3-center BO	O-O-O-O	(AOMix)
O-O-O	1	3	4	0.24	4-CENTER BO index	
O-O-O	1	4	3	0.322	B(ABCD)=	**0.155**
O-O-O	3	1	4	0.322		
O-O-O	3	4	1	0.24		
O-O-O	4	1	3	0.24		
O-O-O	4	3	1	0.322		
Average				**0.281**		

Fragment			Mayer Total Valences (AOMix)	Mulliken Atomic Valences	(Spartan)
O	1	:	3.17	O10	3.158501
O	2	:	3.065	O11	3.055495
O	3	:	3.197	O17	3.187154
O	4	:	3.181	O32	3.168389
Si	5	:	**3.296**	Si4	**3.294649**
O average			**3.153**		**3.14238**

Si11.spartan		-5606.9472951	hartrees	AOMix Atoms/Fragments	
Si11	**DFT**	**B3LYP**	**6-311++G****		
		Wiberg	= Mayer		
atom#1	atom#2	Bond Order	# e (BO x 2)		
Si4	O10	0.63300	1.26600	5	1
Si4	O11	0.97400	1.94800	5	2
Si4	O17	0.96700	1.93400	5	3
Si4	O32	0.63300	1.26600	5	4
Si-O average		**0.80175**	1.60350		
Si-O total		3.20700	**6.41400**		
atom#1	atom#2	Bond Order	# e (BO x 2)		
O10	O11	0.41400	0.82800	1	2
O10	O17	0.35300	0.70600	1	3
O10	O32	0.40000	0.80000	1	4
O11	O17	0.24100	0.48200	2	3
O11	O32	0.35300	0.70600	2	4
O17	O32	0.40700	0.81400	3	4
O-O average		**0.36133**	0.72267		
O-O total		2.16800	**4.33600**		
grand total bonding		5.37500	**10.75000**		
percent Si-O		59.66512	**59.66512**		
percent O-O		40.33488	**40.33488**		
grand total non-bonding		2.62500	**5.25000**		

(AOMix) A	B	Wiberg (P*S)(P*S)	Mayer bond orders B(AB)	B(alpha)	B(beta)
1	2	0.414	0.414	0.207	0.207
1	3	0.353	0.353	0.176	0.176
1	4	0.4	0.4	0.2	0.2
1	5	0.633	0.633	0.316	0.316
2	3	0.241	0.241	0.12	0.12
2	4	0.353	0.353	0.176	0.176
2	5	0.974	0.974	0.487	0.487
3	4	0.407	0.407	0.203	0.203
3	5	0.967	0.967	0.484	0.484
4	5	0.633	0.633	0.317	0.317

(AOMix)	A	B	C	3-center BO
O-O-O	1	2	4	0.365
O-O-O	1	4	2	0.272
O-O-O	2	1	4	0.272
O-O-O	2	4	1	0.365
O-O-O	4	1	2	0.365
O-O-O	4	2	1	0.272
Average				**0.3185**

O-O-O-O	(AOMix)
4-CENTER BO index	
B(ABCD)=	**0.209**

Fragment			Mayer Total Valences (AOMix)	Mulliken Atomic Valences	(Spartan)
O	1	:	3.382	O10	3.369197
O	2	:	3.378	O11	3.368568
O	3	:	3.368	O17	3.356901
O	4	:	3.376	O32	3.364964
Si	5	:	**4.112**	Si4	**4.105702**
O average			**3.376**		**3.36491**

Si15.spartan		-7520.743929	hartrees	AOMix Atoms/Fragments	
Si15	DFT	B3LYP	6-311++G**		
		Wiberg	= Mayer		
atom#1	atom#2	Bond Order	# e (BO x 2)		
Si15	O23	0.786	1.57200	5	1
Si15	O36	0.949	1.89800	5	2
Si15	O39	0.862	1.72400	5	4
Si15	O52	0.679	1.35800	5	3
Si-O average		**0.81900**	1.63800		
Si-O total		3.27600	**6.55200**		
atom#1	atom#2	Bond Order	# e (BO x 2)		
O23	O36	0.421	0.84200	1	2
O23	O39	0.472	0.94400	1	4
O23	O52	0.379	0.75800	1	3
O36	O39	0.263	0.52600	2	4
O36	O52	0.367	0.73400	2	3
O39	O52	0.377	0.75400	4	3
O-O average		**0.37983**	0.75967		
O-O total		2.27900	**4.55800**		
grand total bonding		5.55500	**11.11000**		
percent Si-O		58.97390	**58.97390**		
percent O-O		41.02610	**41.02610**		
grand total non-bonding		2.44500	**4.89000**		

(AOMix) A	B	Wiberg (P*S)(P*S)	Mayer bond orders B(AB)	B(alpha)	B(beta)
1	2	0.421	0.421	0.211	0.211
1	3	0.379	0.379	0.189	0.189
1	4	0.472	0.472	0.236	0.236
1	5	0.786	0.786	0.393	0.393
2	3	0.367	0.367	0.183	0.183
2	4	0.263	0.263	0.132	0.132
2	5	0.949	0.949	0.475	0.475
3	4	0.377	0.377	0.188	0.188
3	5	0.679	0.679	0.34	0.34
4	5	0.862	0.862	0.431	0.431

(AOMix)	A	B	C	3-center BO	O-O-O-O (AOMix)	
O-O-O	1	3	4	0.264	4-CENTER BO index	
O-O-O	1	4	3	0.376	B(ABCD)=	**0.123**
O-O-O	3	1	4	0.376		
O-O-O	3	4	1	0.264		
O-O-O	4	1	3	0.264		
O-O-O	4	3	1	0.376		
Average				**0.320**		

Fragment			Mayer Total Valences (AOMix)	Mulliken Atomic Valences	(Spartan)
O	1	:	3.846	O23	3.83456
O	2	:	3.701	O36	3.690848
O	3	:	3.623	O52	3.612517
O	4	:	3.777	O39	3.767936
Si	5	:	**3.771**	Si15	**3.761655**
O average			**3.737**		**3.72647**

Si18.spartan		-8994.3151280	hartrees	AOMix Atoms/Fragments	
Si18	**DFT**	**B3LYP**	**6-311++G****		
		Wiberg	= Mayer		
atom#1	atom#2	Bond Order	# e (BO x 2)		
Si30	O85	0.68900	1.37800	5	1
Si30	O86	0.96700	1.93400	5	2
Si30	O88	0.94700	1.89400	5	3
Si30	O89	0.62100	1.24200	5	4
Si-O average		**0.80600**	1.61200		
Si-O total		3.22400	**6.44800**		
atom#1	atom#2	Bond Order	# e (BO x 2)		
O85	O86	0.32200	0.64400	1	2
O85	O88	0.55700	1.11400	1	3
O85	O89	0.46000	0.92000	1	4
O86	O88	0.38000	0.76000	2	3
O86	O89	0.32900	0.65800	2	4
O88	O89	0.38900	0.77800	3	4
O-O average		**0.40617**	0.81233		
O-O total		2.43700	**4.87400**		
grand total bonding		5.66100	**11.32200**		
percent Si-O		56.95107	**56.95107**		
percent O-O		43.04893	**43.04893**		
grand total non-bonding		2.33900	**4.67800**		

(AOMix) A	B	Wiberg (P*S)(P*S)	Mayer bond orders B(AB)	B(alpha)	B(beta)
1	2	0.322	0.322	0.161	0.161
1	3	0.557	0.557	0.279	0.279
1	4	0.46	0.46	0.23	0.23
1	5	0.689	0.689	0.345	0.345
2	3	0.38	0.38	0.19	0.19
2	4	0.329	0.329	0.164	0.164
2	5	0.967	0.967	0.483	0.483
3	4	0.389	0.389	0.195	0.195
3	5	0.947	0.947	0.474	0.474
4	5	0.621	0.621	0.31	0.31

(AOMix)	A	B	C	3-center BO	O-O-O-O (AOMix)	
O-O-O	1	3	4	0.322	4-CENTER BO index	
O-O-O	1	4	3	0.444	B(ABCD)=	**0.143**
O-O-O	3	1	4	0.444		
O-O-O	3	4	1	0.322		
O-O-O	4	1	3	0.322	O-Si-O-O (AOMix)	
O-O-O	4	3	1	0.444	4-CENTER BO index	
Average				**0.383**	B(ABCD)=	**0.023**

Fragment			Mayer Total Valences (AOMix)	Mulliken Atomic Valences	(Spartan)
O	1	:	4.033	O85	4.024338
O	2	:	3.649	O86	3.639291
O	3	:	3.957	O88	3.945646
O	4	:	3.606	O89	3.592715
Si	5	:	**3.842**	Si30	**3.831976**
O average			**3.811**		**3.80050**

S21.spartan		-10315.0041005	hartrees		
Si21	**DFT**	**B3LYP**	**6-311++G****		
		Wiberg	= Mayer		
atom#1	atom#2	Bond Order	# e (BO x 2)	AOMix Atoms/Fragments	
Si15	O23	0.709	1.41800	5	1
Si15	O36	0.635	1.27000	5	2
Si15	O52	0.709	1.41800	5	3
Si15	O39	0.636	1.27200	5	4
Si-O average		**0.67225**	1.34450		
Si-O total		2.68900	**5.37800**		
atom#1	atom#2	Bond Order	# e (BO x 2)		
O23	O36	0.604	1.20800	1	2
O23	O52	0.357	0.71400	1	3
O23	O39	0.414	0.82800	1	4
O36	O52	0.412	0.82400	2	3
O36	O39	0.359	0.71800	2	4
O52	O39	0.605	1.21000	3	4
O-O average		**0.45850**	0.91700		
O-O total		2.75100	**5.50200**		
grand total bonding		5.44000	**10.88000**		
percent Si-O		49.43015	**49.43015**		
percent O-O		50.56985	**50.56985**		
grand total non-bonding		2.56000	**5.12000**		

(AOMix)		Wiberg	Mayer bond orders		
A	B	(P*S)(P*S)	B(AB)	B(alpha)	B(beta)
1	2	0.604	0.604	0.302	0.302
1	3	0.357	0.357	0.178	0.178
1	4	0.414	0.414	0.207	0.207
1	5	0.709	0.709	0.355	0.355
2	3	0.412	0.412	0.206	0.206
2	4	0.359	0.359	0.18	0.18
2	5	0.635	0.635	0.318	0.318
3	4	0.605	0.605	0.302	0.302
3	5	0.709	0.709	0.354	0.354
4	5	0.636	0.636	0.318	0.318

(AOMix)	A	B	C	3-center BO	O-O-O-O	(AOMix)
O-O-O	1	3	4	0.271	4-CENTER BO index	
O-O-O	1	4	3	0.371	B(ABCD)=	**0.130**
O-O-O	3	1	4	0.371		
O-O-O	3	4	1	0.271		
O-O-O	4	1	3	0.271		
O-O-O	4	3	1	0.371		
Average				**0.321**		

Fragment			Mayer Total Valences (AOMix)	Mulliken Atomic Valences	(Spartan)
O	1	:	4.125	O23	4.115854
O	2	:	4.071	O36	4.063203
O	3	:	4.127	O52	4.117474
O	4	:	4.072	O39	4.064201
Si	5	:	**3.572**	Si15	**3.565919**
O average			**4.099**		**4.09018**

Si25.spartan		-12228.80179	hartrees	AOMix Atoms/Fragments	
Si25	DFT	B3LYP	6-311++G**		
		Wiberg	= Mayer		
atom#1	atom#2	Bond Order	# e (BO x 2)		
Si15	O31	0.647	1.29400	5	1
Si15	O44	0.697	1.39400	5	2
Si15	O47	0.569	1.13800	5	3
Si15	O63	0.603	1.20600	5	4
Si-O average		**0.62900**	1.25800		
Si-O total		2.51600	**5.03200**		
atom#1	atom#2	Bond Order	# e (BO x 2)		
O31	O44	0.413	0.82600	1	2
O31	O47	0.513	1.02600	1	3
O31	O63	0.422	0.84400	1	4
O44	O47	0.427	0.85400	2	3
O44	O63	0.592	1.18400	2	4
O47	O63	0.365	0.73000	3	4
O-O average		**0.45533**	0.91067		
O-O total		2.73200	**5.46400**		
grand total bonding		5.24800	**10.49600**		
percent Si-O		47.94207	**47.94207**		
percent O-O		52.05793	**52.05793**		
grand total non-bonding		2.75200	**5.50400**		

(AOMix) A	B	Wiberg (P*S)(P*S)	Mayer bond orders B(AB)	B(alpha)	B(beta)
1	2	0.413	0.413	0.206	0.206
1	3	0.513	0.513	0.256	0.256
1	4	0.422	0.422	0.211	0.211
1	5	0.647	0.647	0.324	0.324
2	3	0.427	0.427	0.213	0.213
2	4	0.592	0.592	0.296	0.296
2	5	0.697	0.697	0.348	0.348
3	4	0.365	0.365	0.183	0.183
3	5	0.569	0.569	0.285	0.285
4	5	0.603	0.603	0.302	0.302

(AOMix)	A	B	C	3-center BO	O-O-O-O (AOMix)	
O-O-O	1	2	4	0.281	4-CENTER BO index	
O-O-O	1	4	2	0.417	B(ABCD)=	**0.169**
O-O-O	2	1	4	0.417		
O-O-O	2	4	1	0.281		
O-O-O	4	1	2	0.281	O-Si-O-O (AOMix)	
O-O-O	4	2	1	0.417	4-CENTER BO index	
Average				**0.349**	B(ABCD)=	**0.038**

Fragment			Mayer Total Valences (AOMix)	Mulliken Atomic Valences (Spartan)	
O	1	:	4.289	O31	4.278723
O	2	:	4.165	O44	4.155614
O	3	:	3.981	O47	3.973668
O	4	:	4.254	O63	4.246048
Si	5	:	**3.342**	Si15	**3.334627**
O average			**4.172**		**4.16351**

Si29.spartan		-14142.58951	hartrees		
Si29	DFT	B3LYP	6-311++G**		
		Wiberg	= Mayer		
atom#1	atom#2	Bond Order	# e (BO x 2)	AOMix Atoms/Fragments	
Si15	O23	0.63	1.26000	5	1
Si15	O36	0.675	1.35000	5	2
Si15	O39	0.676	1.35200	5	4
Si15	O52	0.629	1.25800	5	3
Si-O average		**0.65250**	1.30500		
Si-O total		2.61000	**5.22000**		
atom#1	atom#2	Bond Order	# e (BO x 2)		
O23	O36	0.623	1.24600	1	2
O23	O39	0.43	0.86000	1	4
O23	O52	0.383	0.76600	1	3
O36	O39	0.379	0.75800	2	4
O36	O52	0.428	0.85600	2	3
O39	O52	0.625	1.25000	4	3
O-O average		**0.47800**	0.95600		
O-O total		2.86800	**5.73600**		
grand total bonding		5.47800	**10.95600**		
percent Si-O		47.64513	**47.64513**		
percent O-O		52.35487	**52.35487**		
grand total non-bonding		2.52200	**5.04400**		

(AOMix) A	B	Wiberg (P*S)(P*S)	Mayer bond orders B(AB)	B(alpha)	B(beta)
1	2	0.623	0.623	0.312	0.312
1	3	0.383	0.383	0.192	0.192
1	4	0.43	0.43	0.215	0.215
1	5	0.63	0.63	0.315	0.315
2	3	0.428	0.428	0.214	0.214
2	4	0.379	0.379	0.19	0.19
2	5	0.675	0.675	0.337	0.337
3	4	0.625	0.625	0.313	0.313
3	5	0.629	0.629	0.314	0.314
4	5	0.676	0.676	0.338	0.338

(AOMix)	A	B	C	3-center BO	O-O-O-O (AOMix)	
O-O-O	1	3	4	0.262	4-CENTER BO index	
O-O-O	1	4	3	0.431	B(ABCD)=	**0.110**
O-O-O	3	1	4	0.431		
O-O-O	3	4	1	0.262		
O-O-O	4	1	3	0.262		
O-O-O	4	3	1	0.431		
Average				**0.3465**		

Fragment			Mayer Total Valences (AOMix)	Mulliken Atomic Valences	(Spartan)
O	1	:	4.402	O23	4.390613
O	2	:	4.319	O36	4.30722
O	3	:	4.403	O52	4.393025
O	4	:	4.318	O39	4.309564
Si	5	:	**3.148**	Si15	**3.134682**
O average			**4.361**		**4.35011**

Si35.spartan		-16936.8543296	hartrees		
Si35	**DFT**	**B3LYP**	**6-311++G****		
		Wiberg	= Mayer		
atom#1	atom#2	Bond Order	# e (BO x 2)	AOMix Atoms/Fragments	
Si1	O1	0.47500	0.95000	5	1
Si1	O2	0.49600	0.99200	5	2
Si1	O3	0.68200	1.36400	5	3
Si1	O4	0.68600	1.37200	5	4
Si-O average		**0.58475**	1.16950		
Si-O total		2.33900	**4.67800**		
atom#1	atom#2	Bond Order	# e (BO x 2)		
O1	O2	0.34500	0.69000	1	2
O1	O3	0.62600	1.25200	1	3
O1	O4	0.42500	0.85000	1	4
O2	O3	0.43300	0.86600	2	3
O2	O4	0.62300	1.24600	2	4
O3	O4	0.36600	0.73200	3	4
O-O average		**0.46967**	0.93933		
O-O total		2.81800	**5.63600**		
grand total bonding		5.15700	**10.31400**		
percent Si-O		45.35583	**45.35583**		
percent O-O		54.64417	**54.64417**		
grand total non-bonding		2.84300	**5.68600**		

(AOMix) A	B	Wiberg (P*S)(P*S)	Mayer bond orders B(AB)	B(alpha)	B(beta)
1	2	0.345	0.345	0.172	0.172
1	3	0.626	0.626	0.313	0.313
1	4	0.425	0.425	0.212	0.212
1	5	0.475	0.475	0.238	0.238
2	3	0.433	0.433	0.217	0.217
2	4	0.623	0.623	0.312	0.312
2	5	0.496	0.496	0.248	0.248
3	4	0.366	0.366	0.183	0.183
3	5	0.682	0.682	0.341	0.341
4	5	0.686	0.686	0.343	0.343

(AOMix)	A	B	C	3-center BO
O-O-O	2	3	4	0.259
O-O-O	2	4	3	0.378
O-O-O	3	2	4	0.378
O-O-O	3	4	2	0.259
O-O-O	4	2	3	0.259
O-O-O	4	3	2	0.378
Average				**0.3185**

O-O-O-O	(AOMix)
4-CENTER BO index	
B(ABCD)=	**0.120**

O-Si-O-O	(AOMix)
4-CENTER BO index	
B(ABCD)=	**0.141**

Fragment			Mayer Total Valences (AOMix)	Mulliken Atomic Valences	(Spartan)
O	1	:	3.95	O1	3.939064
O	2	:	3.975	O2	3.964072
O	3	:	4.421	O3	4.411214
O	4	:	4.401	O4	4.38968
Si	5	:	**3.255**	Si1	**3.252976**
O average			**4.187**		**4.17601**

$Si_6O_{18}H_{12}$	ring				
Si6.spartan		-3100.0343896	hartrees		
Si6	DFT	B3LYP	6-311++G**		
		Wiberg	= Mayer		
atom#1	atom#2	Bond Order	# e (BO x 2)	AOMix Atoms/Fragments	
O19	O20	0.245	0.49000	1	2
O20	O21	0.214	0.42800	2	3
O21	O22	0.211	0.42200	3	4
O22	O23	0.246	0.49200	4	5
O23	O24	0.213	0.42600	5	6
O24	O19	0.21	0.42000	6	1
O-O average		**0.22317**	0.44633		
O-O total		1.33900	**2.67800**		

(AOMix)		Wiberg	Mayer bond orders		
A	B	(P*S)(P*S)	B(AB)	B(alpha)	B(beta)
1	2	0.245	0.245	0.123	0.123
1	6	0.21	0.21	0.105	0.105
2	3	0.214	0.214	0.107	0.107
3	4	0.211	0.211	0.106	0.106
4	5	0.246	0.246	0.123	0.123
5	6	0.213	0.213	0.106	0.106

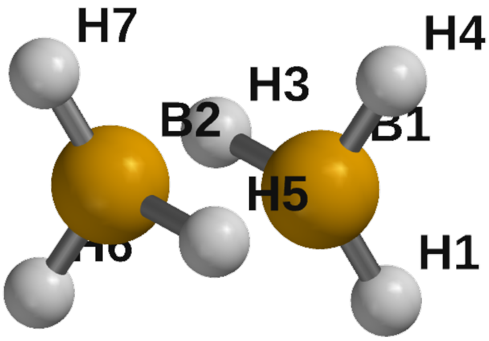

B2H6.spartan		-53.304498	hartrees	
diborane	DFT	B3LYP	6-311++G**	
(AOMix)	A	B	C	3-center BO
B-H-B	2 = B1	3 = H3$_{bridging}$	6 = B2	0.252
B-B-H	2	6	3	0.252
H-B-B	3	2	6	0.252
H-B-B	3	6	2	0.252
B-B-H	6	2	3	0.252
B-H-B	6	3	2	0.252
Average				**0.252**
(AOMix)	A	B	C	3-center BO
B-H-B	2 = B1	5 = H5$_{bridging}$	6 = B2	0.253
B-B-H	2	6	5	0.253
H-B-B	5	2	6	0.253
H-B-B	5	6	2	0.253
B-B-H	6	2	5	0.253
B-H-B	6	5	2	0.253
Average				**0.253**

Valence Bond Path Computations

Valence bond path computations were performed for the *Si21* silica cluster (Table S13), HOON (Table S28), ozone (Table S29), and the *Si5* silica cluster (Table S8). Computations, detailed in the Tables above, were conducted via *Spartan* (B3LYP/6-311++G) and the corresponding *Spartan* Output file (*name*.txt), the *Spartan* Verbose Output file (*name*.text), and the *Spartan* Archive file (*name*.sparchive) were processed by the computational program *Chemissian* (Leonid Skripnikov, Version 4.67, www.chemissian.com, operating on Windows 10).[6] Within *Chemissian*, an electron density job/function was built from all valence molecular orbitals as follows:

Si21: HOMO-223, –28.86 eV through HOMO, –8.24 eV
HOON: HOMO-8, –36.98 eV through HOMO, –7.37 eV
ozone: HOMO-9, –37.97 eV through HOMO, –5.56 eV
Si5: HOMO-63, –28.89 eV through HOMO, –8.51 eV

One-dimensional valence electron density plots, along the O-O (or Si-O) bond axis (centrally located for the *Si5* and *Si21* clusters) were generated with distance on the *x*-axis and electron density (e/bohr^3) on the *y*-axis. These plots are shown in Figures S6–S13 and the Valence Electron Density (*VED*) was quantified within *Chemissian* via the *0D [Points]* function by computing and importing 1001 equidistant *x,y,z* points along the atom-atom axis. This yielded an *x,y,z,VED* data set for Microsoft Excel, allowing generation of the plots in Figure 9a and Figure S13. Microsoft Excel was employed to compute the average Valence Electron Density (VED_{ave}) along each internuclear axis. This value was multiplied by the interatomic distance (converted to bohr) to provide the Valence Electron Density projection (VED_{proj}). The Valence Electron Density minimum (VED_{min}) was taken directly from zoomed plots generated by *Chemissian* (see Figures S6–S12).

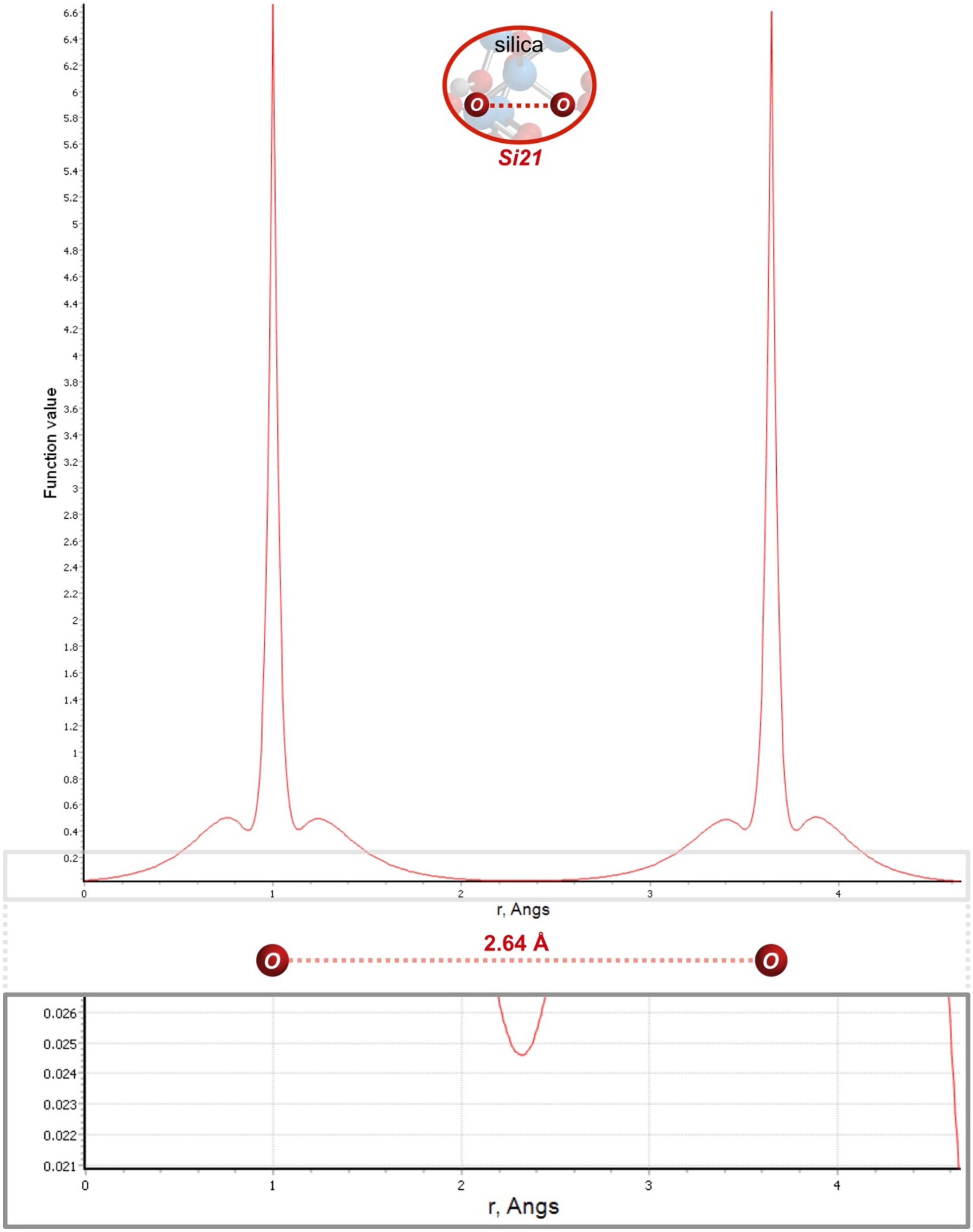

Figure S6. One-dimensional valence electron density plot (e/bohr^3), along a central O-O bond axis of the *Si21* cluster (Table S13, O23–O36, 2.64 Å = 4.99 bohr). Core molecular orbitals are excluded and all valence molecular orbitals are included: HOMO-223, –28.86 eV through HOMO, –8.24 eV. The zoomed plot shows the VED_{min} of 0.025 e/bohr^3 near the midpoint of the O-O bond axis.

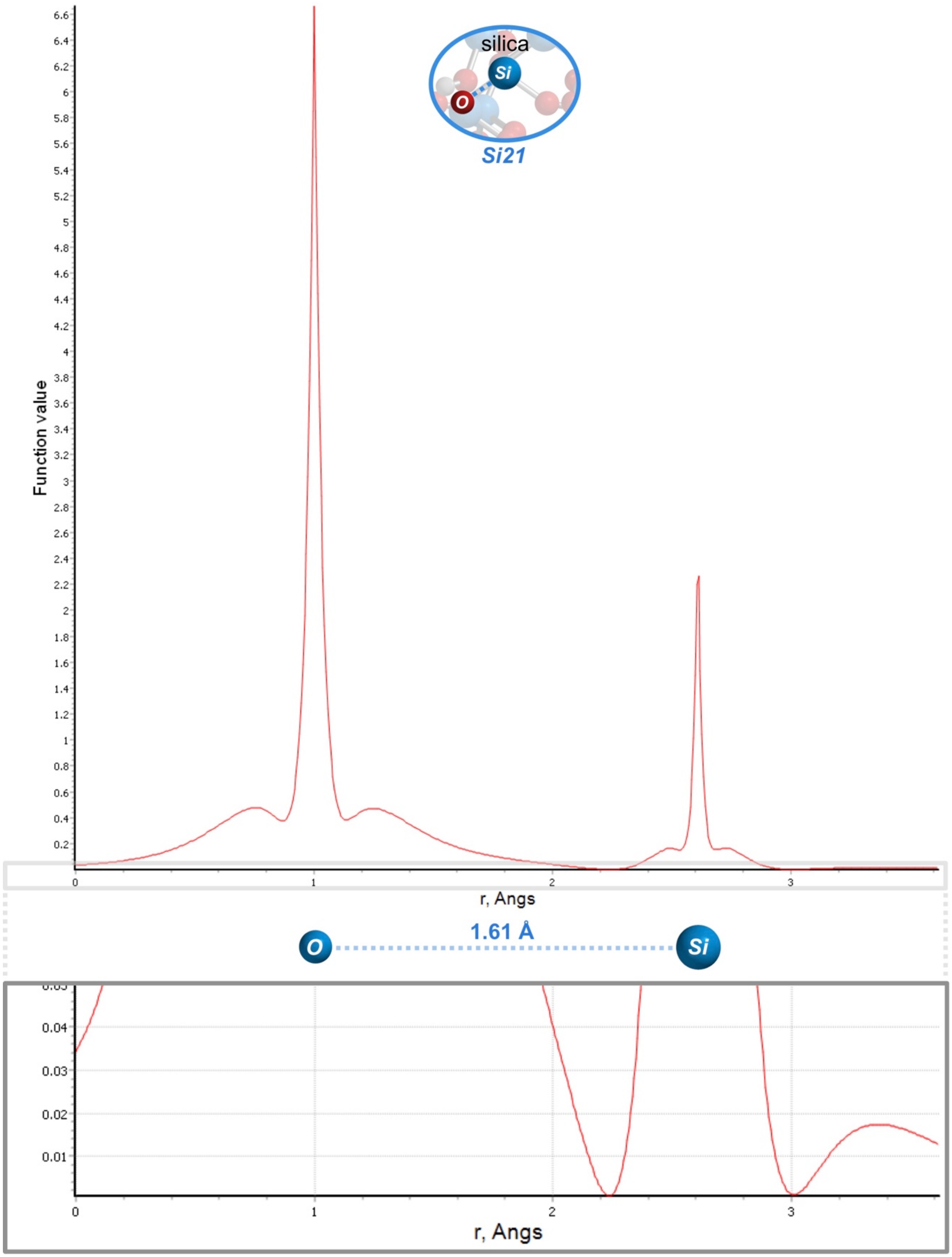

Figure S7. One-dimensional valence electron density plot (e/bohr^3), along a central O-Si bond axis of the *Si21* cluster (Table S13, O23–Si15, 1.61 Å = 3.04 bohr). Core molecular orbitals are excluded and all valence molecular orbitals are included: HOMO-223, –28.86 eV through HOMO, –8.24 eV. The zoomed plot shows the VED_{min} of 0.000 e/bohr^3 about 0.4 Å from the silicon nucleus.

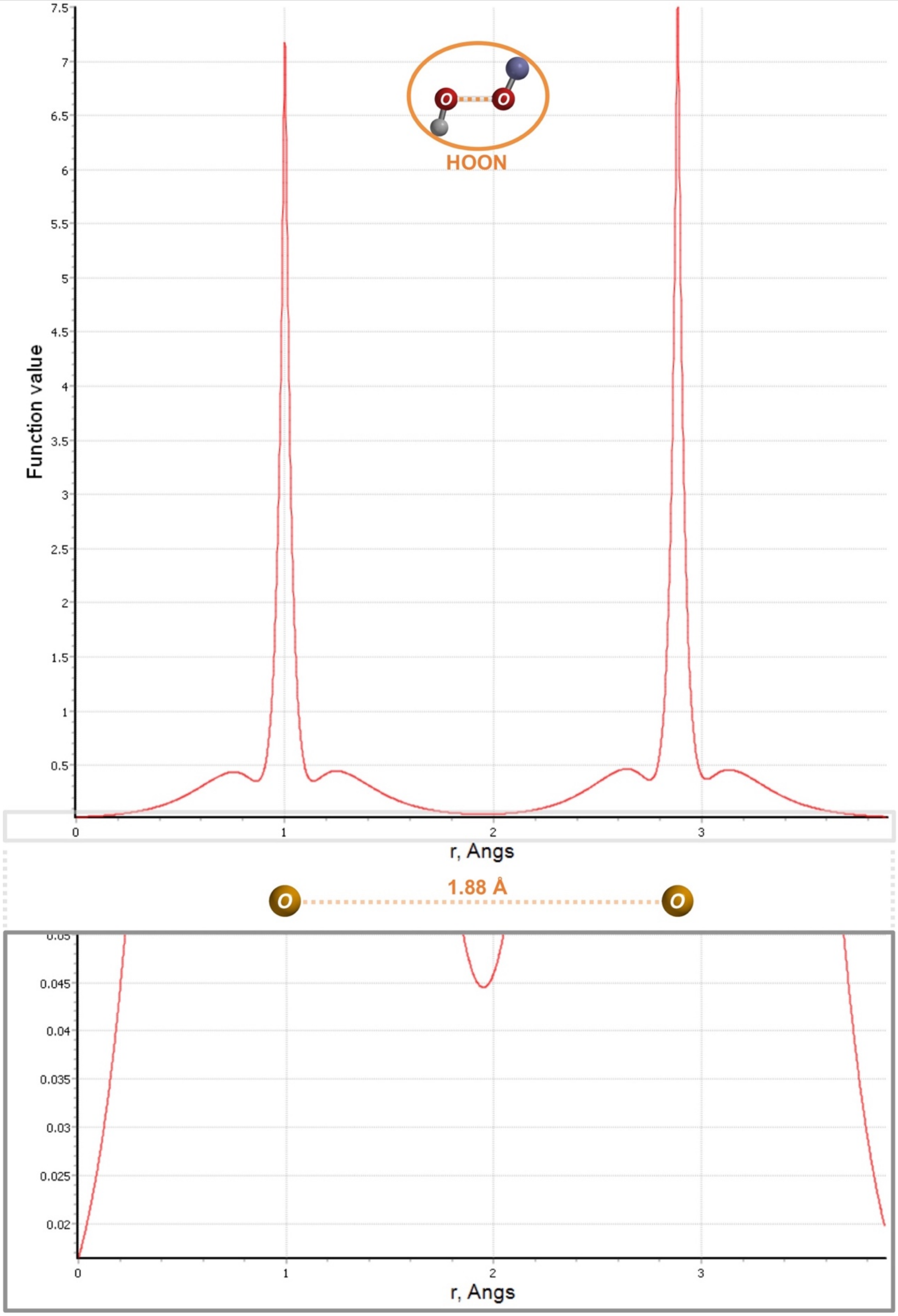

Figure S8. One-dimensional valence electron density plot (e/bohr^3), along the O-O bond axis of HOON (Table S28, O1–O2, 1.88 Å = 3.56 bohr). Core molecular orbitals are excluded and all valence molecular orbitals are included: HOMO-8, –36.98 eV through HOMO, –7.37 eV. The zoomed plot shows the VED_{min} of 0.045 e/bohr^3 near the midpoint of the O-O bond axis.

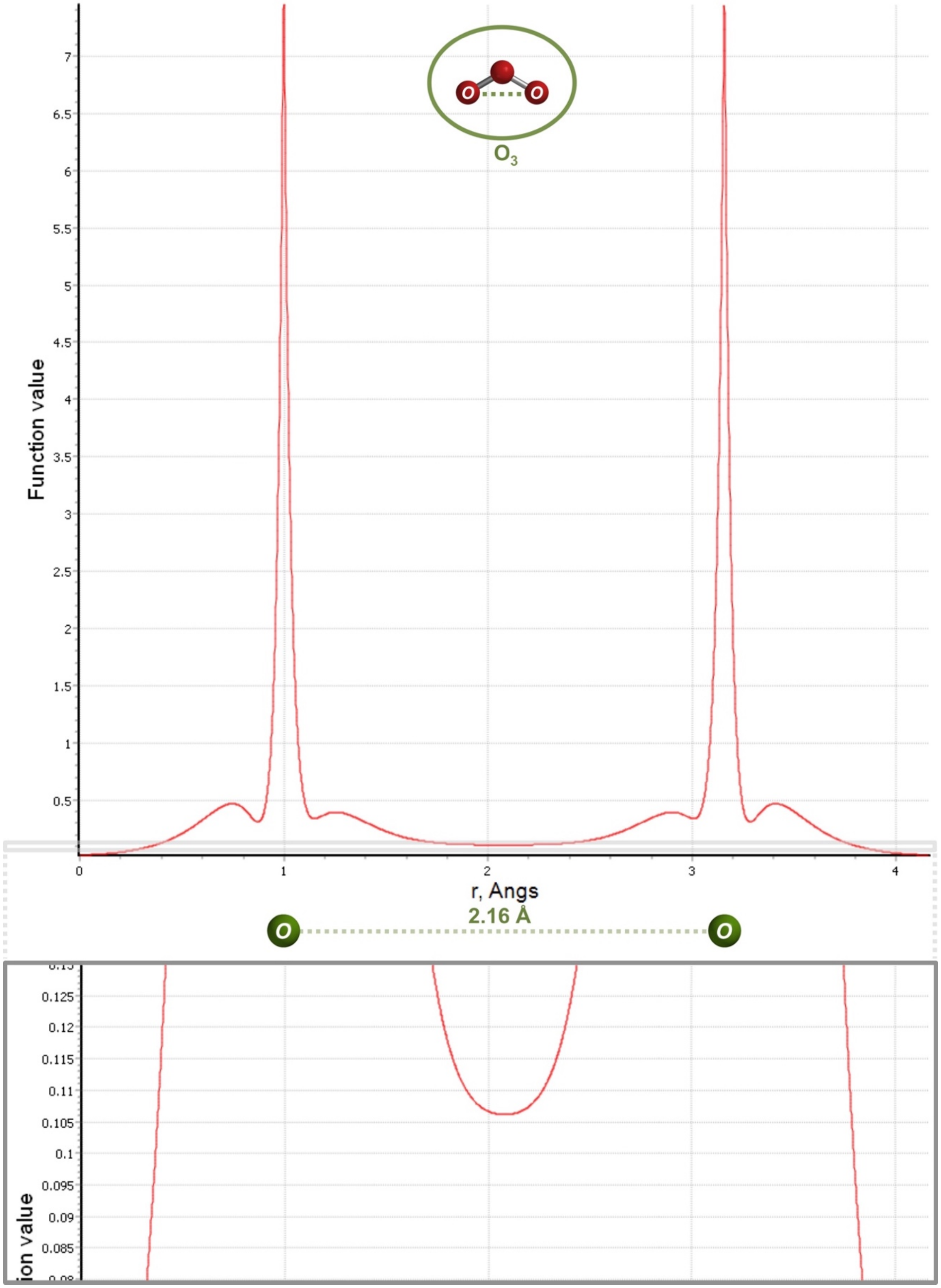

Figure S9. One-dimensional valence electron density plot (e/bohr^3), along the long O-O bond axis of ozone, O_3 (Table S29, O1–O3, 2.16 Å = 4.08 bohr). Core molecular orbitals are excluded and all valence molecular orbitals are included: HOMO-9, –37.97 eV through HOMO, –5.56 eV. The zoomed plot shows the VED_{min} of 0.107 e/bohr^3 near the midpoint of the long O-O bond axis.

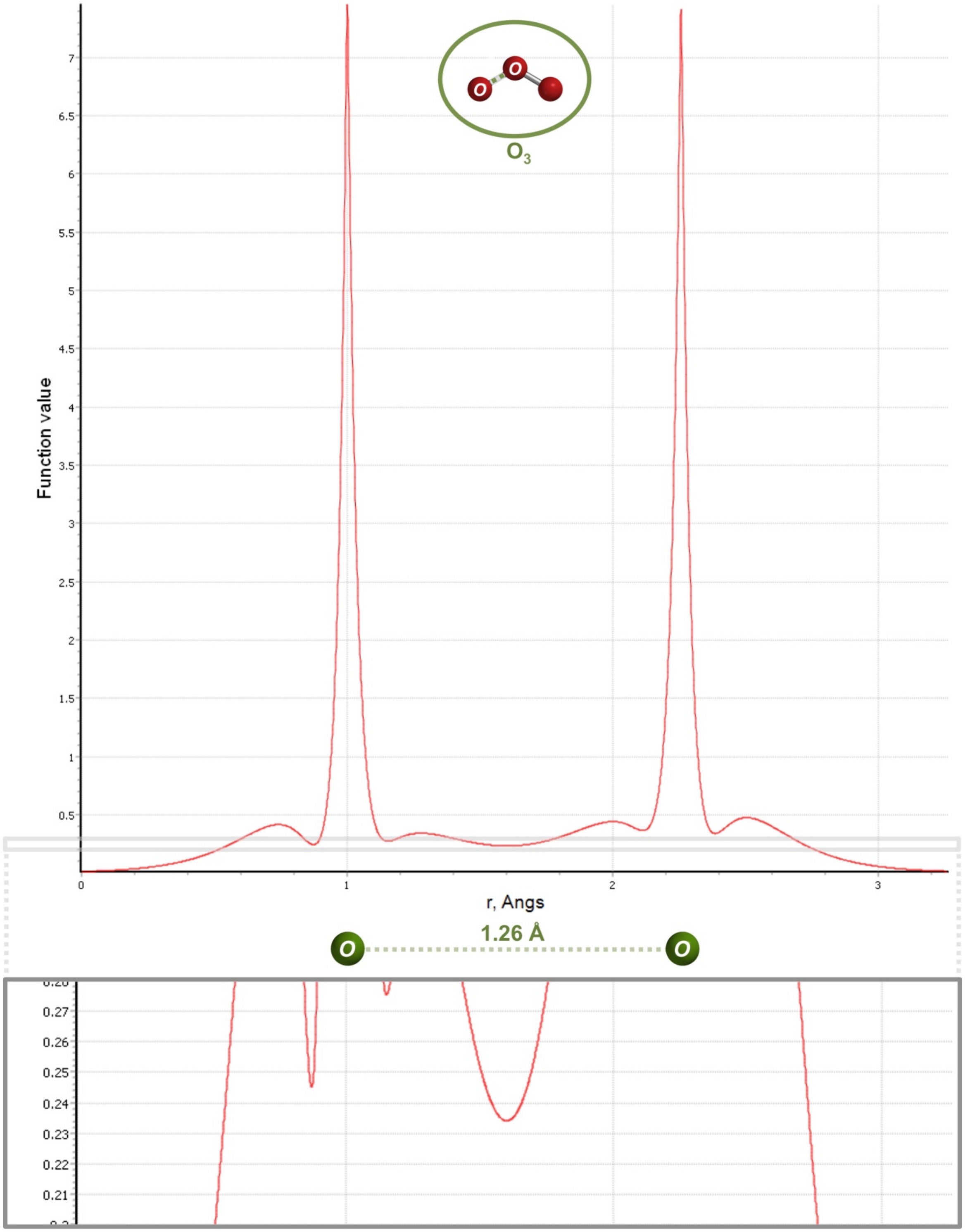

Figure S10. One-dimensional valence electron density plot (e/bohr^3), along a short O-O bond axis of ozone, O_3 (Table S29, O1–O2, 1.26 Å = 2.37 bohr). Core molecular orbitals are excluded and all valence molecular orbitals are included: HOMO-9, –37.97 eV through HOMO, –5.56 eV. The zoomed plot shows the VED_{min} of 0.235 e/bohr^3 near the midpoint of the short O-O bond axis.

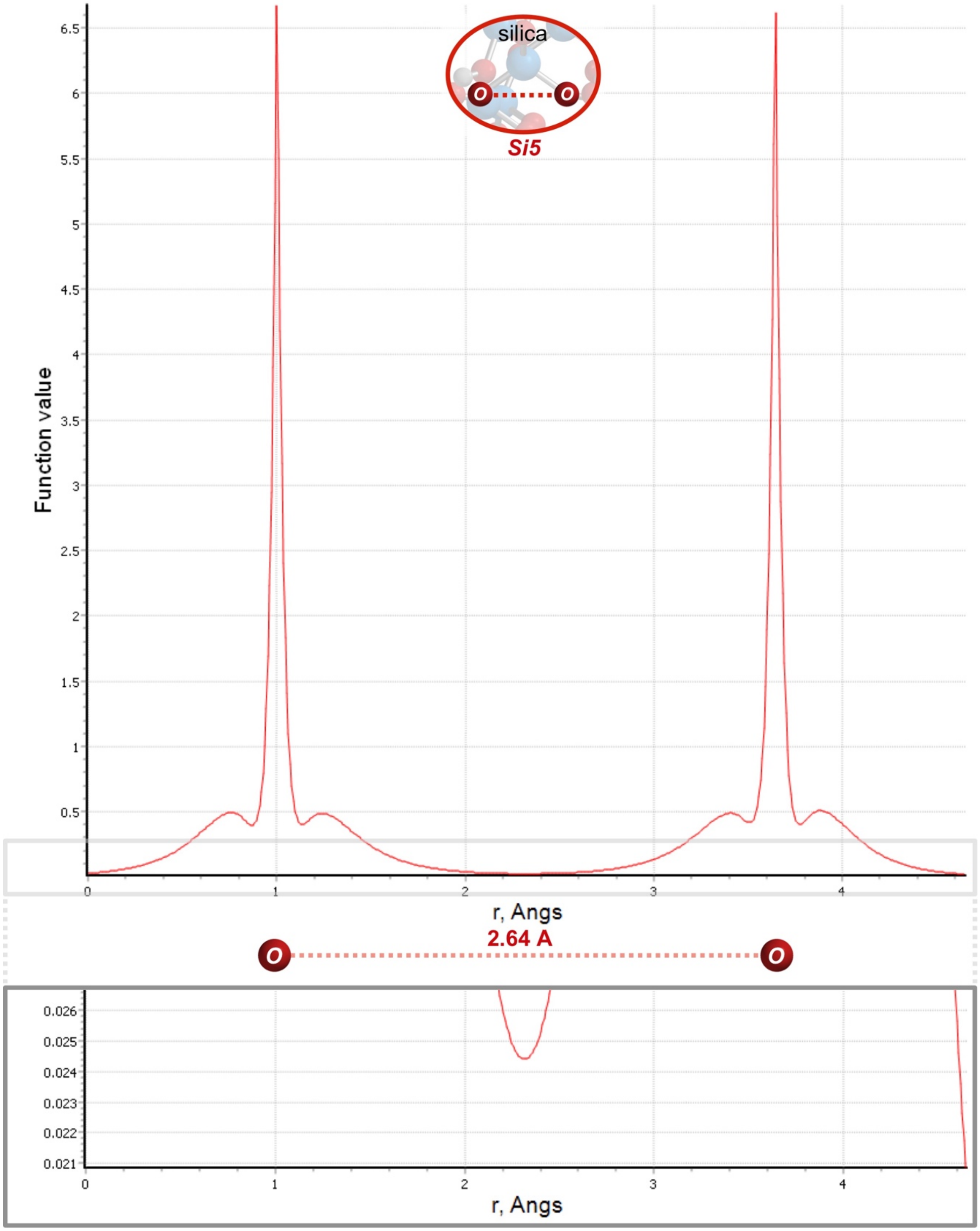

Figure S11. One-dimensional valence electron density plot (e/bohr^3), along a central O-O bond axis of the *Si5* cluster (Table S8, O23–O36, 2.64 Å = 4.99 bohr). Core molecular orbitals are excluded and all valence molecular orbitals are included: HOMO-63, –28.89 eV through HOMO, –8.51 eV. The zoomed plot shows the VED_{min} of 0.0244 e/bohr^3 near the midpoint of the O-O bond axis.

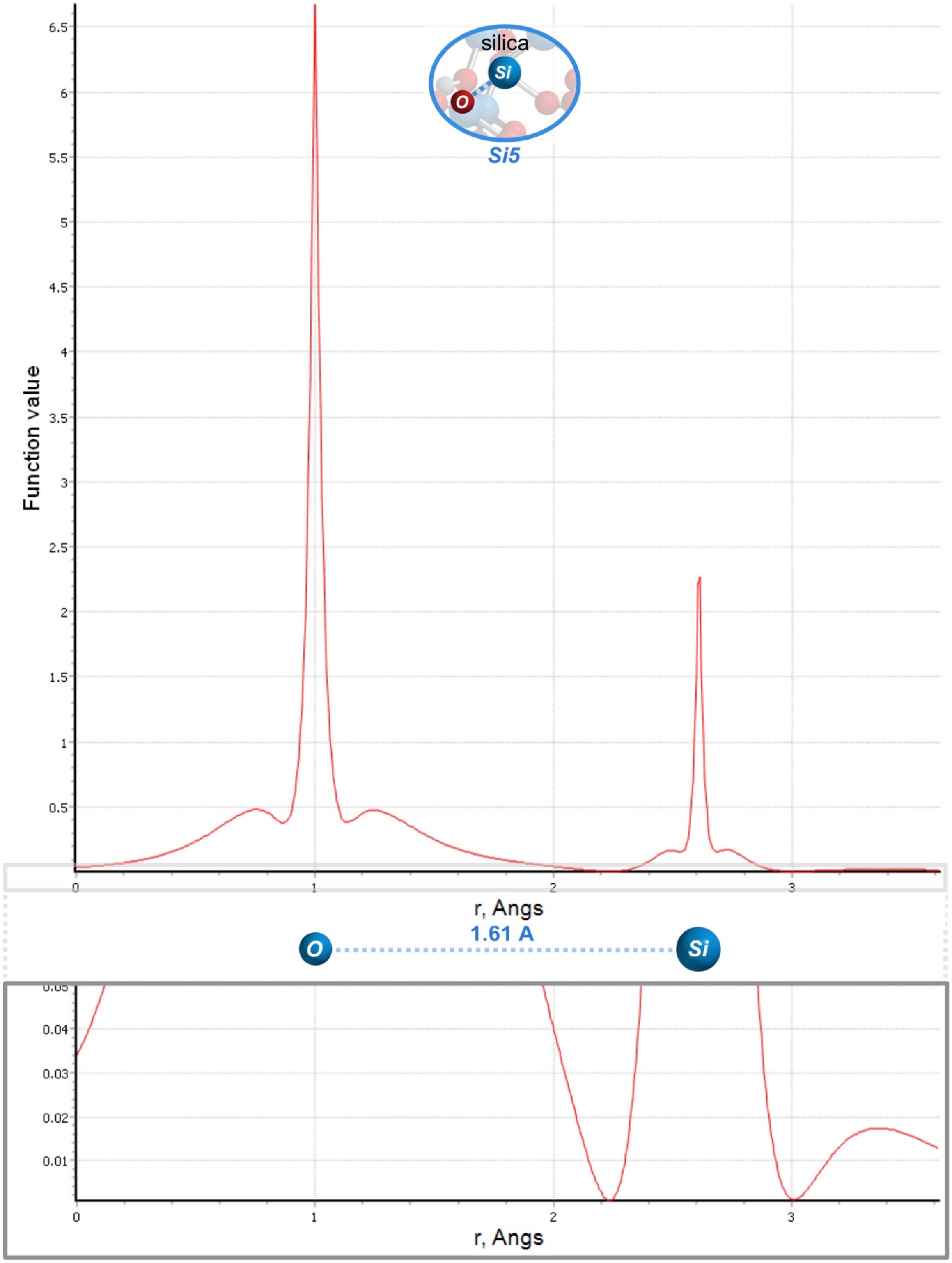

Figure S12. One-dimensional valence electron density plot (e/bohr^3), along a central O-Si bond axis of the *Si5* cluster (Table S8, O23–Si15, 1.61 Å = 3.04 bohr). Core molecular orbitals are excluded and all valence molecular orbitals are included: HOMO-63, –28.89 eV through HOMO, –8.51 eV. The zoomed plot shows the VED_{min} of 0.000 e/bohr^3 about 0.4 Å from the silicon nucleus.

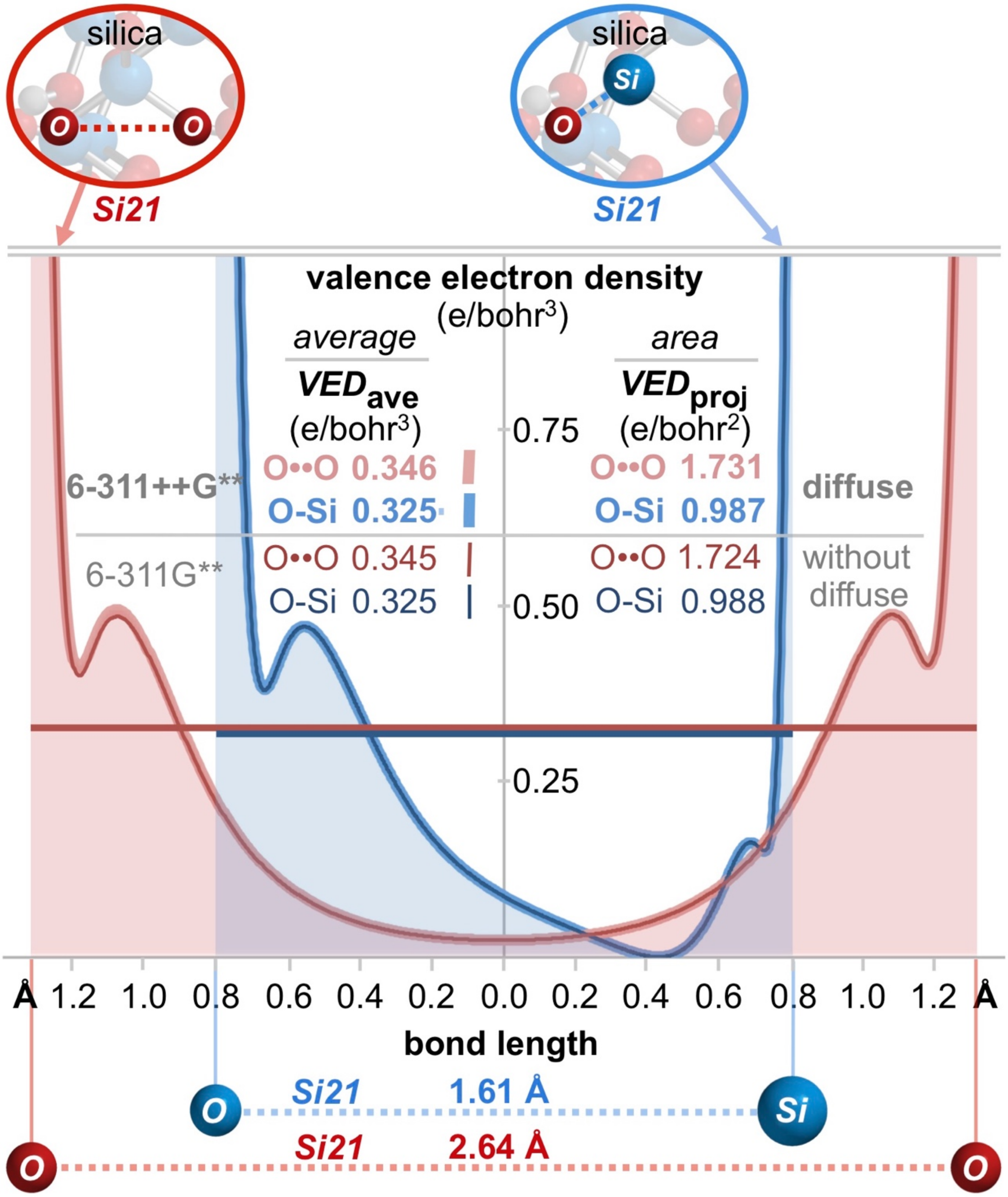

Figure S13. This is similar to Figure 12, with the following elaborated description: One-dimensional valence electron density plots (e/bohr^3), along central O-O (O23–O36, 2.64 Å = 4.99 bohr) and O-Si (O23–Si15, 1.61 Å = 3.04 bohr) bond axes of the *Si21* cluster (Table S13), for computations with diffuse functionals (thick lines; 6-311++G**) and without diffuse functionals (thin lines; 6-311G**). Core molecular orbitals are excluded and all valence molecular orbitals are included: HOMO-223, –28.86 eV through HOMO, –8.24 eV for 6-311++G** and HOMO-223, –28.64 eV through HOMO, –7.97 eV for 6-311G**. The curves are nearly coincident except for a region 0.1 to 0.4 Å from the left oxygen nucleus along the O-O axis.

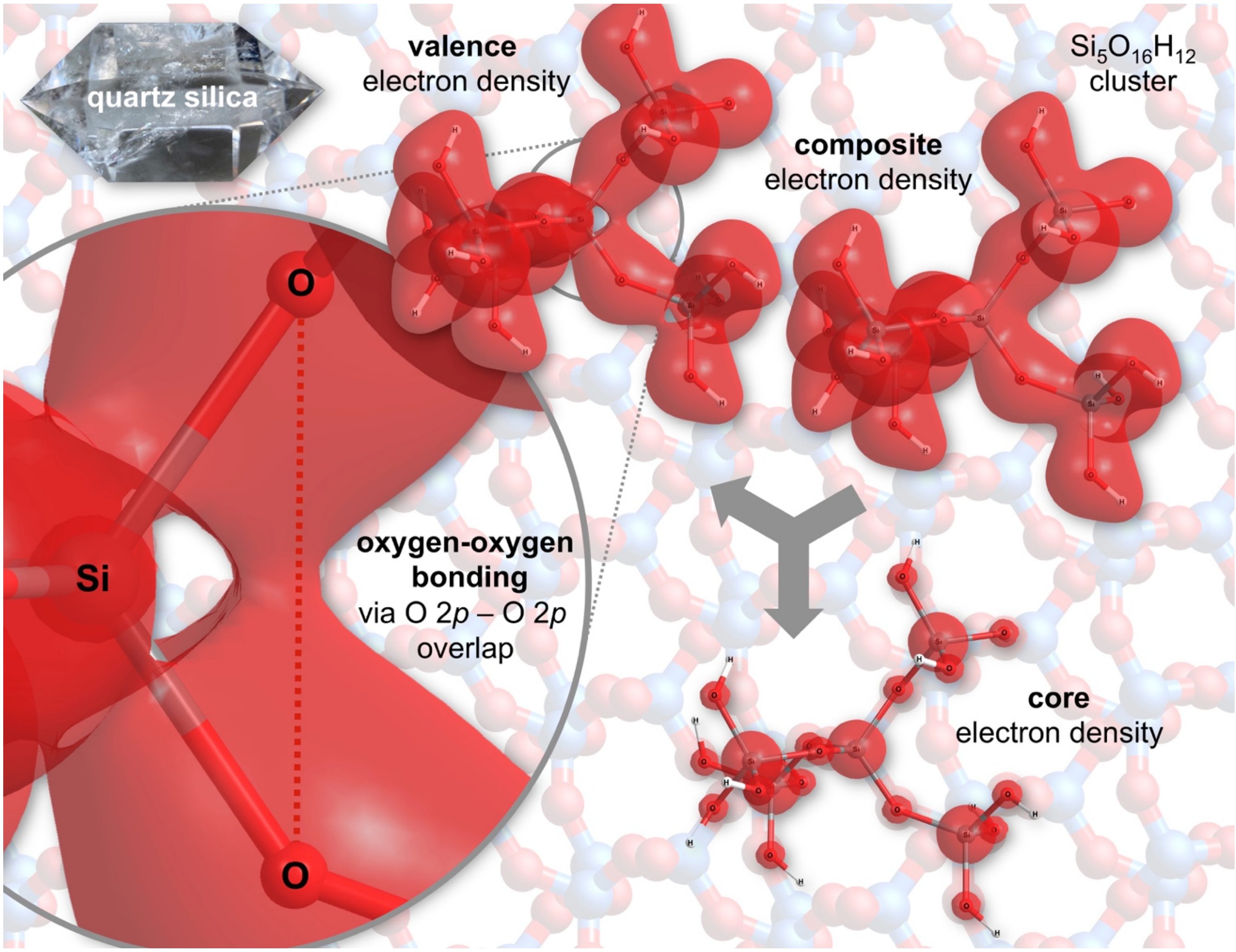

Figure S14. This valence bond path image derives from the *Si5* cluster, $Si_5O_{16}H_{12}$ (Table S8, B3LYP/6-311++G**). The *Spartan* Output file (Si5.txt), the *Spartan* Verbose Output file (Si5.text), and the *Spartan* Archive file (Si5.sparchive) were processed by the computational program *Chemissian* (Leonid Skripnikov**,** Version 4.67, www.chemissian.com, operating on Windows 10). Three-dimensional electron density surfaces (IsoValue = 0.022 e/bohr^3) were generated for the **core** molecular orbitals (HOMO-104, –1800.85 eV through HOMO-64, –100.30 eV), the **valence** molecular orbitals (HOMO-63, –28.89 eV through HOMO, –8.51 eV), and the **composite** (core plus valence) molecular orbitals (HOMO-104, –1800.85 eV through HOMO, –8.51 eV). The zoomed view of the valence electron density surface illustrates an O-O valence bond path and corresponding bond critical point between oxygen atoms separated by 2.64 Å (O23–O36 of Table S8, VED_{min} = 0.025 e/bohr^3 near the midpoint, see Figure S11); this valence bond path is common to all geminal O-O bonds (2.61–2.64 Å) in *Si5*. In the perspective shown, however, only two O-O valence bond paths are clearly visible. Note that the O-O overlap appears somewhat greater for the central SiO_4 unit versus the distal SiO_4 unit, in agreement with the calculated Mulliken bond orders of 0.30 and 0.16, respectively. (Note: These renderings were generated with *Chemissian* at *fine* resolution.)

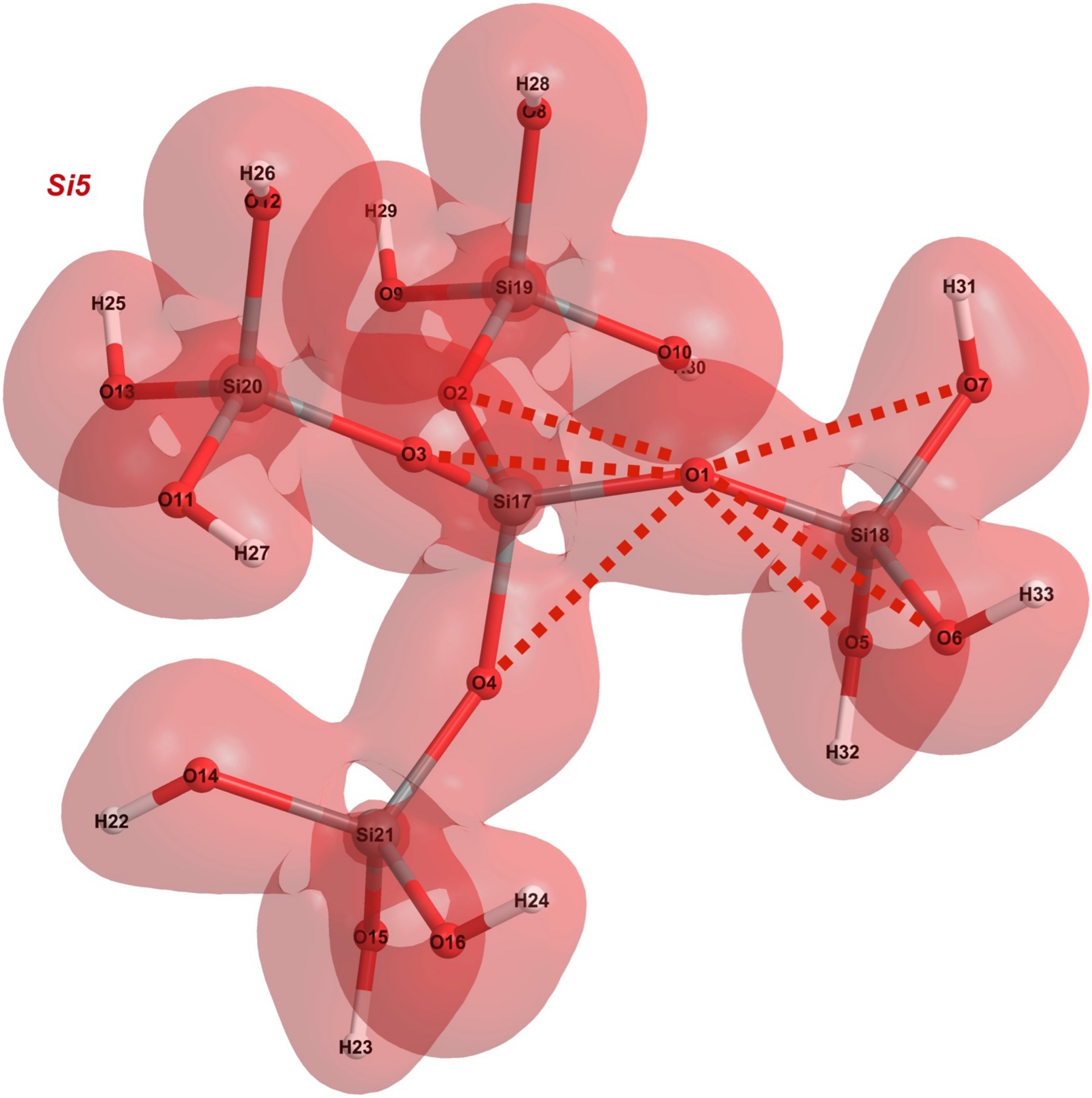

Figure S15. For the *Si5* cluster, $Si_5O_{16}H_{12}$ (Table S8, B3LYP/6-311++G**), each interior oxygen atom has six valence bond paths (surface with IsoValue = 0.022 $e/bohr^3$) to six neighboring oxygen atoms (2.61 to 2.64 Å). The O1-O4 and O1-O7 valence bond paths are the most visible from this perspective. See Figure S16 for additional perspectives. (Note: This rendering was generated with *Chemissian* at *fine* resolution.)

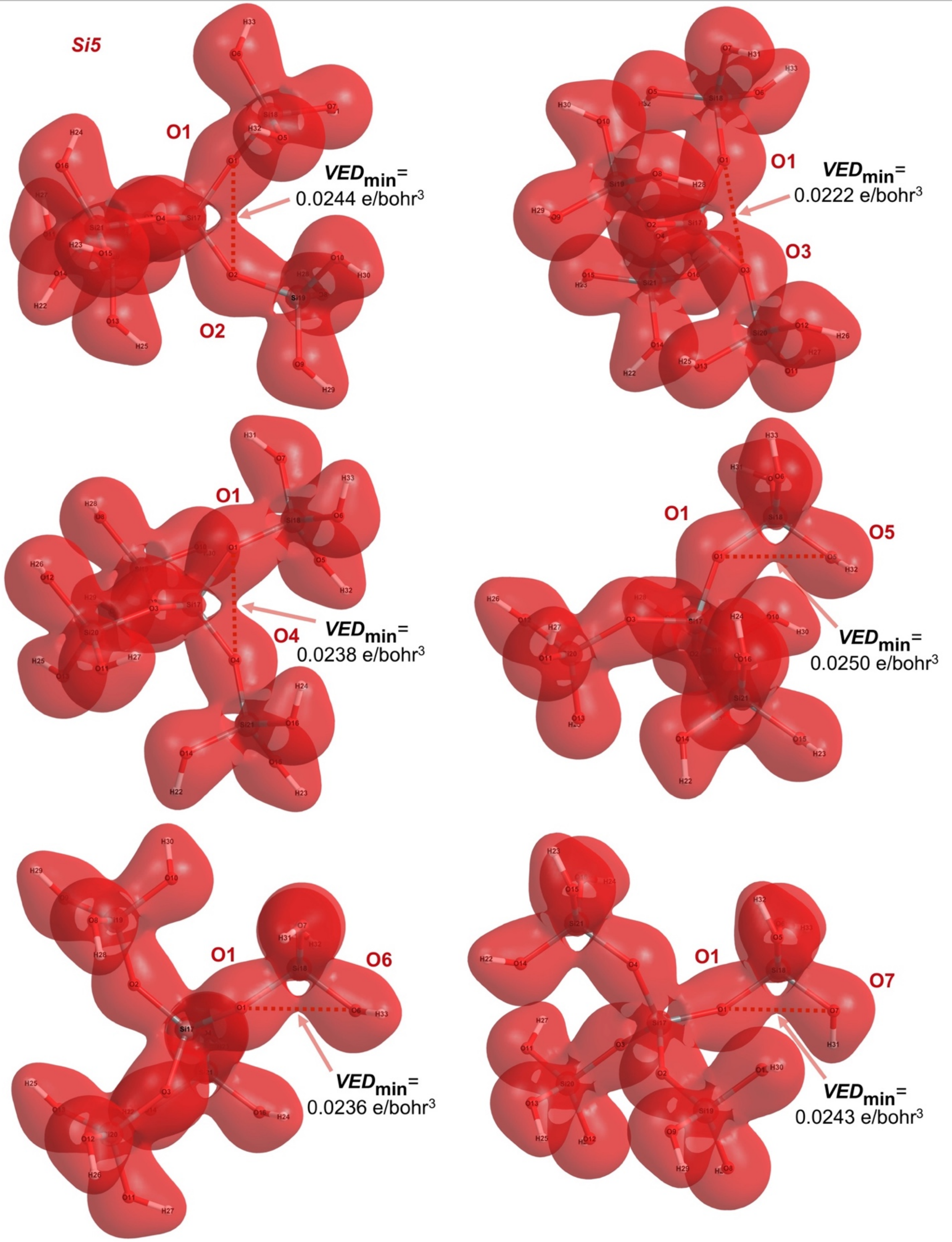

Figure S16. From six different perspectives of the *Si5* cluster, $Si_5O_{16}H_{12}$ (Table S8, B3LYP/6-311++G**), the six valence bond paths emanating from O1 are illustrated via three-dimensional valence electron density surfaces (IsoValue = 0.022 e/bohr^3). The minimum valence electron density (VED_{min} in e/bohr^3) along each O-O axis is shown; these average 0.0239 e/bohr^3. (Note: These renderings were generated with *Chemissian* at *fine* resolution.)

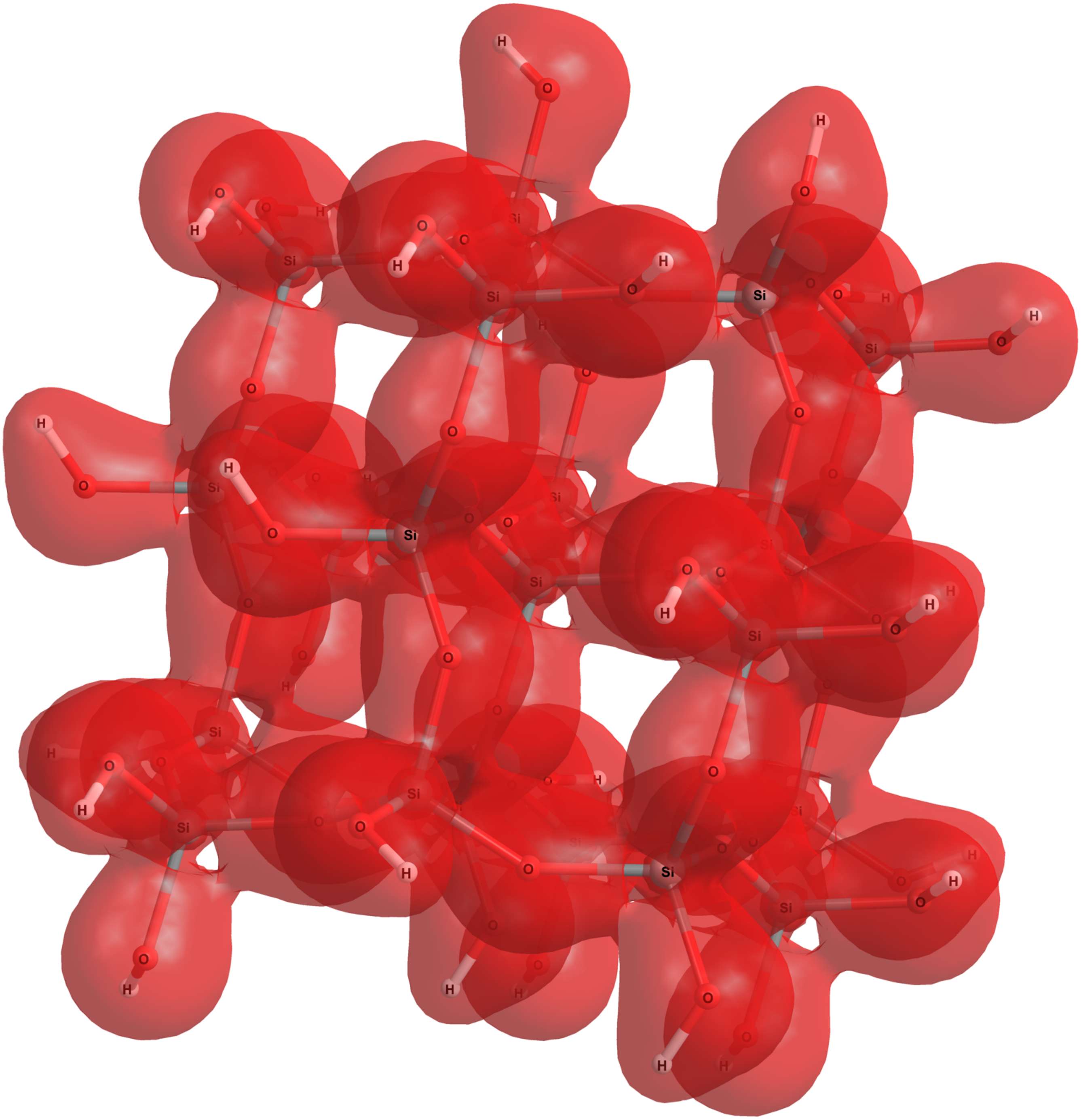

Figure S17. This valence bond path image derives from the *Si21* cluster, $Si_{21}O_{56}H_{28}$ (Table S13, B3LYP/6-311++G**). The *Spartan* Output file (Si21.txt), the *Spartan* Verbose Output file (Si21.text), and the *Spartan* Archive file (Si21.sparchive) were processed by the computational program *Chemissian* (Leonid Skripnikov**,** Version 4.67, www.chemissian.com, operating on Windows 10). A three-dimensional electron density surface (IsoValue = 0.022 e/bohr^3) was generated for the **valence** molecular orbitals (HOMO-223, –28.86 eV through HOMO, –8.24 eV). In the perspective shown, several oxygen-oxygen valence bond paths are visible; this valence bond path is common to all 2.61–2.64 Å O-O bonds in *Si21*. (Note: This rendering was generated with *Chemissian* at *medium* resolution and required about two weeks of computation time.)

Bond Dissociation Energy Calculations

Table S34. Thermodynamic calculations (B3LYP/6-311++G**) for the reactions of Figure 10. Enthalpies are computed via E, the electronic energy, provided in hartrees and converted to kcal/mol.

(a)

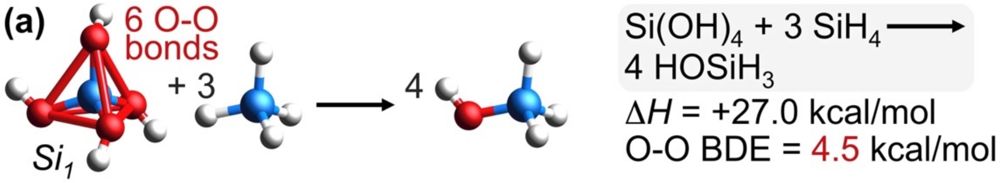

DFT B3LYP 6-311++G**	E (hartrees)	E (kcal/mol)
Si(OH)4 + 3 SiH4 --> 4 H3SiOH		
H3SiOH O11	-367.2049934	-230424.4382
H3SiOH O12	-367.2050867	-230424.4968
H3SiOH O19	-367.205124	-230424.5202
H3SiOH O20	-367.2050262	-230424.4588
SiH4	-291.9141721	-183178.7702
Si(OH)4 (*Si1*)	-593.1208008	-372188.6406
	Δ*H*	27.03735478
	Δ*H* / 6	4.506225797

(b)

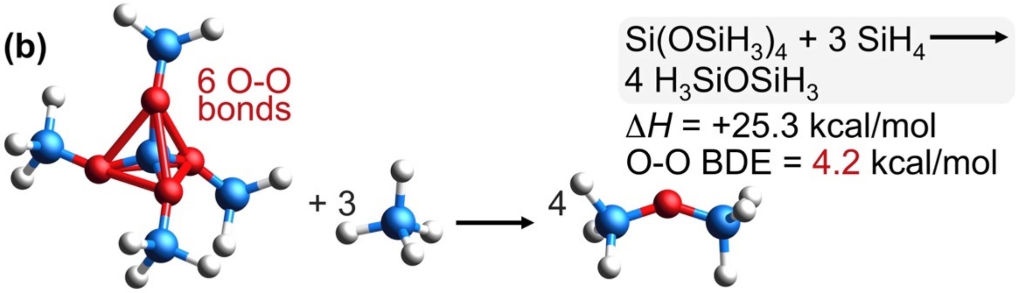

DFT B3LYP 6-311++G**	E (hartrees)	E (kcal/mol)
Si(OSiH3)4 + 3 H3SiH --> 4 H3SiOSiH3		
H3SiOSiH3 O23	-657.962791	-412877.573
H3SiOSiH3 O36	-657.9627572	-412877.5518
H3SiOSiH3 O39	-657.9627235	-412877.5307
H3SiOSiH3 O52	-657.962775	-412877.563
H3SiH	-291.9141721	-183178.7702
Si(OSiH3)4	-1756.148812	-1101999.185
	Δ*H*	25.27700378
	Δ*H* / 6	4.212833964

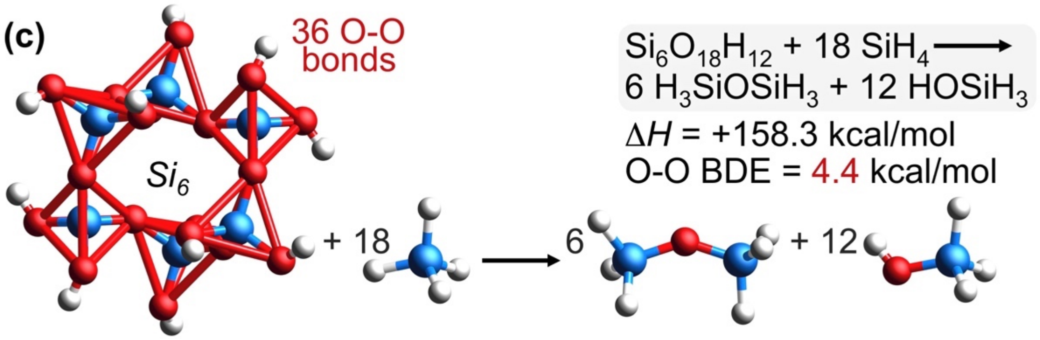

DFT B3LYP 6-311++G**	E (hartrees)	E (kcal/mol)
Si6 cluster + 18 H3SiH --> 6 H3SiOSiH3 + 12 H3SiOH		
H3SiOH Si2 O11	-367.2049935	-230424.4383
H3SiOH Si7 O13	-367.2049643	-230424.4199
H3SiOH Si8 O15	-367.2049795	-230424.4295
H3SiOH Si9 O1	-367.2050011	-230424.443
H3SiOH Si10 O3	-367.2050253	-230424.4582
H3SiOH Si11 O5	-367.2050036	-230424.4446
H3SiOH Si2 O12	-367.2050868	-230424.4968
H3SiOH Si7 O14	-367.2050665	-230424.4841
H3SiOH Si8 O16	-367.2051111	-230424.5121
H3SiOH Si9 O2	-367.2050677	-230424.4848
H3SiOH Si10 O4	-367.2051214	-230424.5185
H3SiOH Si11 O6	-367.2051243	-230424.5203
H3SiOSiH3 O19	-657.9627848	-412877.5691
H3SiOSiH3 O20	-657.9627852	-412877.5694
H3SiOSiH3 O21	-657.9627698	-412877.5597
H3SiOSiH3 O22	-657.9627308	-412877.5352
H3SiOSiH3 O23	-657.9627559	-412877.551
H3SiOSiH3 O24	-657.9628178	-412877.5898
Si 6 cluster	-3100.03439	-1945299.48
SiH4	-291.9141721	-183178.7702
	ΔH	158.3192657
	ΔH / 36	4.39775738

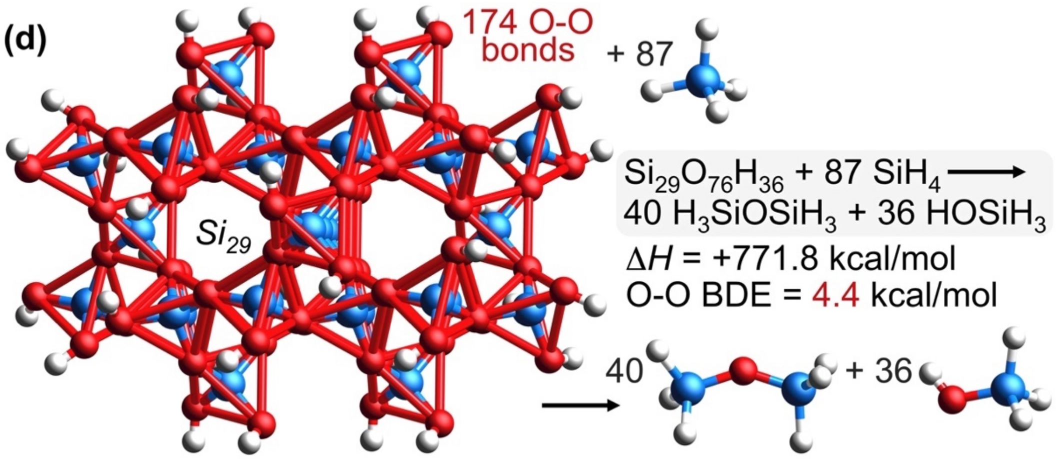

DFT B3LYP 6-311++G**	E (hartrees)	E (kcal/mol)
Si29 cluster + 87 H3SiH --> 40 H3SiOSiH3 + 36 H3SiOH		
H3SiOH Si2 O11	-367.2049935	-230424.4383
H3SiOH Si7 O13	-367.2049643	-230424.4199
H3SiOH Si8 O15	-367.2049795	-230424.4295
H3SiOH Si9 O1	-367.2050011	-230424.443
H3SiOH Si10 O3	-367.2050253	-230424.4582
H3SiOH Si11 O5	-367.2050036	-230424.4446
H3SiOH Si2 O12	-367.2050868	-230424.4968
H3SiOH Si7 O14	-367.2050665	-230424.4841
H3SiOH Si8 O16	-367.2051111	-230424.5121
H3SiOH Si9 O2	-367.2050677	-230424.4848
H3SiOH Si10 O4	-367.2051214	-230424.5185
H3SiOH Si11 O6	-367.2051243	-230424.5203
H3SiOH AVERAGE		-230424.4708
H3SiOSiH3 O19	-657.9627848	-412877.5691
H3SiOSiH3 O20	-657.9627852	-412877.5694
H3SiOSiH3 O21	-657.9627698	-412877.5597
H3SiOSiH3 O22	-657.9627308	-412877.5352
H3SiOSiH3 O23	-657.9627559	-412877.551
H3SiOSiH3 O24	-657.9628178	-412877.5898
H3SiOSiH3 AVERAGE		-412877.5624
Si29 cluster	-14142.58951	-8874602.2
SiH4	-291.9141721	-183178.7702
(computed via average values)	Δ*H*	771.7634672
	Δ*H* / 174	4.435422225

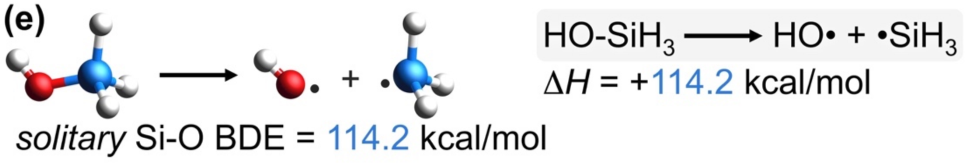

DFT B3LYP 6-311++G**	E (hartrees)	E (kcal/mol)
HO–SiH3 --> HO• + •SiH3		
HOSiH3 Si2 O11	-367.2049935	-230424.4383
•SiH3	-291.2609429	-182768.863
HO•	-75.7621265	-47541.41624
	Δ*H*	114.1590101

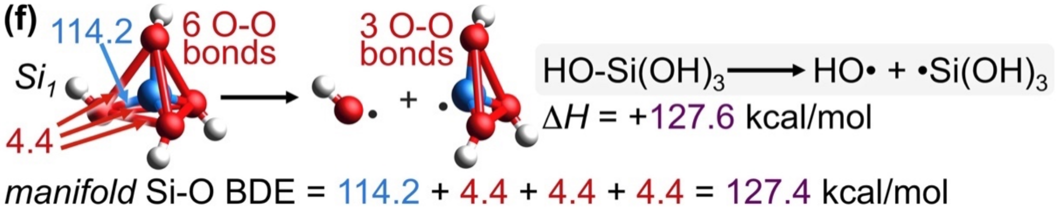

DFT B3LYP 6-311++G**	E (hartrees)	E (kcal/mol)
Si(OH)4 --> HO• +. •Si(OH)3		
Si(OH)4 (*Si1*)	-593.1208008	-372188.6406
•Si(OH)3	-517.15533	-324519.624
HO•	-75.7621265	-47541.41624
	Δ*H*	127.6003783

Table S35. Thermodynamic calculations (B3LYP/6-311++G**) for the reaction of CF_4 + 3 CH_4 → 4 CH_3F. Enthalpies are computed via E, the electronic energy, provided in hartrees and converted to kcal/mol.

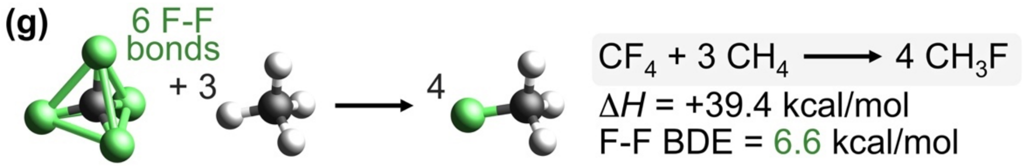

DFT B3LYP 6-311++G**	E (hartrees)	E (kcal/mol)
CF4 + 3 CH4 --> 4 CH3F		
CH3F	-139.791368	-87720.34154
CH4	-40.533956	-25435.4222
CF4	-437.626439	-274614.5291
ΔH		39.42952801
ΔH / 6		6.571588002

Table S36. Computational results for H_3SiOH O11, representative of other computations for H_3SiOH.

Structure

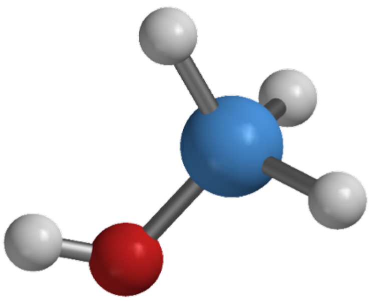

Labeled Structure

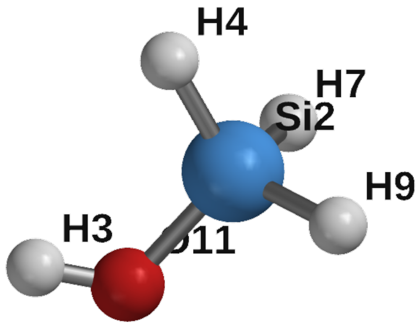

All Si-H bond lengths are 1.481 Å.

SPARTAN '18 Quantum Mechanics Program:	(x86/Darwin) build 1.4.4
SPARTAN file name:	H3SiOH 11.spartan
Job type:	Single point.
Method:	RB3LYP
Basis set:	6-311++G**
Number of	basis functions: 80
Number of electrons:	26
Parallel Job:	3 threads
SCF total energy:	**-367.2049934** hartrees
Reason for exit:	Successful completion
Quantum Calculation CPU Time:	6.40
Quantum Calculation Wall Time:	17.74

Cartesian Coordinates (Ångstroms)

atom#	type	label	X	Y	Z
1	O	O11	-1.2318547	-0.1912884	0.0720534
2	Si	Si2	0.3446878	0.1221147	0.1447331
3	H	H3	-1.603972	-0.5967629	-0.7120913
4	H	H4	0.9632352	-0.0107925	-1.1943323
5	H	H7	0.996831	-0.8349839	1.0677894
6	H	H9	0.5310727	1.5117131	0.6218477

Table S37. Computational results for SiH_4.

Structure

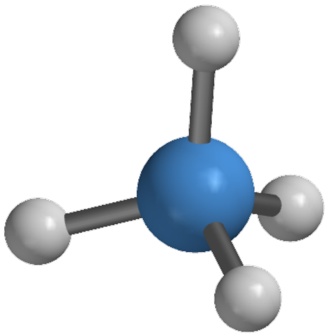

Labeled Structure

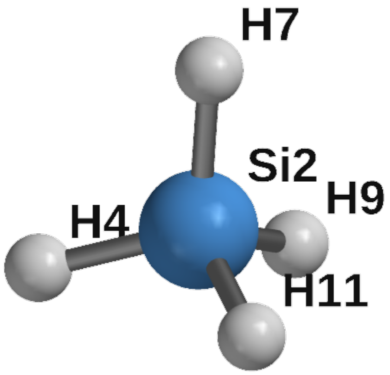

All Si-H bond lengths are 1.481 Å.

SPARTAN '18 Quantum Mechanics Program:	(x86/Darwin) build 1.4.4
SPARTAN file name:	SiH4.spartan
Job type:	Single point.
Method:	RB3LYP
Basis set:	6-311++G**
Number of basis functions:	58
Number of electrons:	18
Parallel Job:	3 threads
SCF total energy:	**-291.9141721** hartrees
Reason for exit:	Successful completion
Quantum Calculation CPU Time:	2.64
Quantum Calculation Wall Time:	2.63

Cartesian Coordinates (Ångstroms)

atom#	type	label	X	Y	Z
1	Si	Si2	-0.0011963	-0.0022255	0.0011582
2	H	H4	0.6173511	-0.1351327	-1.3379072
3	H	H7	-1.4522904	-0.2906906	-0.0657383
4	H	H9	0.1851886	1.3873729	0.4782728
5	H	H11	0.6509469	-0.9593241	0.9242145

Table S38. Computational results for $H_3SiOSiH_3$ O23, representative of other computations for $H_3SiOSiH_3$.

Structure

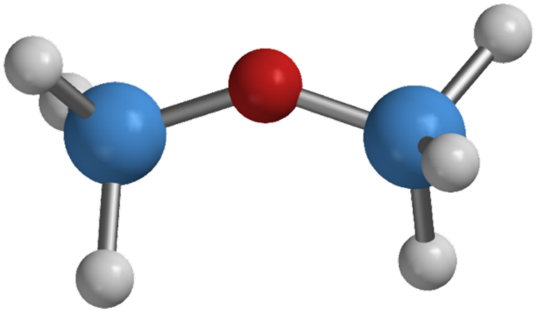

Labeled Structure

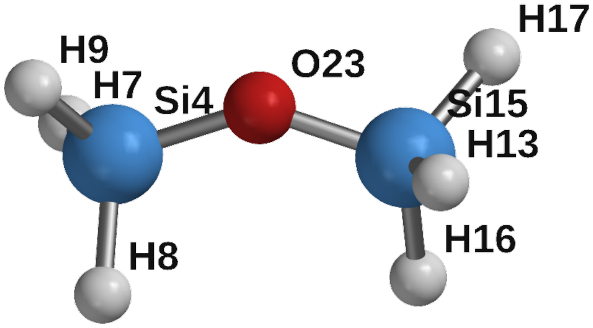

All Si-H bond lengths are 1.481 Å.

SPARTAN '18 Quantum Mechanics Program:	(x86/Darwin) build 1.4.4
SPARTAN file name:	H3SiOSiH3 O23.spartan
Job type:	Single point.
Method:	RB3LYP
Basis set:	6-311++G**
Number of basis functions:	124
Number of electrons:	42
Parallel Job:	3 threads
SCF total energy:	**-657.9627910** hartrees
Reason for exit:	Successful completion
Quantum Calculation CPU Time:	13.32
Quantum Calculation Wall Time:	9.28

Cartesian Coordinates (Ångstroms)

atom#	type	label	X	Y	Z
1	O	O23	-0.0096379	0.4692457	0.2842911
2	Si	Si15	1.1193626	-0.4777547	0.9272913
3	Si	Si4	-1.1186381	0.5562457	-0.8787092
4	H	H7	-0.6538732	1.4820943	-1.937085
5	H	H8	-1.3460704	-0.7779627	-1.4799777
6	H	H9	-2.3895017	1.0378845	-0.2902441
7	H	H13	0.5368395	-1.3345145	1.9855876
8	H	H16	1.691183	-1.3295002	-0.1408436
9	H	H17	2.1703361	0.3742617	1.5296898

Table S39. Computational results for $Si(OSiH_3)_4$.

Structure

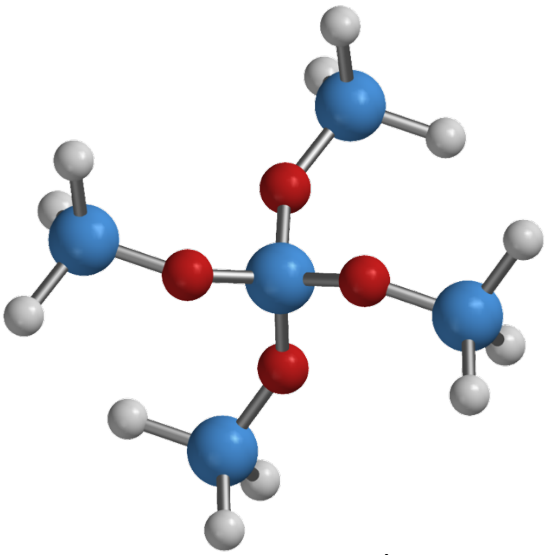

Labeled Structure

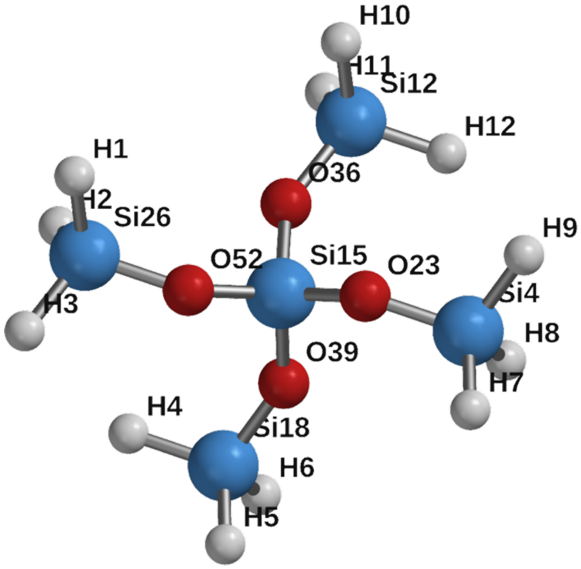

All Si-H bond lengths are 1.481 Å.

SPARTAN '18 Quantum Mechanics Program:	(x86/Darwin) build 1.4.4
SPARTAN file name:	Si(OSiH3)4.spartan
Job type:	Single point.
Method:	RB3LYP
Basis set:	6-311++G**
Number of basis functions:	322
Number of electrons:	114
Parallel Job:	3 threads
SCF total energy:	**-1756.1488119** hartrees
Reason for exit:	Successful completion
Quantum Calculation CPU Time:	16:38.18
Quantum Calculation Wall Time:	6:27.69

Cartesian Coordinates (Ångstroms)

atom#	type	label	X	Y	Z
1	O	O23	-1.1276251	1.1527796	-0.6421325
2	O	O36	-0.6316249	-0.725221	1.1508681
3	O	O52	1.1423756	1.1307797	0.6548679
4	O	O39	0.6223755	-0.719221	-1.1591327
5	Si	Si15	0.0013753	0.2057793	0.0008677
6	Si	Si4	-2.2366253	1.2397796	-1.8051328
7	Si	Si12	-2.0176253	-1.2382212	1.7838681
8	Si	Si26	2.2513759	1.1917797	1.8188682
9	Si	Si18	2.0023759	-1.2422212	-1.7981328
10	H	H1	1.7976317	2.1103125	2.8883423
11	H	H2	2.4631414	-0.1515506	2.4053667
12	H	H3	3.528346	1.6640835	1.2360838
13	H	H4	3.0228934	-1.3232	-0.7279236
14	H	H5	2.4679702	-0.3165531	-2.856302
15	H	H6	1.7749435	-2.5764296	-2.3994013
16	H	H7	-1.7718604	2.1656283	-2.8635085
17	H	H8	-2.4640577	-0.0944288	-2.4064013
18	H	H9	-3.5074889	1.7214185	-1.2166678

19	H	H10	-2.4722032	-0.3198633	2.8531385
20	H	H11	-1.8067098	-2.5820013	2.3696421
21	H	H12	-3.0389839	-1.2934297	0.7128219

Table S40. Computational results for •OH (hydroxy radical).

Structure

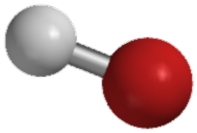

Labeled Structure

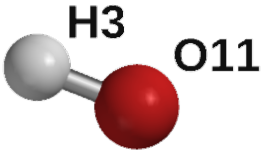

SPARTAN '18 Quantum Mechanics Program: (x86/Darwin) build 1.4.4
SPARTAN file name: OH dot.spartan
Job type: Single point.
Method: UB3LYP
Basis set: 6-311++G**
Number of basis functions: 29
Number of electrons: 9 (1 unpaired)
SCF total energy: **-75.7621265** hartrees
Reason for exit: Successful completion
Quantum Calculation CPU Time: 1.69
Quantum Calculation Wall Time: 3.94
Cartesian Coordinates (Ångstroms)

atom#	type	label	X	Y	Z
1	O	O11	0.1860587	0.2027372	0.3920724
2	H	H3	-0.1860587	-0.2027372	-0.3920724

Table S41. Computational results for •SiH_3 (silyl radical).

Structure

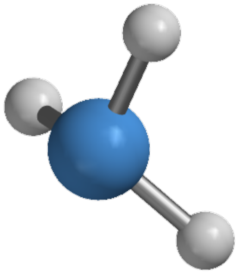

Labeled Structure

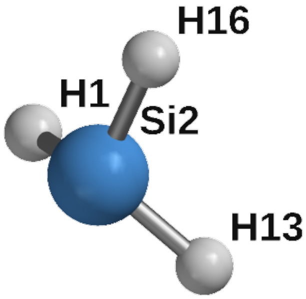

SPARTAN '18 Quantum Mechanics Program: (x86/Darwin) build 1.4.4
SPARTAN file name: H3Si dot.spartan
Job type: Single point.
Method: UB3LYP
Basis set: 6-311++G**
Number of basis functions: 51
Number of electrons: 17 (1 unpaired)
SCF total energy: **-291.2609429** hartrees
Reason for exit: Successful completion

Quantum Calculation CPU Time : 1.36
Quantum Calculation Wall Time: 3.14
Cartesian Coordinates (Ångstroms)

atom#	type	label	X	Y	Z
1	Si	Si2	-0.3642688	-0.0748982	-0.0152764
2	H	H13	0.2878743	-1.0319969	0.90778
3	H	H16	-0.177884	1.3147004	0.4618383
4	H	H1	0.2542785	-0.2078053	-1.3543419

Table S42. Computational results for •Si(OH)3 (trihydroxysilyl radical).

Structure

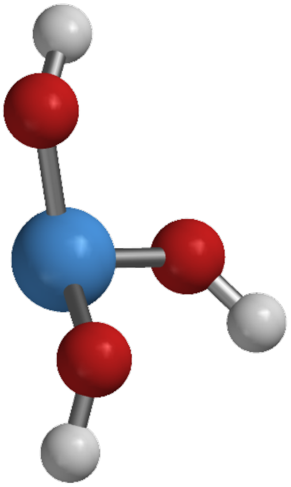

Labeled Structure

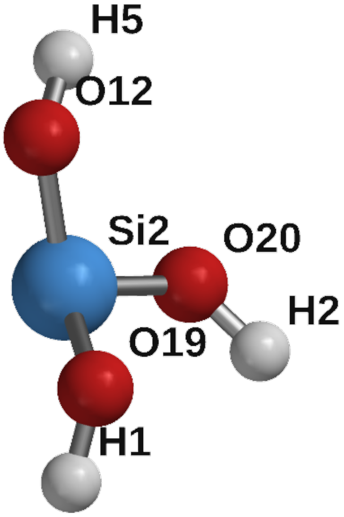

SPARTAN '18 Quantum Mechanics Program: (x86/Darwin) build 1.4.4
SPARTAN file name: Si(OH)3 dot.spartan
Job type: Single point.
Method: UB3LYP
Basis set: 6-311++G**
Number of basis functions: 117
Number of electrons: 41 (1 unpaired)
SCF total energy: **-517.1553300** hartrees
Reason for exit: Successful completion
Quantum Calculation CPU Time: 12.68
Quantum Calculation Wall Time: 16.88
Cartesian Coordinates (Ångstroms)

atom#	type	label	X	Y	Z
1	O	O20	0.2554844	-1.1097986	0.8160039
2	O	O19	-0.2508819	1.4396038	0.3309388
3	O	O12	0.2180776	-0.2138251	-1.6406753
4	Si	Si2	-0.4533047	-0.0695653	-0.18723
5	H	H5	0.5644482	-1.0600639	-1.9264578
6	H	H1	-0.8885032	1.8299622	0.9299572
7	H	H2	0.5546796	-0.8163131	1.6774631

Table S43. Computational results for CF_4, carbon tetrafluoride.

Structure

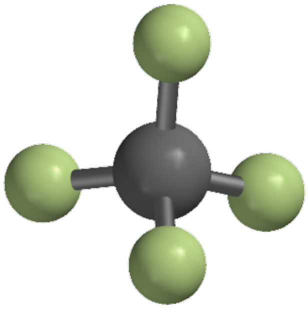

Labeled Structure

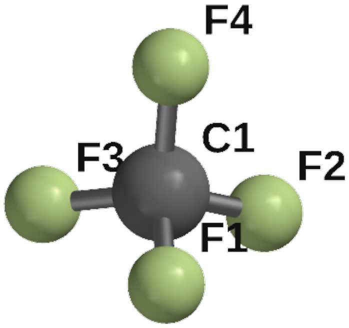

SPARTAN '18 Quantum Mechanics Program: (x86/Darwin) build 1.4.4
SPARTAN file name: carbon tetrafluoride.spartan
Job type: Geometry optimization.
Method: RB3LYP
Basis set: 6-311++G**
Number of basis functions: 110
Number of electrons: 42
Parallel Job: 3 threads
Optimization:

Step	Energy	Max Grad.	Max Dist.
1	-437.615636	0.098392	0.271514
2	-437.625174	0.039617	0.077472
3	-437.626396	0.006989	0.011617
4	**-437.626439**	0.000407	0.000718

Reason for exit: Successful completion
Quantum Calculation CPU Time: 30.19
Quantum Calculation Wall Time: 30.22
Cartesian Coordinates (Ångstroms)

atom#	type	label	X	Y	Z
1	C	C1	0.0001612	0	0.0002979
2	F	F1	-0.0000186	0	1.3288038
3	F	F2	-1.2524696	0	-0.4430543
4	F	F3	0.6261635	1.0847012	-0.4430237
5	F	F4	0.6261635	-1.0847012	-0.4430236

Fluorine-fluorine bonding vs anti-bonding interactions in CF_4. The percent bonding excess (% *be*) is calculated.

molecular orbital	eV	bonding	anti-bonding
**HOMO**	-12.46	2	4
HOMO{-1}	-12.46	1	5
HOMO{-2}	-12.46	2	4
HOMO{-3}	-13.66	4	2
HOMO{-4}	-13.66	5	1
HOMO{-5}	-13.66	3	3
HOMO{-6}	-14.36	4	2
HOMO{-7}	-14.36	6	0
HOMO{-8}	-18.03	6	0
HOMO{-9}	-18.03	5	1
HOMO{-10}	-18.03	6	0
HOMO{-11}	-20.54	6	0
HOMO{-12}	-34.94	3	3
HOMO{-13}	-34.94	0	6
HOMO{-14}	-34.95	3	3
HOMO{-15}	-38.05	6	0
	sum (2*s*)	12	12
	% be_{2s}		**0.000**
	sum (2*p*)	50	22
	% be_{2p}		**38.889**
	sum (2*s*+2*p*)	62	34
	% $be_{F\text{-}F}$		**29.167**

be_{2s} = (12-12)/(12+12) = 0.0 %
be_{2p} = (50-22)/(50+22) = 38.9 %
$be_{F\text{-}F}$ = (62-34)/(62+34) = 29.2 %

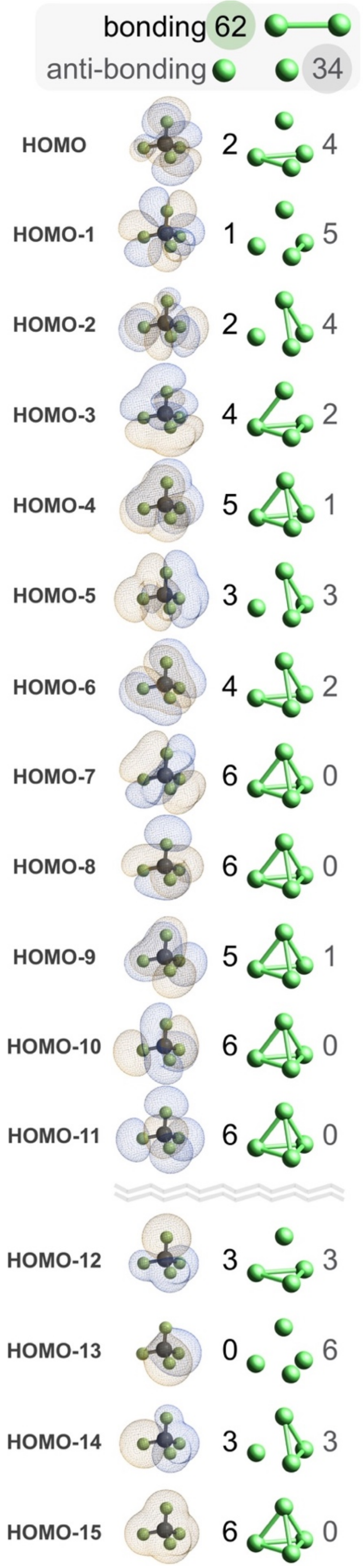

Figure S18. Fluorine-based valence molecular orbitals for CF_4 (IsoValue = 0.01 [e/bohr^3]$^{0.5}$). Bonding and anti-bonding F-F interactions are tabulated. Bonding interactions > anti-bonding interactions and $be_{F\text{-}F}$ = 29%.

Table S44. Computational results for CH_4, methane.

Structure

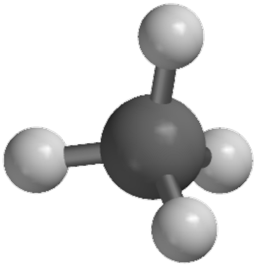

Labeled Structure

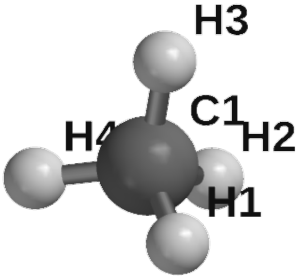

SPARTAN '18 Quantum Mechanics Program: (x86/Darwin) build 1.4.4
SPARTAN file name: methane.spartan
Job type: Geometry optimization.
Method: RB3LYP
Basis set: 6-311++G**
Number of basis functions: 50
Number of electrons: 10
Parallel Job: 3 threads
Optimization:

Step	Energy	Max Grad.	Max Dist.
1	-40.533893	0.006565	0.021826
2	-40.533955	0.000961	0.002787
3	**-40.533956**	0.000013	0.000038

Reason for exit: Successful completion
Quantum Calculation CPU Time: 6.37
Quantum Calculation Wall Time: 4.01
Cartesian Coordinates (Ångstroms)

atom#	type	label	X	Y	Z
1	H	H1	-0.0000198	0	1.090979
2	C	C1	0.0000537	0	0.0000363
3	H	H2	0.5142541	0.8907798	-0.3636748
4	H	H3	-1.0285422	0	-0.3636658
5	H	H4	0.5142541	-0.8907799	-0.3636748

Table S45. Computational results for CH_3F, methyl fluoride.

Structure

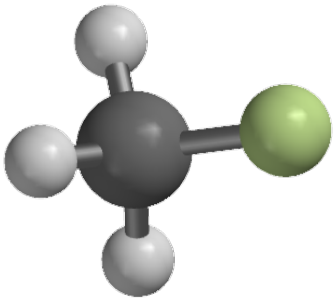

Labeled Structure

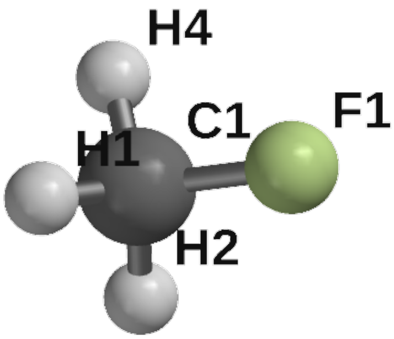

SPARTAN '18 Quantum Mechanics Program:	(x86/Darwin) build 1.4.4
SPARTAN file name:	methyl fluoride.spartan
Job type:	Geometry optimization.
Method:	RB3LYP
Basis set:	6-311++G**
Number of basis functions:	65
Number of electrons:	18
Parallel Job:	3 threads

Optimization:

Step	Energy	Max Grad.	Max Dist.
1	-139.791210	0.007506	0.044540
2	**-139.791368**	0.000460	0.001221

Reason for exit: Successful completion

Quantum Calculation CPU Time:	7.48
Quantum Calculation Wall Time:	9.06

Cartesian Coordinates (Ångstroms)

atom#	type	label	X	Y	Z
1	H	H1	1.0343684	0	0.418294
2	C	C1	-0.0000029	0	0.0701603
3	H	H2	-0.5171992	-0.8957251	0.4183486
4	H	H4	-0.5171992	0.8957251	0.4183486
5	F	F1	0.0000329	0	-1.3251514

Additional Resonance Hybrids

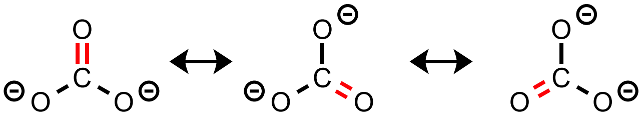

Figure S19. Resonance hybrids for the carbonate dianion, showing the 6 unique bonds (in red) emanating from carbon: 3 sigma and 3 pi.

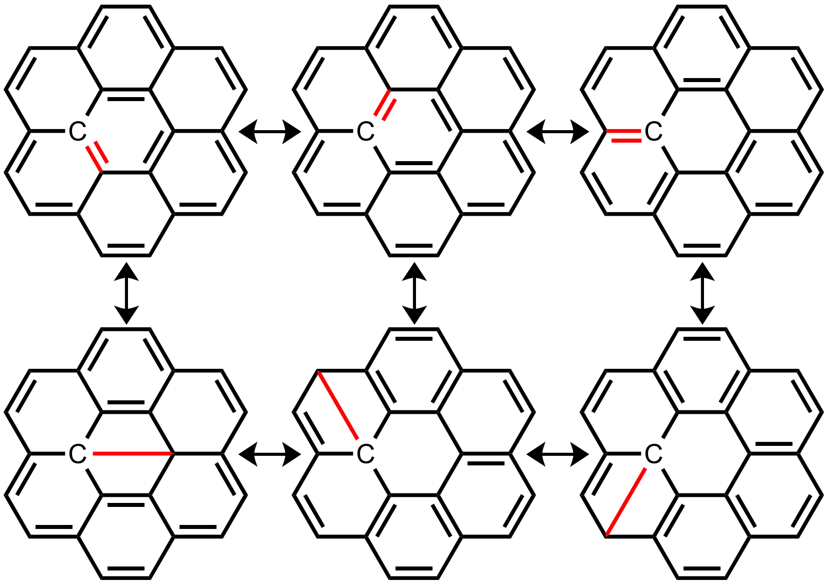

Figure S20. Resonance hybrids for coronene (a model of graphite), showing the 9 unique bonds (in red) emanating from carbon: 3 sigma, 3 pi, and 3 transannular sigma bonds of the Dewar benzene type (bottom row).

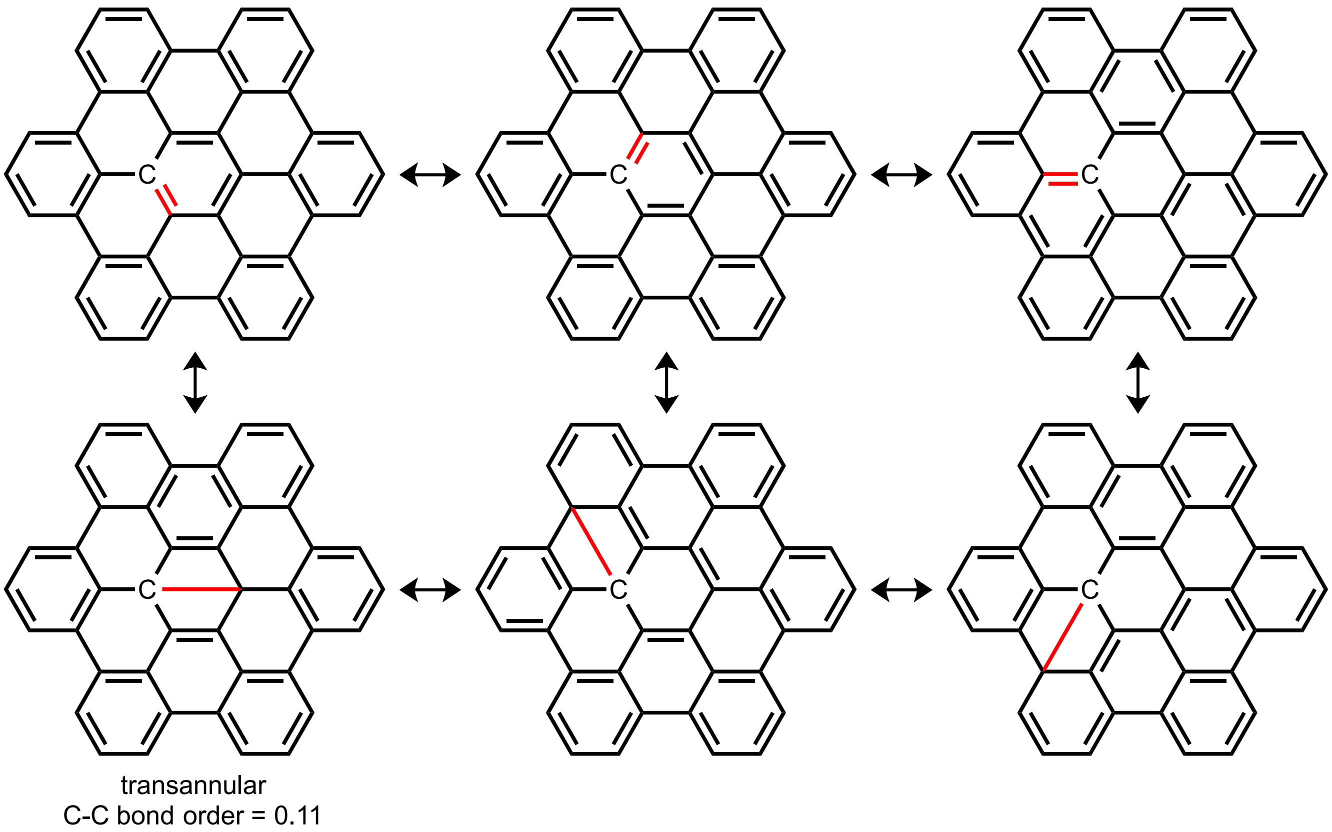

Figure S21. Resonance hybrids for hexabenzocoronene (a model of graphite), showing the 9 unique bonds (in red) emanating from carbon: 3 sigma, 3 pi, and 3 transannular sigma bonds of the Dewar benzene type (bottom row).

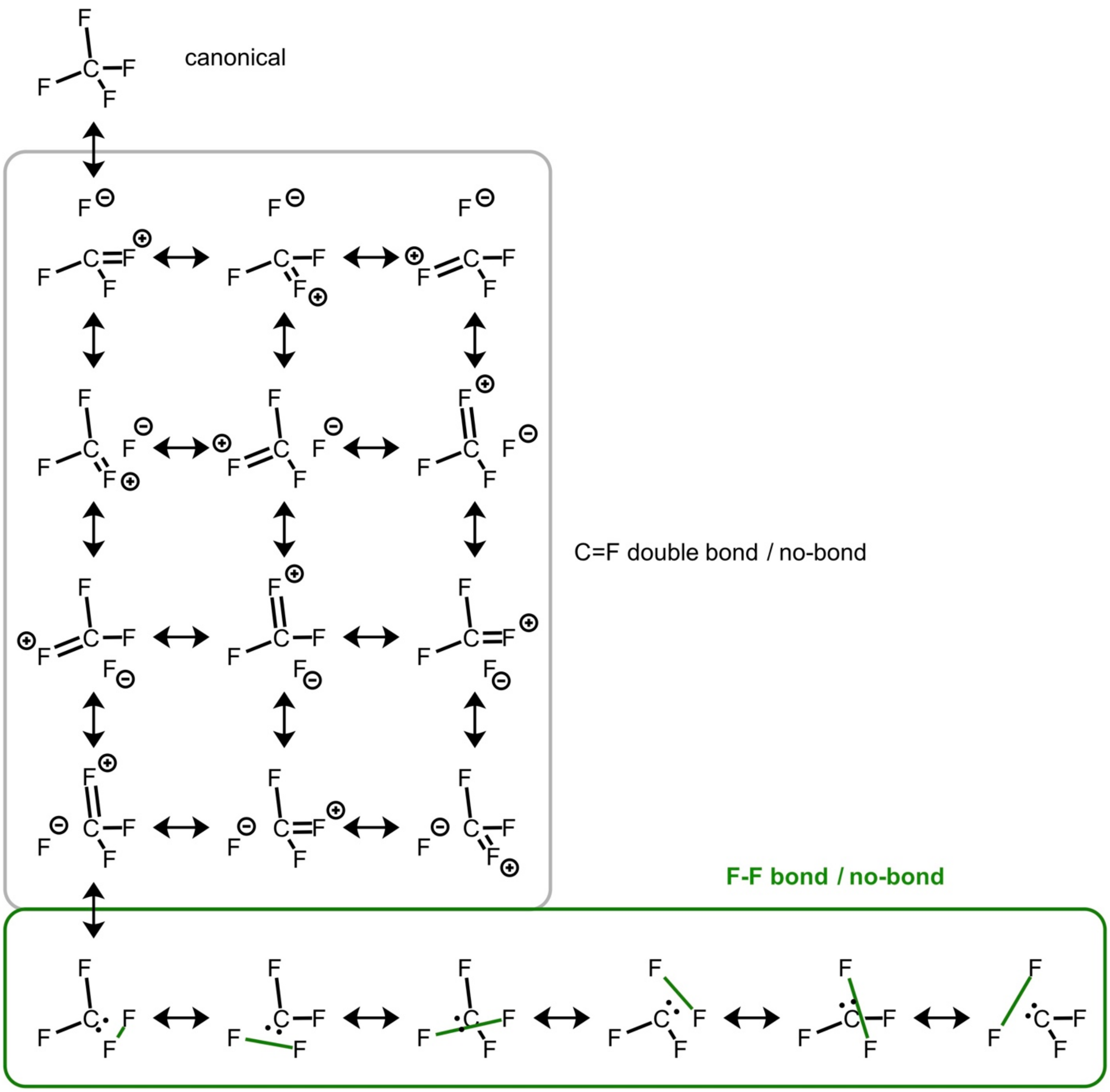

Figure S22. Resonance hybrids for CF_4, showing six proposed **F-F bond / no-bond** resonance hybrids (bottom row) possessing long F-F bonds (2.17 Å) and described as [F-C-F][F-F] in the main text.

Overlap Population Density of States Analysis

Overlap population density of states (OPDOS) analysis was conducted with Multiwfn (version 3.8, written by T. Lu and F. Chen)[7] via Gaussian 09 formatted checkpoint files (Si*xx*.fchk).[8] Gaussian files were created via Spartan-generated pdb files, were computed at the same level of theory (DFT/B3LYP/6-311++G**), and afforded essentially the same computational energies, as tabulated below. Figures S23–S64 show the labeled structures and valence density of states maps generated by Multiwfn for each of the six pairwise oxygen-oxygen interactions for each of the six silica clusters. The following table also defines the two oxygen atoms (Spartan and Multiwfn labeling) per cluster employed to generate the valence density of states maps in Figure 13. For consistency, the same locational pair of oxygen atoms of the central silicate unit was chosen for the comparison in Figure 13.

silica cluster	Spartan Energy (hartrees)	Gaussian Energy (hartrees)	Δ Energy (hartrees)	Δ Energy (kcal/mol)	Spartan O-O	Multiwfn O-O
Si1	-593.1208008	-593.120839363	0.00003856	0.02419863	O12-O19	O2-O3
Si5	-2659.811570	-2659.81140264	-0.00016736	-0.10501991	O23-O52	O1-O3
Si11	-5606.947295	-5606.94698506	-0.00030994	-0.19449014	O10-O32	O20-O21
Si15	-7520.743929	-7520.74416310	0.00023410	0.14689986	O23-O52	O1-O3
Si21	-10315.0041	-10315.0060495	0.00194950	1.22332880	O23-O52	O1-O3
Si25	-12228.80179	-12228.8048650	0.00307500	1.92959018	O44-O31	O36-O37

Note that the data for Table 2 show *averaged* parameters for *all six* pairwise interactions in the central silicate of the six silica clusters. The cumulative OPDOS bonding (+) and anti-bonding (-) data for each pairwise O-O interaction are tabulated and then averaged below. The data in green are locational oxygen-oxygen matches and were used to construct Figure 13.

cluster:	*Si1*	*Si5*	*Si11*	*Si15*	*Si21*	*Si25*
Multiwfn	O1-O2	O1-O2	O17-O18	O1-O2	O1-O2	O33-O34
OPDOS (+)	0.002165	0.012824	0.025385	0.036221	0.064574	0.060824
OPDOS (-)	-0.004528	-0.008482	-0.015350	-0.019110	-0.025893	-0.045378
|(+) / (-)|	0.478136	1.511908	1.653746	1.895395	2.493879	1.340385
Multiwfn	O1-O3	O1-O3	O17-O20	O1-O3	O1-O3	O33-O37
OPDOS (+)	0.003382	0.021471	0.019675	0.049179	0.088523	0.083521
OPDOS (-)	-0.004602	-0.008436	-0.012068	-0.011116	-0.036521	-0.031004
|(+) / (-)|	0.734898	2.545164	1.630345	4.424163	2.423893	2.693878
Multiwfn	O1-O4	O1-O4	O17-O21	O1-O4	O1-O4	O33-O36
OPDOS (+)	0.004748	0.014245	0.019664	0.030588	0.059374	0.068747
OPDOS (-)	-0.003799	-0.006078	-0.013287	-0.018460	-0.022527	-0.032435
|(+) / (-)|	1.249803	2.343699	1.479943	1.656988	2.635682	2.119531
Multiwfn	O2-O3	O2-O3	O18-O20	O2-O3	O2-O3	O34-O36
OPDOS (+)	0.004582	0.014324	0.019746	0.038118	0.059801	0.087364
OPDOS (-)	-0.004921	-0.006159	-0.013332	-0.017147	-0.023036	-0.025609
|(+) / (-)|	0.931112	2.325702	1.481098	2.223013	2.595980	3.411457
Multiwfn	O2-O4	O2-O4	O18-O21	O2-O4	O2-O4	O34-O37
OPDOS (+)	0.003317	0.014333	0.019980	0.029950	0.064825	0.082992
OPDOS (-)	-0.004520	-0.010993	-0.012349	-0.017574	-0.044322	-0.027987
|(+) / (-)|	0.733850	1.303830	1.617945	1.704222	1.462592	2.965377
Multiwfn	O3-O4	O3-O4	O20-O21	O3-O4	O3-O4	O36 -O37
OPDOS (+)	0.002115	0.012911	0.038454	0.035157	0.064010	0.087858
OPDOS (-)	-0.004480	-0.008527	-0.014109	-0.017636	-0.025243	-0.032437
|(+) / (-)|	0.472098	1.514132	2.725494	1.993479	2.535752	2.708574
average for all six O-O interactions						
OPDOS (+)	0.003385	0.015018	0.023817	0.036536	0.066851	0.078551
OPDOS (-)	-0.004475	-0.008113	-0.013416	-0.016841	-0.029590	-0.032475
|(+) / (-)|	0.756387	1.851217	1.775315	2.169502	2.259223	2.418814

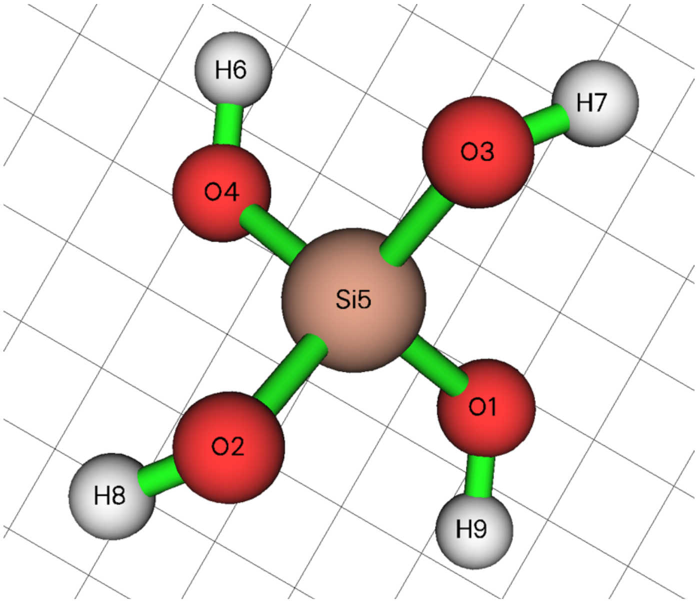

Figure S23. Multiwfn labeling of *Si1*. The OPDOS plot of Figure 13 corresponds to O2 and O3.

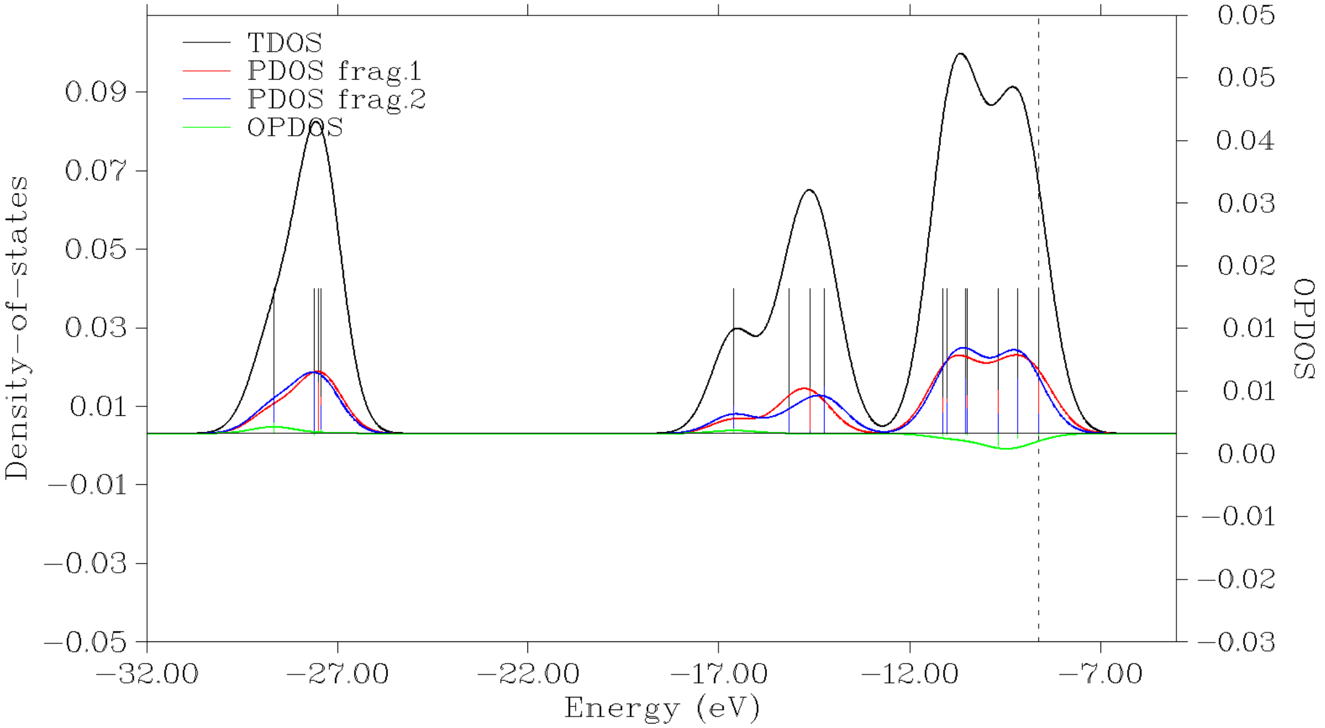

Figure S24. OPDOS analysis of O1-O2 (Multiwfn) for the *Si1* cluster.

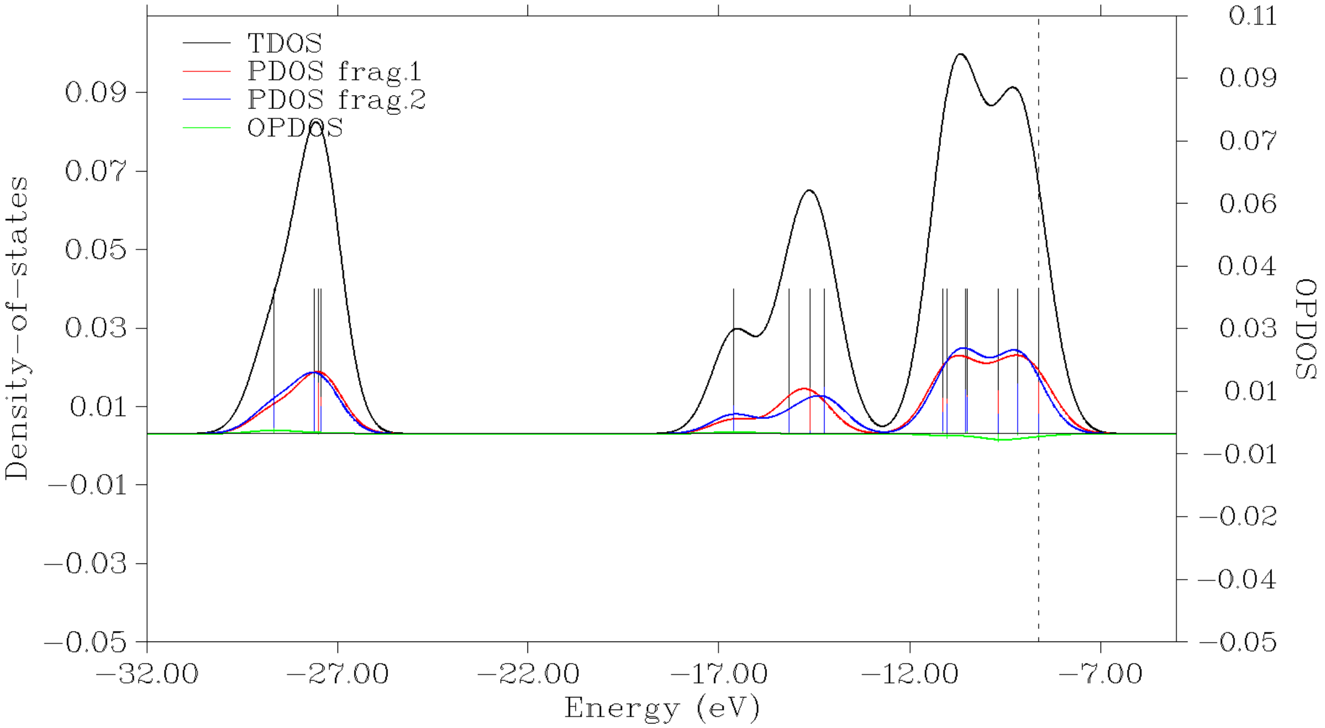

Figure S25. OPDOS analysis of O1-O3 (Multiwfn) for the *Si1* cluster.

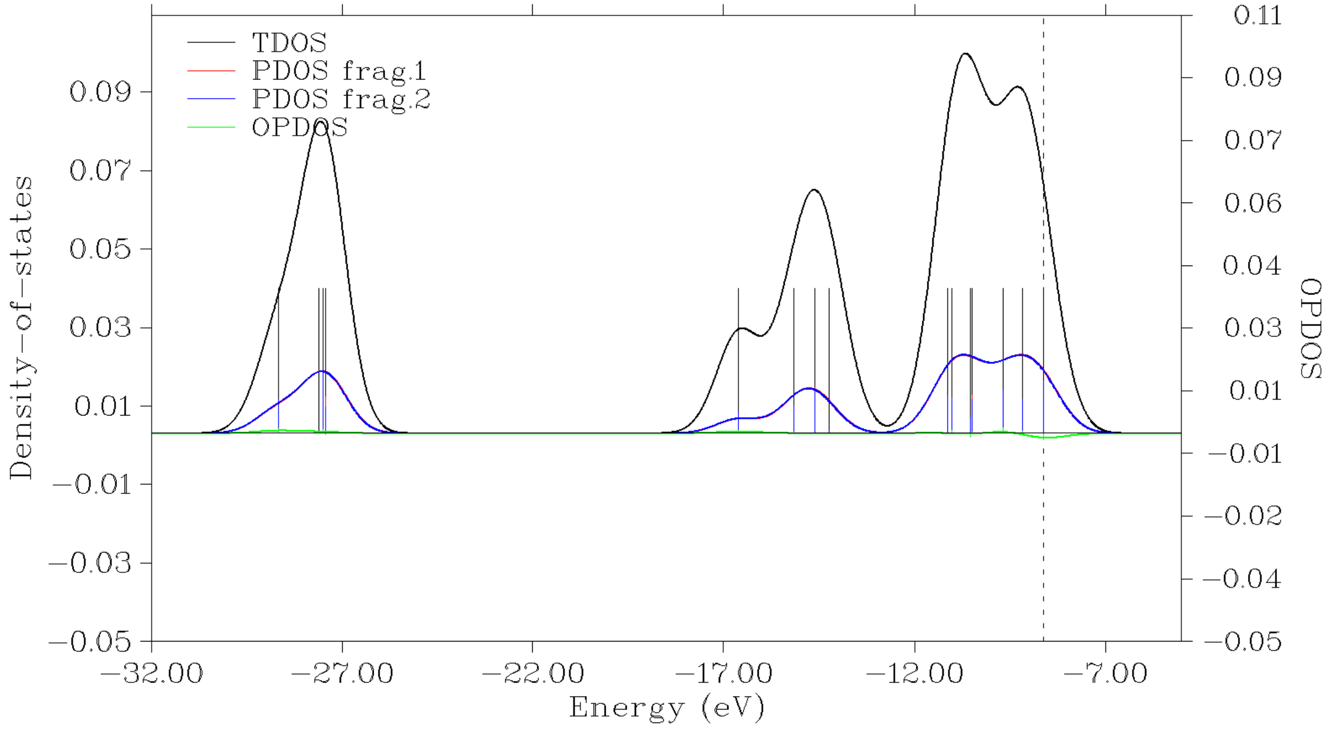

Figure S26. OPDOS analysis of O1-O4 (Multiwfn) for the *Si1* cluster.

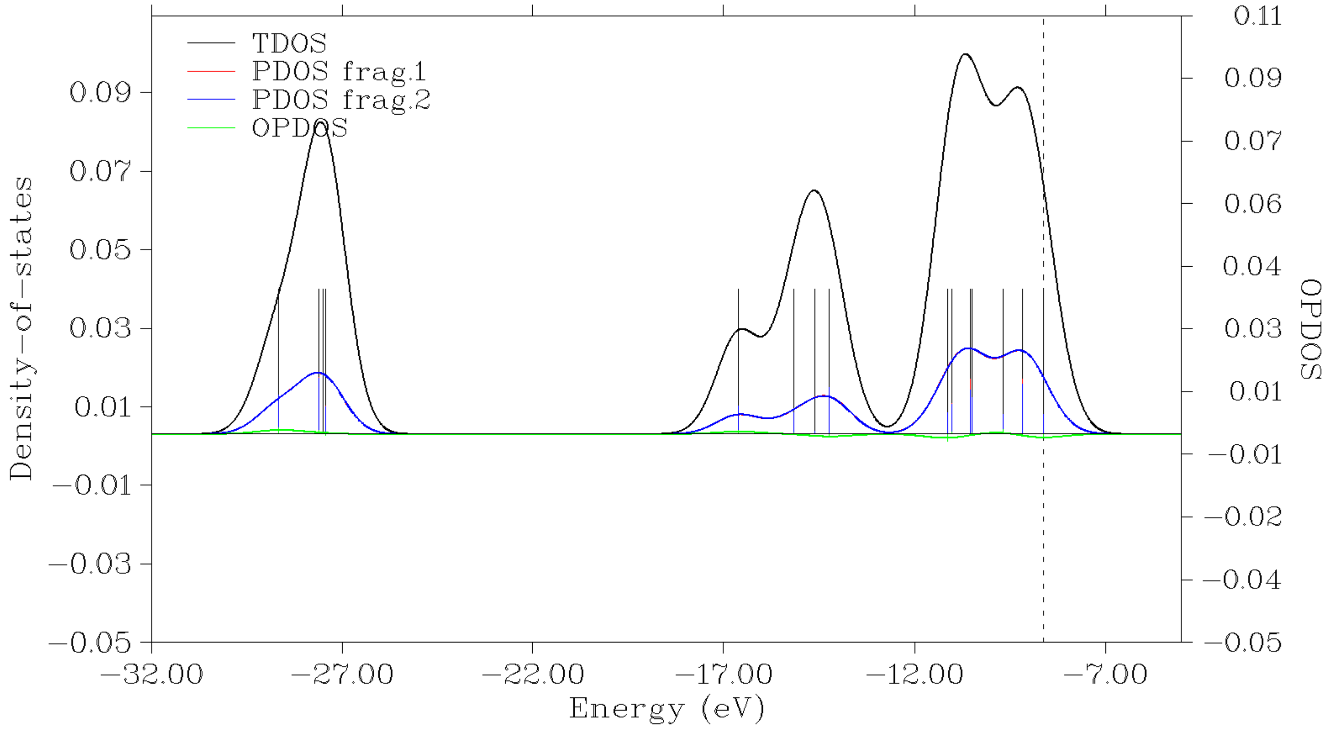

Figure S27. OPDOS analysis of O2-O3 (Multiwfn) for the *Si1* cluster.

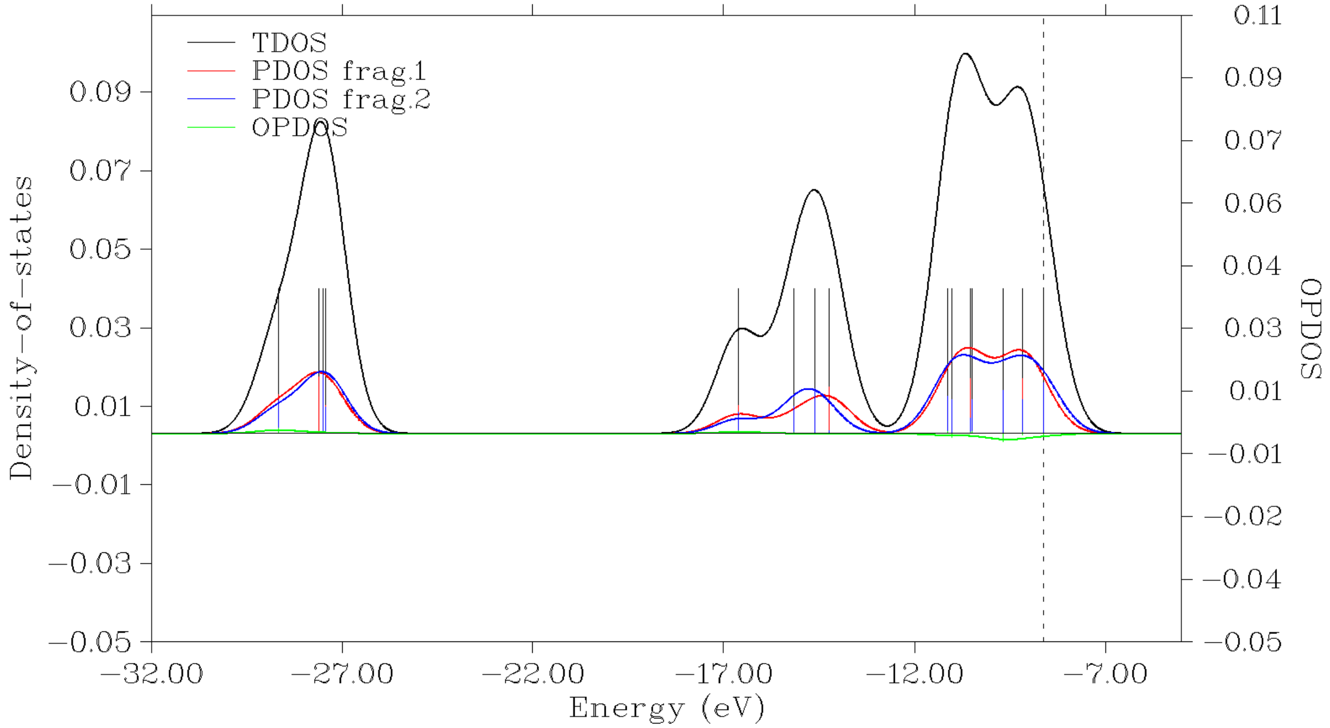

Figure S28. OPDOS analysis of O2-O4 (Multiwfn) for the *Si1* cluster.

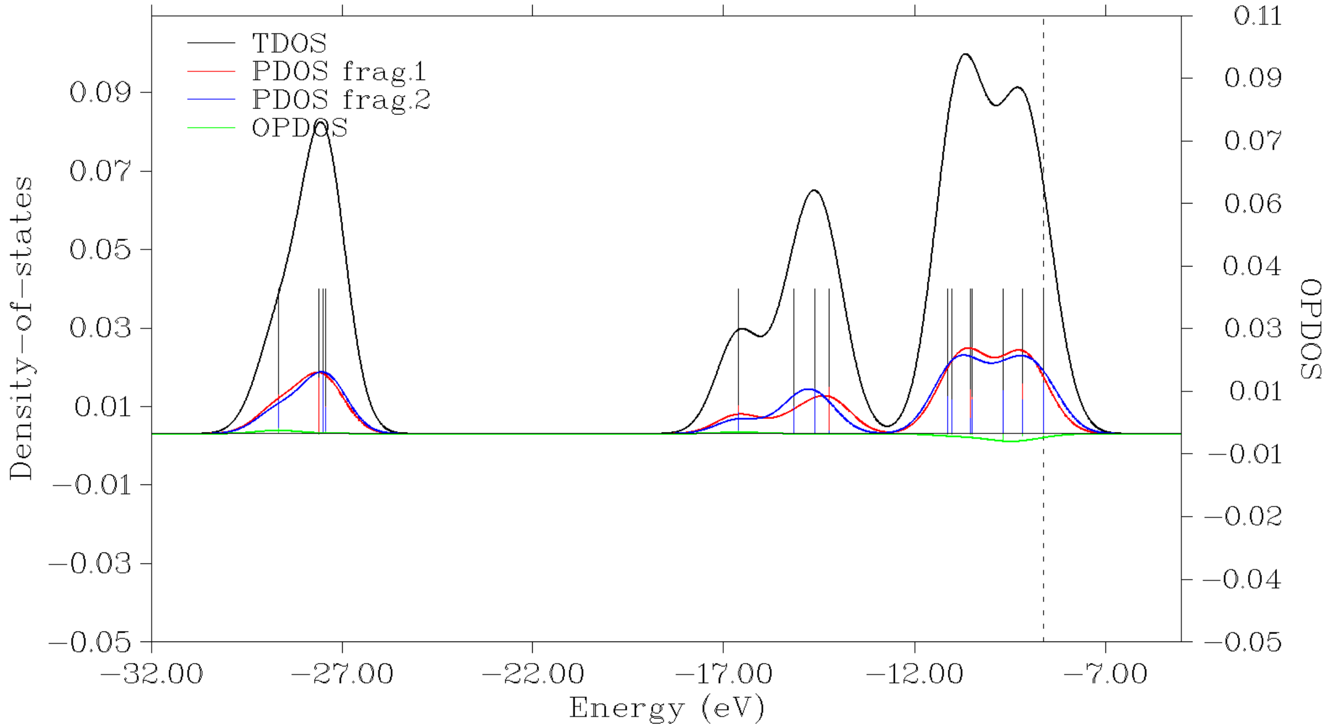

Figure S29. OPDOS analysis of O3-O4 (Multiwfn) for the *Si1* cluster.

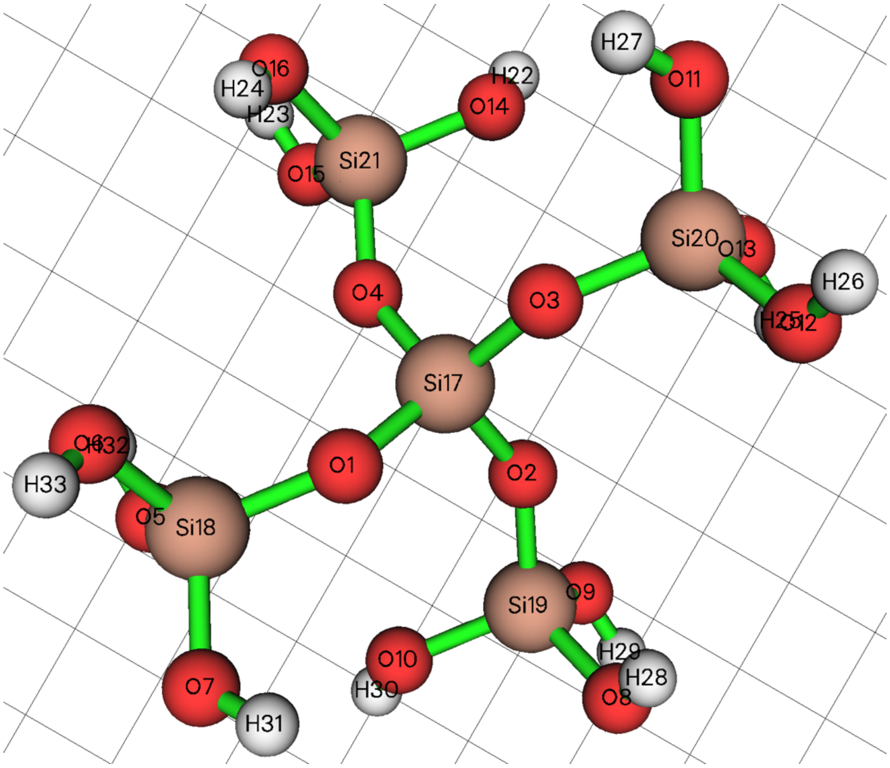

Figure S30. Multiwfn labeling of *Si5*. The OPDOS plot of Figure 13 corresponds to O1 and O3.

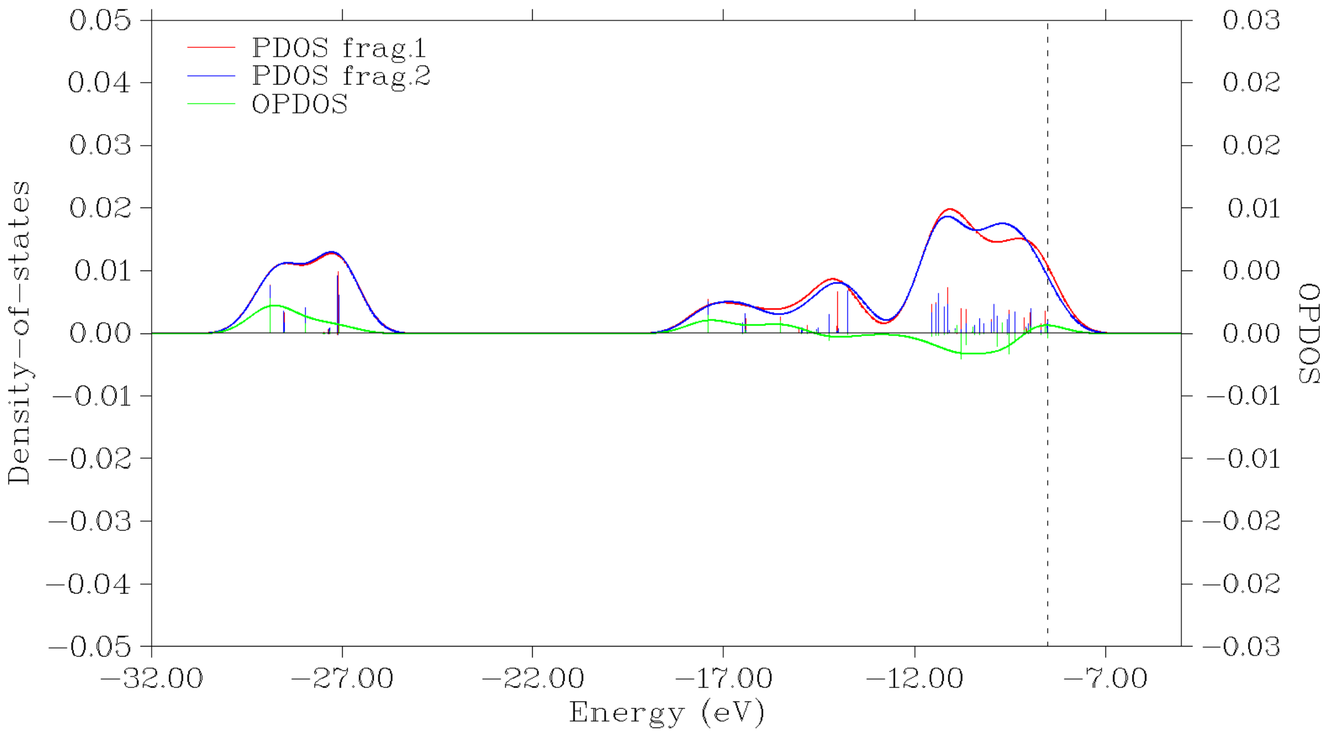

Figure S31. OPDOS analysis of O1-O2 (Multiwfn) for the *Si5* cluster.

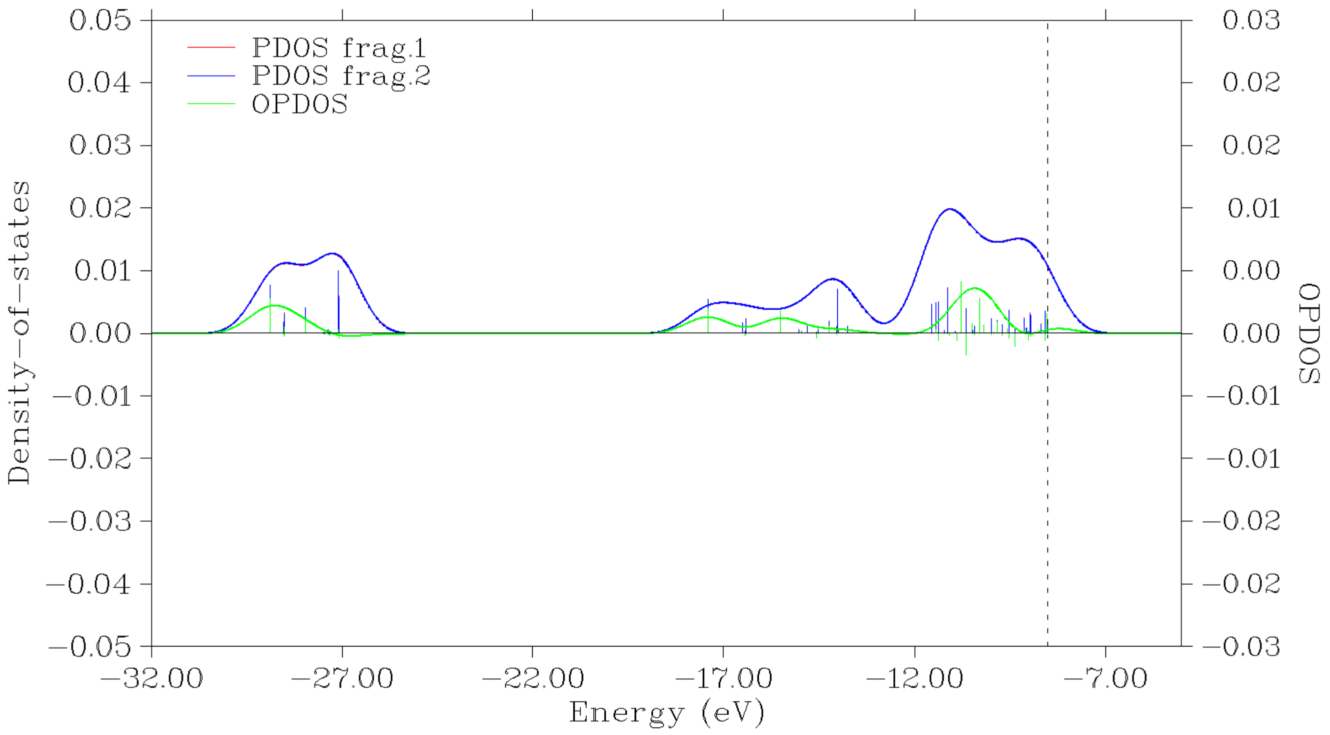

Figure S32. OPDOS analysis of O1-O3 (Multiwfn) for the *Si5* cluster.

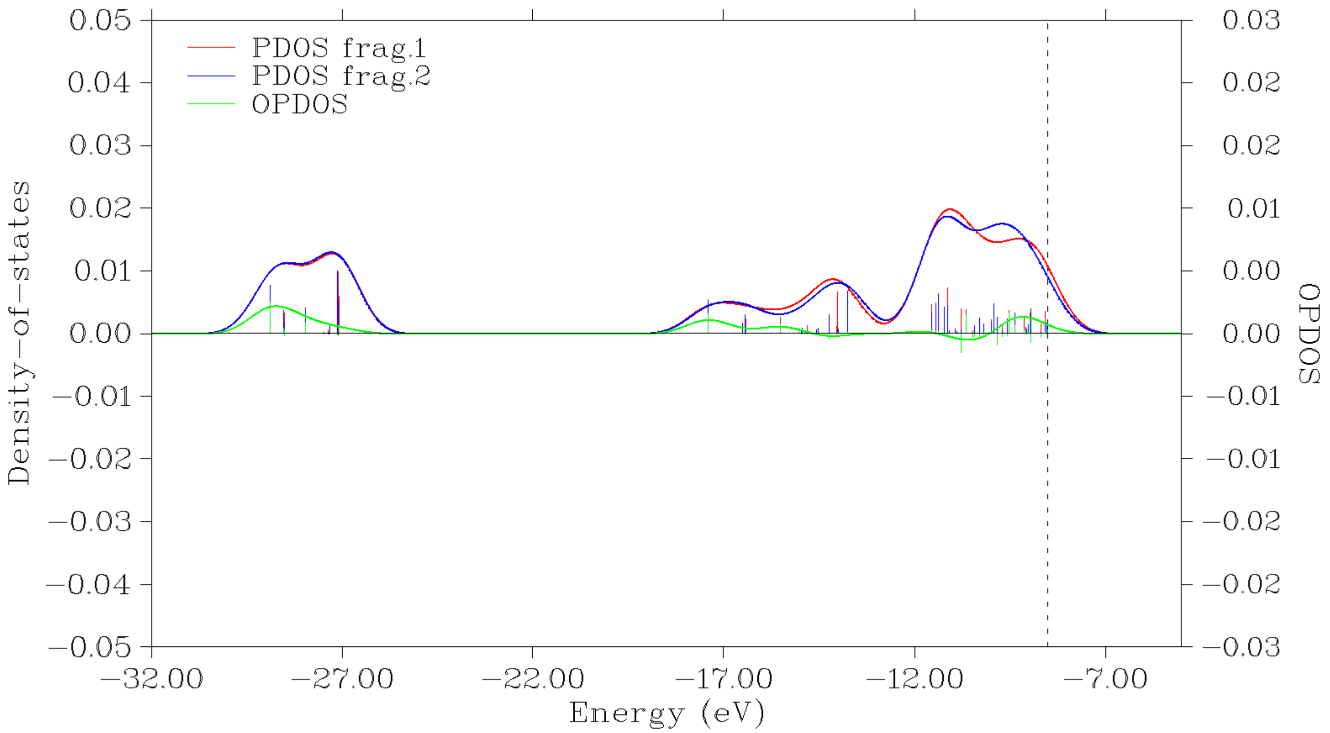

Figure S33. OPDOS analysis of O1-O4 (Multiwfn) for the *Si5* cluster.

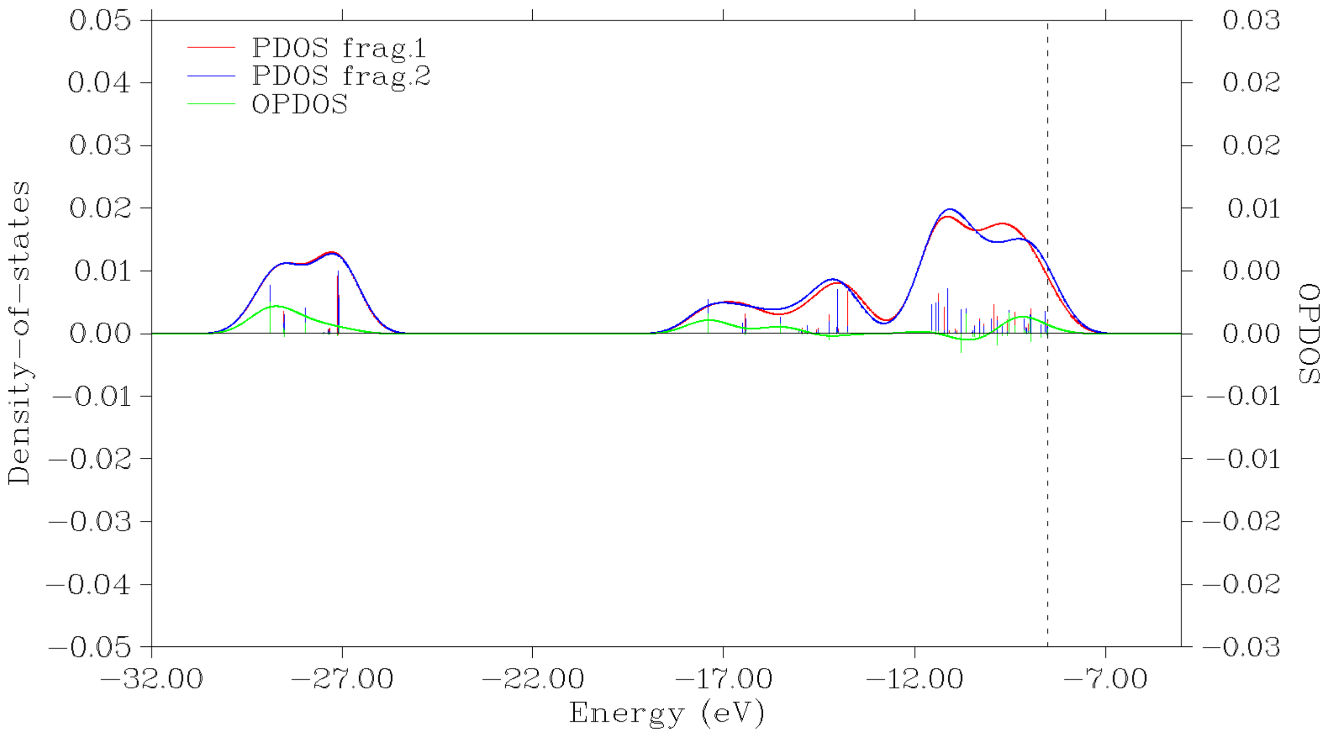

Figure S34. OPDOS analysis of O2-O3 (Multiwfn) for the *Si5* cluster.

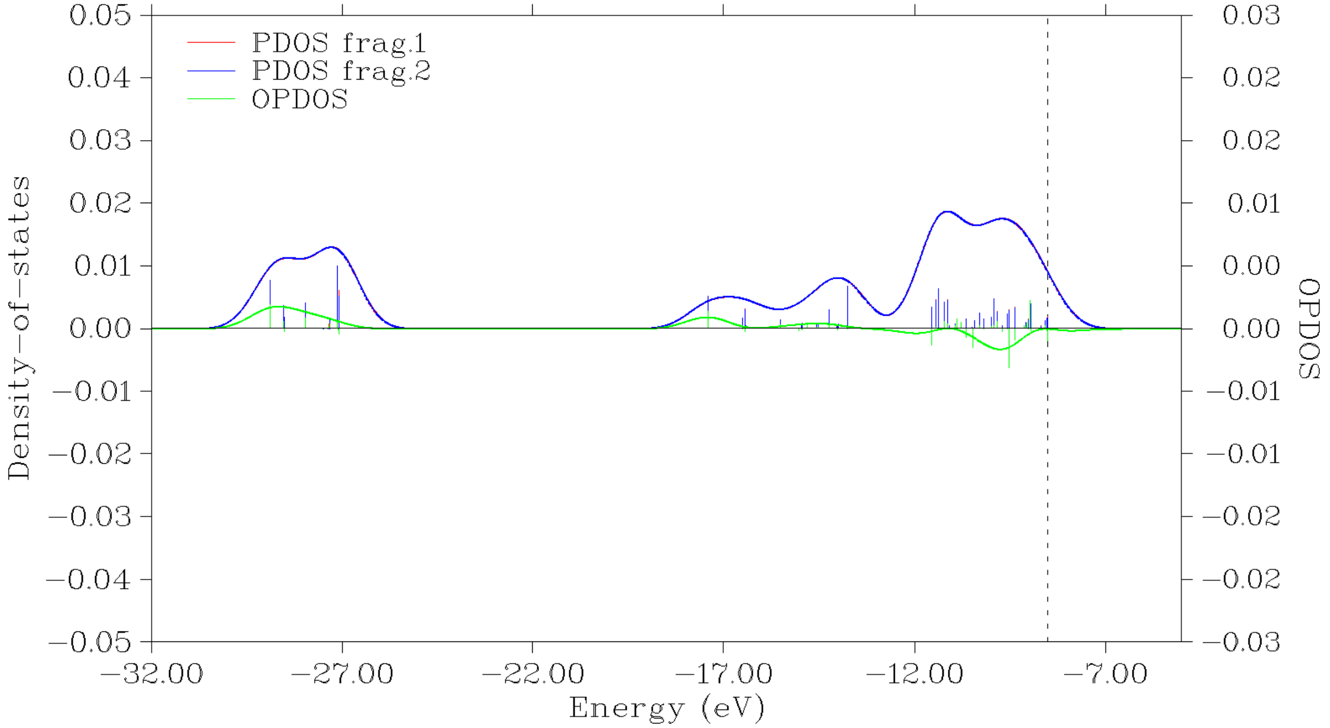

Figure S35. OPDOS analysis of O2-O4 (Multiwfn) for the *Si5* cluster.

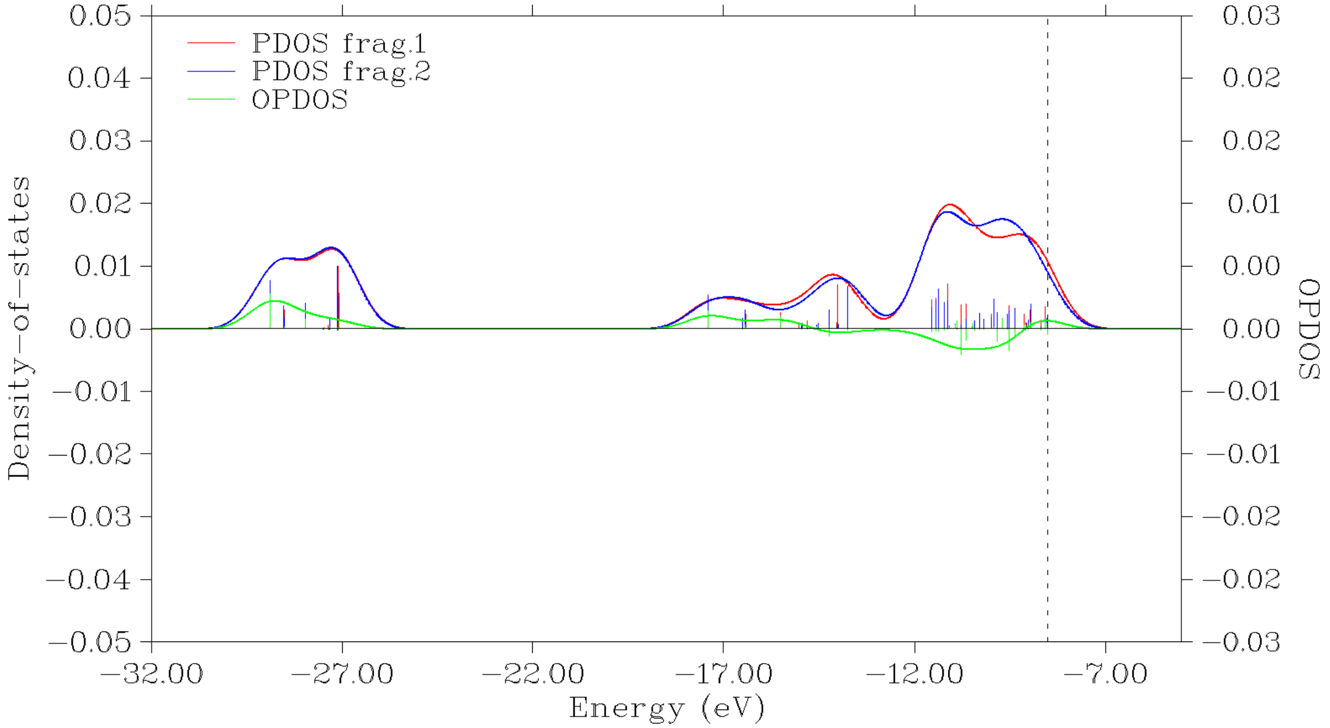

Figure S36. OPDOS analysis of O3-O4 (Multiwfn) for the *Si5* cluster.

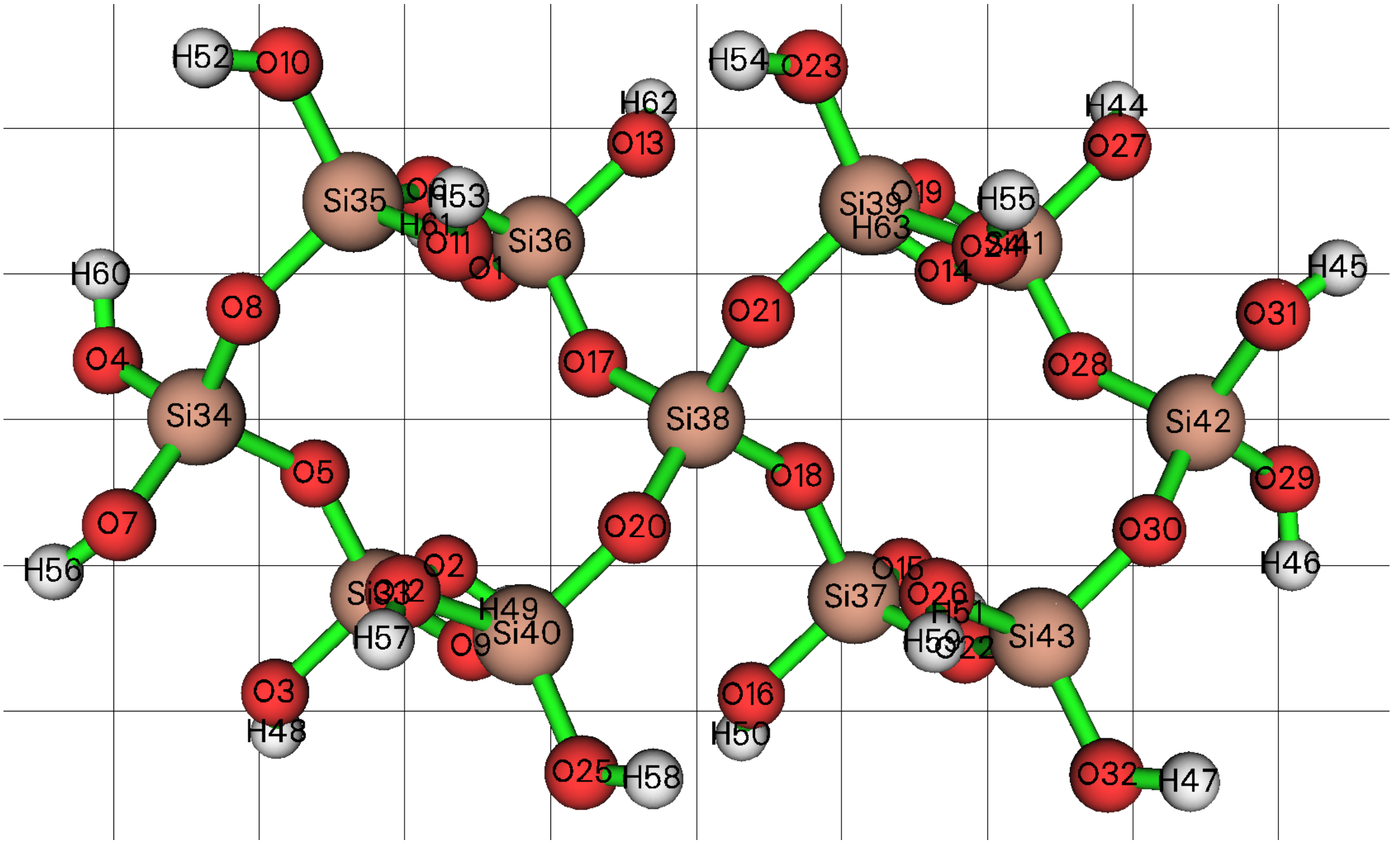

Figure S37. Multiwfn labeling of *Si11*. The OPDOS plot of Figure 13 corresponds to O20 and O21.

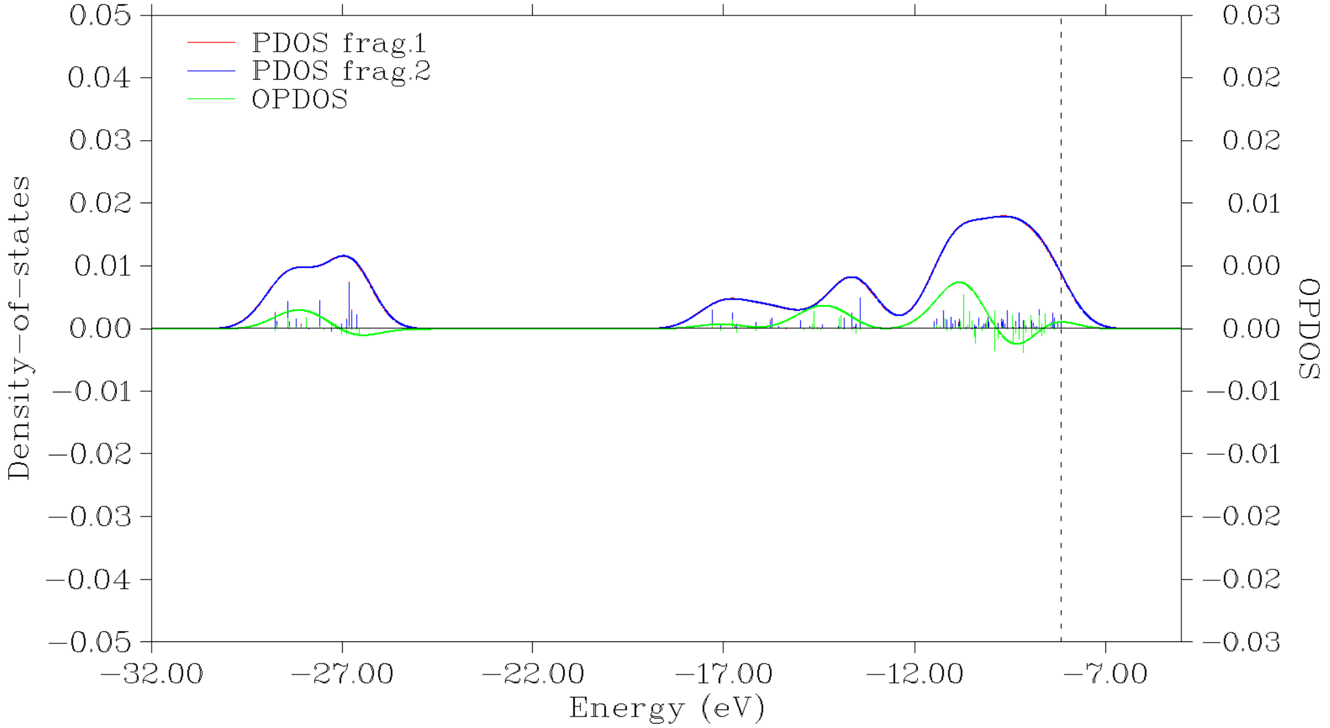

Figure S38. OPDOS analysis of O17-O18 (Multiwfn) for the *Si11* cluster.

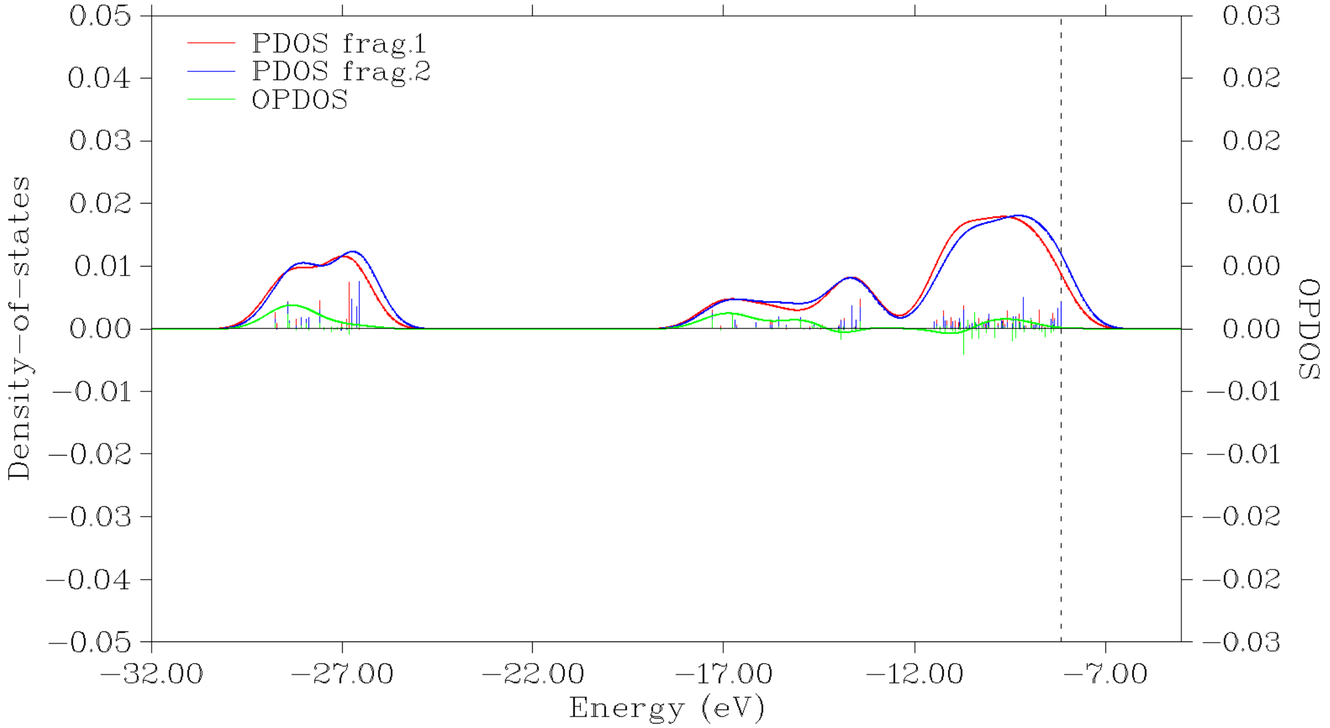

Figure S39. OPDOS analysis of O17-O20 (Multiwfn) for the *Si11* cluster.

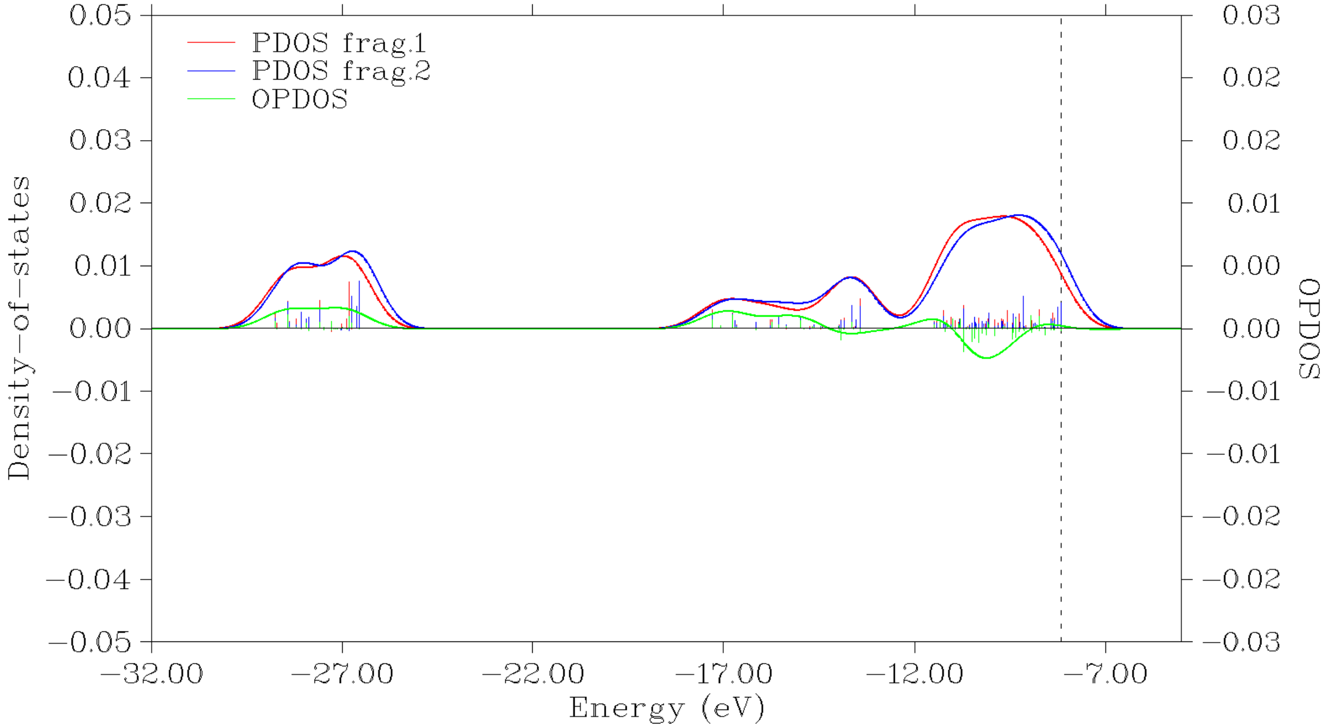

Figure S40. OPDOS analysis of O17-O21 (Multiwfn) for the *Si11* cluster.

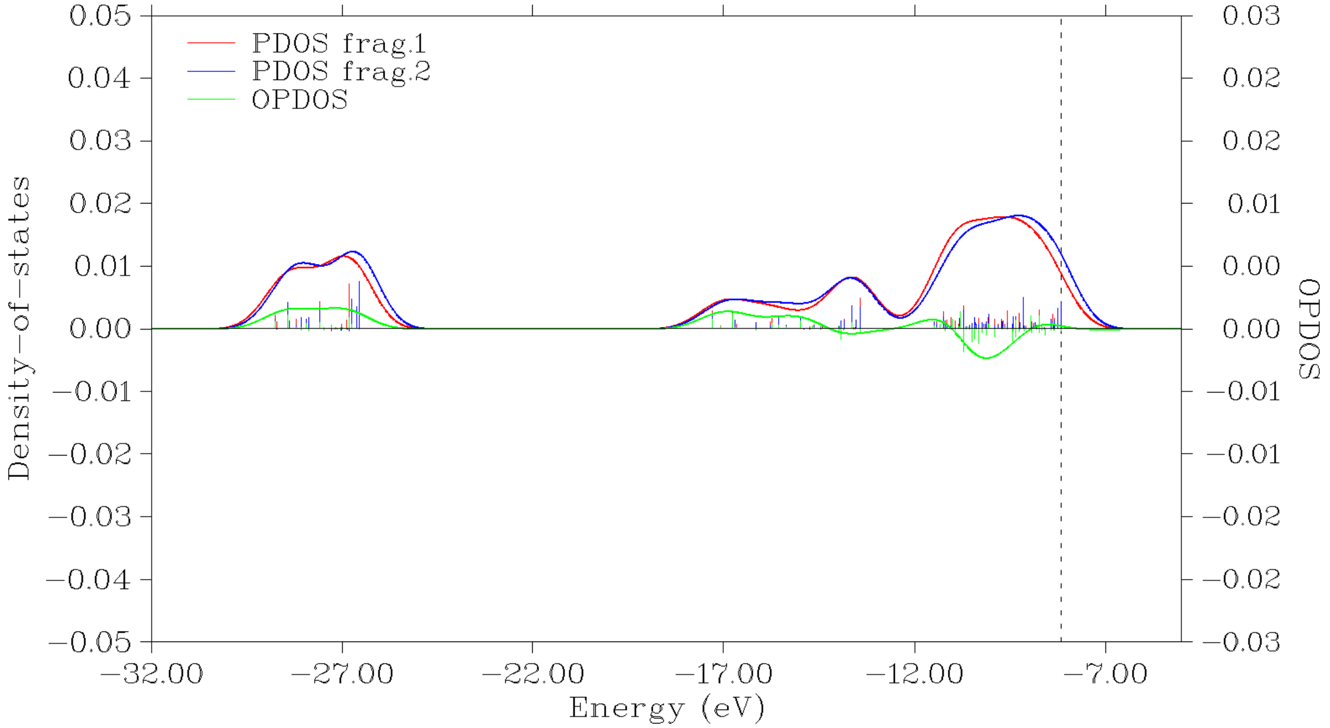

Figure S41. OPDOS analysis of O18-O20 (Multiwfn) for the *Si11* cluster.

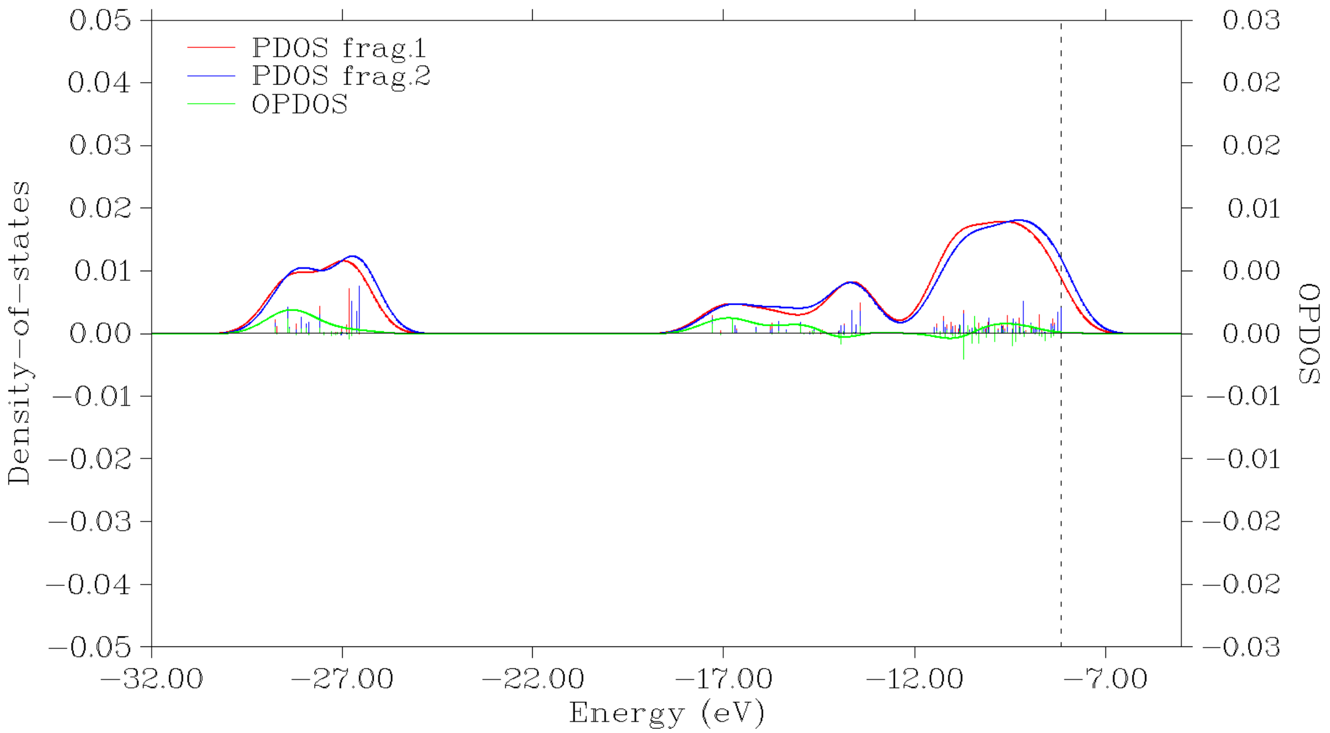

Figure S42. OPDOS analysis of O18-O21 (Multiwfn) for the *Si11* cluster.

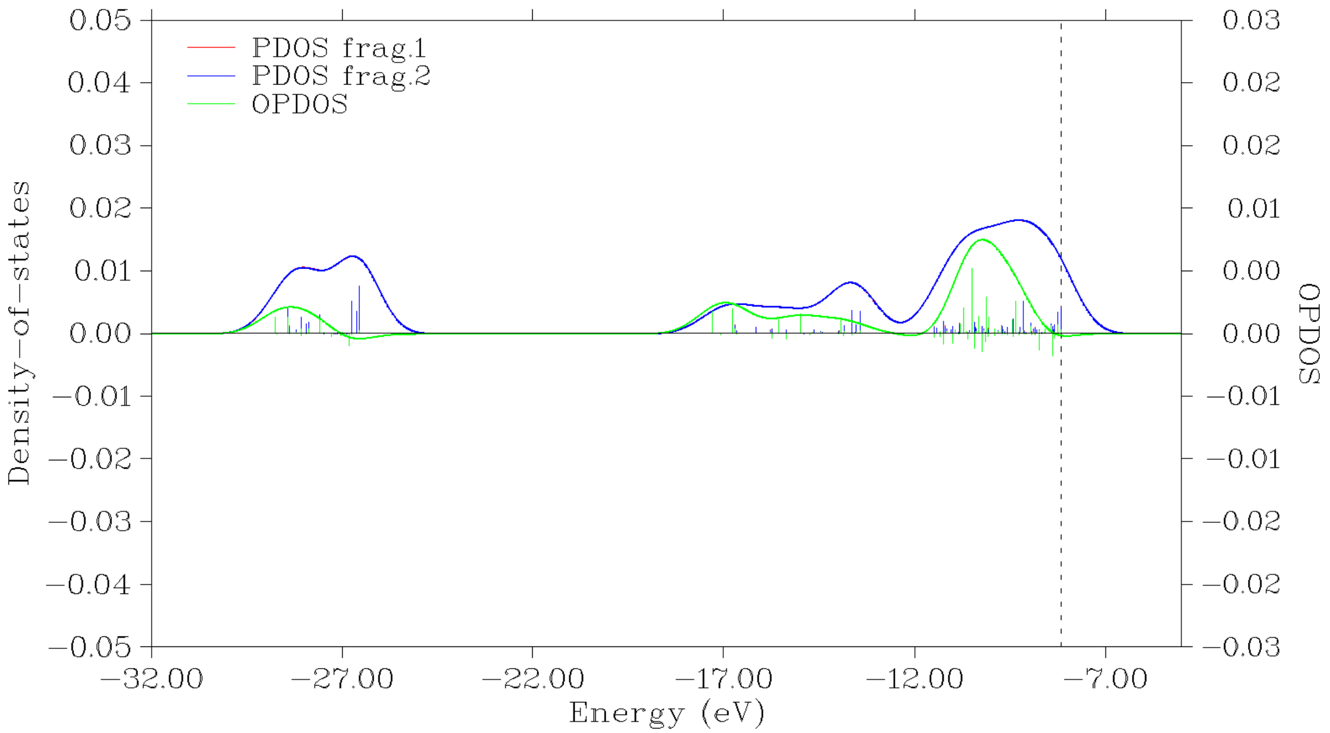

Figure S43. OPDOS analysis of O20-O21 (Multiwfn) for the *Si11* cluster.

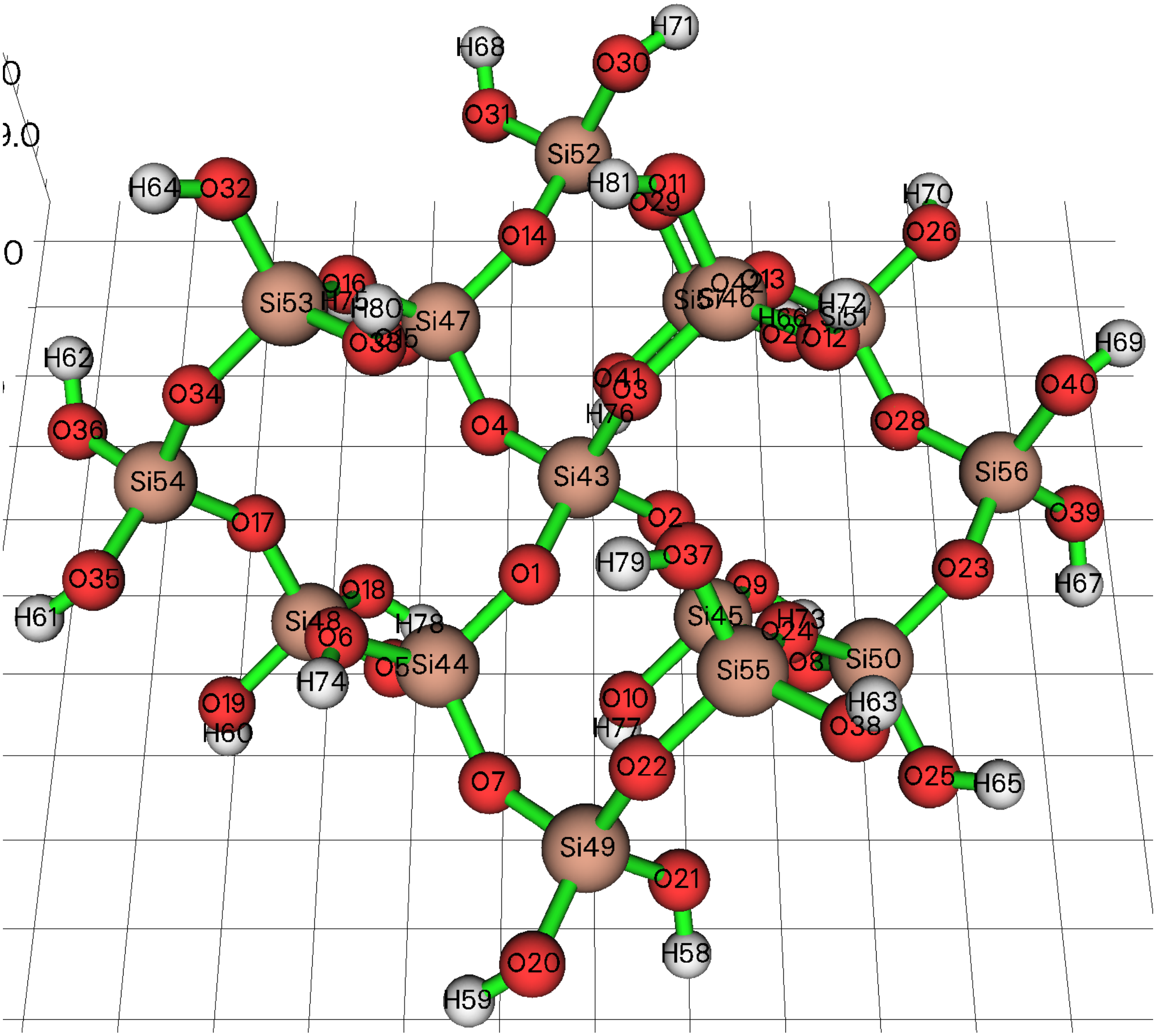

Figure S44. Multiwfn labeling of *Si15*. The OPDOS plot of Figure 13 corresponds to O1 and O3.

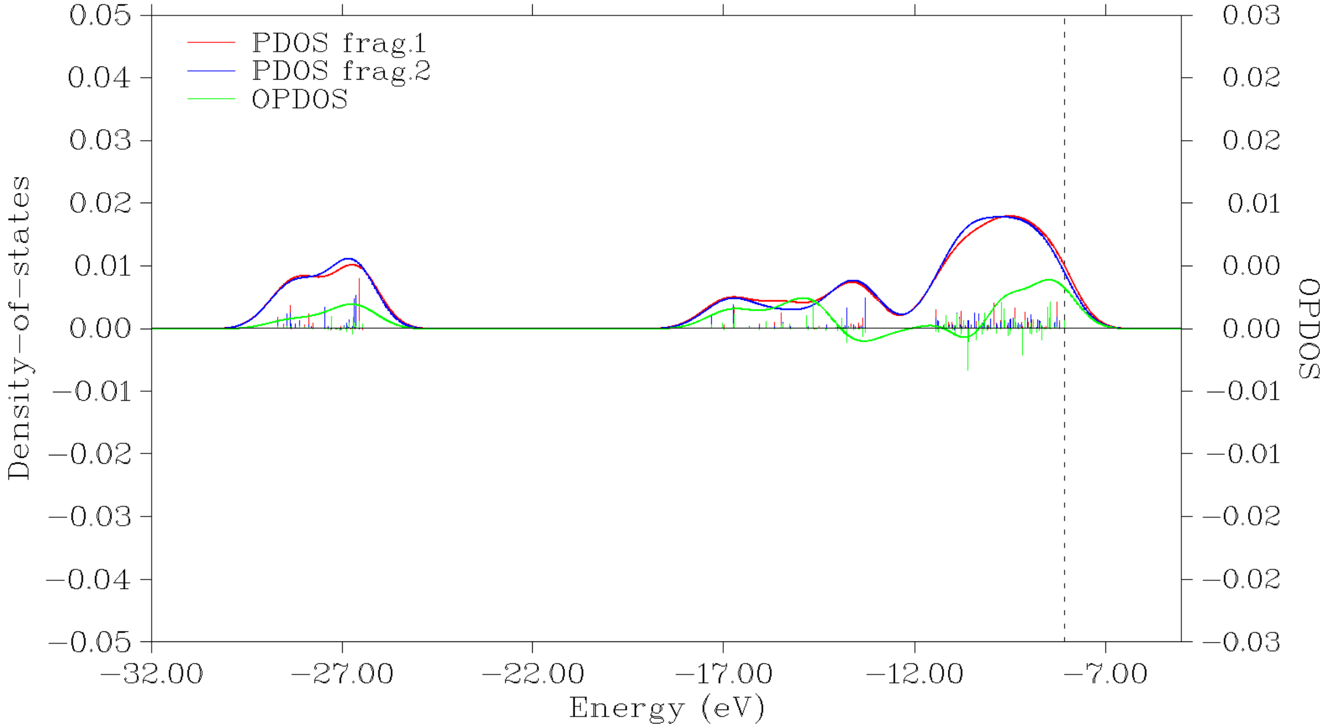

Figure S45. OPDOS analysis of O1-O2 (Multiwfn) for the *Si15* cluster.

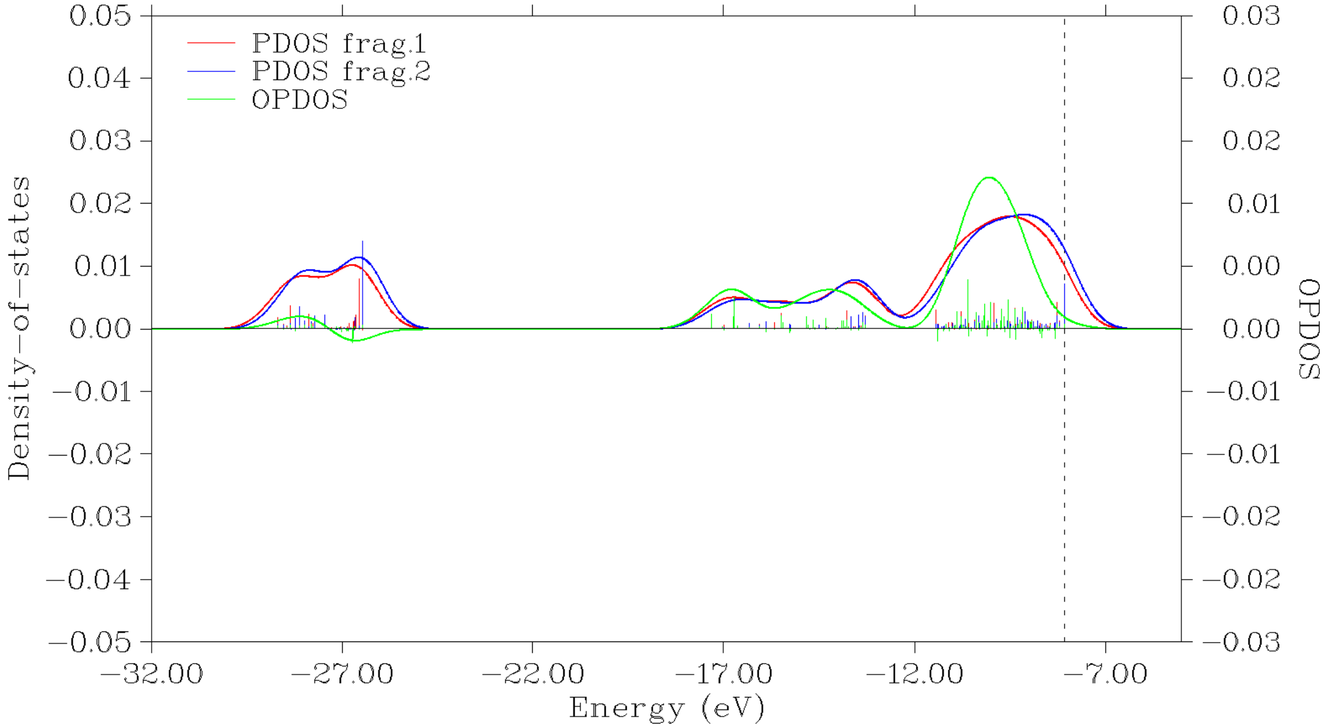

Figure S46. OPDOS analysis of O1-O3 (Multiwfn) for the *Si15* cluster.

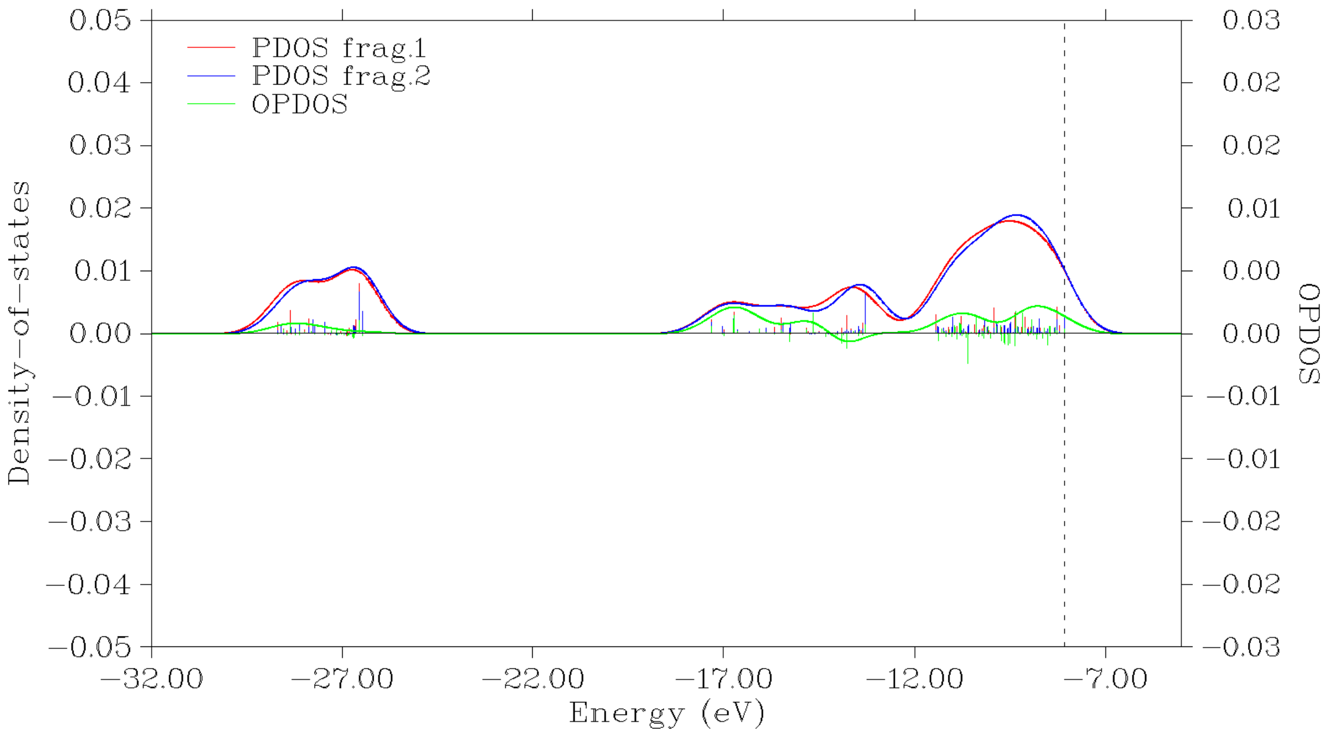

Figure S47. OPDOS analysis of O1-O4 (Multiwfn) for the *Si15* cluster.

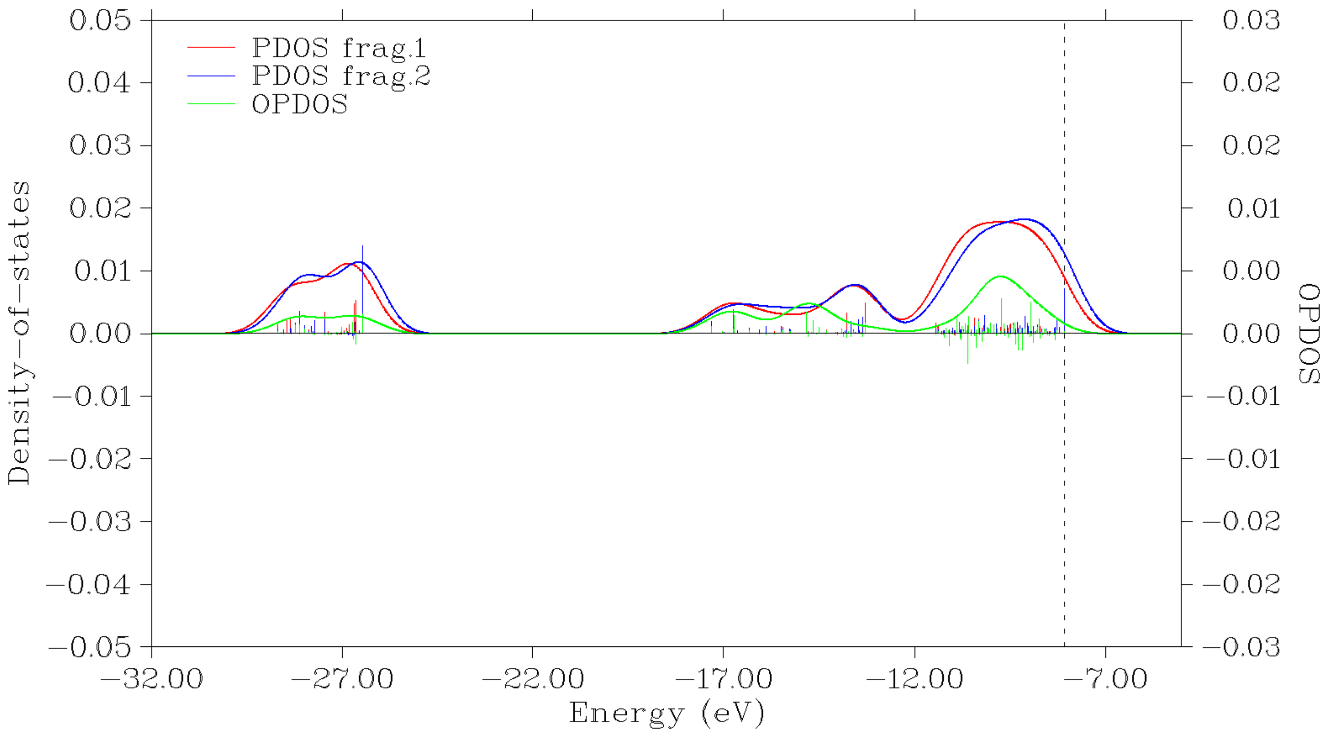

Figure S48. OPDOS analysis of O2-O3 (Multiwfn) for the *Si15* cluster.

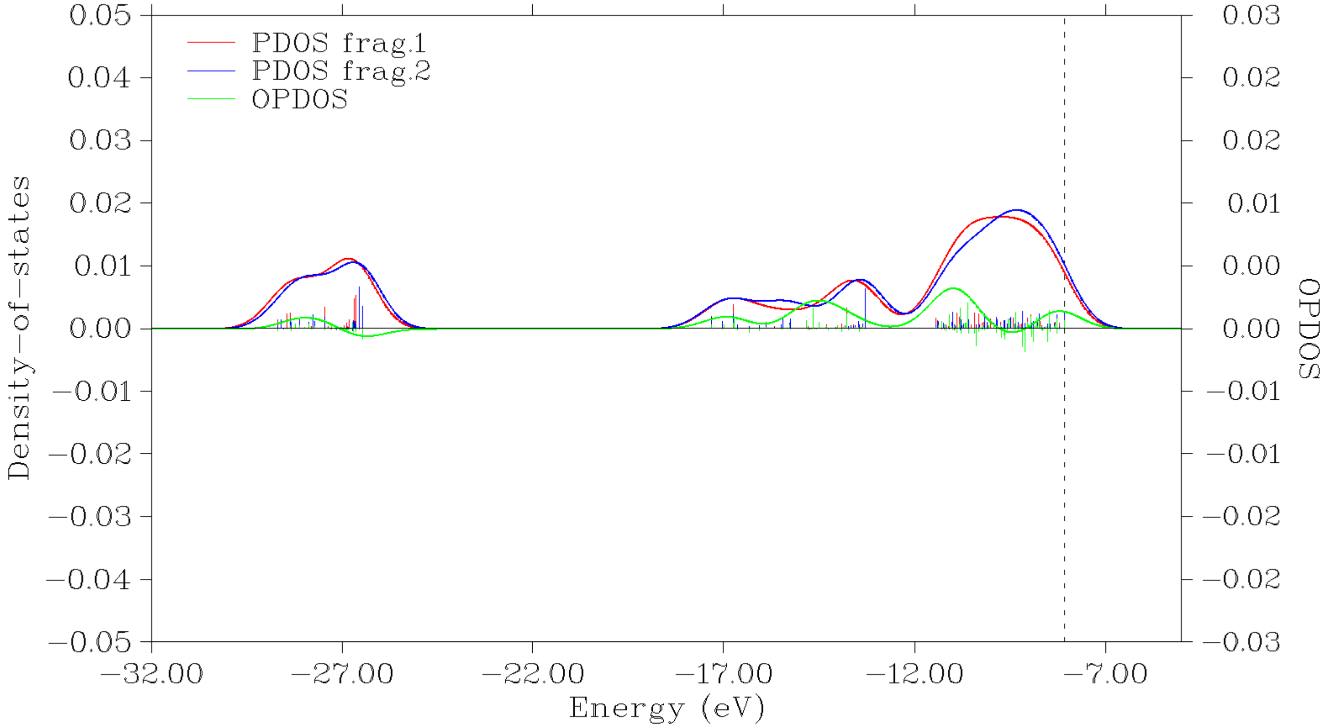

Figure S49. OPDOS analysis of O2-O4 (Multiwfn) for the *Si15* cluster.

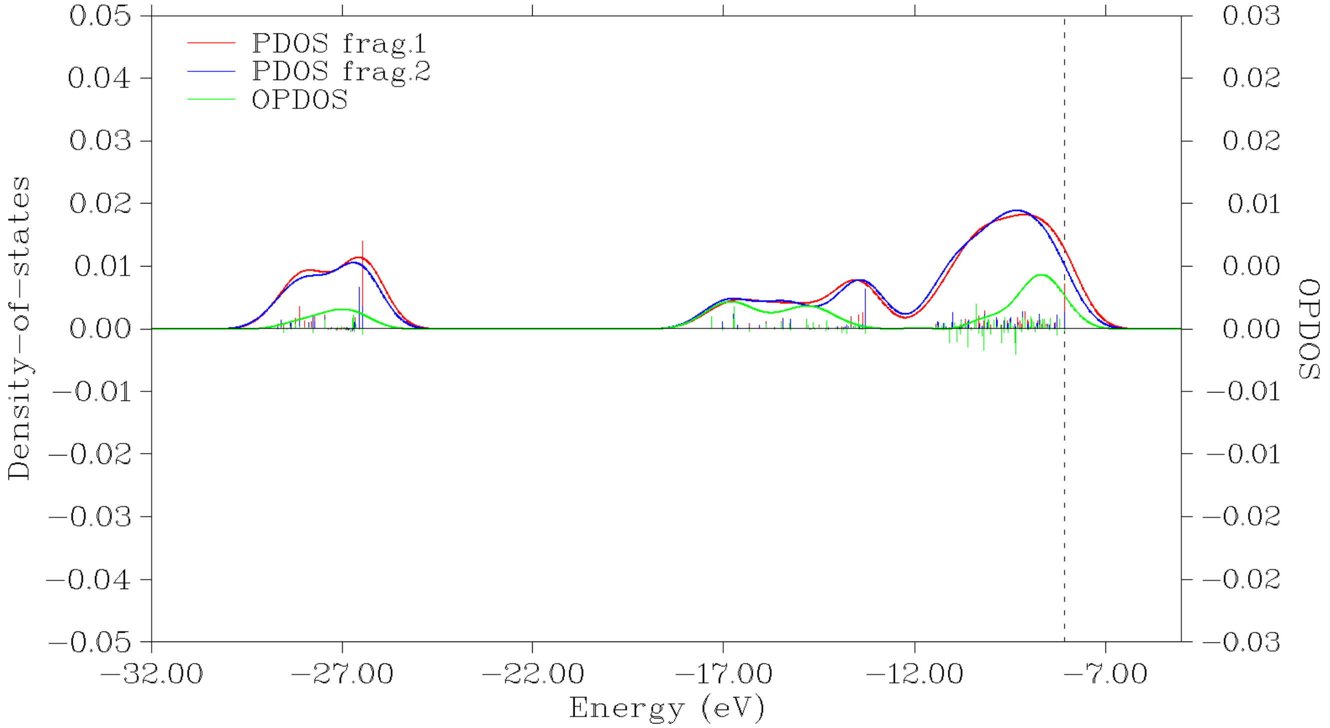

Figure S50. OPDOS analysis of O3-O4 (Multiwfn) for the *Si15* cluster.

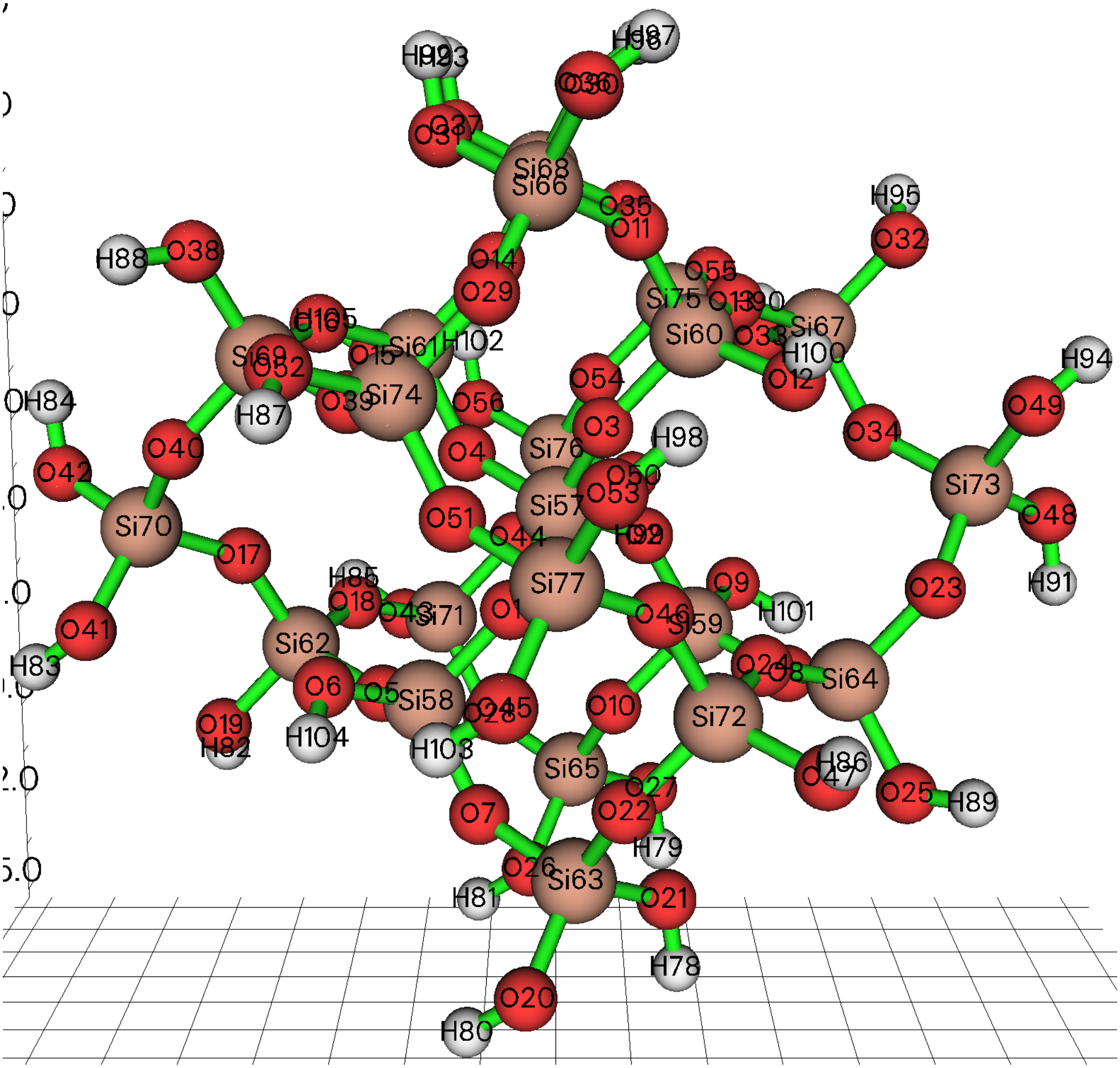

Figure S51. Multiwfn labeling of *Si21*. The OPDOS plot of Figure 13 corresponds to O1 and O3.

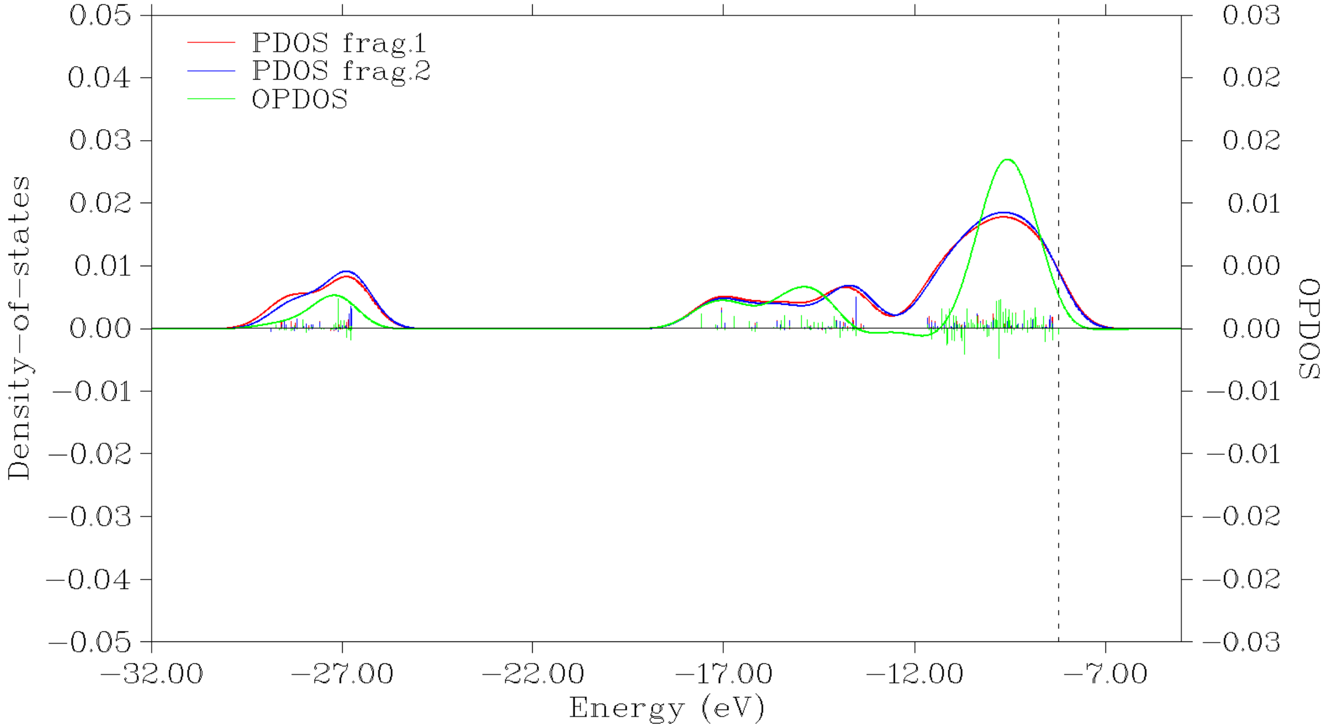

Figure S52. OPDOS analysis of O1-O2 (Multiwfn) for the *Si21*cluster.

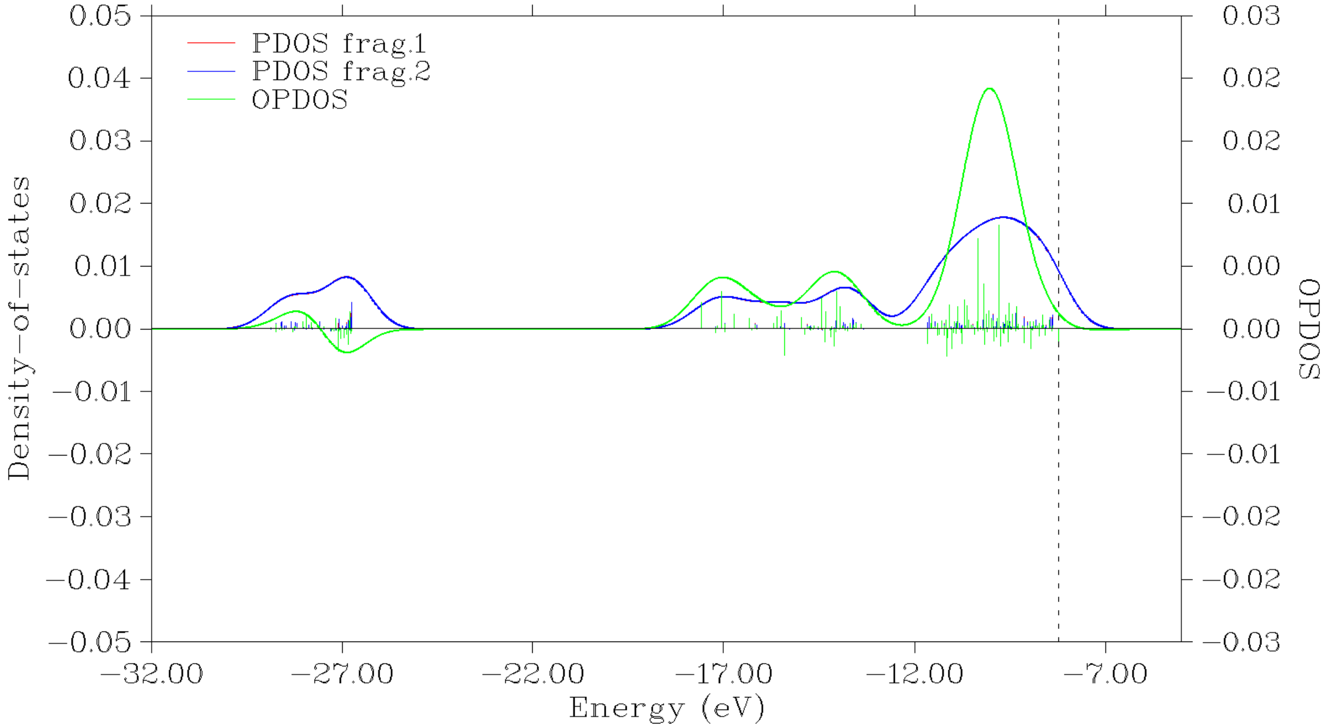

Figure S53. OPDOS analysis of O1-O3 (Multiwfn) for the *Si21* cluster.

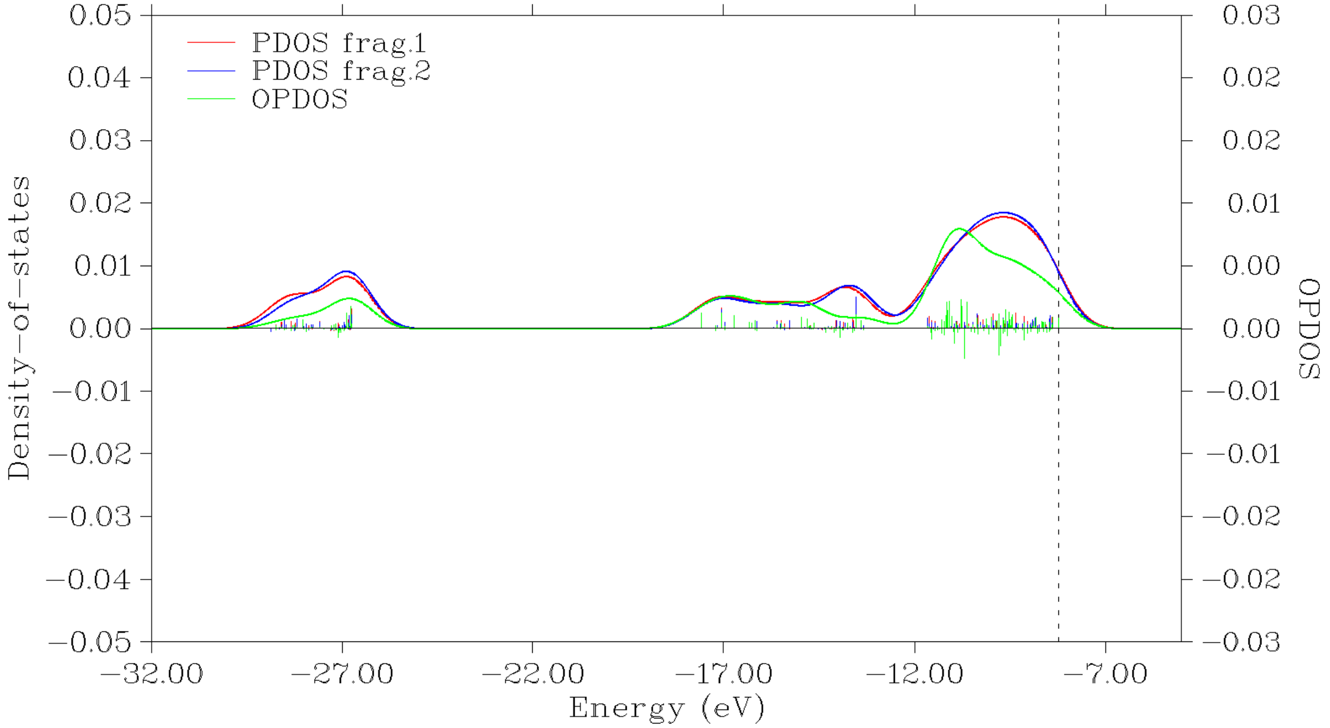

Figure S54. OPDOS analysis of O1-O4 (Multiwfn) for the *Si21* cluster.

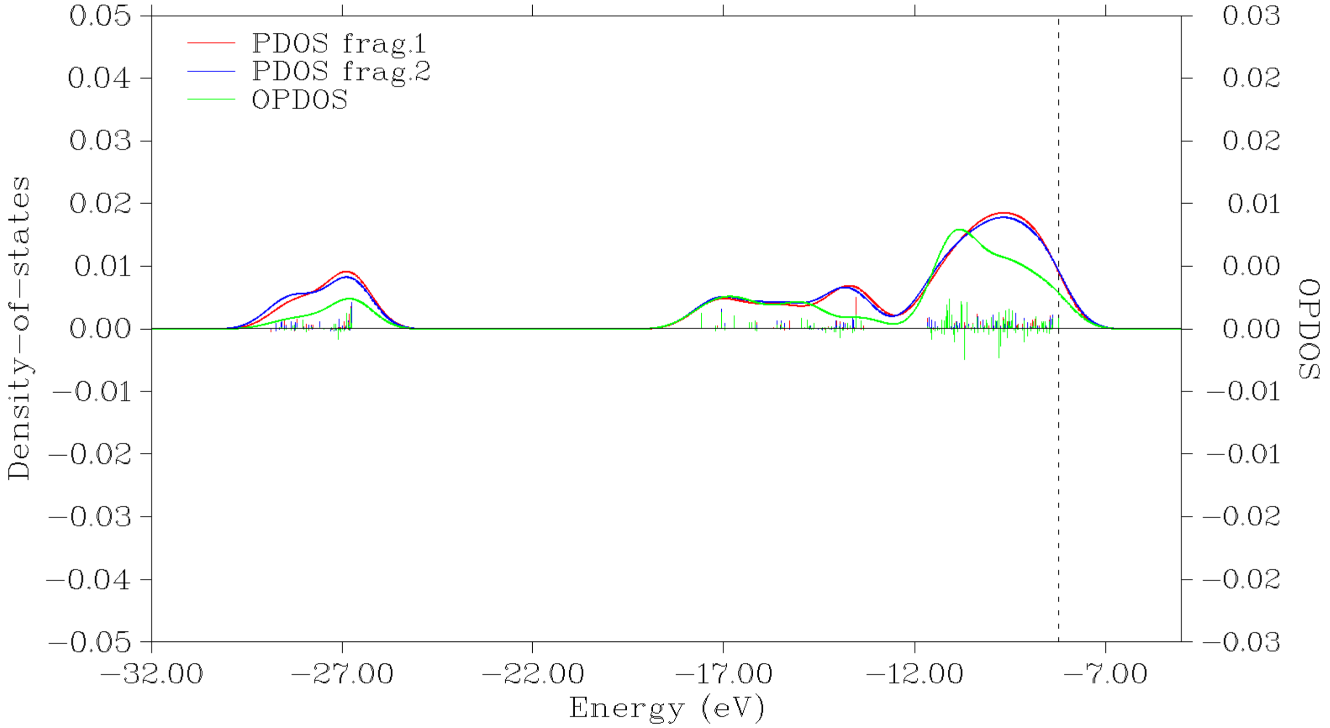

Figure S55. OPDOS analysis of O2-O3 (Multiwfn) for the *Si21*cluster.

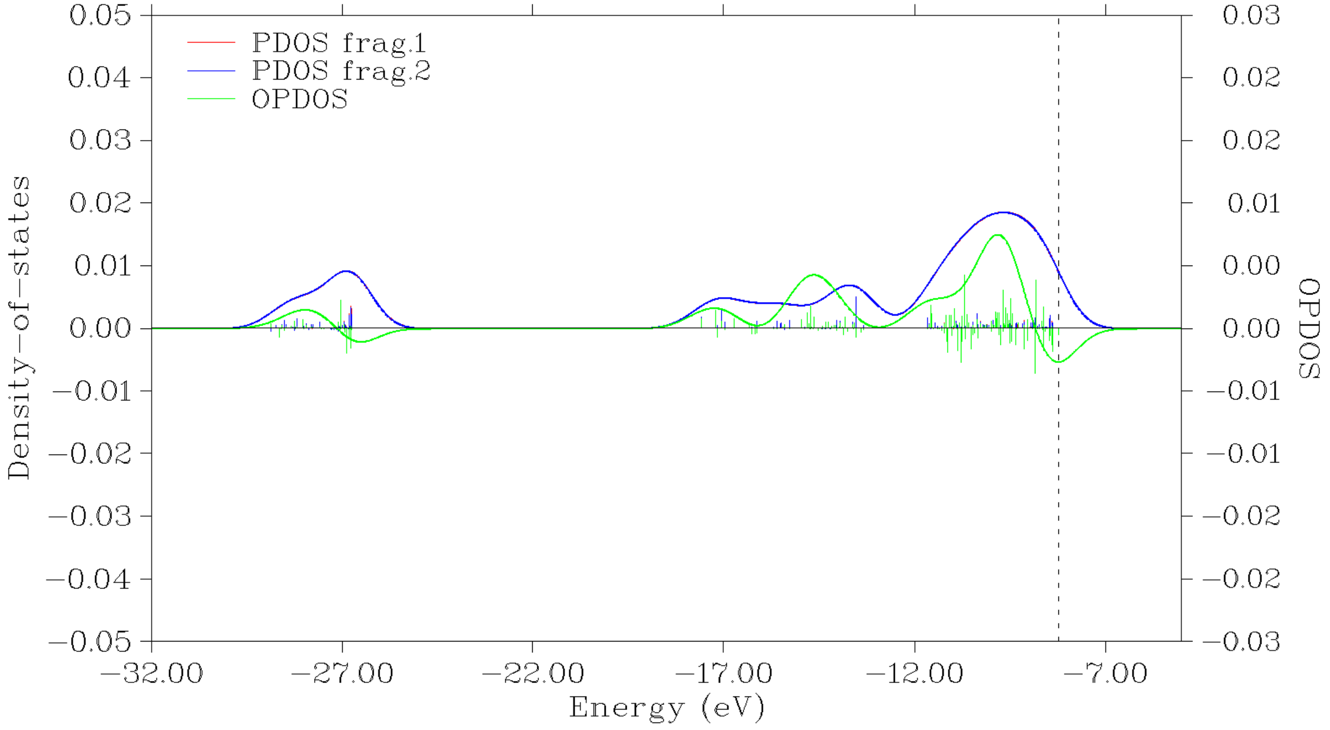

Figure S56. OPDOS analysis of O2-O4 (Multiwfn) for the *Si21* cluster.

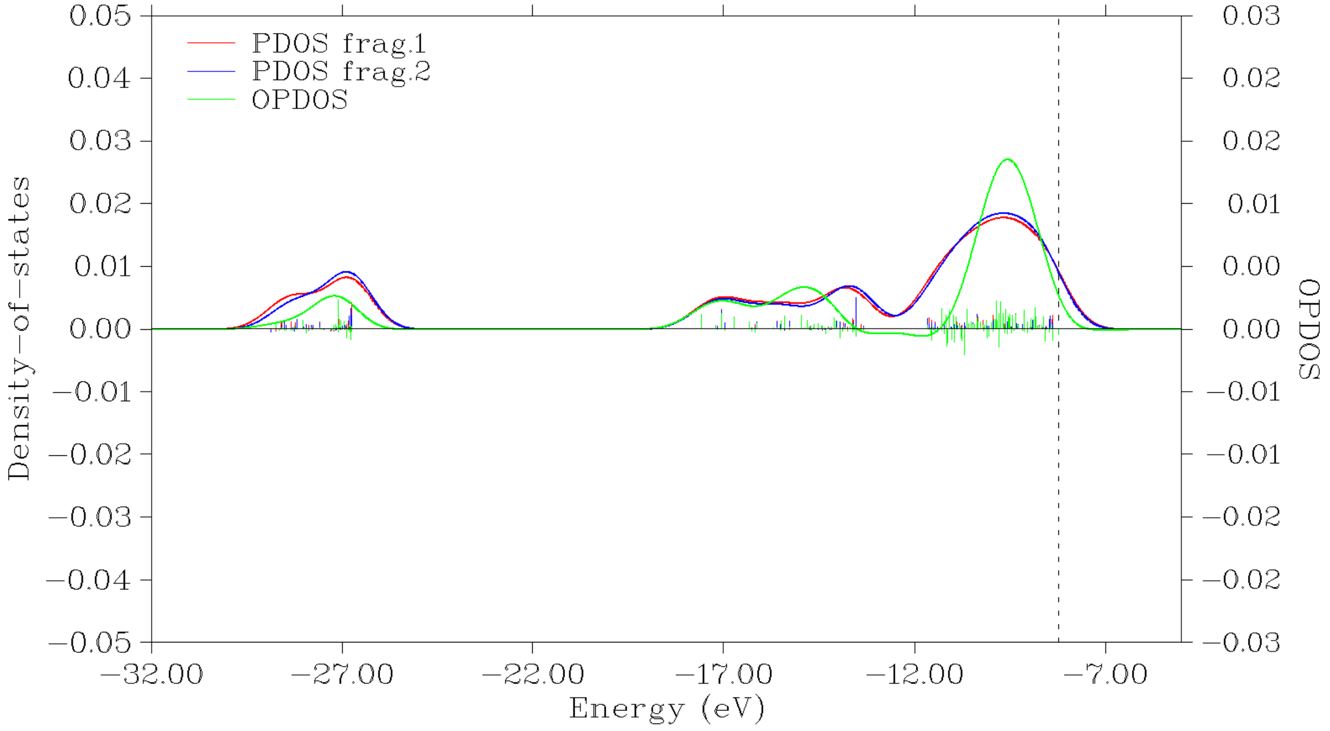

Figure S57. OPDOS analysis of O3-O4 (Multiwfn) for the *Si21* cluster.

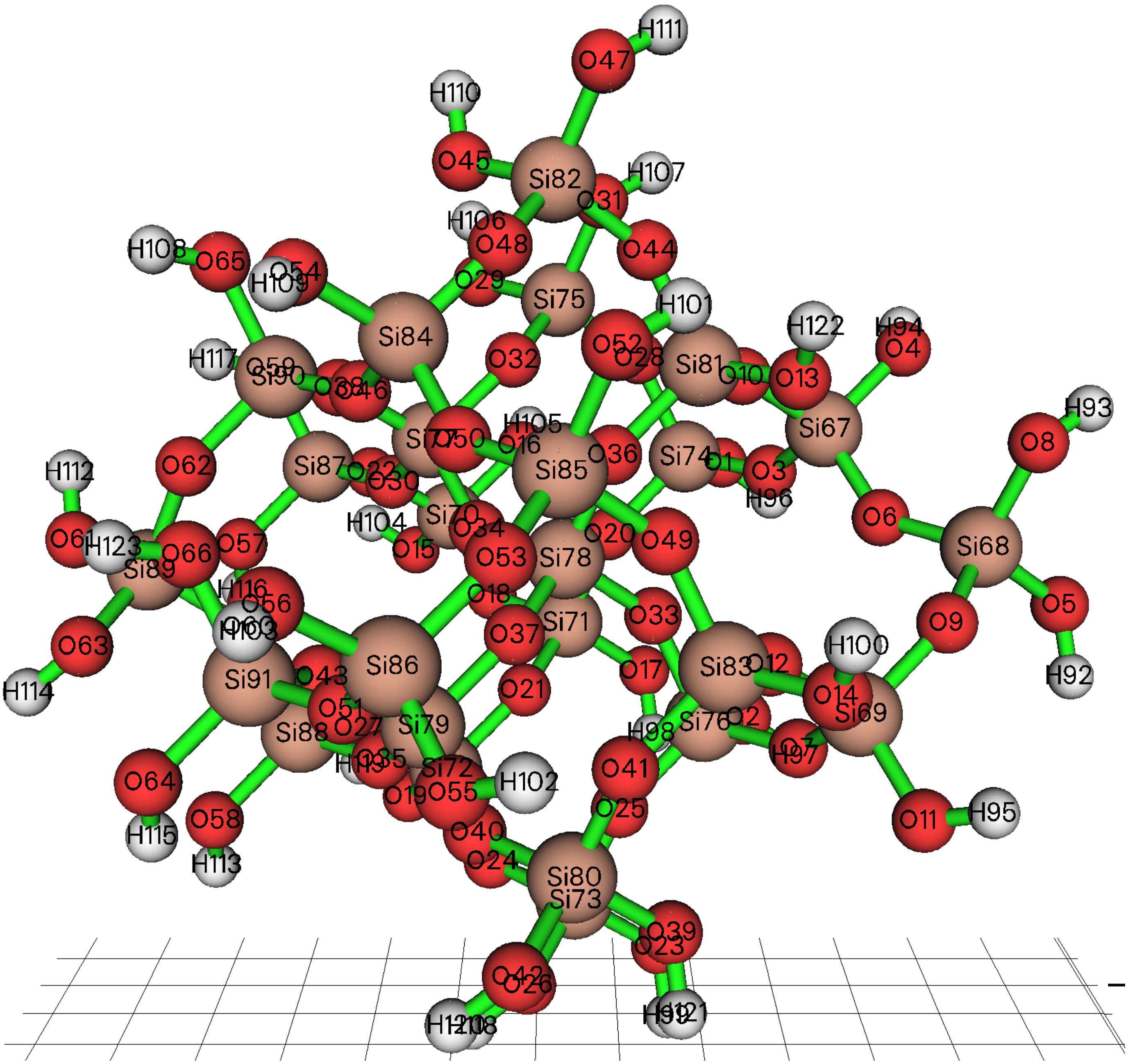

Figure S58. Multiwfn labeling of *Si25*. The OPDOS plot of Figure 13 corresponds to O36 and O37.

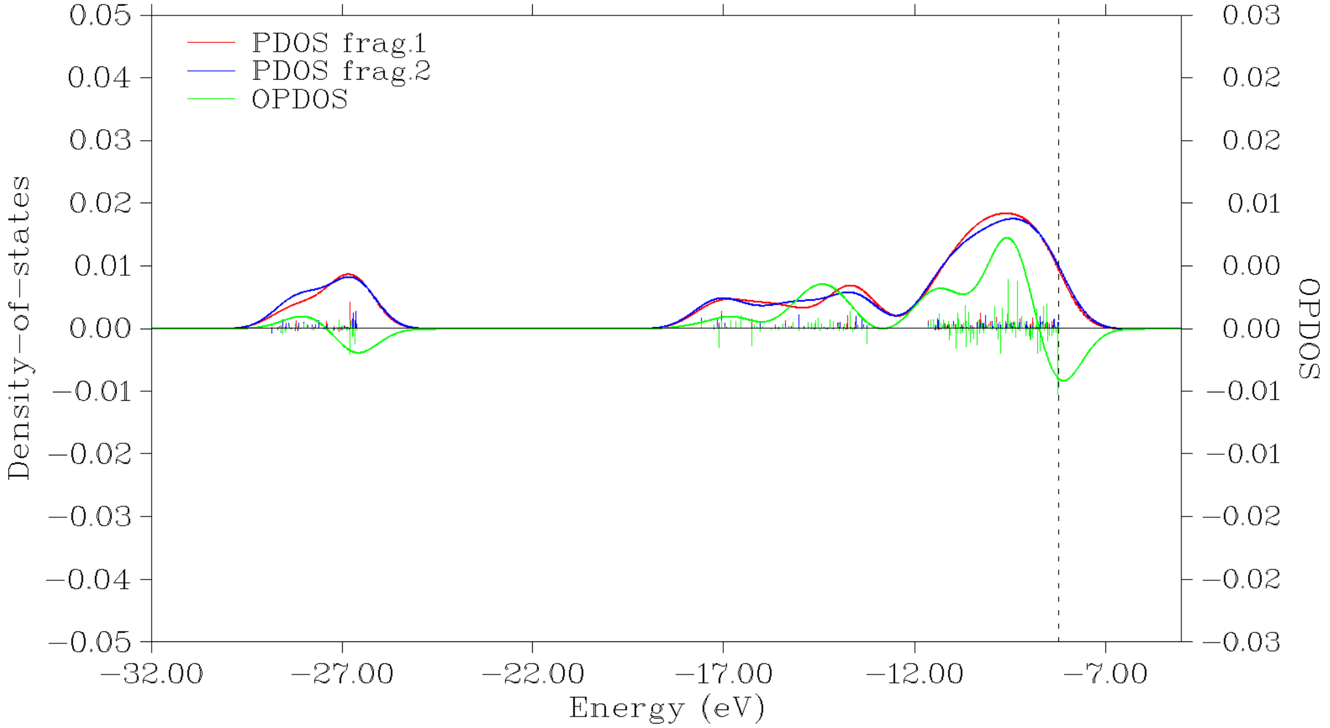

Figure S59. OPDOS analysis of O33-O34 (Multiwfn) for the *Si25* cluster.

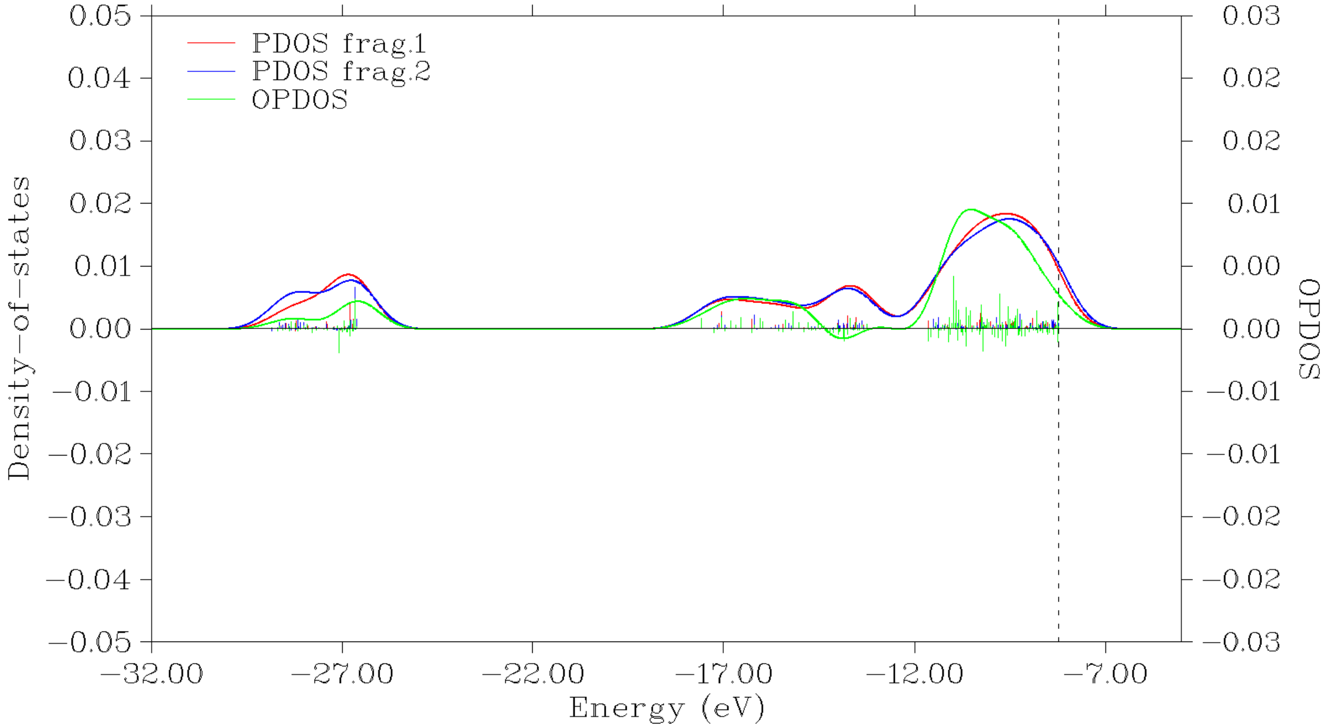

Figure S60. OPDOS analysis of O33-O36 (Multiwfn) for the *Si25* cluster.

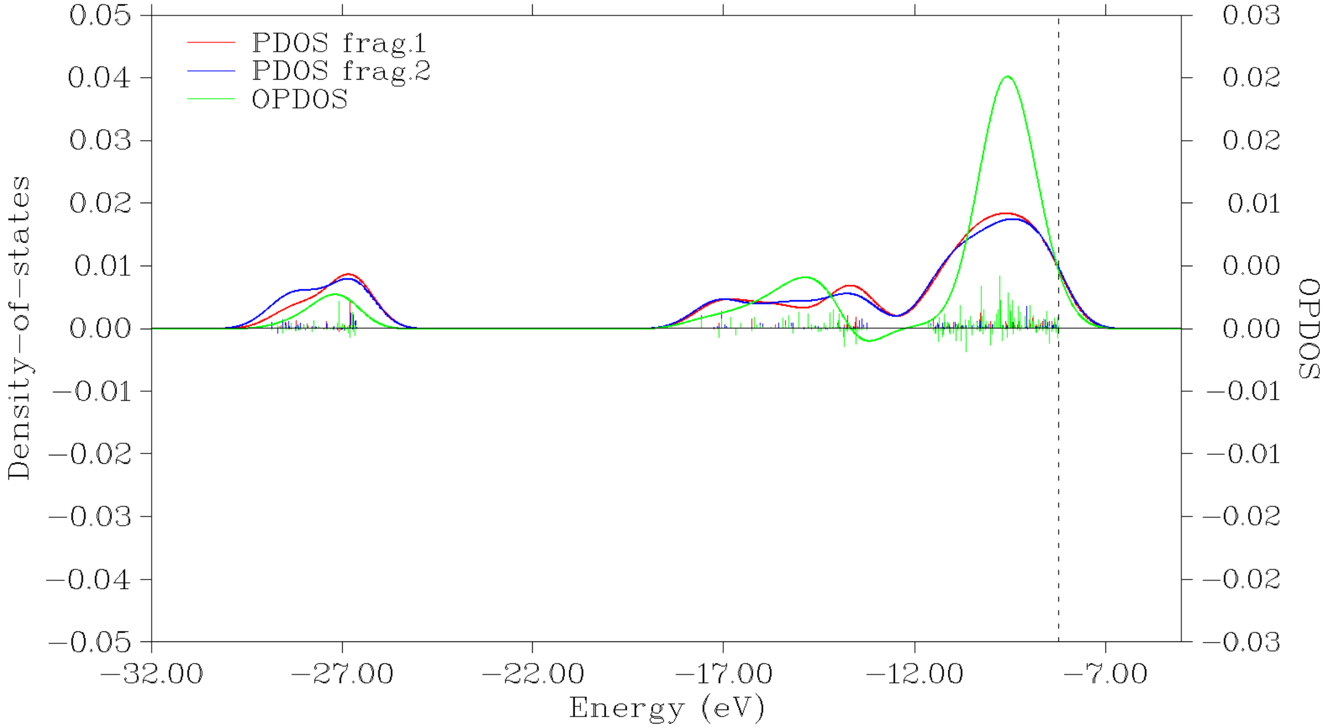

Figure S61. OPDOS analysis of O33-O37 (Multiwfn) for the *Si25* cluster.

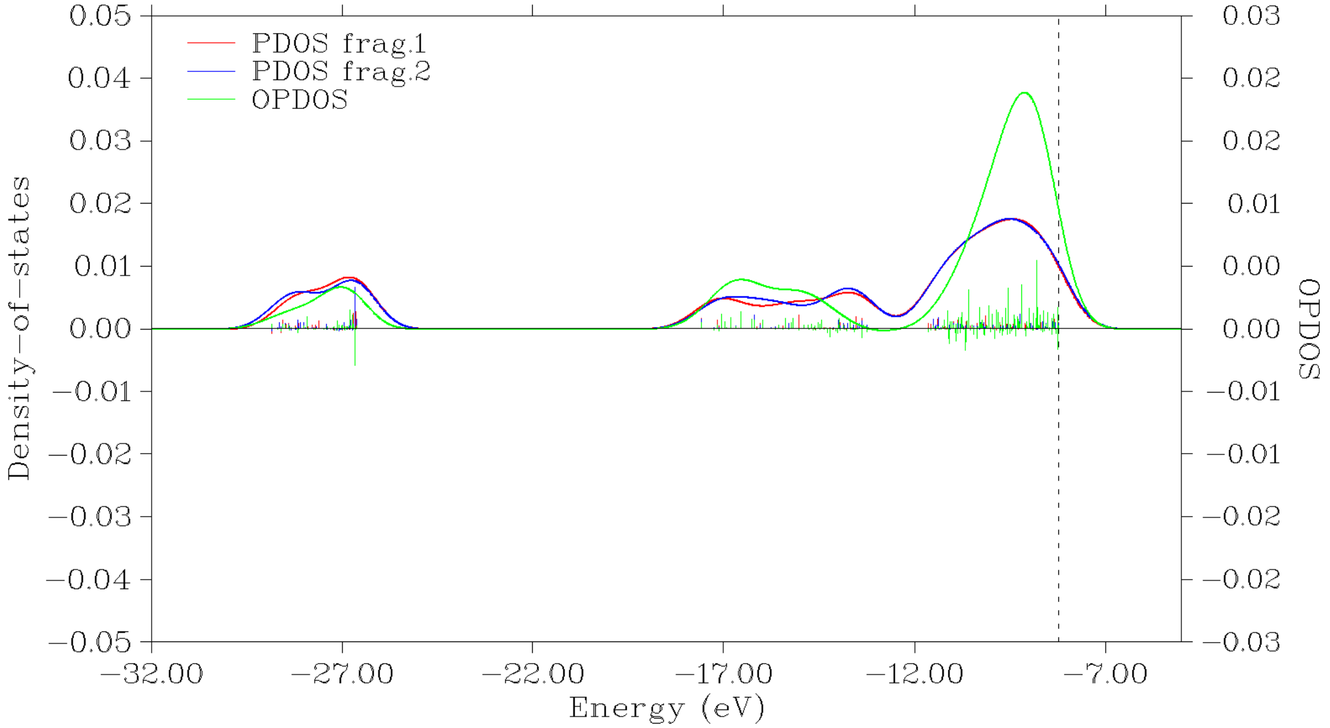

Figure S62. OPDOS analysis of O34-O36 (Multiwfn) for the *Si25* cluster.

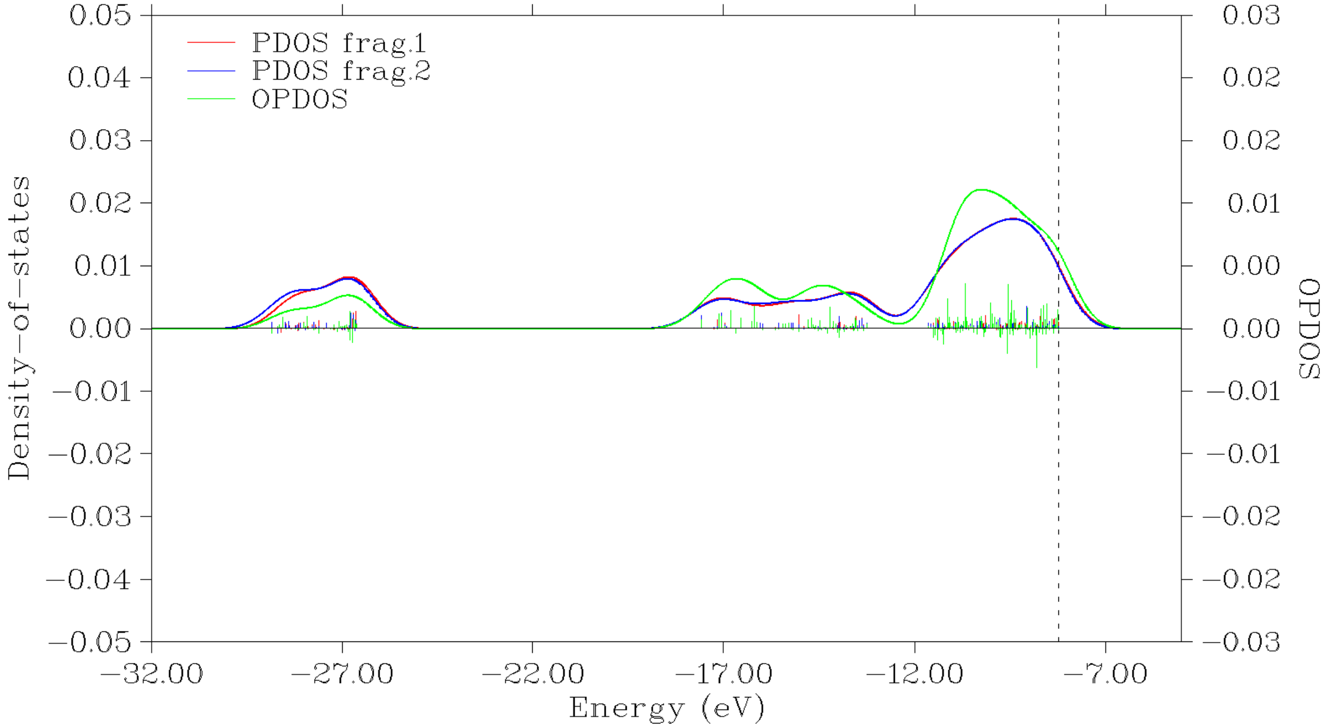

Figure S63. OPDOS analysis of O34-O37 (Multiwfn) for the *Si25* cluster.

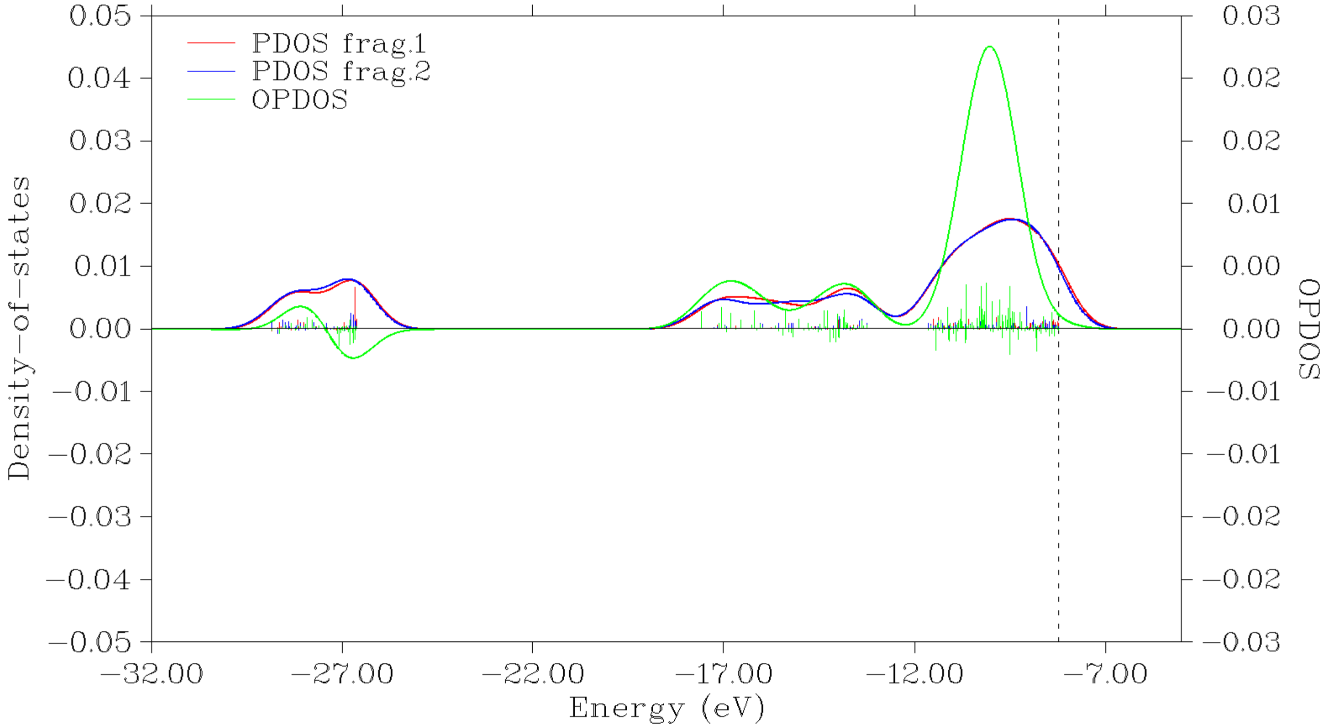

Figure S64. OPDOS analysis of O36-O37 (Multiwfn) for the *Si25* cluster.

Valence Electron Calculations for the Earth's Crust

Table S46. Valence electrons in the Earth's crust. This calculation yields the percentage of crustal valence electrons attributable to the long oxygen-oxygen covalent bonds of silica: 34.5%. The crust is assumed to be the outer 1% of Earth, by mass.

M_{Earth} (kg)	5.9720E+24
M_{crust} = 1% M_{Earth} (kg)	5.9720E+22

element	crustal abundance (%)[9]	mass (kg)	atomic mass	atoms (moles)	valence electrons per atom	total valence electrons (moles)	% crustal valence electrons
oxygen	46.1	2.7531E+22	15.999	1.7208E+24	6	1.0325E+25	72.530
silicon	28.2	1.6841E+22	28.085	5.9965E+23	4	2.3986E+24	16.850
aluminum	8.23	4.9150E+21	26.982	1.8216E+23	3	5.4647E+23	3.839
iron	5.63	3.3622E+21	55.845	6.0207E+22	8	4.8165E+23	3.384
calcium	4.15	2.4784E+21	40.078	6.1839E+22	2	1.2368E+23	0.869
sodium	2.36	1.4094E+21	22.99	6.1305E+22	1	6.1305E+22	0.431
magnesium	2.33	1.3915E+21	24.305	5.7251E+22	2	1.1450E+23	0.804
potassium	2.09	1.2481E+21	39.098	3.1924E+22	1	3.1924E+22	0.224
titanium	0.565	3.3742E+20	47.867	7.0491E+21	4	2.8196E+22	0.198
hydrogen	0.14	8.3608E+19	1.008	8.2944E+22	1	8.2944E+22	0.583
phosphorus	0.105	6.2706E+19	30.974	2.0245E+21	5	1.0122E+22	0.071
manganese	0.095	5.6734E+19	54.938	1.0327E+21	7	7.2288E+21	0.051
fluorine	0.0585	3.4936E+19	18.998	1.8389E+21	7	1.2873E+22	0.090
barium	0.0425	2.5381E+19	137.327	1.8482E+20	2	3.6964E+20	0.003
strontium	0.037	2.2096E+19	87.62	2.5218E+20	2	5.0437E+20	0.004
sulfur	0.035	2.0902E+19	32.06	6.5197E+20	6	3.9118E+21	0.027
carbon	0.02	1.1944E+19	12.011	9.9442E+20	4	3.9777E+21	0.028
zirconium	0.0165	9.8538E+18	91.224	1.0802E+20	4	4.3207E+20	0.003
chlorine	0.0145	8.6594E+18	35.45	2.4427E+20	7	1.7099E+21	0.012
SUM	100.219	5.98508E+22		2.8724E+24		1.42351E+25	100

1.42351E+25 moles of valence electrons x 6.02E+23 = 8.57E+48 or **10^{49} valence electrons** (rounded)

silica	mass (kg)	atomic mass	atoms (moles)	valence electrons per atom	silica valence electrons (moles)	% crustal valence electrons
crustal silica = 59% M_{crust}[10]	3.5235E+22					
silicon in crustal silica	1.6470E+22	28.085	5.8644E+23	4	2.3457E+24	16.479
oxygen in crustal silica	1.8765E+22	15.999	1.1729E+24	6	7.0372E+24	49.436
SUM	3.5235E+22		1.7593E+24		9.3830E+24	65.914
						**65.914%**
see Table S3 for calculated 52.30% valence electrons in O-O						x 0.5230
**long O-O bonds in silica**						**34.47%**
						65.914
see Table S3 for calculated 47.70% valence electrons in Si-O						x 0.4770
Si-O bonds in silica						31.44%

Of the 7.0372E+24 moles of oxygen-based crustal valence electrons in silica, 25% (see reference/note 32 of the manuscript) or **10^{48}** electrons are Möbius aromatic (= 7.0372E+24 x 6.02E+23 x 0.25).

References

[1] Shao, Y.; Gan, Z.; Epifanovsky, E.; Gilbert, A. T. B.; Wormit, M.; Kussmann, J.; Lange, A. W.; Behn, A.; Deng, J.; Feng, X.; Ghosh, D.; Goldey, M.; Horn, P. R.; Jacobson, L. D.; Kaliman, I.; Khaliullin, R. Z.; Kuś, T.; Landau, A.; Liu, J.; Proynov, E. I.; Rhee, Y. M.; Richard, R. M.; Rohrdanz, M. A.; Steele, R. P.; Sundstrom, E. J.; Woodcock III, H. L.; Zimmerman, P. M.; Zuev, D.; Albrecht, B.; Alguire, E.; Austin, B.; Beran, G. J. O.; Bernard, Y. A.; Berquist, E.; Brandhorst, K.; Bravaya, K. B.; Brown, S. T.; Casanova, D.; Chang, C.-M.; Chen, Y.; Chien, S. H.; Closser, K. D.; Crittenden, D. L.; Diedenhofen, M.; DiStasio Jr., R. A.; Do, H.; Dutoi, A. D.; Edgar, R. G.; Fatehi, S.; Fusti-Molnar, L.; Ghysels, A.; Golubeva-Zadorozhnaya, A.; Gomes, J.; Hanson-Heine, M. W. D.; Harbach, P. H. P.; Hauser, A. W.; Hohenstein, E. G.; Holden, Z. C.; Jagau, T.-C.; Ji, H.; Kaduk, B.; Khistyaev, K.; Kim, J.; Kim, J.; King, R. A.; Klunzinger, P.; Kosenkov, D.; Kowalczyk, T.; Krauter, C. M.; Lao, K. U.; Laurent, A. D.; Lawler, K. V.; Levchenko, S. V.; Lin, C. Y.; Liu, F.; Livshits, E.; Lochan, R. C.; Luenser, A.; Manohar, P.; Manzer, S. F.; Mao, S.-P.; Mardirossian, N.; Marenich, A. V.; Maurer, S. A.; Mayhall, N. J.; Neuscamman, E.; Oana, C. M.; Olivares-Amaya, R.; O'Neill, D. P.; Parkhill, J. A.; Perrine, T. M.; Peverati, R.; Prociuk, A.; Rehn, D. R.; Rosta, E.; Russ, N. J.; Sharada, S. M.; Sharma, S.; Small, D. W.; Sodt, A.; Stein, T.; Stück, D.; Su, Y.-C.; Thom, A. J. W.; Tsuchimochi, T.; Vanovschi, V.; Vogt, L.; Vydrov, O.; Wang, T.; Watson, M. A.; Wenzel, J.; White, A.; Williams, C. F.; Yang, J.; Yeganeh, S.; Yost, S. R.; You, Z.-Q.; Zhang, I. Y.; Zhang, X.; Zhao, Y.; Brooks, B. R.; Chan, G. K. L.; Chipman, D. M.; Cramer, C. J.; Goddard III, W. A.; Gordon, M. S.; Hehre, W. J.; Klamt, A.; Schaefer III, H. F.; Schmidt, M. W.; Sherrill, C. D.; Truhlar, D. G.; Warshel, A.; Xu, X.; Aspuru-Guzik, A.; Baer, R.; Bell, A. T.; Besley, N. A.; Chai, J.-D.; Dreuw, A.; Dunietz, B. D.; Furlani, T. R.; Gwaltney, S. R.; Hsu, C.-P.; Jung, Y.; Kong, J.; Lambrecht, D. S.; Liang, W.; Ochsenfeld, C.; Rassolov, V. A.; Slipchenko, L. V.; Subotnik, J. E.; Van Voorhis, T.; Herbert, J. M.; Krylov, A. I.; Gill, P. M. W.; Head-Gordon, M. Advances in molecular quantum chemistry contained in the Q-Chem 4 program package. *Mol. Phys.* **2015**, *113*, 184–215. https://doi.org/10.1080/00268976.2014.952696

[2] Levien, L.; Prewitt, C. T.; Weidner, D. J. Structure and elastic properties of quartz at pressure. *Am. Mineral.* **1980**, *65*, 920–930. https://pubs.geoscienceworld.org/msa/ammin/article-abstract/65/9-10/920/41195

[3] Kirfel, A; Eichhorn, K. Accurate structure analysis with synchrotron radiation. The electron density in Al_2O_3 and Cu_2O. *Acta Cryst.* **1990**, *A46*, 271–284. https://doi.org/10.1107/S0108767389012596

[4] Rettig, S. J.; Trotter, J. Refinement of the Structure of Orthorhombic Sulfur, α-S_8. *Acta Cryst.* **1987**, ***C43***, 2260–2262. https://doi.org/10.1107/S0108270187088152

[5] Gorelsky, S. I. *AOMix: Program for Molecular Orbital Analysis*; version 6.94, **2018.** http://www.sg-chem.net/

[6] Skripnikov, L. *Chemissian*; version 4.67, **2020**. www.chemissian.com

[7] Lu, T.; Chen, F. Multiwfn: a multifunctional wavefunction analyzer. *J. Comput. Chem.* **2012**, *33*, 580–592. https://doi.org/10.1002/jcc.22885

[8] Gaussian 09, Revision D.01, Frisch, M. J.; Trucks, G. W.; Schlegel, H. B.; Scuseria, G. E.; Robb, M. A.; Cheeseman, J. R.; Scalmani, G.; Barone, V.; Mennucci, B.; Petersson, G. A.; Nakatsuji, H.; Caricato, M.; Li, X.; Hratchian, H. P.; Izmaylov, A. F.; Bloino, J.; Zheng, G.; Sonnenberg, J. L.; Hada, M.; Ehara, M.; Toyota, K.; Fukuda, R.; Hasegawa, J.; Ishida, M.; Nakajima, T.; Honda, Y.; Kitao, O.; Nakai, H.; Vreven, T.; Montgomery, J. A., Jr.; Peralta, J. E.; Ogliaro, F.; Bearpark, M.; Heyd, J. J.; Brothers, E.; Kudin, K. N.; Staroverov, V. N.; Kobayashi, R.; Normand, J.; Raghavachari, K.; Rendell, A.; Burant, J. C.; Iyengar, S. S.; Tomasi, J.; Cossi, M.; Rega, N.; Millam, J. M.; Klene, M.; Knox, J. E.; Cross, J. B.; Bakken, V.; Adamo, C.; Jaramillo, J.; Gomperts, R.; Stratmann, R. E.; Yazyev, O.; Austin, A. J.; Cammi, R.; Pomelli, C.; Ochterski, J. W.; Martin, R. L.; Morokuma, K.; Zakrzewski, V. G.; Voth, G. A.; Salvador, P.; Dannenberg, J. J.; Dapprich, S.; Daniels, A. D.; Farkas, Ö.; Foresman, J. B.; Ortiz, J. V.; Cioslowski, J.; Fox, D. J., *Gaussian, Inc.*, Wallingford CT, **2009**. https://gaussian.com/g09citation/

[9] Haynes, W. M. (Editor), CRC Handbook of Chemistry and Physics, 97th Edition, CRC Press, **2016–2017**, section 14–17. http://www.worldcat.org/oclc/961861918

[10] Clarke, F. W.; Washington, H. S. The Composition of the Earth's Crust. USGS Professional Paper 127, Department of the Interior, United States Geological Survey, U.S. Government Printing Office, Washington, DC, **1924**.
https://doi.org/10.3133/pp127

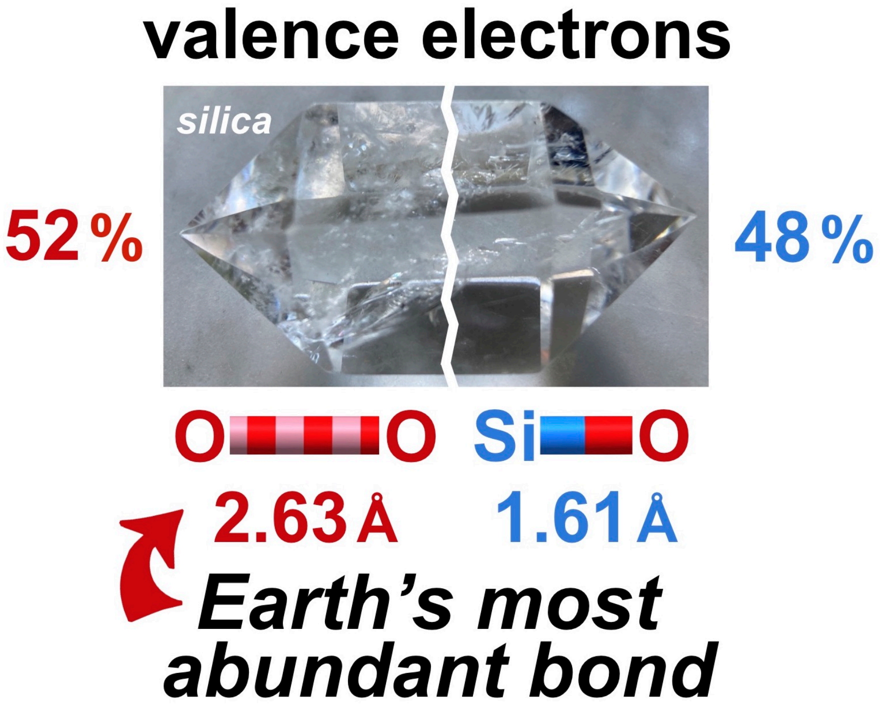

Supplement: Supplementary file 1 [file DataSheet1.pdf]
